# *N*‐Trifluoromethyl Hydrazines, Indoles and Their Derivatives

**DOI:** 10.1002/anie.202004321

**Published:** 2020-05-14

**Authors:** Samir Bouayad‐Gervais, Thomas Scattolin, Franziska Schoenebeck

**Affiliations:** ^1^ Institute of Organic Chemistry RWTH Aachen University Landoltweg 1 52074 Aachen Germany

**Keywords:** fluorine, *N*-CF_3_ hydrazine, *N*-CF_3_ indole, synthesis

## Abstract

Reported herein is the first efficient strategy to synthesize a broad range of unsymmetrical N‐CF_3_ hydrazines, which served as platform to unlock numerous currently inaccessible derivatives, such as tri‐ and tetra‐substituted N‐CF_3_ hydrazines, hydrazones, sulfonyl hydrazines, and valuable N‐CF_3_ indoles. These compounds proved to be remarkably robust, being compatible with acids, bases, and a wide range of synthetic manipulations. The feasibility of RN(CF_3_)‐NH_2_ to function as a directing group in C−H functionalization is also showcased.

## Graphical abstract

**The first synthetic access** to *N*‐trifluoromethyl hydrazines, indoles and their derivatives is described. These compounds proved to be remarkably robust, being compatible with acids, bases, and a wide range of synthetic manipulations.

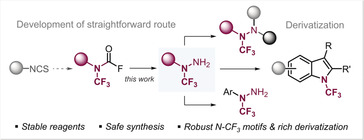

Hydrazines (R_2_N‐NR_2_) are ubiquitous motifs in materials,^1^ pharmaceuticals,^2^ agrochemicals,2d dyes^3^ (Figure 1) as well as enabling functional groups in synthesis, being popular directing groups in catalysis^4^ and valuable precursors to heterocycles (such as indoles),^5^ or tetrazenes. Consequently, there is a significant interest in devising efficient synthetic strategies to novel hydrazine motifs. While mono‐substituted hydrazines (i.e. R‐NH‐NH_2_) are readily accessible via arylation of NH_2_‐NH_2_ (and protected NH_2_‐NH‐PG),^6^ the syntheses of higher substituted, and especially unsymmetrically substituted hydrazines as well as their fluorinated derivatives have been challenging to date.^7^ In this context, a straightforward method to generate trifluoromethylated and unsymmetrically substituted hydrazines would be particularly impactful, owing to the powerful effects of fluorination on the (metabolic) stabilities as well as physical properties of organic molecules.^8^ However, the current methodological repertoire to access *N*‐CF_3_ hydrazines is of limited scope^9^, ^10^ (see Figure 1, middle), relying on harsh photolysis or unselective oxidations with XeF_2_ (to yield **I**) as well as pyrolysis to yield polytrifluoromethylated hydrazines (**III**), or low‐yielding trifluoromethylation of a single class of diazo compounds (to yield **II**), for which the feasibility of Boc deprotection and potential derivatizations has not been demonstrated however.10g

**Figure 1:**
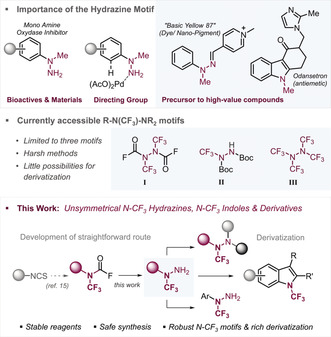
Importance of the hydrazine motif (top), current limited approaches to *N*‐CF_3_ hydrazines (middle) and this work (bottom).

In this context, we envisioned that if we could develop a general method to access pharmaceutically and agrochemically relevant aromatic *N*‐CF_3_ hydrazines, this might potentially allow us to unlock valuable and currently inaccessible *N*‐CF_3_ derivatives, such as *N*‐CF_3_ hydrazones, sulfonyl hydrazines, or indoles. While the former have never been made, *N*‐CF_3_ indoles can currently only be synthesized with strongly basic or oxidizing conditions that limit generality and functional group tolerance, involving either deprotonation of the indole *N*‐H and reaction with gaseous CF_3_I (of unknown efficiency; no yield reported),^11^ or trifluoromethylation of the non‐aromatic indoline precursor, followed by re‐aromatization under highly oxidizing conditions.^12^ Interestingly, the *N*‐deprotonation of indoles, followed by reaction with an electrophilic CF_3_ source, such Togni's reagent, does not yield *N*‐CF_3_ indoles.^13^

We herein describe an efficient strategy to *N*‐CF_3_ hydrazines and showcase their robustness in follow‐up transformations to yield *N*‐CF_3_ indoles, tri‐ and tetra‐substituted hydrazines, sulfonyl hydrazines, acyl hydrazines, as well as their functionalizations via modern catalytic strategies (cross coupling, C−H activation, thiolation, cyanation and borylation).

Our group previously developed a strategy to trifluoromethylate secondary amines,^14^ however, we found that the application of the same protocol to *N′*‐protected hydrazines was not a viable strategy to *N*‐CF_3_ hydrazines, as the key thiocarbamoyl fluoride intermediate did not form. We therefore embarked on developing an alternative strategy: building instead on our previous development of a direct synthetic route to *N*‐CF_3_ carbamoyl fluorides from isothiocyanates (R‐NCS),^15^ we set out to explore whether these *N*‐CF_3_ carbamoyl fluorides could be converted to the corresponding azides and would then potentially rearrange to the corresponding *N*‐CF_3_ hydrazines (see Figure 2). Such an envisioned Curtius‐type rearrangement^16^ is unknown for *N*‐CF_3_ carbonyl compounds; certainly the key *N*‐CF_3_ migration (from **II** to **III**, see Figure 2) is not free of challenges, as fluoride elimination from the transient and partially negatively charged *N*‐CF_3_ could alternatively take place.

**Figure 2:**
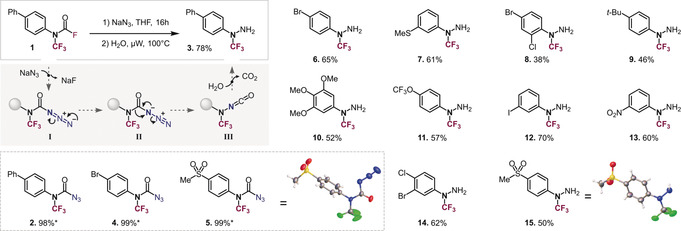
Proposed mechanism for the formation of hydrazines; *yield of carbamoyl azides after filtration; prepared *N*‐CF_3_ hydrazines (**3**, **6**–**15**) and X‐ray structures.^17^

We initially set out to prepare the biphenyl carbamoyl fluoride **1** from the corresponding R‐NCS compound. To our delight, when we subsequently subjected sodium azide to **1** in THF for 16 hours at room temperature, we successfully formed the corresponding carbamoyl azide **2**, which we isolated by filtration over celite in quantitative (98 %) yield (see Figure 2). We unambiguously confirmed the structural integrity of the *N*‐CF_3_ carbamoyl azide by X‐ray crystallographic analysis (see **5**, Figure 2). With the key precursor in hand, we subsequently tested its propensity to undergo the envisioned Curtius‐type rearrangement. To our delight, after examining various conditions, we uncovered that *N*‐CF_3_ carbamoyl azide **2** forms the corresponding *N*‐CF_3_ hydrazine **3** after heating at 100 °C for 3 days in aqueous THF^18^ in 77 % yield.

With the goal to potentially decrease the reaction time, we next turned to microwave‐based heating and found that the application of constant temperature (100 °C) using power modulation led to a 24‐fold rate increase, shortening the reaction time to only 3 h at 100 °C to reach the same yield of **3**. With this promising protocol in hand, we subsequently set out to explore the wider scope.

With a view towards potential follow‐up diversifications, we next studied a derivative bearing an aromatic bromide (**4**). After reacting the corresponding bromo *N*‐CF_3_ carbamoyl fluoride with NaN_3_ for 16 h at room temperature, we filtered and subsequently diluted the reaction mixture with THF and water, before subjecting to microwave irradiation. With this minimally disruptive reaction sequence free of any elaborate work‐up or column chromatography, we were able to obtain **6** in 65 % yield. However, the *N*‐CF_3_ carbamoyl azides proved to be robust and can also be isolated and purified by column chromatography. Alternative halides other than C‐Br, that is, iodide (**12**), chloride and polyhalogenated compounds (**8**, **14**) were similarly well tolerated (see Figure 2). Aside from being of value on their own to induce additional function via halogen bonding,^19^ which has become increasingly important in medicinal and material science,^20^ these halogen sites could serve as ideal handles for further diversification via established cross‐coupling methodology. In our tests for compatibility of alternative functionalities, we found the protocol to be rather general, tolerating electron‐rich as well as electron‐deficient arenes. The electron‐withdrawing nitro‐ (**13**), sulfone‐ (**15**) and even OCF_3_ (**11**) groups were equally compatible as the donating methoxy (**10**), sulfide (**7**) or alkyl substituents (**9**). In this context, *meta*‐ and *para*‐substitution were generally well tolerated, whereas the presence of *ortho*‐substituents considerably slows the reaction, giving for example, **8** in only moderate yield (=38 %) after microwave irradiation for 36 h (along with unreacted carbamoyl azide). Non‐aromatic carbamoyl fluorides, that is, alkyl derivatives, did not result in the corresponding hydrazines.

We envisioned that instead of exploring modified conditions to better tolerate *ortho*‐substituents in the Curtius‐type rearrangement, a more powerful strategy might potentially be to build on the established ability of hydrazines to function as the directing group in C−H functionalizations.^21^ If the novel *N*‐CF_3_ hydrazines were similarly able to direct transition metals and simultaneously tolerate modern C−H activation catalysis conditions (which is currently unknown), then this would be a powerful diversification strategy to access a number of different derivatives from a common hydrazine precursor.

Pleasingly, the application of Rh‐catalyzed C−H activation21b on hydrazines **6** and **9** allowed us to functionalize the *ortho* position upon reaction with an alkyne to give the corresponding olefins **20** and **21** (see Figure 3), showcasing that the CF_3_ substituent does not impede the hydrazine's ability to function as a directing group. A strong feature of this transformation is the orthogonality of rhodium catalysis to typical palladium‐catalyzed cross‐coupling, as the C‐Br moiety in **20** remained untouched, which leaves additional opportunities for diversification. With this in mind, we next examined the compatibility of the *N*‐CF_3_ motif with Pd‐catalyzed cross‐coupling conditions.

**Figure 3:**
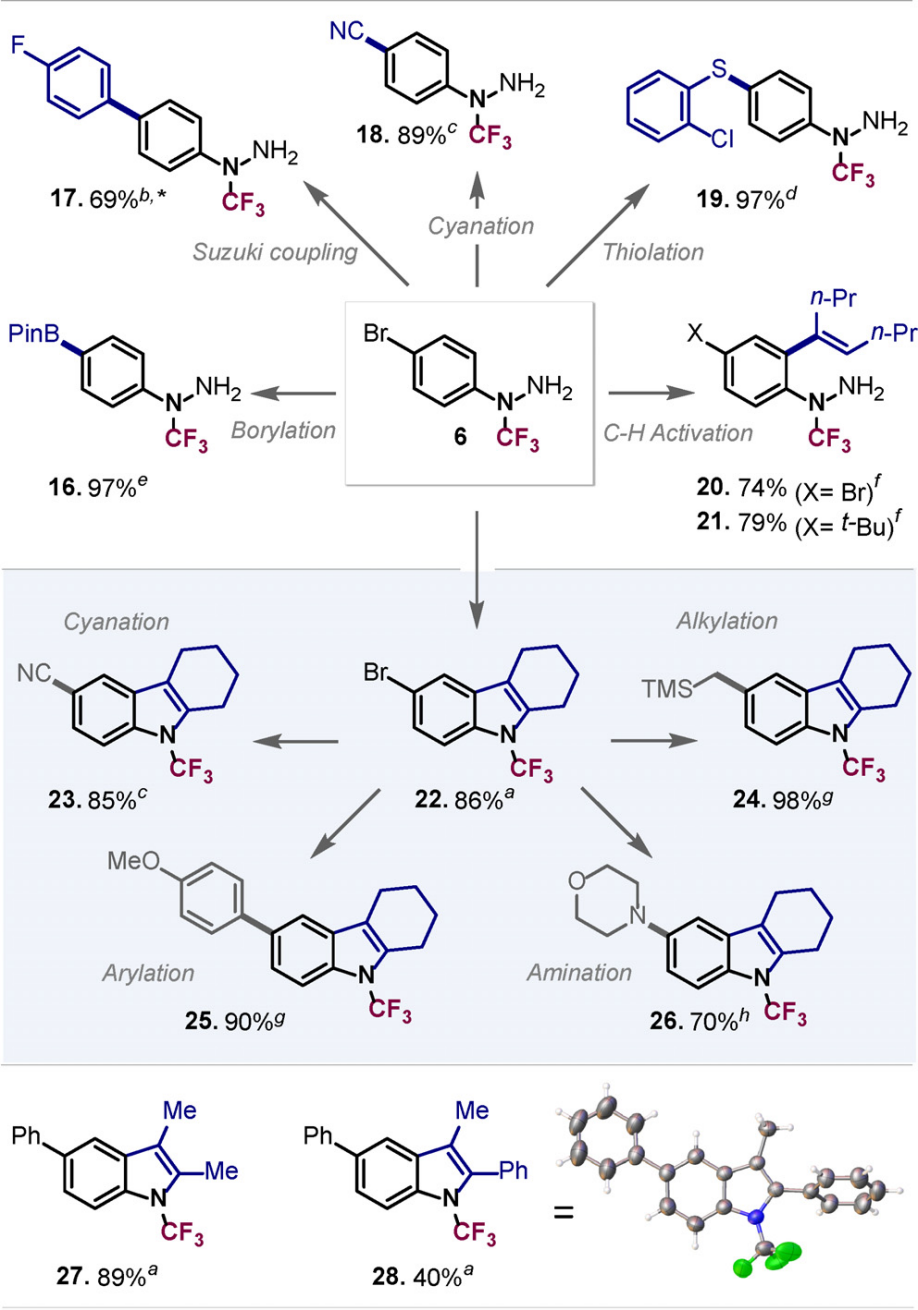
Derivatization of *N*‐CF_3_ hydrazines and indoles: a) ketone (2.0 equiv), H_2_SO_4_, MeOH, 80 °C, 4 h; b) ArBpin (1.2 equiv), KOAc (10.0 equiv), Pd(PPh_3_)_4_ (10 mol %), H_2_O/toluene, 120 °C, 16 h; c) NaCN (1.1 equiv), Pd_2_dba_3_/P*t*Bu_3_ (1.0 mol %), THF/MeCN, 70 °C, 16 h; d) Na‐thiolate (1.2 equiv), [Pd^I^(*μ*‐I)(P*t*Bu_3_)]_2_ (5 mol %), toluene, 70 °C, 16 h; e) (BPin)_2_ (1.1 equiv), KOAc (3.0 equiv), PdCl_2_(dppf) ⋅DCM (3 mol %), H_2_O/toluene, 100 °C, 16 h; f) alkyne (2.0 equiv), AcOH, AgSbF_6_, [RhCp*Cl_2_]_2_ (2 mol %), MeOH, r.t., 72 h; g) Grignard reagent (1.5 equiv), [Pd^I^(*μ*‐I)(P*t*Bu_3_)]_2_ (2.5 mol %), toluene, r.t., 10 min; h) amine (1.2 equiv), Cs_2_CO_3_ (2.6 equiv), Pd(OAc)_2_/BINAP (10 mol %), toluene 120 °C, 16 h.^17^ *Yield of **17** includes a small amount of inseparable impurity.

Typical Suzuki cross‐coupling conditions were found to be well tolerated, giving arylated **17** in 69 % yield. Similarly, C−S bond formation under Pd^I^ dimer catalysis^22^ and even the usually sensitive cyanation^23^ proceeded efficiently and gave **19** and **18**, respectively, in high yields. To our delight, the introduction of B‐Pin (**16**) was also possible, which offers additional opportunities for functionalization. As such, the *N*‐CF_3_ hydrazine moiety proved to be compatible with various reaction conditions, including strong base and elevated temperature, suggesting that it is a rather stable and robust motif.

Encouraged by these stability observations, we next set out to tackle the challenge of accessing *N*‐CF_3_ indoles, and explored whether the *N*‐CF_3_ hydrazines could also sustain highly acidic conditions and participate in a Fischer indole synthesis. The application of the standard Fischer indole conditions, that is, using H_2_SO_4_ as a catalyst, methanol as a solvent, and heating at 80 °C,^24^ indeed afforded the *N*‐CF_3_ indoles **22** and **27** in high yields (86–89 %) and phenylindole **28**, the X‐ray crystallographic analysis of which unambiguously confirmed the structure (see Figure 3).

The *N*‐CF_3_ bond length of **28** in the solid state was determined to be 1.377 Å, which is slightly shorter than similar *N*‐CH_3_ indoles (1.457 Å). We reproduced this trend also computationally, that is, using DFT optimizations on a variety of compounds, we found fluorination to consistently shorten the N−C bond.^25^ Another noteworthy characteristic was the pyramidalization of the nitrogen, which caused the *N*‐CF_3_ bond to be 13° out‐of‐plane with respect to the indole ring. This is analogous to the stereo‐electronic situation of other fluorinated motifs, for example, PhOCF_3_ or *N*‐CF_3_ amides,8a, 8e, 15, 26 resulting in conformers usually inaccessible for the non‐fluorinated analogs.

With the C‐Br being also present in indole **22**, there is once again opportunity for diversification. Indeed, using dinuclear Pd^I^ catalysis^27^ it was possible to rapidly alkylate (**24**) and arylate (**25**) the indole core in less than 5 minutes at room temperature in excellent yields with the corresponding Grignard reagents as coupling partner (see Figure 3). The Pd^0^/Pd^II^‐catalyzed cyanation and Buchwald–Hartwig amination were also possible, giving **23** and **26**, respectively, in high yields. As such, the indole *N*‐CF_3_ motif seems to not only tolerate highly acidic (Fischer indole synthesis5e, 28) but also strongly nucleophilic and basic conditions (e.g. Grignard), suggesting that they are highly robust entities, which should enable widespread applications.

With the free NH_2_ group in the newly synthesized ArN(CF_3_)‐NH_2_ hydrazines, there is further potential to access higher substituted and unsymmetrical derivatives, and we next set out to also explore this chemical space. We successfully obtained hydrazone **30** in quantitative yield after heating at 80 °C for 3 h in acetic acid (see Figure 4). Hydrazones have uses in catalysis^29^ and material science1a, 1c and are also useful synthetic intermediates, for example, in reductive aminations of amines to ultimately generate monoalkylated amines/hydrazines. Indeed, our mild reduction of **30** generated the *N*‐alkylated hydrazine **31**, leaving the aromatic C‐I and *N*‐CF_3_ groups fully untouched (see Figure 4). Alternatively, we found that the NH_2_ could also be selectively coupled with 3‐chlorobromobenzene via Pd‐catalysis in a Buchwald–Hartwig amination to give **32**. Double arylation did not take place, which showcases that the R*N*(CF_3_)NH_2_ motif can be selectively alkylated and arylated to generate the *N*‐CF_3_ analogs of higher substituted hydrazines. Straightforward carbonylation with a chloroformate gave carbamate **33**, which demonstrates the possibility for protecting the NH_2_, if desired. For this transformation, the stoichiometry had to be carefully controlled to avoid double addition of the chloroformate. Compound **33** was also further acylated to **34** in good yield using similar conditions to generate a fully substituted *N*‐CF_3_ hydrazine with four different substituents. Since sulfonamides and their derivatives are also of significant interest in a medicinal and pharmaceutical context,^30^ we reacted **10** with sulfonyl chloride to prepare the sulfonyl hydrazide **29,** which was fully characterized by X‐ray crystallographic analysis (see Figure 4).

**Figure 4:**
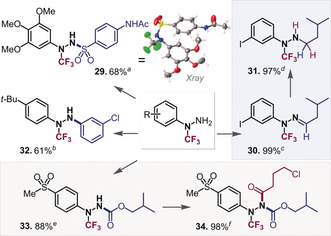
Derivatizations of *N*‐CF_3_ hydrazines on the free NH_2_ moiety: a) sulfonyl‐Cl (1.5 equiv), pyridine (1.5 equiv), DCM, r.t., 16 h; b) ArBr (1.5 equiv), Cs_2_CO_3_ (1.5 equiv), Pd(OAc)_2_/*t*Bu_3_P (20 mol %), toluene, 120 °C, 16 h; c) aldehyde (2.0 equiv), AcOH, MeOH, 80 °C, 4 h; d) BH_3_NMe_3_ (1.0 equiv), HCl (gas), Et_2_O; e) iso‐butyl chloroformate (1.2 equiv), pyridine (1.5 equiv), DCM, r.t., 2 h; f) acylchloride (2.0 equiv), pyridine (2.0 equiv), DCM, r.t. 24 h.^17^

In summary, a straightforward method was developed to readily access a wide range of *N*‐CF_3_ hydrazines. These compounds proved to be very stable under a broad range of conditions, including strong acids, bases, and high temperatures. The hydrazine core was also showcased to readily serve as a platform for the construction of more complex and unsymmetrically substituted *N*‐CF_3_ derivatives, including *N*‐CF_3_ hydrazones, sulfonyl hydrazines, and *N*‐CF_3_ indoles. The feasibility for downstream diversification via modern metal catalysis was also shown. We anticipate numerous applications of this methodology in synthesis, materials sciences, as well as the pharmaceutical and agrochemical arenas to unleash the currently untapped potential of these novel *N*‐trifluoromethylated compounds.

## Conflict of interest

The authors declare no conflict of interest.

## Supporting information

As a service to our authors and readers, this journal provides supporting information supplied by the authors. Such materials are peer reviewed and may be re‐organized for online delivery, but are not copy‐edited or typeset. Technical support issues arising from supporting information (other than missing files) should be addressed to the authors.

Supplementary

## References

[1a] X. Su , I. Aprahamian , Chem. Soc. Rev. 2014, 43, 1963; 24429467 10.1039/c3cs60385g

[1b] G. Men , J.-M. Lehn , J. Am. Chem. Soc. 2017, 139, 2474; 28145690 10.1021/jacs.6b13072

[1c] R. Lygaitis , V. Getautis , J. V. Grazulevicius , Chem. Soc. Rev. 2008, 37, 770; 18362983 10.1039/b702406c

[1d] A. Mahmoodi , M. Ebrahimi , Prog. Org. Coat. 2018, 114, 223.

[2a] R. Narang , B. Narasimhan , S. Sharma , Curr. Med. Chem. 2012, 19, 569; 22204327 10.2174/092986712798918789

[2b] D. K. Kölmel , E. T. Kool , Chem. Rev. 2017, 117, 10358; 28640998 10.1021/acs.chemrev.7b00090 PMC5580355

[2c] C. S. Meira , J. M. dos Santos Filho , C. C. Sousa , P. S. Anjos , J. V. Cerqueira , H. A. Dias Neto , R. G. da Silveira , H. M. Russo , J.-L. Wolfender , E. F. Queiroz , D. R. M. Moreira , M. B. P. Soares , Bioorg. Med. Chem. 2018, 26, 1971; 29523468 10.1016/j.bmc.2018.02.047

[2d] G. Verma , A. Marella , M. Shaquiquzzaman , M. Akhtar , M. Ali , M. Alam , J. Pharm. Bioall. Sci. 2014, 6, 69; 10.4103/0975-7406.129170 PMC3983749 24741273

[2e] E. Nieddu , B. Pollarolo , M. T. Mazzei , M. Anzaldi , S. Schenone , N. Pedemonte , L. J. V. Galietta , M. Mazzei , Arch. Pharm. Chem. Life Sci. 2016, 349, 112; 10.1002/ardp.201500352 26701662

[2f] F. López-Muñoz , C. Álamo , G. Juckel , H.-J. Assion , J. Clin. Psychopharmacol. 2007, 27, 555. 18004120 10.1097/jcp.0b013e3181bb617

[3a] M. P. Elizalde-González , S. A. Lozano-Morales , Mater. Chem. Phys. 2019, 228, 15;

[3b] A. Matoliukstyte , R. Lygaitis , J. V. Grazulevicius , V. Gaidelis , V. Jankauskas , E. Montrimas , Z. Tokarski , N. Jubran , Mol. Cryst. Liq. Cryst. 2005, 427, 107/[419].

[4a] C. Sambiagio , D. Schönbauer , R. Blieck , T. Dao-Huy , G. Pototschnig , P. Schaaf , T. Wiesinger , M. F. Zia , J. Wencel-Delord , T. Besset , B. U. W. Maes , M. Schnürch , Chem. Soc. Rev. 2018, 47, 6603; 30033454 10.1039/c8cs00201k PMC6113863

[4b] Y. Xue , Z. Fan , X. Jiang , K. Wu , M. Wang , C. Ding , Q. Yao , A. Zhang , Eur. J. Org. Chem. 2014, 7481.

[5a] P. Xu , G. Wang , Z. Wu , S. li , C. Zhu , Chem. Sci. 2017, 8, 1303; 28616139 10.1039/c6sc03888c PMC5460599

[5b] C. Song , C. Yang , F. Zhang , J. Wang , J. Zhu , Org. Lett. 2016, 18, 4510; 27584580 10.1021/acs.orglett.6b02103

[5c] S. Han , Y. Shin , S. Sharma , N. K. Mishra , J. Park , M. Kim , M. Kim , J. Jang , I. S. Kim , Org. Lett. 2014, 16, 2494; 24754303 10.1021/ol500865j

[5d] B. W. Boal , A. W. Schammel , N. K. Garg , Org. Lett. 2009, 11, 3458; 19601608 10.1021/ol901383j

[5e] D. L. Hughes , Org. Prep. Proced. Int. 1993, 25, 607.

[6a] M. Wolter , A. Klapars , S. L. Buchwald , Org. Lett. 2001, 3, 3803; 11700143 10.1021/ol0168216

[6b] P. Ruiz-Castillo , S. L. Buchwald , Chem. Rev. 2016, 116, 12564; 27689804 10.1021/acs.chemrev.6b00512 PMC5070552

[6c] A. DeAngelis , D.-H. Wang , S. L. Buchwald , Angew. Chem. Int. Ed. 2013, 52, 3434; 10.1002/anie.201208544 23404773

[7] U. Ragnarsson , Chem. Soc. Rev. 2001, 30, 205.

[8a] K. Müller , C. Faeh , F. Diederich , Science 2007, 317, 1881; 17901324 10.1126/science.1131943

[8b] S. Purser , P. R. Moore , S. Swallow , V. Gouverneur , Chem. Soc. Rev. 2008, 37, 320; 18197348 10.1039/b610213c

[8c] C. Isanbor , D. O'Hagan , J. Fluorine Chem. 2006, 127, 303;

[8d] O. A. Tomashenko , V. V. Grushin , Chem. Rev. 2011, 111, 4475; 21456523 10.1021/cr1004293

[8e] L. E. Zimmer , C. Sparr , R. Gilmour , Angew. Chem. Int. Ed. 2011, 50, 11860; 10.1002/anie.201102027 21953782

[8f] P. A. Champagne , J. Desroches , J.-D. Hamel , M. Vandamme , J.-F. Paquin , Chem. Rev. 2015, 115, 9073; 25854146 10.1021/cr500706a

[8g] A. Tlili , F. Toulgoat , T. Billard , Angew. Chem. Int. Ed. 2016, 55, 11726; 10.1002/anie.201603697 27467551

[8h] N. A. Meanwell , J. Med. Chem. 2018, 61, 5822. 29400967 10.1021/acs.jmedchem.7b01788

[9a] T. Milcent , B. Crousse , Comptes Rendus Chimie 2018, 21, 771;

[9b] T. Liang , C. N. Neumann , T. Ritter , Angew. Chem. Int. Ed. 2013, 52, 8214; 10.1002/anie.201206566 23873766

[10a] G. H. Sprenger , J. M. Shreeve , J. Am. Chem. Soc. 1974, 96, 1770;

[10b] W. Lutz , W. Sundermeyer , Chem. Ber. 1979, 112, 2158;

[10c] R. Fisher , R. N. Haszeldine , A. E. Tipping , J. Fluorine Chem. 1983, 22, 155;

[10d] R. E. Banks , M. S. Falou , A. E. Tipping , J. Fluorine Chem. 1988, 38, 279;

[10e] G. Newsholme , A. E. Tipping , J. Fluorine Chem. 1994, 68, 39;

[10f] W. Sundermeyer , M. Witz , J. Fluorine Chem. 1986, 34, 251;

[10g] M. Mamone , E. Morvan , T. Milcent , S. Ongeri , B. Crousse , J. Org. Chem. 2015, 80, 1964. 25565083 10.1021/jo502638y

[11a] S. W. C. Cheng , H. Joyce LI , US Patent US20190169171 A1, 2018;

[11b] K. W. Bair , Patent WO 2014164767 A1, 2013;

[11c] K. W. Bair , D. R. Lancia , H. Li , J. Loch , W. Lu , M. W. Martin , D. S. Millan , S. E. Schiller , M. J. Tebbe , Patent WO 2014164749 A1, 2014.

[12] S. J. A. Graven , E. Jesper , F. Bastlund , Patent WO2012131031A1, 2012.

[13] In our tests, deprotonation of 2,3-dimethyl-1*H*-indole and subsequent treatment with Togni's reagent did not yield the corresponding *N*-CF_3_ indole. Electrophilic reactions of indoles with Togni's reagent also do not yield *N*-CF_3_ indoles, see: M. S. Wiehn , E. V. Vinogradova , A. Togni , J. Fluorine Chem. 2010, 131, 951.

[14] T. Scattolin , K. Deckers , F. Schoenebeck , Angew. Chem. Int. Ed. 2017, 56, 221; 10.1002/anie.201609480 PMC6680219 27936300

[15] T. Scattolin , S. Bouayad-Gervais , F. Schoenebeck , Nature 2019, 573, 102. 31485055 10.1038/s41586-019-1518-3

[16a] N. Koga , J. P. Anselme , J. Org. Chem. 1968, 33, 3963;

[16b] M. Kurz , W. Reichen , Tetrahedron Lett. 1978, 19, 1433;

[16c] R. Stollé , J. Prakt. Chem. 1927, 116, 192;

[16d] R. Stollé , H. Nieland , M. Merkle , J. Prakt. Chem. 1927, 117, 185.

[17] CCDC 1989758, 1989759, 1989760 and 1989761 (**5**, **15**, **28** & **29**) contain the supplementary crystallographic data for this paper. These data can be obtained free of charge from The Cambridge Crystallographic Data Centre.

[18] For 0.2 mmol scale, 0.1 mL of water in 0.5 mL of THF was used.

[19] “Halogen Bonding II: Impact on Materials Chemistry and Life Science”: G. R. Pierangelo Metrangolo , Topics in Current Chemistry, Springer, Heidelberg, 2015, p. 359.

[20a] A. Linke , S. H. Jungbauer , S. M. Huber , S. R. Waldvogel , Chem. Commun. 2015, 51, 2040; 10.1039/c4cc09163a 25535842

[20b] S. H. Jungbauer , S. M. Huber , J. Am. Chem. Soc. 2015, 137, 12110; 26329271 10.1021/jacs.5b07863

[20c] R. L. Sutar , S. M. Huber , ACS Catal. 2019, 9, 9622.

[21a] C. R. Reddy , B. Sridhar , B. V. Subba Reddy , ACS Omega 2018, 3, 9746; 31459104 10.1021/acsomega.8b01118 PMC6644781

[21b] S. Zhou , J. Wang , F. Zhang , C. Song , J. Zhu , Org. Lett. 2016, 18, 2427; 27135982 10.1021/acs.orglett.6b00949

[21c] S. Zhou , J. Wang , L. Wang , K. Chen , C. Song , J. Zhu , Org. Lett. 2016, 18, 3806. 27434348 10.1021/acs.orglett.6b01805

[22] T. Scattolin , E. Senol , G. Yin , Q. Guo , F. Schoenebeck , Angew. Chem. Int. Ed. 2018, 57, 12425; 10.1002/anie.201806036 PMC6468269 30014554

[23] A. V. Ushkov , V. V. Grushin , J. Am. Chem. Soc. 2011, 133, 10999. 21699208 10.1021/ja2042035

[24] I.-K. Park , S.-E. Suh , B.-Y. Lim , C.-G. Cho , Org. Lett. 2009, 11, 5454. 19886645 10.1021/ol902250x

[25a] M. J. Frisch, et al., Gaussian 09, Revision D.01, Gaussian, Inc., Wallingford CT (**2013**);

[25b] structures were optimized with ωB97XD/6- 31G(d); for complete details see supporting information.

[26] I. F. Shishkov , H. J. Geise , C. Van Alsenoy , L. V. Khristenko , L. V. Vilkov , V. M. Senyavian , B. Van der Veken , W. Herrebout , B. V. Lokshin , O. G. Garkusha , J. Mol. Struct. 2001, 567–568, 339.

[27] For alkylation and arylations with Pd^I^, see:

[27a] I. Kalvet , G. Magnin , F. Schoenebeck , Angew. Chem. Int. Ed. 2017, 56, 1581; 10.1002/anie.201609635 PMC5299498 28032945

[27b] I. Kalvet , T. Sperger , T. Scattolin , G. Magnin , F. Schoenebeck , Angew. Chem. Int. Ed. 2017, 56, 7078; 10.1002/anie.201701691 PMC5488304 28508520

[27c] S. T. Keaveney , G. Kundu , F. Schoenebeck , Angew. Chem. Int. Ed. 2018, 57, 12573; 10.1002/anie.201808386 PMC6175235 30091504

[27d] M. Mendel , I. Kalvet , D. Hupperich , G. Magnin , F. Schoenebeck , Angew. Chem. Int. Ed. 2020, 59, 2115; 10.1002/anie.201911465 PMC7003813 31733009

[28] B. Robinson , Chem. Rev. 1963, 63, 373.

[29a] A. Ros , R. López-Rodríguez , B. Estepa , E. Álvarez , R. Fernández , J. M. Lassaletta , J. Am. Chem. Soc. 2012, 134, 4573; 22369472 10.1021/ja300308c

[29b] Z. Huang , C. Wang , G. Dong , Angew. Chem. Int. Ed. 2016, 55, 5299; 10.1002/anie.201600912 27001210

[29c] K. Watanabe , T. Mino , C. Hatta , S. Ito , M. Sakamoto , Org. Biomol. Chem. 2015, 13, 11645. 26467698 10.1039/c5ob01959a

[30] C. Zhao , K. P. Rakesh , L. Ravidar , W.-Y. Fang , H.-L. Qin , Eur. J. Med. Chem. 2019, 162, 679. 30496988 10.1016/j.ejmech.2018.11.017 PMC7111228

