# Supplementary material for: N‐Trifluoromethyl Hydrazines, Indoles and Their Derivatives
Source: Angew Chem Int Ed Engl. 2020 May 14;59(29):11908–12. doi: 10.1002/anie.202004321 (PMC7384184; doi:10.1002/anie.202004321)
Supplement: Supplementary file 1 — Supplementary [file ANIE-59-11908-s001.pdf]

Angewandte Chemie
Eine Zeitschrift der Gesellschaft Deutscher Chemiker

Supporting Information

N-Trifluoromethyl Hydrazines, Indoles and Their Derivatives

*Samir Bouayad-Gervais, Thomas Scattolin, and Franziska Schoenebeck**

anie_202004321_sm_miscellaneous_information.pdf

Contents

1. Materials and Methods ... 2
Note on Silver fluoride ... 2
2. Experimental section ... 5
General procedure for the synthesis of *N*-CF_3 carbamoyl azides ... 5
General procedure for the synthesis of *N*-CF_3 hydrazines ... 7
2.1 Procedures for derivatization of *N*-CF_3 hydrazines ... 11
General procedure for C-H activation using *N*-CF_3 hydrazine as a directing group ... 13
General procedure for the synthesis of *N*-CF_3 Indoles ... 15
Derivatization of *N*-CF_3 Indoles ... 17
Procedures for the derivatization of the free NH_2 moiety of *N*-CF_3 hydrazines ... 20
General procedure for the synthesis of *N*-trifluoromethylcarbamic fluorides ... 23
General procedure for the synthesis of isothiocyanates ... 26
3. Crystallographic details ... 28
4. Electrophilic *N*-CF_3 indole synthesis ... 29
5. General Computational details ... 30
5.1 Coordinates ... 32
6. NMR ... 39
Carbamoyl Azides ... 39
Hydrazines ... 44
Derivatized hydrazines ... 61
Indoles ... 70
Derivatized indoles ... 75
N-Substituted Hydrazines-CF_3 ... 81
Carbamic fluorides ... 90
Isothiocyanates ... 99
7. References ... 106

1. Materials and Methods

Techniques

All reactions were performed without any precaution for moisture and oxygen unless stated otherwise. Silver fluoride was stored and weighed inside a glovebox before being brought outside to set up reactions. Liquid reagents, solutions or solvents were added via syringe. Unless otherwise stated, experiments were carried out at room temperature (23 ± 2 °C). The removal of solvents *in vacuo* was achieved using a rotary evaporator (bath temperatures up to 40 °C) at a pressure of 20 mmHg (diaphragm pump), or at 0.1 mmHg (oil pump) on a vacuum line at room temperature.

Reagents and solvents

Commercially available solvents and reagents were used directly as supplied without further purification. Acetonitrile was bought from Fischer Scientific. BTC was purchased from TCI, silver fluoride was purchased from Fluorochem [i.e. ChemPUR in Germany]. 3-Iodophenyl isothiocyanate, and all anilines were commercially available and used as received. All anhydrous solvents were either purchased from Aldrich or dried using a solvent purification system (Innovative Technology PS-MD-5).

Note on Silver fluoride

We observed significant variation of the quality of silver fluoride depending on batches. Based on our tests silver fluoride of lower quality will convert isothiocyanates to carbamic fluorides but might require heating at 50°C and may affect the overall yield and quality. While optimum silver fluoride which we used in previous reports[1] and the current, should be bright orange and a loose powder with some aggregates (left). Lower quality silver fluoride shows a rough appearance and various colors including darker orange (right).

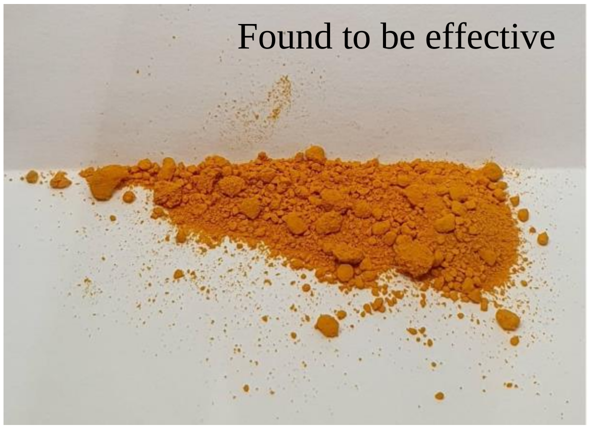

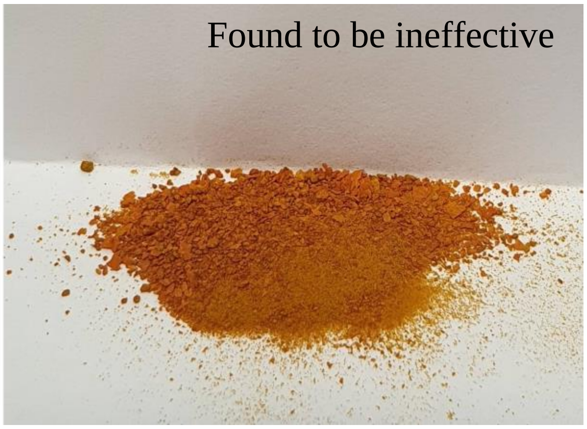

Purification

Column chromatography was performed on Merck silica gel 60 (35 to 70 mesh). Thin layer chromatography was performed on Macherey-Nagel ALUGRAM® Xtra SIL G/UV_{254} plates with unmodified silica and visualized either under UV light or stained with vanillin, PMA, ceric ammonium molybdate or ninhydrin.

Reaction

Microwave reactions were carried in a CEM Discover model 909155 using 10 mL reaction vessels. Temperature was controlled via infrared probe and the pressure release limit was set to 5 bars.

Characterization

All compounds were characterized by NMR (^{1}H, ^{13}C and ^{19}F when applicable), high resolution mass spectrometry (if susceptible to ionization) and FT-IR. ^{1}H, ^{13}C and ^{19}F NMR spectra were recorded on either a Varian VNMRS 600, Varian VNMRS 400 or Varian Mercury 300 spectrometer. Spectra were recorded at ambient temperature in $CDCl_3$ (unless stated otherwise). Chemical shifts are reported in ppm, relative to residual solvent peaks and coupling constants *J* in Hertz (Hz). Signals are described as br = broad, s = singlet, d = doublet, dd = doublet of doublets, t = triplet, q = quartet, qn = quintet, sext = sextet, sept = septet, dsept = doublet of septets and m = multiplet. High-resolution mass spectrometry was performed on a Thermo Scientific LTQ Orbitrap XL spectrometer or on a Finnigan SSQ 7000, EI: 70 eV (EI). Gas chromatography coupled with mass spectrometry (GC-MS) analyses were performed using an Agilent Technologies 5975 series MSD mass spectrometer coupled with an Agilent Technologies 7820A gas chromatograph (with an Agilent 19091s-433 HP-SMS column (30 m x 0.250 µm x 0.25 µm)). The molecular ion

fragment is indicated by an ‘M’. IR spectra were measured on a PerkinElmer Spectrum 100 FT-IR using the universal ATR (UATR) accessory.

2. Experimental section

General procedure for the synthesis of *N*-CF_3 carbamoyl azides

A 4 mL vial was charged with the carbamic fluoride (1 mmol, 1 equiv.) THF (1.25 mL) and sodium azide (78 mg, 1.2 mmol, 1.2 equiv.). The suspension was stirred for 16 h at room temperature. The suspension was then filtered over 1 cm of celite in a glass pipette and rinsed with 500 µL of THF. The combined filtrates were concentrated in vacuo to afford the pure carbamoyl azide.

Note: Isolation of carbamoyl azide is not necessary and follow-up reactions can be conducted without further purification. However, carbamoyl azides are stable and can be isolated if desired as shown by the following examples.

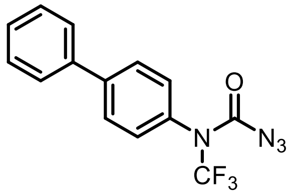

2

[1,1'-Biphenyl]-4-yl(trifluoromethyl)carbamoyl azide 2: The title compound was obtained as a white solid in 98% yield (300 mg) from [1,1'-biphenyl]-4-yl(trifluoromethyl)carbamic fluoride (283 mg) following the general procedure. M.p.: 96-97 °C. ^{1}H NMR (600 MHz, $CDCl_3$) δ 7.67 (d, *J* = 8.4 Hz, 2H), 7.60 (d, *J* = 7.0 Hz, 2H), 7.48 (dd, *J* = 7.7, 7.7 Hz, 2H), 7.41 (dd, *J* = 7.4, 7.4 Hz, 1H), 7.33 (d, *J* = 8.4 Hz, 2H). ^{19}F NMR (564 MHz, $CDCl_3$) δ -55.1 (s, 3F). ^{13}C NMR (151 MHz, $CDCl_3$) δ 155.7, 143.3, 139.7, 133.5, 129.5, 129.1, 128.4, 128.2, 127.4, 119.7 (q, *J* = 263.8 Hz). IR (neat, cm^{-1}): 2157, 1714, 1513, 1483, 1321, 1280, 1194, 1151, 1008, 983, 959, 929, 830, 759, 734, 698. HRMS (+c ESI) calculated for $C_{14}H_9ON_4F_3Na$: 329.06207 $[M+Na]^+$, Found: 329.06189.

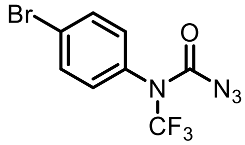

4

(4-Bromophenyl)(trifluoromethyl)carbamoyl azide 4: The title compound was obtained as a beige solid in 99% yield (306 mg) from (4-bromophenyl)(trifluoromethyl)carbamic fluoride (286mg) following the general procedure. M.p.: 60 - 61 °C. ^{1}H NMR (600 MHz, $CDCl_3$) δ 7.59 (d, *J* = 8.6 Hz, 2H), 7.14 (d, *J* = 8.6 Hz, 2H). ^{19}F NMR (564 MHz, $CDCl_3$) δ -55.1 (s, 3F). ^{13}C NMR (151 MHz, $CDCl_3$) δ 155.3, 133.4, 133.1, 130.8, 124.5, 119.4 (q, *J* = 264.1 Hz). IR (neat, cm^{-1}): 2167, 1719, 1596, 1523, 1485, 1402, 1276, 1149, 1068, 1016, 979, 929, 819, 733, 688. MS (70eV, EI): m/z (%): 279.8 (39) [^{79}Br-M – N_2], 282 (38), 241 (68), 239 (59), 219 (36), 221 (30), 159 (100), 155 (75), 75 (45), 69 (29).

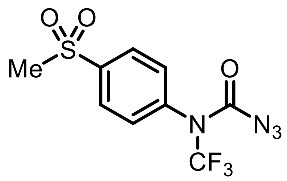

5

(4-(Methylsulfonyl)phenyl)(trifluoromethyl)carbamoyl azide 5: The title compound was obtained as a colorless solid in 99% yield (305 mg) from (4-(methylsulfonyl)phenyl)(trifluoromethyl)carbamic fluoride (285 mg) following the general procedure. M.p.: 76-77 °C. ^{1}H NMR (600 MHz, $CDCl_3$) δ 8.06 (d, J = 8.6 Hz, 2H), 7.49 (d, J = 8.6 Hz, 2H), 3.11 (s, 3H). ^{19}F NMR (564 MHz, $CDCl_3$) δ -54.5 (s, 3F). ^{13}C NMR (151 MHz, $CDCl_3$) δ 154.9, 142.2, 139.0, 130.5, 129.1, 119.4 (q, J = 264.9 Hz), 44.5. IR (neat, cm^{-1}): 3006, 2157, 1718, 1592, 1528, 1492, 1406, 1309, 1276, 1186, 1140, 1085, 963, 929, 832, 770, 734, 696. HRMS (+c ESI) calculated for $C_9H_7N_4O_3F_3{}^{32}SNa$: 331.00832 $[M+Na]^+$, Found: 331.00839. Crystals suitable for X-ray diffraction analysis were grown by diffusion from a mixture of water and THF. CCDC 1989758 contains the supplementary crystallographic data for this compound.

General procedure for the synthesis of *N*-CF_3 hydrazines

A 4 mL vial was charged with the carbamic fluoride (1 mmol, 1 equiv.) THF (1.25 mL) and sodium azide (78 mg, 1.2 mmol, 1.2 equiv.). The suspension was stirred for 16 h at room temperature. The suspension was then filtered over 1 cm of celite in a glass pipette and rinsed with 500 µL of THF directly into a 10 mL microwave reaction vessel. To the carbamoyl azide solution was added water (500 µL) and THF (2.5 mL) and the vessel was sealed. This solution was heated under stirring and microwave irradiation at 100 °C for the indicated time. The aqueous phase was then removed and extracted with CH_2Cl_2. The combined organic phases were adsorbed onto celite and purified by column chromatography on silica gel using the indicated solvent system.

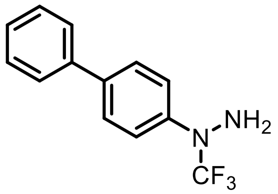

3

1-([1,1'-Biphenyl]-4-yl)-1-(trifluoromethyl)hydrazine 3: The title compound was obtained as a white solid in 78% yield (196 mg) from [1,1'-biphenyl]-4-yl(trifluoromethyl)carbamic fluoride (283 mg) following the general procedure after heating for 3 h and purification by column chromatography using pentane/CH_2Cl_2 (50/50, R_f = 0.31). M.p.: 73-74 °C. ^{1}H NMR (600 MHz, $CDCl_3$) δ 7.62 – 7.56 (m, 4H), 7.48 – 7.44 (m, 2H), 7.7.41 (d, J = 7.8 Hz), 2H), 7.39 – 7.34 (m, 1H), 4.11 (s, 2H). ^{19}F NMR (564 MHz, $CDCl_3$) δ -65.9 (s, 3F). ^{13}C NMR (151 MHz, $CDCl_3$) δ 142.9, 140.3, 139.6, 129.0, 127.9, 127.6, 127.2, 123.7 (q, *J* = 257.8 Hz), 123.7. IR (neat, cm^{-1}): 3362, 3320, 3198, 1800, 1632, 1521, 1485, 1453, 1409, 1339, 1237, 1156, 1123, 1071, 969, 895, 838, 764, 723, 692. HRMS (APCI +) calculated for $C_{13}H_{11}N_2F_3$: 252.08688 [M]$^+$, Found: 252.08653.

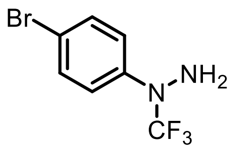

6

1-(4-Bromophenyl)-1-(trifluoromethyl)hydrazine 6: The title compound was obtained as a yellow liquid in 40% yield (102 mg) from (4-bromophenyl)(trifluoromethyl)carbamic fluoride (286 mg) following the general procedure after heating for 4 h and purification by column chromatography using pentane/CH_2Cl_2 (60/40, R_f = 0.34). ^{1}H NMR (600 MHz, $CDCl_3$) δ 7.50 -7.60 (m, 2H), 7.21 (d, *J* = 7.9 Hz, 2H), 4.06 (s, 2H). ^{19}F NMR (564 MHz, $CDCl_3$) δ -66.1 (s, 3F). ^{13}C NMR (151 MHz, $CDCl_3$) δ 142.6, 132.3, 124.8 (d, *J* = 2.0 Hz), 123.4 (q, *J* = 258.2 Hz), 119.8. IR (neat, cm^{-1}): 3378, 2327, 2090, 1618, 1489, 1402, 1305, 1247, 1209, 1141, 1075, 1010, 941, 865, 825, 759, 717, 676. HRMS (EI) calculated for $C_7H_6N_2{}^{79}BrF_3$: 253.96610 [M]$^{\bullet+}$, Found: 253.96565.

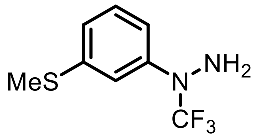

7

1-(3-(Methylthio)phenyl)-1-(trifluoromethyl)hydrazine 7: The title compound was obtained as a dark purple oil in 61% yield (135 mg) from (3-(methylthio)phenyl)(trifluoromethyl)carbamic fluoride (253 mg) following the general procedure after heating for 4 h and purification by column

chromatography using pentane/CH_2Cl_2 (50/50, R_f = 0.29). ^1H NMR (600 MHz, $CDCl_3$) δ 7.27 (dd, *J* = 8.0, 8.0 Hz, 1H), 7.24 – 7.20 (m, 1H), 7.15 – 7.08 (m, 2H), 4.05 (s, 2H), 2.49 (s, 3H). ^{19}F NMR (564 MHz, $CDCl_3$) δ -65.7 (s, 3F). ^{13}C NMR (151 MHz, $CDCl_3$) δ 144.2, 140.0, 129.5, 124.5, 123.6 (q, *J* = 257.8 Hz), 121.5, 119.8, 15.9. IR (neat, cm^{-1}): 3540, 3351, 3219, 2999, 2924, 2160, 1786, 1698, 1584, 1477, 1432, 1234, 1145, 1084, 962, 869, 779, 752, 690. HRMS (APCI+) calculated for $C_8H_{10}F_3N_2{}^{32}S$: 223.05113 $[M+H]^+$, Found: 223.05091.

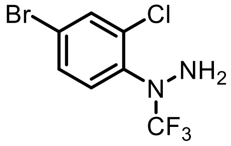

8

1-(4-Bromo-2-chlorophenyl)-1-(trifluoromethyl)hydrazine 8: The title compound was obtained as a green liquid in 38% yield (110 mg) from (4-bromo-2-chlorophenyl)(trifluoromethyl)carbamic fluoride (320 mg) following the general procedure after heating for 36 h and purification by column chromatography using pentane/CH_2Cl_2 (50/50, R_f = 0.51). ^1H NMR (600 MHz, $CDCl_3$) δ Rotamer A (80%): 7.64 (d, *J* = 2.2 Hz, 1H), 7.43 (dd, *J* = 8.5, 2.2 Hz, 1H), 7.28 (d, *J* = 8.5 Hz, 1H), 4.12 (s, 2H); Rotamer B (20%): 7.38 (d, *J* = 2.2 Hz, 1H), 7.16 (dd, *J* = 8.5, 2.2 Hz, 1H), 6.64 (d, *J* = 8.5 HZ, 1H), 4.06 (s, 2H). ^{19}F NMR (564 MHz, $CDCl_3$) δ -64.7 (s, 3F). ^{13}C NMR (151 MHz, $CDCl_3$) δ Rotamer A: 139.6, 135.5, 131.0, 127.6, 123.0 (q, *J* = 257.2 Hz), 121.9, 116.8; Rotamer B: 142.1, 133.8, 131.6, 130.5, 123.0 (q, *J* = 257.2 Hz), 119.9, 109.3. IR (neat, cm^{-1}): 3377, 1620, 1578, 1476, 1379, 1318, 1286, 1243, 1210, 1084, 942, 868, 818, 789, 718. HRMS (APCI+) calculated for $C_7H_5N_2{}^{79}Br^{35}ClF_3$: 287.92767 $[M]^+$, Found: 287.92644.

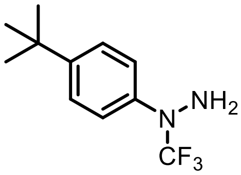

9

1-(4-(*tert*-Butyl)phenyl)-1-(trifluoromethyl)hydrazine 9: The title compound was obtained as a yellow liquid in 46% yield (107 mg) from (4-(*tert*-butyl)phenyl)(trifluoromethyl)carbamic fluoride (263 mg) following the general procedure after heating for 4 h and purification by column chromatography using pentane/CH_2Cl_2 (60/40, R_f = 0.26). ^1H NMR (600 MHz, $CDCl_3$) δ 7.41 – 7.38 (m, 2H), 7.27 (d, *J* = 8.7 Hz, 2H), 4.04 (br, 2H), 1.32 (s, 9H). ^{19}F NMR (564 MHz, $CDCl_3$) δ -66.1 (s, 3F). ^{13}C NMR (151 MHz, $CDCl_3$) δ 150.0, 141.3, 126.2, 123.8 (q, *J* = 257.3 Hz), 123.5 (d, *J* = 1.8 Hz), 34.6, 31.4. IR (neat, cm^{-1}): 3370, 2963, 2908, 2872, 2159, 1786, 1619, 1512, 1468, 1397, 1366, 1312, 1253, 1210, 1143, 1111, 1077, 1019, 943, 873, 835, 745, 697. HRMS (EI) calculated for $C_{11}H_{15}N_2F_3$: 232.11818 $[M]^+$, Found: 232.11844.

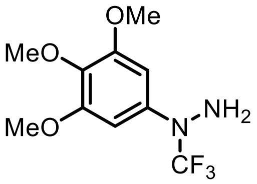

10

1-(Trifluoromethyl)-1-(3,4,5-trimethoxyphenyl)hydrazine 10: The title compound was obtained as an orange solid in 52% yield (138 mg) from (trifluoromethyl)(3,4,5-trimethoxyphenyl)carbamic fluoride (297 mg) following the general procedure after heating for 4 h and purification by column chromatography on neutralized silica gel using pentane/CH_2Cl_2 (60/40, R_f = 0.29). M.p.: 55-56 °C. ^{1}H NMR (600 MHz, $CDCl_3$) δ 6.57 (s, 2H), 4.04 (s, 2H), 3.85 (s, 6H), 3.83 (s, 3H). ^{19}F NMR (564 MHz, $CDCl_3$) δ -66.1 (s, 3F). ^{13}C NMR (151 MHz, $CDCl_3$) δ 153.5, 139.6, 137.2, 123.7 (q, *J* = 257.5 Hz), 102.1, 61.0, 56.3. IR (neat, cm^{-1}): 3368, 2943, 2840, 1594, 1504, 1459, 1420, 1307, 1229, 1123, 1082, 1046, 1000, 901, 832, 772, 745, 695. HRMS (+c ESI) calculated for $C_{10}H_{13}O_3N_2F_3Na$: 289.07705 $[M+Na]^+$, Found: 289.07690.

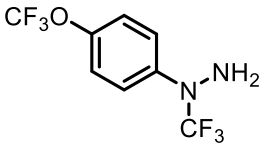

11

1-(4-(Trifluoromethoxy)phenyl)-1-(trifluoromethyl)hydrazine 11: The title compound was obtained as a yellow liquid in 62% yield (180 mg) from (4-(trifluoromethoxy)phenyl)(trifluoromethyl)carbamic fluoride (291 mg) following the general procedure after heating for 5 h and purification by column chromatography using pentane/CH_2Cl_2 (60/40, R_f = 0.53). ^{1}H NMR (600 MHz, $CDCl_3$) δ 7.36 (d, *J* = 8.8 Hz, 2H), 7.22 (d, *J* = 8.8 Hz, 2H), 4.09 (s, 2H). ^{19}F NMR (564 MHz, $CDCl_3$) δ -58.1 (s, 3F), -66.3 (s, 3F). ^{13}C NMR (151 MHz, $CDCl_3$) δ 147.2, 142.1, 124.6, 123.5 (q, *J* = 258.1 Hz), 121.8, 120.6 (q, *J* = 257.7 Hz). IR (neat, cm^{-1}): 1623, 1509, 1211, 1158, 1082, 1018, 945, 846, 811, 680. HRMS (APCI+) calculated for $C_8H_7F_6N_2O$: 261.04571 $[M+H]^+$, Found: 261.04502.

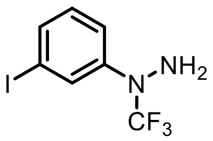

12

1-(3-Iodophenyl)-1-(trifluoromethyl)hydrazine 12: The title compound was obtained as a yellow liquid in 70% yield (211 mg) from (3-iodophenyl)(trifluoromethyl)carbamic fluoride (333 mg) following the general procedure after heating for 3 h and purification by column chromatography using pentane/CH_2Cl_2 (60/40, R_f = 0.30). ^{1}H NMR (600 MHz, $CDCl_3$) δ 7.71 (s, 1H), 7.56 (d, *J* = 7.9 Hz, 1H), 7.30 (d, *J* = 8.2 Hz, 1H), 7.08 (dd, *J* = 8.0, 8.0 Hz, 1H), 4.06 (s, 2H). ^{19}F NMR (564 MHz, $CDCl_3$) δ -65.7 (s, 3F). ^{13}C NMR (151 MHz, $CDCl_3$) δ 144.7, 135.3, 132.0 (d, *J* = 2.1 Hz), 130.6, 123.4 (q, *J* = 258.4 Hz), 122.2 (q, *J* = 2.1 Hz), 94.1. IR (neat, cm^{-1}): 3373, 2334, 2088, 1895, 1582, 1472, 1421, 1242, 1206, 1143, 1081, 991, 956, 867, 776, 691. HRMS (APCI+) calculated for $C_7H_7F_3IN_2$: 302.9601 $[M+H]^+$, Found: 302.9597.

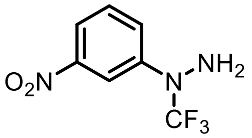

13

1-(3-Nitrophenyl)-1-(trifluoromethyl)hydrazine 13: The title compound was obtained as a yellow liquid in 60% yield (133 mg) from (3-nitrophenyl)(trifluoromethyl)carbamic fluoride (252 mg) following the general

procedure after heating for 4 h and purification by column chromatography using pentane/CH_2Cl_2, 60/40 (50/50, R_f = 0.28). ^{1}H NMR (600 MHz, $CDCl_3$) δ 8.23 (s, 1H), 8.02 (dd, *J* = 8.2, 2.2 Hz, 1H), 7.65 (d, *J* = 8.3 Hz, 1H), 7.51 (t, *J* = 8.2 Hz, 1H), 4.19 (br, 2H). ^{19}F NMR (564 MHz, $CDCl_3$) δ -65.4 (s, 3F). ^{13}C NMR (151 MHz, $CDCl_3$) δ 148.7, 144.4, 129.8, 127.4 (q, *J* = 2.6 Hz), 123.1 (q, *J* = 253.1 Hz), 120.1, 116.7 (q, *J* = 2.5 Hz). IR (neat, cm^{-1}): 3386, 3098, 2923, 2325, 2086, 1902, 1626, 1528, 1483, 1347, 1246, 1213, 1145, 1081, 979, 888, 847, 802, 768, 738, 687. HRMS (+c ESI) calculated for $C_7H_6O_2N_3F_3Na$: 244.03043 $[M+Na]^+$, Found: 244.03040.

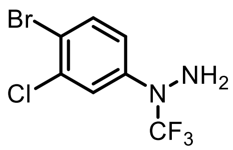

14

1-(4-Bromo-3-chlorophenyl)-1-(trifluoromethyl)hydrazine 14: The title compound was obtained as a blue liquid in 62% yield (180 mg) from (4-bromo-3-chlorophenyl)(trifluoromethyl)carbamic fluoride (320 mg) following the general procedure after heating for 5 h and purification by column chromatography using pentane/CH_2Cl_2 (60/40, R_f = 0.30). ^{1}H NMR (600 MHz, $CDCl_3$) δ 7.57 (d, *J* = 8.7 Hz, 1H), 7.47 (d, *J* = 2.7 Hz, 1H), 7.11 (dd, *J* = 8.7, 1.5 Hz, 1H), 4.07 (br, 2H). ^{19}F NMR (564 MHz, $CDCl_3$) δ -65.7 (s, 3F). ^{13}C NMR (151 MHz, $CDCl_3$) δ 143.5, 135.0, 133.9, 124.3, 123.2 (q, *J* = 258.9 Hz), 121.8, 119.2. IR (neat, cm^{-1}): 3383, 2163, 1622, 1588, 1562, 1468, 1384, 1244, 1209, 1138, 1084, 1017, 966, 866, 814, 707, 664. MS (70eV, EI): m/z (%): 290 [^{35}Cl,^{81}Br-M] (62), 288 (51) [^{35}Cl,^{79}Br-M], 255 (25), 252 (20), 221 (100), 219 (80), 193 (25), 112 (47), 75 (46), 69 (44).

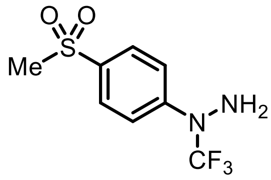

15

1-(4-(Methylsulfonyl)phenyl)-1-(trifluoromethyl)hydrazine 15: The title compound was obtained as a colorless solid in 50% yield (127 mg) from (4-(methylsulfonyl)phenyl)(trifluoromethyl)carbamic fluoride (285 mg) following the general procedure after heating for 4 h and purification by column chromatography using CH_2Cl_2/Et_2O (15/1, R_f = 0.45). M.p.: 75-76 °C. ^{1}H NMR (600 MHz, $CDCl_3$) δ 7.89 (d, *J* = 8.8 Hz, 2H), 7.51 (d, *J* = 8.8 Hz, 2H), 4.19 (s, 2H), 3.04 (s, 3H). ^{19}F NMR (564 MHz, $CDCl_3$) δ -64.0 (s, 3F). ^{13}C NMR (151 MHz, $CDCl_3$) δ 147.8, 136.0, 128.6, 123.0 (q, *J* = 259.7 Hz), 120.4 (d, *J* = 2.8 Hz), 44.8. IR (neat, cm^{-1}): 3399, 3013, 2930, 2159, 1595, 1499, 1282, 1211, 1186, 1142, 1073, 967, 938, 872, 832, 779, 713, 961. HRMS (EI) calculated for $C_8H_9N_2O_2F_3{}^{32}SNa$: 277.02290 $[M]^+$, Found: 277.02292. Crystals suitable for X-ray diffraction analysis were grown by slow evaporation from a solution of pentane and diethyl ether. CCDC 1989759 contains the supplementary crystallographic data for this compound.

2.1 Procedures for derivatization of *N*-CF_3 hydrazines

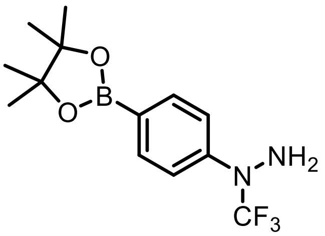

16

1-(4-(4,4,5,5-Tetramethyl-1,3,2-dioxaborolan-2-yl)phenyl)-1-(trifluoromethyl)hydrazine 16: Under argon atmosphere a vial was charged with 1-(4-bromophenyl)-1-(trifluoromethyl)hydrazine (51 mg, 0.2 mmol, 1 equiv.), bis(pinacolato)diboron (56 mg, 0.22 mmol, 1.1 equiv.) and potassium acetate (59 mg, 0.6 mmol, 3 equiv.). A solution of Pd(dppf)•CH_2Cl_2 (5 mg, 0.006 mmol, 3 mol%) in DMF (850 μL) was then added to the solids. The reaction mixture was then stirred at 100°C for 16 h. The obtained mixture was allowed to cool to room temperature, was diluted with EtOAc and washed (2x) with 10% NH_4Cl and water (1x). The organic phase was dried over Na_2SO_4, concentrated *in vacuo* and purified. The compound was obtained as a light brown oil in 96% yield (58 mg) after column chromatography on neutralized silica using pentane/CH_2Cl_2 (50/50, R_f = 0.30). ^{1}H NMR (600 MHz, $CDCl_3$) δ 7.80 (d, *J* = 8.5 Hz, 2H), 7.32 (d, *J* = 7.9 Hz, 2H), 4.07 (s, 2H), 1.34 (s, 12H). ^{19}F NMR (564 MHz, $CDCl_3$) δ -65.2 (s, 3F). ^{13}C NMR (151 MHz, $CDCl_3$) δ 162.6, 146.1, 135.8, 123.5 (q, *J* = 258.0 Hz), 121.4 (d, *J* = 2.0 Hz), 84.0, 25.0. IR (neat, cm^{-1}): 3387, 2982, 2330, 1671, 1608, 1466, 1395, 1358, 1314, 1264, 1207, 1139, 1073, 1018, 939, 853, 823, 737, 659. HRMS (APCI+) calculated for $C_{13}H_{19}BF_3N_2O_2$: 303.1486 $[M+H]^+$, Found: 303.1493.

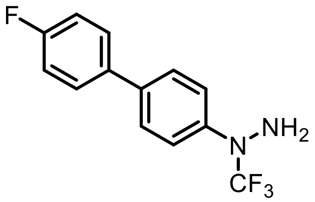

17

1-(4'-Fluoro-[1,1'-biphenyl]-4-yl)-1-(trifluoromethyl)hydrazine 17: To a solution of 1-(4-bromophenyl)-1-(trifluoromethyl)hydrazine (51 mg), 2-(4-fluorophenyl)-pinacolatoborane (53 mg, 0.24 mmol, 1.2 equiv.) and $Pd(PPh_3)_4$ (22 mg, 0.02 mmol, 0.1 equiv.) in toluene (5 mL) was added a solution of potassium acetate (200 mg in 1 mL H_2O, 10 equiv.). The reaction mixture was sealed and heated at 110°C for 16 h. The obtained mixture was allowed to cool to room temperature, was diluted with EtOAc and washed with water (3x). The organic phase was then dried over Na_2SO_4 and concentrated *in vacuo* and purified. The title compound was obtained as an off-white solid in 69% yield (37 mg) after column chromatography using pentane/CH_2Cl_2 60/40 (50/50, R_f = 0.43). M.p.: 34 - 35 °C. ^{1}H NMR (600 MHz, $CDCl_3$) δ 7.56 – 7.50 (m, 4H), 7.40 (d, *J* = 7.8 Hz, 2H), 7.16 – 7.11 (m, 2H), 4.11 (s, 2H). ^{19}F NMR (564 MHz, $CDCl_3$) δ -65.9 (s, 3F), -115.4 (tt, *J* = 8.6, 5.3 Hz, 1F). ^{13}C NMR (151 MHz, $CDCl_3$) δ 162.7 (d, *J* = 246.7), 142.9, 138.5, 136.5 (d, *J* = 3.2 Hz), 128.7 (d, *J* = 8.3 Hz), 127.8, 123.7 (q, *J* = 257.8 Hz), 123.7, 115.9 (d, *J* = 21.1 Hz). IR (neat, cm^{-1}): 3386, 1602, 1555, 1516, 1483, 1304, 1229, 1140, 1082, 1006, 967, 870, 822, 795, 737, 709, 681. MS (70eV, EI): m/z (%): 270 (100) [M], 201 (46), 172 (43), 152 (17), 133 (8), 69 (4).

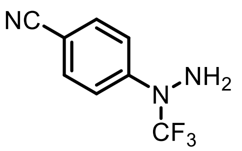

18

4-(1-(Trifluoromethyl)hydrazineyl)benzonitrile 18:

Preparation of catalyst stock solution: In a argon filled glovebox a 20 mL vial was charged with $Pd_2(dba)_3$ (48 mg), P(*t*-Bu)$_3$ (23 mg), anhydrous acetone (60 µL) and anhydrous THF (4 mL). The solution was then stirred for 16 h and then used as a stock solution without further purification.[2]

Cyanation: Under argon, a 1.5 mL vial was charged with 1-(4-bromophenyl)-1-(trifluoromethyl)hydrazine **6** (46 mg, 0.2 mmol, 1 equiv.), anhydrous NaCN (10 mg, 0.21 mmol, 1.06 equiv.), Zn powder (5 mg, 0.015 mmol, 8 mol%) and anhydrous acetonitrile (200 µL). To it was added the catalyst stock solution (80 µL). The reaction was sealed and stirred at 70°C for 15 h under argon atmosphere. The reaction mixture was allowed to cool to room temperature and was diluted with CH_2Cl_2 and stirred for 10 min before filtration over celite. The filtrate was concentrated to dryness *in vacuo* and redissolved in CH_2Cl_2. 100 mg of activated charcoal was added and the mixture was stirred for 2 h before being filtered over silica gel. The filtrate was concentrated *in vacuo* and then purified by column chromatography using pentane/EtOAc (85/15, R_f = 0.25) affording **18** as a parchment colored solid in 89% yield (determined by 1H-NMR quantification due to small amounts of starting material being present). M.p.: 45 - 46 °C. ^{1}H NMR (600 MHz, $CDCl_3$) δ 7.64 -7.60 (m, 2H), 7.43 (d, *J* = 8.0 Hz, 2H), 4.15 (s, 2H). ^{19}F NMR (564 MHz, $CDCl_3$) δ -64.0 (s, 3F). ^{13}C NMR (151 MHz, $CDCl_3$) δ 146.9, 133.1, 122.9 (q, *J* = 259.7 Hz), 120.4, 118.8, 107.8. IR (neat, cm^{-1}): 3381, 2225, 2169, 1609, 1506, 1246, 1085, 950, 832, 756. HRMS (ESI + c) calculated for $C_8H_6N_3F_3Na$: 224.04060 [M+Na]$^+$, Found: 224.04041.

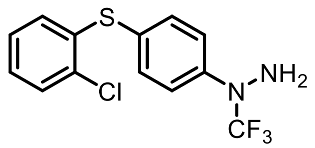

19

1-(4-((2-Chlorophenyl)thio)phenyl)-1-(trifluoromethyl)hydrazine 19: In an argon-filled glovebox a vial was charged with 1-(4-bromophenyl)-1-(trifluoromethyl)hydrazine (51 mg, 0.2 mmol, 1 equiv.), Pd^I iodo-dimer[3] (8.7 mg, 0.001 mmol, 0.05 equiv.) and toluene (1 mL). To the solution was added sodium 2-chlorobenzenethiolate (40 mg, 0.24 mmol, 1.2 equiv.). The vial was sealed and stirred at 80°C for 16 h. The mixture was then filtered through a short celite plug, concentrated *in vacuo* and directly purified. The title compound was obtained as a yellow solid in 98% yield (62 mg) after column chromatography using pentane/CH_2Cl_2 (50/50, R_f = 0.33). M.p.: 181 - 182 °C. ^{1}H NMR (600 MHz, $CDCl_3$) δ 7.43 – 7.38 (m, 3H), 7.34 (d, J = 7.9 Hz, 2H), 7.17 – 7.11 (m, 2H), 7.02 – 6.98 (m, 1H), 4.10 (s, 2H). ^{19}F NMR (564 MHz, $CDCl_3$) δ -65.5 (s, 3F). ^{13}C NMR (151 MHz, $CDCl_3$) δ 143.6, 136.2, 133.9, 133.4, 130.3, 130.1, 129.9, 127.6, 127.4, 123.5 (q, *J* = 258.4 Hz), 123.6 (q, *J* = 2.3 Hz). IR (neat, cm^{-1}): 3377, 1596, 1491, 1450, 1247, 1209, 1141, 1078, 1033, 943, 830, 748, 696, 660. HRMS (ESI + c) calculated for $C_{13}H_{10}N_2{}^{35}ClF_3Na^{32}S$: 341.00975 [M+Na]$^+$, Found: 341.00934.

General procedure for C-H activation using *N*-CF_3 hydrazine as a directing group

In an argon-filled glovebox a vial was loaded with $[RhCp^*Cl_2]_2$ (2.4 mg, 2 mol%) and $AgSbF_6$ (5.6 mg, 8 mol%). It was brought outside and a solution of the hydrazine (0.2 mmol, 1 equiv.) in MeOH (600 μL) was added followed by acetic acid (14 μL, 1.2 equiv.) and the electrophile (2 equiv.). The reaction mixture was sealed and stirred under the exclusion of light (vial was wrapped in aluminum foil) for 36 h at room temperature. The reaction was then diluted with CH_2Cl_2 and filtered through silica. The filtrate was concentrated *in vacuo* before being purified by column chromatography on silica gel using the indicated solvent system.[4]

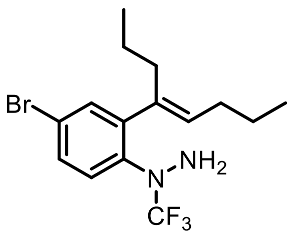

20

(*E*)-1-(4-bromo-2-(oct-4-en-4-yl)phenyl)-1-(trifluoromethyl)hydrazine 20: The title compound was obtained as a yellow oil in 74% yield (54 mg) from 1-(4-bromophenyl)-1-(trifluoromethyl)hydrazine **6** (51 mg, 0.2 mmol, 1 equiv.) and 4-octyne (44 μL, 0.4 mmol, 2 equiv.) following the general procedure for C-H activation after column chromatography on neutralized silica using CH_2Cl_2/pentane (40/60 R_f = 0.43). ^{1}H NMR (600 MHz, $CDCl_3$) δ 7.39 (dd, *J* = 8.5, 2.4 Hz, 1H), 7.32 (d, *J* = 2.4 Hz, 1H), 7.19 (d, *J* = 8.6 Hz, 1H), 5.41 (t, *J* = 7.3 Hz, 1H), 3.85 (s, 2H), 2.39 – 2.42 (m, 2H), 2.17 (q, *J* = 7.4 Hz, 2H), 1.46 (sext, *J* = 7.4Hz, 2H), 1.26 (sext, *J* = 7.4 Hz, 2H), 0.96 (t, *J* = 7.3 Hz, 3H), 0.86 (t, *J* = 7.4 Hz, 3H). ^{19}F NMR (564 MHz, $CDCl_3$) δ -64.2 (s, 3F). ^{13}C NMR (151 MHz, $CDCl_3$) δ 145.4, 140.9, 139.2, 134.3, 132.4, 131.0, 125.6, 123.6 (q, *J* = 256.4 Hz), 121.5, 32.7, 30.3, 23.0, 21.7, 14.2, 14.0. IR (neat, cm^{-1}): 3368, 2959, 2870, 2327, 2102, 1894, 1632, 1584, 1560, 1473, 1386, 1318, 1281, 1235, 1205, 1142, 1071, 939, 880, 821, 729. HRMS (APCI+) calculated for $C_{15}H_{21}N_2F_3{}^{79}Br$: 365.08347 $[M+H]^+$, Found: 365.08395.

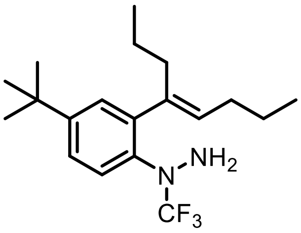

21

(*E*)-1-(4-(*tert*-butyl)-2-(oct-4-en-4-yl)phenyl)-1-(trifluoromethyl)hydrazine 21: The title compound was obtained as a yellow oil in 79% yield (54 mg) from 1-(4-(tert-butyl)phenyl)-1-(trifluoromethyl)hydrazine (46 mg, 0.2 mmol, 1 equiv.) and 4-octyne (44 μL, 0.4 mmol, 2 equiv.) following the general procedure for C-H activation after column chromatography on neutralized silica using pentane (R_f = 0.50). ^{1}H NMR (600 MHz, $CDCl_3$) δ 7.28 (dd, *J* = 8.4, 2.4 Hz, 1H), 7.24 (d, *J* = 8.4 Hz, 1H), 7.15 (d, *J* = 2.3 Hz, 1H), 5.39 (t, *J* = 7.3 Hz, 1H), 3.84 (s, 2H), 2.43 (dd, *J* = 7.8, 7.8 Hz, 2H), 2.19 (q, *J* = 7.4 Hz, 2H), 1.48 (sext, *J* = 7.4 Hz, 2H), 1.32 (s, 9H), 1.27 (dq, *J* = 14.8, 7.4 Hz, 2H), 0.98 (t, *J* = 7.3 Hz, 3H), 0.87 (t, *J* = 7.3 Hz, 3H). ^{19}F NMR (564 MHz, $CDCl_3$) δ -63.8 (s, 3F). ^{13}C NMR (151 MHz, $CDCl_3$) δ 150.9, 142.8, 140.9, 139.3, 131.0, 128.4, 124.9, 124.0 (q, *J* = 255.8 Hz), 123.3, 34.7, 33.1, 31.4, 30.4, 23.2, 21.7, 14.2, 14.0. IR (neat, cm^{-1}): 3365, 2959, 2870, 2326, 2089, 1988, 1625, 1495, 1461, 1397, 1364, 1322, 1245, 1202, 1143,

1071, 942, 879, 827, 728, 662. HRMS (+c ESI) calculated for $C_{19}H_{30}N_2F_3$: 343.23556 $[M + H]^+$, Found: 343.23511.

General procedure for the synthesis of *N*-CF_3 Indoles

A pressure tube was filled with hydrazine (0.2 mmol, 1 equiv.). It was solubilized with a solution of sulfuric acid (33 mg, 0.34 mmol, 1.6 equiv.) in methanol (1 mL). Then a ketone (0.4 mmol, 2 equiv.) was added and the tube was sealed. The reaction mixture was then stirred at 80°C for 4 h. The obtained solution was allowed to cool to room temperature, was diluted with methanol and stirred for 5 minutes together with a spatula tip of sodium bicarbonate. Celite was then added and the reaction mixture was concentrated *in vacuo* before being purified by column chromatography on silica gel using the indicated solvent system.[5]

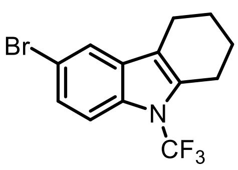

22

6-Bromo-9-(trifluoromethyl)-2,3,4,9-tetrahydro-1*H*-carbazole 22: The title compound was obtained as a colorless solid in 86% yield (55 mg) from 1-(4-bromophenyl)-1-(trifluoromethyl)hydrazine (51 mg) and cyclohexanone (43 µL) following the general procedure for indoles after column chromatography using pentane (R_f = 0.78). M.p.: 45 - 46 °C. ^1H NMR (600 MHz, $CDCl_3$) δ 7.56 (d, *J* = 1.9 Hz, 1H), 7.40 (dd, *J* = 8.7, 1.9 Hz, 1H), 7.32 (dd, *J* = 8.8, 2.0 Hz, 1H), 2.82 (t, *J* = 6.2 Hz, 2H), 2.61 (t, *J* = 6.2 Hz, 2H), 1.92 (dt, *J* = 12.1, 6.1 Hz, 1H), 1.92 (q, *J* = 6.1, 1H), 1.84 (dt, *J* = 11.7, 6.0 Hz, 1H), 1.84 (q, *J* = 6.0 Hz, 1H). ^{19}F NMR (564 MHz, $CDCl_3$) δ -52.5 (s, 3F). ^{13}C NMR (151 MHz, $CDCl_3$) δ 135.1, 135.5, 131.4, 126.2, 121.2, 120.9 (q, *J* = 261.4 Hz), 115.8, 115.6, 113.6 (q, *J* = 4.4 Hz), 23.3 (q, *J* = 3.0 Hz), 23.0, 22.2, 20.8. IR (neat, cm^{-1}): 2943, 2852, 1596, 1460, 1398, 1367, 1329, 1269, 1216, 1191, 1110, 1056, 999, 968, 945, 863, 787, 731, 683. HRMS (APCI+) calculated for $C_{13}H_{12}NF_3{}^{79}Br$: 318.00997 $[M+H]^+$, Found: 318.01015.

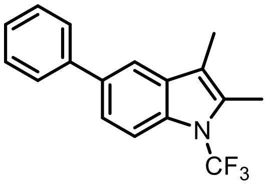

27

2,3-Dimethyl-5-phenyl-1-(trifluoromethyl)-1H-indole 27: The title compound was obtained as a colorless solid in 89% yield (48 mg) from 1-([1,1'-biphenyl]-4-yl)-1-(trifluoromethyl)hydrazine (50 mg) and butanone (36 µL) following the general procedure for indoles after column chromatography using pentane (R_f = 0.52). M.p.: 57 - 58 °C. ^1H NMR (600 MHz, $CDCl_3$) δ 7.68 – 7.66 (m, 3H), 7.62 (d, *J* = 8.6 Hz, 1H), 7.51 – 7.45 (m, 3H), 7.36 (dd, *J* = 7.2, 7.2 Hz, 1H), 2.46 (s, 3H), 2.26 (s, 3H). ^{19}F NMR (564 MHz, $CDCl_3$) δ -51.8 (s, 3F). ^{13}C NMR (151 MHz, $CDCl_3$) δ 141.9, 135.7, 134.2, 131.2, 131.0, 128.9, 127.5, 127.0, 123.8, 121.2 (q, *J* = 261.2 Hz), 117.2, 114.0, 112.5 (q, J = 4.9 Hz), 11.38 (q, *J* = 3.5 Hz), 8.7. IR (neat, cm^{-1}): 2932, 1622, 1463, 1391, 1349, 1233, 1192, 1113, 1065, 1011, 947, 870, 813, 756, 690, 656. HRMS (+c ESI) calculated for $C_{17}H_{15}NF_3$: 290.11511 $[M+H]^+$, Found: 290.11508.

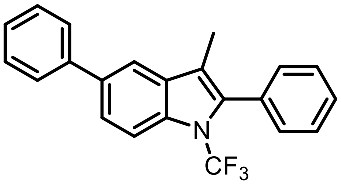

28

3-Methyl-2,5-diphenyl-1-(trifluoromethyl)-1H-indole 28: The title compound was obtained as a beige solid in 40% yield (28 mg) from 1-([1,1'-biphenyl]-4-yl)-1-(trifluoromethyl)hydrazine (50 mg) and propiophenone (53 μL) following the general procedure for indoles after column chromatography using pentane (R_f = 0.33). M.p.: 90 - 91 °C. ^{1}H NMR (600 MHz, $CDCl_3$) δ 7.79 (d, *J* = 1.8 Hz, 1H), 7.74 – 7.69 (m, 3H), 7.62 (dd, *J* = 8.6, 1.8 Hz, 1H), 7.52 – 7.47 (m, 5H), 7.47 – 7.44 (m, 2H), 7.39 (dd, *J* = 7.4, 7.4 Hz, 1H), 2.19 (s, 3H). ^{19}F NMR (564 MHz, $CDCl_3$) δ -50.1 (s, 3F). ^{13}C NMR (151 MHz, $CDCl_3$) δ 141.7, 136.3, 135.3, 134.6, 131.4, 131.0, 130.7, 128.9, 128.7, 128.2, 127.6, 127.1, 124.1, 120.9 (q, *J* = 262.8 Hz), 117.9, 116.7, 113.2 (q, *J* = 4.4 Hz), 9.2. IR (neat, cm^{-1}): 3060, 3033, 2922, 2327, 2094, 1996, 1599, 1463, 1339, 1280, 1222, 1131, 1071, 956, 878, 847, 813, 758, 697. HRMS (ESI + c) calculated for $C_{22}H_{17}NF_3$: 352.13076 $[M + H]^+$, Found: 352.13046. Crystals suitable for X-ray diffraction analysis were grown by slow evaporation from a solution of chloroform. CCDC 1989760 contains the supplementary crystallographic data for this compound.

Derivatization of *N*-CF_3 Indoles

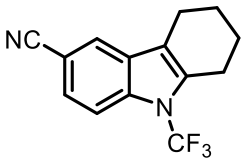

23

9-(Trifluoromethyl)-2,3,4,9-tetrahydro-1*H*-carbazole-6-carbonitrile 23:

Preparation of catalyst stock solution: In a argon filled glovebox a 20 mL vial was charged with $Pd_2(dba)_3$ (48 mg), P(*t*-Bu)$_3$ (23 mg), anhydrous acetone (60 µL) and anhydrous THF (4 mL). The solution was then stirred for 16 h and then used as a stock solution without further purification.[2]

Cyanation: Under argon-atmosphere, a 1.5 mL vial was charged with bromo-9-(trifluoromethyl)-2,3,4,9-tetrahydro-1*H*-carbazole (63 mg, 0.2 mmol), anhydrous NaCN (10 mg, 0.21 mmol, 1.06 equiv.), acetonitrile (200 µL), Zn powder (5 mg, 0.015 mmol, 8 mol%) and catalyst stock solution (80 µL). The reaction mixture was sealed and stirred at 70°C for 15h under argon atmosphere. The reaction mixture was allowed to cool to room temperature and was then diluted with CH_2Cl_2 and stirred for 10 min before being filtered through celite. The filtrate was concentrated to dryness *in vacuo* and redissolved in CH_2Cl_2. Activated charcoal (100 mg) was added and the mixture was stirred for 2 h before filtration over silica gel. The filtrate was concentrated *in vacuo* and adsorbed onto Celite. The title compound was obtained as a colorless solid in 86% yield (45 mg) after column chromatography using 10/90 CH_2Cl_2/pentane (0/100, R_f = 0.1). M.p.: 131-132 °C. ^{1}H NMR (600 MHz, $CDCl_3$) δ 7.74 (s, 1H), 7.57 (dd, *J* = 8.6, 2.0 Hz, 1H), 7.46 (dd, *J* = 8.6, 1.6 Hz, 1H), 2.83 (dd, *J* = 6.2, 6.2 Hz, 2H), 2.65 (dd, *J* = 6.2, 6.2 Hz, 2H), 1.98 -1.92 (m, 2H), 1.89 – 1.83 (m, 2H). ^{19}F NMR (564 MHz, $CDCl_3$) δ -52.5 (s, 3F). ^{13}C NMR (151 MHz, $CDCl_3$) δ 136.3, 136.1, 129.5, 126.6, 123.1, 120.5 (q, *J* = 262.5 Hz), 119.8, 116.2, 112.8 (q, *J* = 4.7 Hz), 105.6, 23.2 (q, *J* = 3.1 Hz), 22.8, 22.0, 20.6. IR (neat, cm^{-1}): 2944, 2856, 2218, 2045, 1619, 1466, 1360, 1140, 970, 901, 809, 736, 682. MS (70eV, EI): m/z (%): 264 (50) [M], 236 (100), 223 (7), 192 (5), 167 (11), 140 (10), 69 (4).

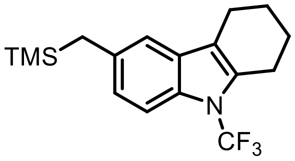

24

9-(Trifluoromethyl)-6-((trimethylsilyl)methyl)-2,3,4,9-tetrahydro-1*H*-carbazole 24: Under inert atmosphere bromo-9-(trifluoromethyl)-2,3,4,9-tetrahydro-1H-carbazole (63 mg, 0.2 mmol, 1 equiv.) and Pd^I iodo-dimer[6] (5 mg, 0.005 mmol, 2.5 mol%) were dissolved in toluene (3 mL) and ((trimethylsilyl)methyl)magnesium bromide solution (600 µL, 0.3 mmol, 1.5 equiv.) was added. The reaction was stirred for 10 min and then exposed to air for 5 min. The reaction mixture was then quenched with sat. NH_4Cl solution. The organic phase was collected, dried over Na_2SO_4, adsorbed onto celite. The title compound was obtained as a colorless oil in 98% yield (64 mg) after column chromatography using pentane (R_f = 0.63). ^{1}H NMR (600 MHz, $CDCl_3$) δ 7.39 (dd, *J* = 8.4, 2.0 Hz, 1H), 7.03 (s, 1H), 6.98 (dd, *J* = 8.4, 1.8 Hz, 1H), 2.80 (t, *J* = 5.5 Hz, 2H), 2.63 (t, *J* = 6.1 Hz, 2H), 2.16 (s, 2H), 1.95 – 1.89 (m, 2H), 1.86

– 1.81 (m, 2H), 0.00 (s, 9H). ^{19}F NMR (564 MHz, $CDCl_3$) δ -52.4 (s, 3F). ^{13}C NMR (151 MHz, $CDCl_3$) δ 134.3, 133.6, 132.2, 129.8, 124.1, 121.2 (q, *J* = 260.7 Hz), 117.0, 115.9, 111.8 (q, *J* = 4.4 Hz), 26.7, 23.4 (q, *J* = 3.0 Hz), 23.2, 22.4, 21.0, -1.7. IR (neat, cm^{-1}): 2943, 2855, 1611, 1469, 1366, 1327, 1302, 1246, 1216, 1192, 1111, 1008, 974, 952, 843, 755, 690. HRMS (+c ESI) calculated for $C_{17}H_{23}NF_3Si$: 326.15464 $[M+H]^+$, Found: 326.15427.

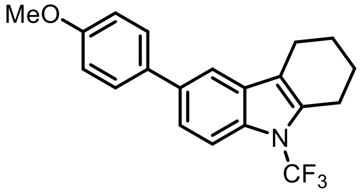

25

6-(4-Methoxyphenyl)-9-(trifluoromethyl)-2,3,4,9-tetrahydro-1*H*-carbazole 25: Under inert atmosphere bromo-9-(trifluoromethyl)-2,3,4,9-tetrahydro-1*H*-carbazole (63 mg, 0.2 mmol, 1 equiv.) and Pd^I iodo-dimer[7] (5 mg, 0.005 mmol, 2.5 mol%) were dissolved in toluene (3 mL) and (4-methoxyphenyl)magnesium bromide solution (600 μL, 0.3 mmol, 1.5 equiv) was added. The reaction was stirred for 10 min and then exposed to air for 5 min. The reaction mixture was then quenched with sat. NH_4Cl solution. The organic phase was collected, dried over Na_2SO_4, adsorbed onto celite. The title compound was obtained as a colorless oil in 90% yield (63 mg) after column chromatography using CH_2Cl_2/pentane (20/80, R_f = 0.34). ^{1}H NMR (600 MHz, $CDCl_3$) δ 7.60 - 7.56 (m, 4H), 7.44 (dd, *J* = 8.7, 1.8 Hz, 1H), 7.00 (d, *J* = 8.7 Hz, 2H), 3.87 (s, 3H), 2.84 (t, *J* = 5.8 Hz, 2H), 2.70 (t, *J* = 6.1 Hz, 2H), 1.98 - 1.92 (m, 2H), 1.90 – 1.84 (m, 2H). ^{19}F NMR (564 MHz, $CDCl_3$) δ -52.4 (s, 3F). ^{13}C NMR (151 MHz, $CDCl_3$) δ 159.0, 135.4, 134.5, 134.3, 133.9, 130.1, 128.5, 122.7, 121.1 (q, *J* = 261.0 Hz), 116.5, 116.4, 114.3, 112.4 (q, *J* = 4.2 Hz), 55.5, 23.4 (q, *J* = 2.6 Hz), 23.2, 22.4, 21.0. IR (neat, cm^{-1}): 2943, 2854, 2032, 1881, 1745, 1604, 1519, 1464, 1366, 1222, 1180, 1109, 1022, 969, 948, 880, 804, 736, 705, 680. HRMS (+c ESI) calculated for $C_{20}H_{19}ONF_3$: 326.14133 $[M+H]^+$, Found: 326.14114.

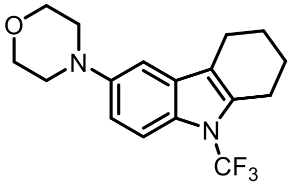

26

4-(9-(Trifluoromethyl)-2,3,4,9-tetrahydro-1*H*-carbazol-6-yl)morpholine 26: A 4 mL vial was charged bromo-9-(trifluoromethyl)-2,3,4,9-tetrahydro-1*H*-carbazole (65 mg, 0.2 mmol, 1 equiv.) along with Cs_2CO_3 (169 mg, 0.52 mmol, 2.6 equiv.), $Pd(OAc)_2$ (5 mg, 0.02 mmol, 10 mol%), BINAP (19 mg, 0.03 mmol, 5 mol%). The vial was sealed and purged with argon. Anhydrous toluene (2 mL) was added followed by anhydrous morpholine (21 μL, 0.24 mmol, 1.2 equiv.). The reaction was then stirred at 110°C for 16h. The reaction mixture was allowed to cool to room temperature and the crude was then purified by column chromatography by directly loading it onto silica and eluting using 100% CH_2Cl_2. The title compound was obtained as a brown oil in 70% yield (45 mg) after column chromatography using 100% CH_2Cl_2 (R_f = 0.20). ^{1}H NMR (600 MHz, $CDCl_3$) δ 7.46 (dd, *J* = 9.7, 1.9 Hz, 1H), 6.96 – 6.92 (m, 2H), 3.94 – 3.89 (m, 4H), 3.20 – 3.14 (m, 4H), 2.80 (t, *J* = 6.4 Hz, 2H), 2.63 (t, *J* = 6.2 Hz, 2H), 1.95 – 1.90 (m, 2H), 1.87 – 1.82 (m, 2H). ^{19}F NMR (564 MHz, $CDCl_3$) δ -52.6 (s, 3F). ^{13}C NMR (151 MHz, $CDCl_3$) δ 147.6, 134.4, 130.3, 129.8, 121.2 (q, *J* = 260.6 Hz), 116.3, 114.6, 112.8 (q, *J* = 4.1

Hz), 105.2, 67.2, 51.3, 23.3 (q, J = 2.5 Hz), 23.1, 22.3, 21.0. IR (neat, cm^{-1}): 2935, 2853, 1729, 1687, 1614, 1475, 1367, 1327, 1258, 1218, 1196, 1110, 1046, 972, 951, 844, 798, 732, 683. HRMS (+c ESI) calculated for $C_{17}H_{20}N_2OF_3$: 325.15222 $[M+H]^+$, Found: 325.15222.

Procedures for the derivatization of the free NH_2 moiety of *N*-CF_3 hydrazines

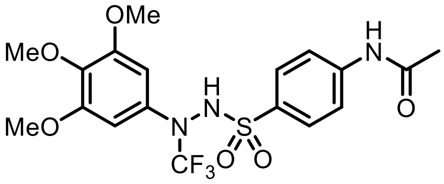

29

***N*-(4-((2-(trifluoromethyl)-2-(3,4,5-trimethoxyphenyl)hydrazineyl)sulfonyl)phenyl)acetamide 29:** DMAP (5.5 mg, 0.045 mmol, 0.22 equiv.) and pyridine (24 µL, 0.3 mmol, 1.5 equiv.) were added to a solution of 1-(trifluoromethyl)-1-(3,4,5-trimethoxyphenyl)hydrazine (53 mg, 0.2 mmol, 1 equiv.) in DCM (200 µL) at 0°C. 4-acetamidobenzenesulfonyl chloride (70.1 mg, 0.3 mmol, 1.5 equiv.) was added at 0°C and the mixture was stirred for 2.5 h at 0°C. Then aqueous sat. sodium bicarbonate solution was added to quench the reaction. Organic materials were extracted with EtOAc (3x). The combined extracts were washed with brine and dried over anhydrous sodium sulfate. After removal of the solvent under reduced pressure, the residue was purified. The title compound was obtained as a colorless solid in 68% yield (63 mg) after column chromatography using pentane/EtOAc/NEt_3, 10/20/1 to 10/40/1 (10/20/1, R_f = 0.06). M.p.: 252 -253 °C (decomp.). ^{1}H NMR (600 MHz, Acetone-d_6) δ 9.50 (s, 1H), 9.11 (s, 1H), 7.66 (d, *J* = 8.9 Hz, 2H), 7.63 (d, *J* = 8.9 Hz, 2H), 6.55 (s, 2H), 3.72 (s, 6H), 3.63 (s, 3H), 2.09 (s, 3H). ^{19}F NMR (564 MHz, Acetone-d_6) δ -66.0 (s, 3F). ^{13}C NMR (151 MHz, Acetone-d_6) δ 169.5, 154.0, 144.7, 139.2, 135.8, 133.2, 130.3, 123.4 (q, *J* = 258.5 Hz), 118.9, 105.8, 60.5, 56.5, 24.4. IR (neat, cm^{-1}): 3330, 3153, 2938, 2890, 2165, 1678, 1591, 1530, 1502, 1459, 1412, 1335, 1238, 1192, 1159, 1122, 1087, 1043, 1000, 911, 848, 826, 790, 726, 705. HRMS (+c ESI) calculated for $C_{18}H_{20}O_6N_3F_3NaS$: 486.09048 $[M+Na]^+$, Found: 486.09048. Crystals suitable for X-ray diffraction analysis were grown by slow evaporation from a solution of chloroform and acetone. CCDC 1989761 contains the supplementary crystallographic data for this compound.

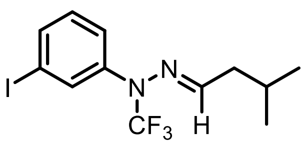

30

(*E*)-1-(3-iodophenyl)-2-(3-methylbutylidene)-1-(trifluoromethyl)hydrazine 30: To a solution of 1-(3-iodophenyl)-1-(trifluoromethyl)hydrazine (60 mg, 0.2 mmol, 1 equiv.) in acetic acid (1 mL) was added isovaleraldehyde (43 µL, 0.4 mmol, 2 equiv.). The obtained mixture was then heated at 80°C for 4 h. After allowing the reaction mixture to cool to room temperature, it was diluted with EtOAc and washed with H_2O (4x). The organic phase was concentrated *in vacuo* and directly purified. The title compound was obtained as an orange oil in quantitative yield (74 mg) after column chromatography using pentane/CH_2Cl_2 85/15 (70/30, R_f = 0.58). ^{1}H NMR (600 MHz, $CDCl_3$) δ 7.76 (ddd, *J* = 7.8, 1.4, 1.4 Hz, 1H), 7.63 (dd, *J* = 1.9, 1.9 Hz, 1H), 7.27 – 7.24 (m, 1H), 7.20 (dd, *J* = 7.9, 7.9 Hz, 1H), 6.74 (dd, *J* = 5.9, 5.9 Hz, 1H), 2.16 (dd, *J* = 7.0, 5.9 Hz, 2H), 1.78 (dsept, *J* = 13.6, 6.8 Hz, 1H), 0.89 (d, *J* = 6.7 Hz, 6H). ^{19}F NMR (564 MHz, $CDCl_3$) δ -62.6 (s, 3F). ^{13}C NMR (151 MHz, $CDCl_3$) δ

150.9, 139.4, 138.8, 138.7, 131.1, 129.1, 122.2 (q, J = 257.8 Hz), 94.6, 41.3, 26.9, 22.5. IR (neat, cm^{-1}): 2957, 2873, 2329, 2083, 1882, 1568, 1467, 1412, 1377, 1301, 1234, 1196, 1123, 1065, 999, 957, 880, 786, 715, 684. HRMS (+c ESI) calculated for $C_{12}H_{15}N_2F_3I$: 371.02265 $[M+H]^+$, Found: 371.02240.

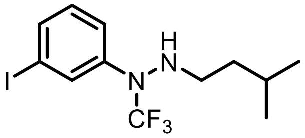

31

1-(3-iodophenyl)-2-isopentyl-1-(trifluoromethyl)hydrazine 31: (*E*)-1-(3-iodophenyl)-2-(3-methylbutylidene)-1-(trifluoromethyl)hydrazine (74 mg, 0.2 mmol, 1 equiv.) was dissolved in toluene and BH_3•NMe_3 (15 mg, 0.2 mmol, 1 equiv.) was added. HCl gas (generated from a NaCl/H_2SO_4 mixture) was then bubbled through the solution for 30 minutes. The crude solution was diluted with DCM and washed with 0.5M NaOH and water. The organic phase was dried over Na_2SO_4, concentrated *in vacuo*, redissolved in 0.5 mL CH_2Cl_2/pentane (6/4), filtered through silica and washed with 5 mL of the eluent. It was then concentrated *in vacuo* to obtain the title compound as a colorless oil in 97% yield without further purification. ^{1}H NMR (600 MHz, $CDCl_3$) δ 7.68 (s, 1H), 7.61 (d, J = 8.0 Hz, 1H), 7.28 (d, J = 7.1 Hz, 1H), 7.11 (t, J = 8.0 Hz, 1H), 3.69 (t, J = 5.3 Hz, 1H), 2.79 (td, J = 7.4, 5.3 Hz, 2H), 1.63 (dsept, J = 13.5, 6.8 Hz, 1H), 1.36 (q, J = 7.1 Hz, 2H), 0.87 (d, J = 6.6 Hz, 6H). ^{19}F NMR (564 MHz, $CDCl_3$) δ -63.2 (s, 3F). ^{13}C NMR (151 MHz, $CDCl_3$) δ 142.7, 136.0, 133.6, 130.6, 123.9, 123.1 (q, J = 258.1 Hz), 94.2, 47.7, 36.5, 25.7, 22.6. IR (neat, cm^{-1}): 2955, 2870, 2160, 1788, 1583, 1468, 1413, 1376, 1303, 1239, 1197, 1128, 1089, 990, 944, 881, 843, 782, 782, 746, 701, 680. HRMS (+c ESI) calculated for $C_{12}H_{17}N_2F_3I$: 373.03830 $[M+H]^+$, Found: 373.03836.

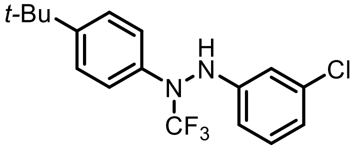

32

1-(4-(*tert*-Butyl)phenyl)-2-(3-chlorophenyl)-1-(trifluoromethyl)hydrazine 32: In an argon-filled glove box a vial was loaded with 1-(4-(*tert*-butyl)phenyl)-1-(trifluoromethyl)hydrazine (46 mg, 0.2 mmol, 1 equiv.), $Pd(OAc)_2$, (9 mg, 0.04 mmol, 20 mol%), tri-*tert*-butylphosphine (8 mg, 0.04 mmol, 20 mol%), Cs_2CO_3 (98 mg, 0.3 mmol, 1.5 equiv.), 1-bromo-3-chlorobenzene (35 µL, 0.3 mmol, 1.5 equiv.) and anhydrous toluene (2 mL). The reaction mixture was then stirred at 110 °C for 16 h. The reaction mixture was allowed to cool to room temperature and then directly loaded onto the column. The title compound was obtained as a dark orange oil in 61% yield (42 mg) after column chromatography using 10% EtOAc/pentane (20% CH_2Cl_2/pentane R_f = 0.37). ^{1}H NMR (600 MHz, $CDCl_3$) δ 7.38 (dd, J = 8.7, 2.2 Hz, 2H), 7.27 (d, J = 8.5 Hz, 2H), 7.17 (dd, J = 8.0, 8.0 Hz, 1H), 7.02 (d, J = 2.1 Hz, 1H), 6.88 (d, J = 7.9 Hz, 1H), 6.84 (d, J = 8.1 Hz, 1H), 5.77 (s, 1H), 1.31 (s, 9H). ^{19}F NMR (564 MHz, $CDCl_3$) δ -64.7 (s, 3F). ^{13}C NMR (151 MHz, $CDCl_3$) δ 150.6, 148.1, 139.1, 135.3, 130.4, 126.6, 123.3 (q, J = 259.4 Hz), 122.8, 121.2, 113.5, 111.6, 34.7, 31.4. IR (neat, cm^{-1}): 3347, 2963, 2871, 2327, 2089, 1921, 1596, 1507, 1477, 1419, 1365, 1297, 1241, 1143, 1094, 1019, 993, 936, 888, 839, 770, 732, 682. HRMS (+c ESI) calculated for $C_{17}H_{19}N_2{}^{35}ClF_3$: 343.11834 $[M+H]^+$, Found: 343.11816.

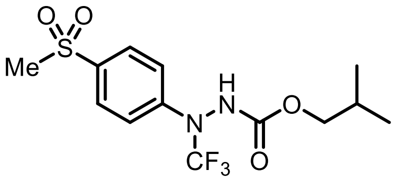

33

***iso*-Butyl 2-(4-(methylsulfonyl)phenyl)-2-(trifluoromethyl)hydrazine-1-carboxylate 33:** To a solution of 1-(4-(methylsulfonyl)phenyl)-1-(trifluoromethyl)hydrazine (51 mg, 0.2 mmol, 1 equiv.), pyridine (24 µL, 0.3 mmol, 1.5 equiv.) and DMAP (12 mg, 0.02 mmol, 0.1 equiv.) in $CHCl_3$ (1 mL) was slowly added isobutyl chloroformate (27 µL, 0.24 mmol, 1.2 equiv.). The reaction mixture was stirred for 1 h, concentrated *in vacuo* and directly purified. The title compound was obtained as a colorless solid in 88% yield (62 mg) after column chromatography using 20% EtOAc/pentane (50% EtOAc/pentane, R_f = 0.42). M.p.: 105 - 106 °C. ^{1}H NMR (600 MHz, $CDCl_3$) δ 7.85 (br, 2H), 7.41 (br, 1H), 7.32 (d, *J* = 8.5 Hz, 2H), 3.95 (br, 2H), 3.02 (s, 3H), 1.94 (br, 1H), 0.98-0.74 (m, 6H). ^{19}F NMR (564 MHz, $CDCl_3$) δ -60.9 (s, 3F). ^{13}C NMR (151 MHz, $CDCl_3$) δ 155.4, 145.5, 136.2, 128.9, 121.6 (q, *J* = 262.0 Hz), 118.8, 118.3, 72.8, 44.7, 28.0, 18.9. IR (neat, cm^{-1}): 3324, 2970, 1729, 1596, 1494, 1299, 1246, 1181, 1152, 1117, 1035, 944, 835, 795, 765, 700. HRMS (+c ESI) calculated for $C_{13}H_{17}O_4N_2F_3NaS$: 377.07533 $[M+Na]^+$, Found: 377.07504.

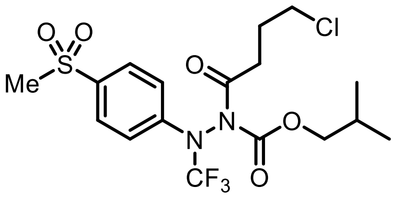

34

***iso*-Butyl 1-(4-chlorobutanoyl)-2-(4-(methylsulfonyl)phenyl)-2-(trifluoromethyl)hydrazine-1-carboxylate 34:** To a solution of *iso*-butyl 2-(4-(methylsulfonyl)phenyl)-2-(trifluoromethyl)hydrazine-1-carboxylate (71 mg, 0.2 mmol, 1 equiv.), pyridine (52 µL, 0.64 mmol, 2 equiv.) and DMAP (12 mg, 0.02 mmol, 0.1 equiv.) in $CHCl_3$ (1 mL) was slowly added 4-chlorobutanoyl chloride (68 µL, 0.4 mmol, 2 equiv.). The reaction mixture was stirred for 24 h, concentrated *in vacuo* and directly purified. The title compound was obtained as a colorless oil in 98% yield (90 mg) after column chromatography using pentane/EtOAc, 4/1 to 2/1 (4/1, R_f = 0.17). ^{1}H NMR (600 MHz, $CDCl_3$) δ 7.91 (d, *J* = 9 Hz, 2H), 7.11 (d, *J* = 8.1 Hz, 2H), 4.06 (dd, *J* = 10.4, 6.3 Hz, 1H), 3.98 (dd, *J* = 10.4, 6.3 Hz, 1H), 3.63 (t, *J* = 6.2 Hz, 2H), 3.30 – 3.16 (m, 2H), 3.02 (s, 3H), 2.21 – 2.13 (m, 2H), 1.88 (sept, *J* = 6.6 Hz, 1H), 0.81 (dd, *J* = 6.8, 4.3 Hz, 6H). ^{19}F NMR (564 MHz, $CDCl_3$) δ -58.8 (s, 3F). ^{13}C NMR (151 MHz, $CDCl_3$) δ 172.0, 152.8, 143.5, 135.5, 129.4, 123.1, 120.5 (q, *J* = 262.8 Hz), 115.4 (d, *J* = 2.6 Hz), 74.9, 44.8, 43.9, 34.3, 27.7, 27.1, 18.7 (d, *J* = 3.7 Hz). IR (neat, cm^{-1}): 2966, 1757, 1596, 1501, 1469, 1403, 1377, 1302, 1245, 1189, 1144, 1097, 1048, 950, 830, 766, 734, 704. HRMS (EI) calculated for $C_{17}H_{22}N_2O_5{}^{35}ClF_3{}^{32}SNa$: 481.07823 $[M+Na]^+$, Found: 481.07840.

General procedure for the synthesis of *N*-trifluoromethylcarbamic fluorides

[1,1'-biphenyl]-4-yl(trifluoromethyl)carbamic fluoride **1**, (3-iodophenyl)(trifluoromethyl)carbamic fluoride, (4-bromo-2-chlorophenyl)(trifluoromethyl)carbamic fluoride, (3-(methylthio)phenyl)(trifluoromethyl)carbamic fluoride and (4-(methylsulfonyl)phenyl)(trifluoromethyl)carbamic fluoride were prepared using previously reported procedure and analyses matched previously reported data. The same procedure was used to prepare all new carbamic fluorides without any changes and all compounds were fully characterized.[1]

A 20 mL vial was charged with the isothiocyanate (2 mmol, 1 equiv.), silver fluoride (10 mmol, 5 equiv.) and bis(trichloromethyl) carbonate (BTC) (237 mg, 0.8 mmol, 0.4 equiv.). Acetonitrile (10 mL) was added quickly and the vial was sealed (if the isothiocyanate was a liquid or an oil it was added as a solution in acetonitrile). The mixture was stirred at room temperature. After the indicated time, the crude mixture was added at once to Et_2O (40 mL) and stirred for 10 minutes. The obtained mixture was filtered through celite and the solvents were then evaporated. The crude material was redissolved in Et_2O and was filtered once more through a pad of celite to remove the last traces of salt byproducts. The *N*-trifluoromethylcarbamic fluoride was then obtained in a technical grade purity ranging from 90 to 99%.

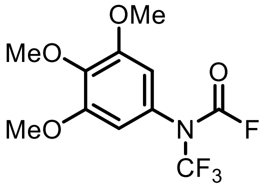

(Trifluoromethyl)(3,4,5-trimethoxyphenyl)carbamic fluoride : The title compound was obtained as an off-white solid after 18 h in 99% yield (588 mg) from 5-isothiocyanato-1,2,3-trimethoxybenzene (450 mg) following the general procedure for *N*-trifluoromethylcarbamic fluorides. M.p.: 118-119 °C. ^{1}H NMR (600 MHz, $CDCl_3$) δ 6.52 (s, 2H), 3.88 (s, 3H), 3.87 (s, 6H). ^{19}F NMR (564 MHz, $CDCl_3$) δ -2.6 (brs, 1F), -56.5 (brs, 3F). ^{13}C NMR (151 MHz, $CDCl_3$) δ 154.0, 142.3 (d, J = 300.1 Hz), 139.8, 128.4, 119.4 (q, J = 264.6 Hz), 105.9, 61.1, 56.5. IR (neat, cm^{-1}): 2950, 2842, 1830, 1600, 1506, 1452, 1418, 1389, 1316, 1234, 1158, 1127, 1053, 1013, 974, 837, 783, 751, 720, 697. HRMS (EI) calculated for $C_{11}H_{11}O_4NF_4$: 297.06187 $[M]^+$, Found: 297.06196.

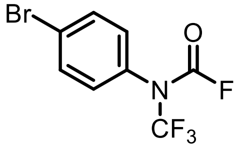

(4-Bromophenyl)(trifluoromethyl)carbamic fluoride : The title compound was obtained as a yellow liquid after 8 h in 94% yield (535 mg) from 1-bromo-4-isothiocyanatobenzene (428 mg) following the general procedure for *N*-trifluoromethylcarbamic fluorides. ^{1}H NMR (600 MHz, $CDCl_3$) δ 7.63 (dd, J = 8.6, 1.6 Hz, 2H), 7.21 (d, J = 8.6 Hz, 2H). ^{19}F NMR (376 MHz, $CDCl_3$) δ -2.1 (brs, 1F), -56.1 (brs, 3F). ^{13}C NMR (151 MHz, $CDCl_3$) δ 141.8 (d, J = 301.4 Hz), 133.4, 132.2, 130.1, 125.0, 119.1 (q, J = 265.1 Hz). IR (neat, cm^{-1}): 1835, 1489, 1367, 1326, 1269, 1169, 1106, 1070, 1020, 1001, 961, 947, 830, 755, 723, 677.

MS (70eV, EI): m/z (%): 287 (99) [^{81}Br-M], 285 (100) [^{79}Br-M], 174 (48), 176 (46), 159 (66), 137 (50), 96 (26), 69 (33).

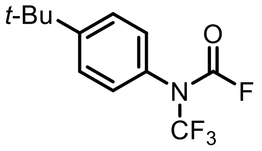

(4-(*tert*-Butyl)phenyl)(trifluoromethyl)carbamic fluoride : The title compound was obtained as a yellow liquid after 8 h in 99% yield (520 mg) from 1-(*tert*-butyl)-4-isothiocyanatobenzene (383 mg) following the general procedure for *N*-trifluoromethylcarbamic fluorides. ^{1}H NMR (600 MHz, $CDCl_3$) δ 7.46 (d, *J* = 8.6 Hz, 2H), 7.22 (d, *J* = 8.6 Hz, 2H), 1.31 (s, 9H). ^{19}F NMR (376 MHz, $CDCl_3$) δ -1.9 (brs, 1F), -56.3 (brs, 3F). ^{13}C NMR (151 MHz, $CDCl_3$) δ 153.8, 142.3 (d, *J* = 299.3 Hz), 130.4, 127.8, 126.9, 119.4 (q, *J* = 264.5 Hz), 34.9, 31.2. IR (neat, cm^{-1}): 2966, 2875, 1835, 1510, 1468, 1362, 1326, 1271, 1167, 1111, 1021, 1000, 963, 838, 754, 718, 684. HRMS (EI) calculated for $C_{12}H_{13}ONF_4$: 263.09278 $[M]^+$, Found: 263.09282. IR (neat, cm^{-1}): 2966, 2877, 1825, 1509, 1329, 1275, 1170, 1109, 1001, 960, 839, 754, 716, 682.

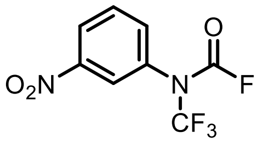

(3-Nitrophenyl)(trifluoromethyl)carbamic fluoride : The title compound was obtained as a orange liquid after 2 h in 90% yield (227 mg) from 1-isothiocyanato-3-nitrobenzene (180 mg) following the general procedure for *N*-trifluoromethylcarbamic fluorides. ^{1}H NMR (600 MHz, $CDCl_3$) δ 8.41 (d, *J* = 8.1 Hz, 1H), 8.25 (s, 1H), 7.79 -7.68 (m, 2H). ^{19}F NMR (376 MHz, $CDCl_3$) δ -3.2 (brs, 1F), -55.6 (brs, 3F). ^{13}C NMR (151 MHz, $CDCl_3$) δ 149.0, 141.5 (d, *J* = 303.3 Hz), 134.6, 134.0, 131.2, 125.6, 124.1, 119.0 (q, *J* = 266.6 Hz). IR (neat, cm^{-1}): 3109, 2162, 1828, 1533, 1481, 1374, 1343, 1257, 1169, 1090, 1024, 973, 914, 862, 816, 778, 749, 693. MS (70eV, EI): m/z (%): 252 (100) [M], 206 (33), 159 (38), 137 (59), 69 (79).

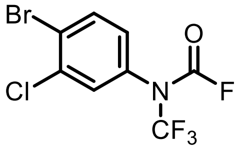

(4-Bromo-3-chlorophenyl)(trifluoromethyl)carbamic fluoride : The title compound was obtained as a yellow liquid in 96% yield (614 mg) from 1-bromo-2-chloro-4-isothiocyanatobenzene (497 mg) following the general procedure for *N*-trifluoromethylcarbamic fluorides. ^{1}H NMR (600 MHz, $CDCl_3$) δ 7.76 (d, *J* = 8.6 Hz, 1H), 7.47 (d, *J* = 2.5 Hz, 1H), 7.13 (dd, *J* = 8.6, 2.5 Hz, 1H). ^{19}F NMR (376 MHz, $CDCl_3$) δ -1.3 (brs, 1F), -56.2 (brs, 3F). ^{13}C NMR (151 MHz, $CDCl_3$) δ 141.5 (d, *J* = 301.9 Hz), 136.2, 135.1, 132.8, 130.5, 127.9, 125.6, 119.0 (q, *J* = 265.8 Hz). IR (neat, cm^{-1}): 1837, 1578, 1463, 1367, 1270, 1175, 1113, 1024, 975, 882, 815, 744. MS (70eV, EI): m/z (%): 321 (100) [^{35}Cl,^{81}Br-M], 319 [^{35}Cl,^{79}Br-M] (76), 210 (28), 208 (21), 171 (17), 69 (10).

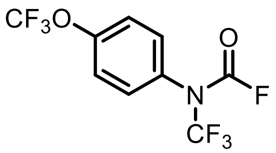

(4-(Trifluoromethoxy)phenyl)(trifluoromethyl)carbamic fluoride : The title compound was obtained as an orange liquid after 5 h in 67% yield (390 mg) from 1-isothiocyanato-4-(trifluoromethoxy)benzene (438 mg) following the general procedure for *N*-trifluoromethylcarbamic fluorides. ^{1}H NMR (600 MHz, $CDCl_3$)

δ 7.39 (d, J = 9.0 Hz, 2H), 7.34 (d, J = 8.6 Hz, 2H). ^{19}F NMR (376 MHz, $CDCl_3$) δ -1.3 (brs, 1F), -56.3 (brs, 3F), -58.0 (brs, 3F). ^{13}C NMR (151 MHz, $CDCl_3$) δ 150.5 (d, J = 1.8 Hz), 141.9 (d, J = 301.0 Hz), 131.4, 130.3, 122.3, 120.4 (q, J = 258.9 Hz), 119.2 (q, J = 265.2 Hz). IR (neat, cm^{-1}): 1839, 1510, 1372, 1336, 1256, 1166, 1010, 958, 850, 756, 689. MS (70eV, EI): m/z (%): 291 (100) [M], 272 (5), 244 (14), 180 (40), 158 (24), 111 (22), 69 (44).

General procedure for the synthesis of isothiocyanates

A 100 mL round-bottom flask was charged with the aniline (5 mmol, 1 equiv.), CH_2Cl_2 (25 mL) and saturated aqueous $NaHCO_3$ (25 mL). Under strong stirring thiophosgene (460 μL, 6 mmol, 1.2 equiv.) was slowly added to the biphasic system at room temperature. After 1 h, the two phases were separated and the aqueous phase was extracted with CH_2Cl_2 (2x). The combined organic phases were dried over $MgSO_4$ and concentrated under reduced pressure. The product was then used without further purification.

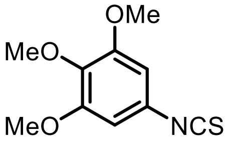

5-Isothiocyanato-1,2,3-trimethoxybenzene : The title compound was obtained as a brown solid in 99% yield (2.25 g) from 3,4,5-trimethoxyaniline (1.83 g) following the general procedure for isothiocyanates. ^{1}H NMR (400 MHz, $CDCl_3$) δ 6.45 (s, 2H), 3.84 (s, 6H), 3.82 (s, 3H). ^{13}C NMR (101 MHz, $CDCl_3$) δ 153.7, 137.8, 134.9, 126.7, 103.4, 61.2, 56.4. IR (neat, cm^{-1}): 3097, 2973, 2942, 2837, 2117, 1739, 1581, 1498, 1459, 1416, 1338, 1228, 1120, 991, 870, 819, 759, 726, 671. HRMS (+c ESI) calculated for $C_{10}H_{11}O_3NNaS$: 248.03519 $[M+Na]^+$, Found: 248.03494.

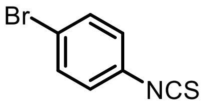

1-Bromo-4-isothiocyanatobenzene : The title compound was obtained as an off-white solid in 94% yield (1.06 g) from 4-bromoaniline (1.72 g) following the general procedure for isothiocyanates. M.p.: 56 - 57 °C. ^{1}H NMR (400 MHz, $CDCl_3$) δ 7.47 (d, *J* = 8.7 Hz, 2H), 7.09 (d, *J* = 8.7 Hz, 2H). ^{13}C NMR (101 MHz, $CDCl_3$) δ 137.0, 132.9, 130.6, 127.3, 120.9. IR (neat, cm^{-1}): 3082, 2602, 2482, 2172, 2072, 1889, 1758, 1634, 1574, 1472, 1399, 1272, 1242, 1169, 1099, 1061, 1010, 918, 811, 715. HRMS (EI) calculated for $C_7H_4N^{32}S^{79}Br$: 212.92423 $[M]^+$, Found: 212.92467.

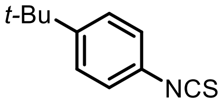

1-(*tert*-Butyl)-4-isothiocyanatobenzene : The title compound was obtained as an orange solid in 92% yield (898 mg) from 4-(*tert*-butyl)aniline (745 mg) following the general procedure for isothiocyanates. M.p.: 26-27 °C. ^{1}H NMR (400 MHz, $CDCl_3$) δ 7.36 (d, *J* = 8.6 Hz, 2H), 7.16 (d, *J* = 8.6 Hz, 2H), 1.31 (s, 9H). ^{13}C NMR (101 MHz, $CDCl_3$) δ 150.9, 134.4, 128.4, 126.6, 125.5, 34.9, 31.3. IR (neat, cm^{-1}): 2959, 2902, 2868, 2182, 2086, 1901, 1655, 1593, 1500, 1463, 1412, 1365, 1263, 1202, 1106, 1019, 925, 831, 724. HRMS (+c ESI) calculated for $C_{11}H_{14}NS$: 192.08415 $[M+H]^+$, Found: 192.08400.

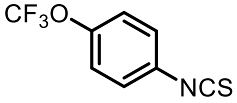

1-Isothiocyanato-4-(trifluoromethoxy)benzene : The title compound was obtained as a black liquid in 99% yield (2.17 g) from 4-(trifluoromethoxy)aniline (1.77 g) following the general procedure for isothiocyanates. ^{1}H NMR (400 MHz, $CDCl_3$) δ 7.25 – 7.19 (m, 4H). ^{19}F NMR (376 MHz, $CDCl_3$) δ -58.2 (s, 3F). ^{13}C NMR (101 MHz, $CDCl_3$) δ 147.7 (q, *J* = 1.9 Hz), 137.3, 130.3, 127.2, 122.3, 120.4 (q, *J* = 258.1 Hz). IR (neat, cm^{-1}): 2193, 2078, 1501,

1251, 1160, 1103, 1016, 931, 843, 807, 726, 665. HRMS (EI) calculated for $C_8H_4NOF_3{}^{32}S$: 218.99602 $[M]^+$, Found: 218.99593.

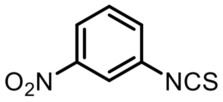

1-Isothiocyanato-3-nitrobenzene : The title compound was obtained as an orange solid in 96% yield (864 mg) from 3-nitroaniline (691 mg) following the general procedure for isothiocyanates. M.p.: 57 - 58°C. ^1H NMR (600 MHz, $CDCl_3$) δ 8.13 (ddd, *J* = 7.6, 2.0, 2.0 Hz, 1H), 8.07 (s, 1H), 7.60 – 7.51 (m, 2H). ^{13}C NMR (151 MHz, $CDCl_3$) δ 148.9, 139.8, 133.4, 131.6, 130.6, 122.0, 120.9. IR (neat, cm^{-1}): 3097, 3094, 2226, 2112, 1567, 1520, 1343, 1163, 1090, 973, 890, 849, 808, 726, 664. HRMS (APCI+) calculated for $C_7H_5N_2O_2S$: 181.00662 $[M+H]^+$, Found: 181.00645.

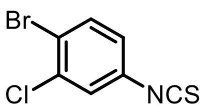

1-Bromo-2-chloro-4-isothiocyanatobenzene : The title compound was obtained as a brown liquid in 92% yield (1.14 g) using 4-bromo-3-chloroaniline (1.03 g) following the general procedure for isothiocyanates. ^1H NMR (600 MHz, $CDCl_3$) δ 7.56 (d, *J* = 8.6 Hz, 1H), 7.29 (d, *J* = 2.4 Hz, 1H), 6.96 (dd, *J* = 8.6, 2.4 Hz, 1H). ^{13}C NMR (151 MHz, $CDCl_3$) δ 138.8, 135.6, 134.5, 131.8, 127.3, 125.1, 121.2. IR (neat, cm^{-1}): 3083, 2806, 2639, 2346, 2236, 2195, 2085, 1730, 1576, 1552, 1457, 1377, 1273, 1232, 1108, 1018, 961, 864, 807, 759, 678. MS (70eV, EI): m/z (%): 249 (100) [$^{35}Cl,^{81}Br$-M], 247 (76) [$^{35}Cl,^{79}Br$-M], 133 (29), 74 (23).

3. Crystallographic details

Compound	5	15	28	29
CCDC number	1989758	1989759	1989760	1989761
Formula	$C_9H_7F_3N_4O_3S$	$C_8H_9F_3N_2O_2S$	$C_{22}H_{16}F_3N$	$C_{18}H_{20}F_3N_3O_6S$
Formula Weight	308.25	254.23	351.36	463.43
Colour	colourless	colourless	colourless	colourless
Shape	prism	needle	plate	prism
Size/mm^3	0.97×0.70×0.37	0.43×0.34×0.13	0.74×0.71×0.17	0.42×0.42×0.18
Temperature/K	296(2)	296(2)	296(2)	296(2)
Crystal System	monoclinic	monoclinic	monoclinic	monoclinic
Space Group	$P2_1/n$	$P2_1/c$	$P2_1/c$	Cc
a/Å	8.2033(9)	9.6419(14)	12.843(9)	15.5035(18)
b/Å	13.5470(15)	5.3527(8)	16.212(11)	8.6710(8)
c/Å	11.2952(17)	20.548(3)	8.636(6)	17.2119(17)
α/°	90	90	90	90
β/°	94.902(2)	92.367(4)	102.485(15)	114.138(4)
γ/°	90	90	90	90
V/Å^3	1250.6(3)	1059.6(3)	1756(2)	2111.5(4)
Z	4	4	4	4
Z'	1	1	1	1
ρ_{calc}/g cm^{-3}	1.637	1.594	1.329	1.458
μ/mm^{-1}	0.310	0.335	0.100	0.220
Radiation type	MoK$_\alpha$	MoK$_\alpha$	MoK$_\alpha$	MoK$_\alpha$
Wavelength/Å	0.71073	0.71073	0.71073	0.71073
Θ_{min}/°	2.353	1.984	1.624	2.593
Θ_{max}/°	38.817	35.628	28.104	30.930
Measured Refl.	105787	70249	62772	50001
Independent Refl.	104065	65393	61534	48148
Reflections with I > 2(I)	5157	3560	2751	5600
R_{int}	0.0229	0.0236	0.0392	0.0247
Parameters	210	147	264	288
Restraints	93	0	3	41
Largest Peak/eÅ^{-3}	0.370	0.356	0.222	0.187
Largest Hole/eÅ^{-3}	-0.473	-0.236	-0.235	-0.218
GooF on F^2	1.065	1.053	1.029	1.043
wR_2 (all data)	0.1485	0.1317	0.1361	0.0955
wR_2	0.1184	0.1115	0.1081	0.0866
R_1 (all data)	0.0650	0.0598	0.0840	0.0452
R_1	0.0433	0.0400	0.0470	0.0334

Suitable crystals for X-ray diffraction analysis were selected and mounted on a glass fiber with grease on a Bruker APEX-II CCD diffractometer. The crystals were kept at T = 296 K during data collection. The structures were solved with the ShelXT[8] structure solution program using the direct solution method and by using Olex2[9] as the graphical interface. The model was refined with ShelXL[10] using least squares minimization.

4. Electrophilic *N*-CF_3 indole synthesis

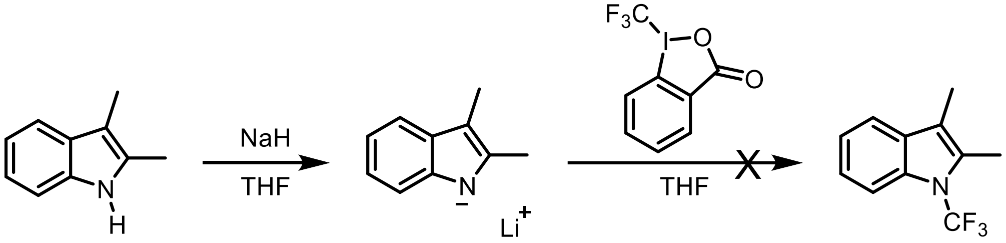

In an argon-filled glovebox, a solution of 2,3-dimethylindole (29 mg, 0.2 mmol, 1 equiv.) in anhydrous THF (0.5 mL) was slowly added to a suspension of dry NaH (5.8 mg, 0.24 mmol, 1.2 equiv.) in anhydrous THF (0.5 mL) . The mixture was stirred for 1 h before filtration under argon using a syringe filter. The obtained filtrate was added dropwise to a suspension of Togni's reagent (158 mg, 0.2 mmol, 1 equiv., 60 wt. %) in dry THF. The mixture was then stirred for 1.5 h before being analyzed by quantitative ^{19}F NMR by diluting an aliquot of the reaction in $CDCl_3$. Only traces of a compound were visible in the expected region for *N*-CF_3 indoles. (-50.1 to -52.6 ppm)

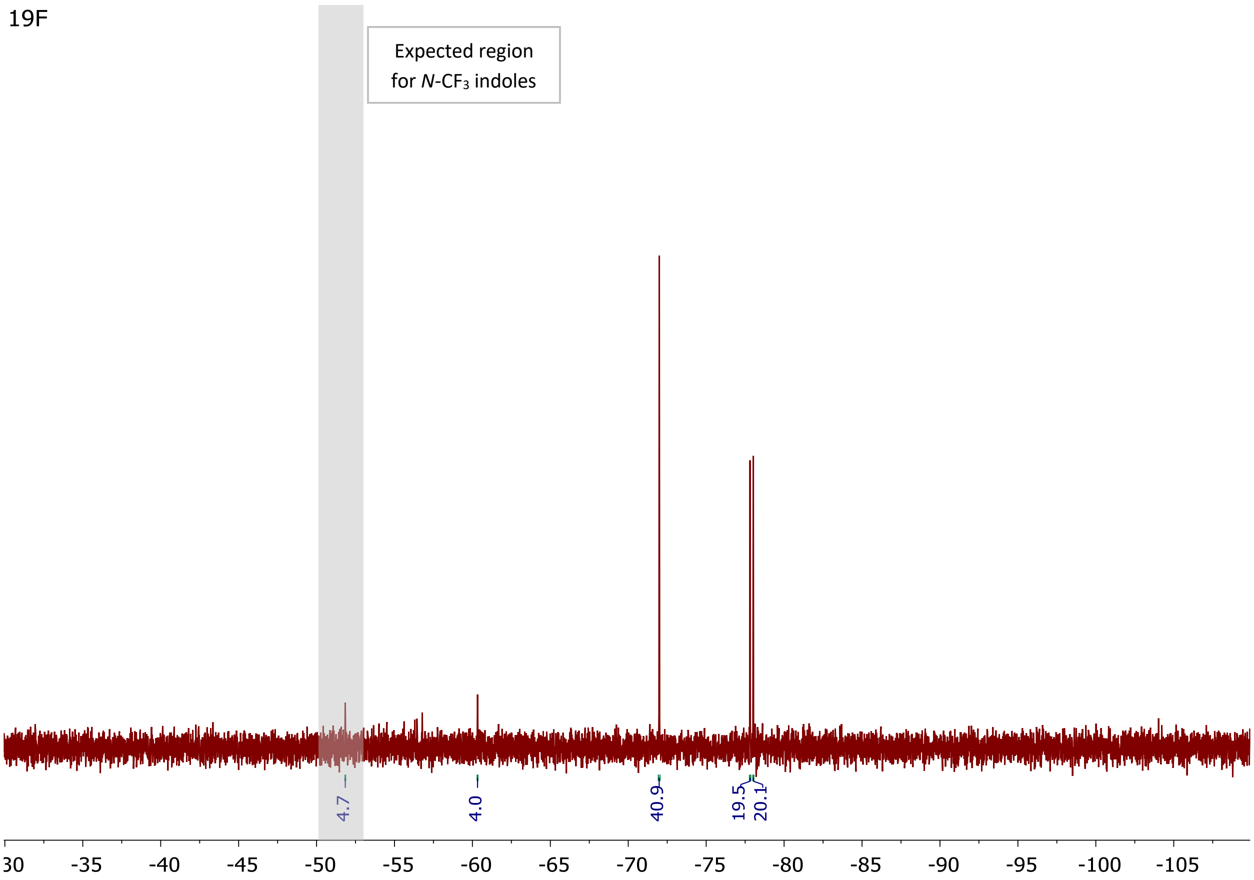

5. General Computational details

Available X-ray structures were imported from the CCDC and reoptimized using DFT. Calculations were performed using Gaussian 09, Revision D.01.[11] Geometry optimization was conducted in the gas-phase at the ωB97XD/6-31G(d) level of theory. Frequencies were calculated at the same level of theory and used to verify the nature of all stationary points as minima (no imaginary frequencies).

Images were created using the CYLview software.[12]

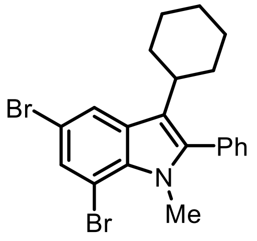

GAZXIR

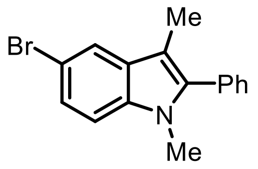

ICOPAU

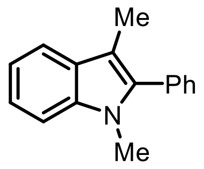

INUXIA

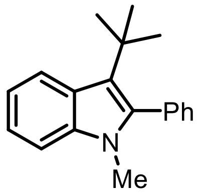

SEFTOM

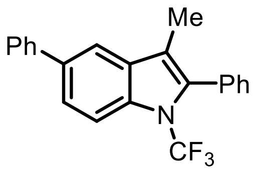

28

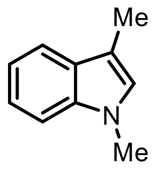

3-Methylindole

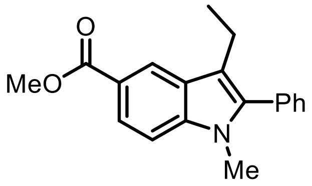

HOMBAP

Table S1 and chart S1 display the correlation between bond length and dihedral angle in various *N*-CH_3 indoles and their *N*-CF_3 counterpart. Two distinct groups arise as replacing the CH_3 moiety with a CF_3 consistently shorten the C-N bond and pyramidalize the nitrogen within the indole.

Table S1. Reported data and calculated values for various *N*-CH_3 and corresponding *N*-CF_3 indoles

**CCDC ID** or **name**	**X-Ray Length (Å)**	**DFT N-CH_3**	**DFT N-CF_3**	**dihedral-N-CH_3**	**dihedral-N-CF_3**	**diff Length (pm)**	**diff Angle (°)**
GAZXIR	1.459	1.454	1.432	169.1	152.6	2.247	16.545
ICOPAU	1.466	1.446	1.418	173.1	154.4	2.726	18.738
INUXIA	1.461	1.445	1.417	172.3	154.2	2.822	18.189
SEFTOM	1.444	1.444	1.416	180.0	166.1	2.841	13.923
**28** (*N*-CF_3)	1.377 (CF_3)	1.445	1.417	172.5	153.6	2.812	18.908
3-Methylindole	N/A	1.441	1.407	180.0	170.2	3.434	9.783
HOMBAP	1.454	1.446	1.415	173.8	177.4	3.083	-3.581

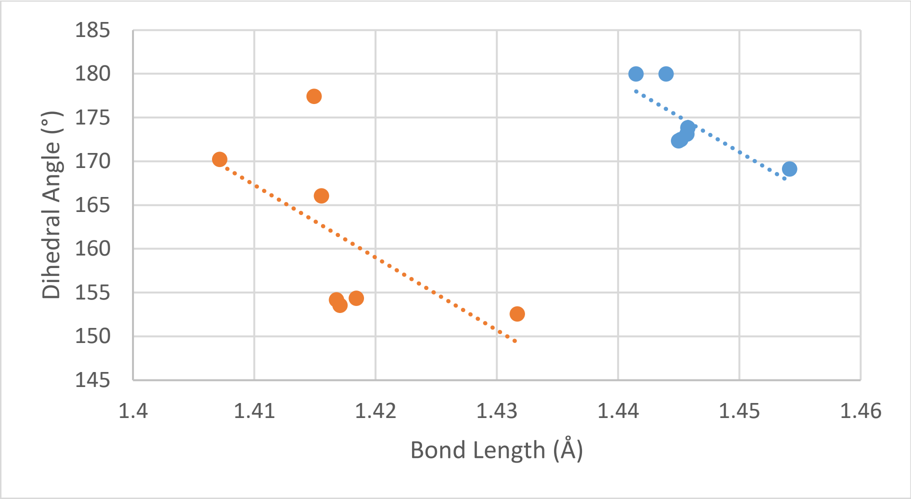

Chart S1. Correlation between *N*-CR_3 bond length and dihedral angle; Orange: *N*-CF_3, Blue: *N*-CH_3.

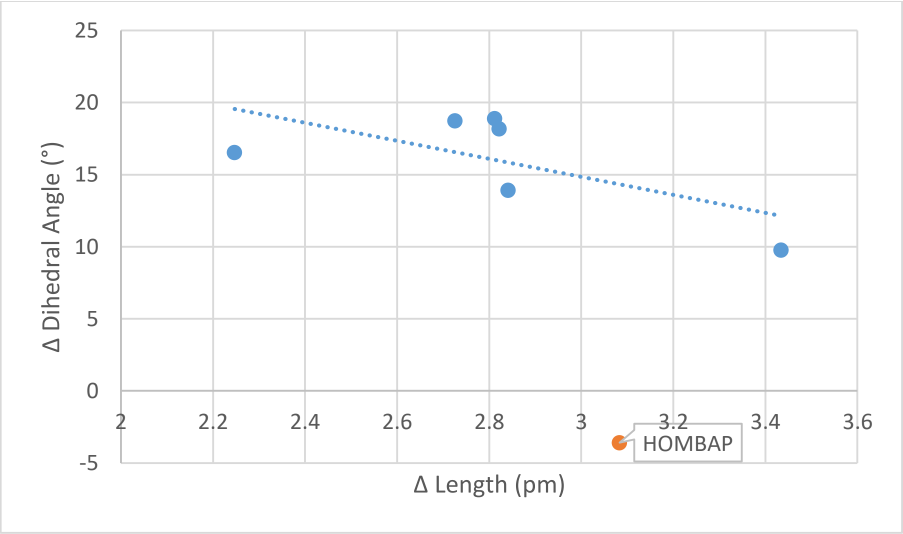

Chart S2. Correlation between *N*-CH_3 and *N*-CF_3 difference in bond length and dihedral angle.

5.1 Coordinates

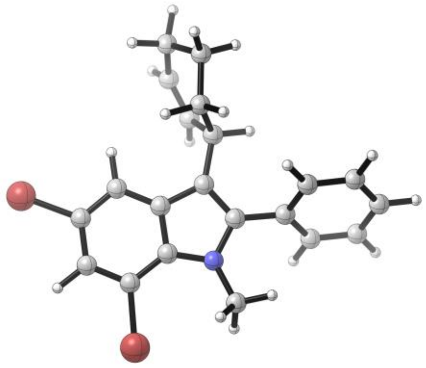

GAZXIR

C	1.474638000	1.126571000	0.055042000
C	0.337401000	0.309179000	0.015936000
C	0.455734000	-1.102805000	-0.048420000
C	1.731629000	-1.684068000	-0.073362000
C	2.851628000	-0.873438000	-0.048712000
C	2.710138000	0.519447000	0.013885000
C	-1.061622000	0.621232000	0.041183000
C	-1.724067000	-0.577994000	-0.007259000
N	-0.816492000	-1.632555000	-0.060326000
H	1.381162000	2.203742000	0.128398000
H	3.838798000	-1.318457000	-0.065932000
C	-1.175273000	-3.013675000	-0.340192000
H	-0.925583000	-3.666229000	0.499803000
H	-2.248622000	-3.061535000	-0.520671000
H	-0.652865000	-3.369458000	-1.230344000
C	-1.665173000	1.996842000	0.133582000
H	-2.739485000	1.869137000	0.331809000
C	-1.516530000	2.773065000	-1.188350000
C	-1.079579000	2.784291000	1.288523000
H	-0.916481000	2.232150000	2.212139000
H	-0.476969000	2.702161000	-1.534409000
H	-2.134777000	2.300464000	-1.960341000
C	-1.895117000	4.243148000	-1.011841000
H	-1.860944000	4.765654000	-1.974157000
H	-2.929506000	4.307282000	-0.648812000
C	-0.758239000	4.076297000	1.222383000
H	-0.325163000	4.561443000	2.095146000
C	-0.964285000	4.924524000	-0.005215000
H	0.010054000	5.130236000	-0.473041000
H	-1.368256000	5.902306000	0.284997000
C	-3.181270000	-0.805548000	0.041815000
C	-4.019027000	-0.209720000	-0.905721000
C	-3.752858000	-1.583998000	1.055533000
C	-5.397988000	-0.382972000	-0.839295000
C	-5.129688000	-1.764375000	1.116093000
C	-5.955842000	-1.162408000	0.169605000
H	-3.579470000	0.384491000	-1.701356000
H	-3.109774000	-2.037829000	1.804642000
H	-6.036288000	0.086703000	-1.581564000
H	-5.559804000	-2.368621000	1.909132000
H	-7.031643000	-1.300525000	0.219727000
Br	2.023831000	-3.560167000	-0.099339000
Br	4.281122000	1.583687000	0.056934000

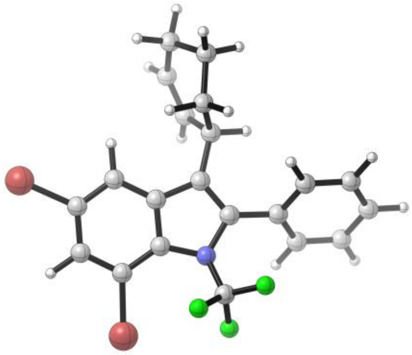

GAZXIR *N*-CF_3

C	-1.807519000	1.140421000	0.006959000
C	-0.555695000	0.520070000	0.039183000
C	-0.438020000	-0.884831000	0.051241000
C	-1.587567000	-1.677640000	-0.042644000
C	-2.829235000	-1.059365000	-0.053845000
C	-2.926519000	0.333623000	-0.013714000
C	0.775565000	1.076560000	0.003926000
C	1.648229000	0.031529000	0.022447000
N	0.930443000	-1.188204000	0.071860000
H	-1.893037000	2.219777000	-0.023285000
H	-3.724688000	-1.664424000	-0.123487000
C	1.468026000	-2.388518000	0.637515000
F	1.615954000	-3.346011000	-0.284479000
F	2.661968000	-2.171715000	1.184058000
F	0.672581000	-2.859564000	1.601060000
C	1.107879000	2.539852000	-0.112824000
H	2.177480000	2.612710000	-0.354084000
C	0.869280000	3.276255000	1.218621000
C	0.343474000	3.200444000	-1.242434000
H	0.250544000	2.626290000	-2.162271000
H	-0.126736000	3.019475000	1.603067000
H	1.590958000	2.924660000	1.964862000
C	0.968945000	4.790002000	1.033597000
H	0.874396000	5.299340000	1.998659000
H	1.961592000	5.038710000	0.635731000
C	-0.198917000	4.415270000	-1.159979000
H	-0.740948000	4.813318000	-2.015633000
C	-0.103825000	5.289873000	0.062438000
H	-1.082314000	5.320000000	0.564510000
H	0.108345000	6.322954000	-0.238867000
C	3.109560000	0.074441000	-0.168782000
C	3.906571000	0.902727000	0.624490000
C	3.706960000	-0.676628000	-1.187184000
C	5.276310000	0.989381000	0.396377000
C	5.075419000	-0.593752000	-1.408469000
C	5.863327000	0.242266000	-0.619888000
H	3.451037000	1.465275000	1.433967000
H	3.091572000	-1.325934000	-1.803166000
H	5.886000000	1.635079000	1.020983000
H	5.528475000	-1.180279000	-2.201859000
H	6.932775000	0.307174000	-0.795591000
Br	-1.562246000	-3.551661000	-0.275444000
Br	-4.648798000	1.123862000	-0.039828000

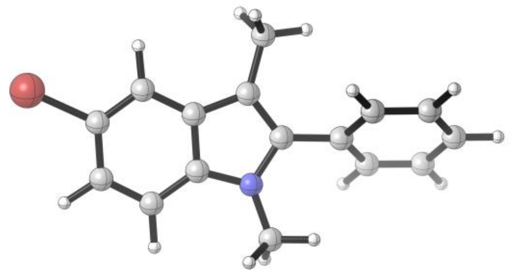 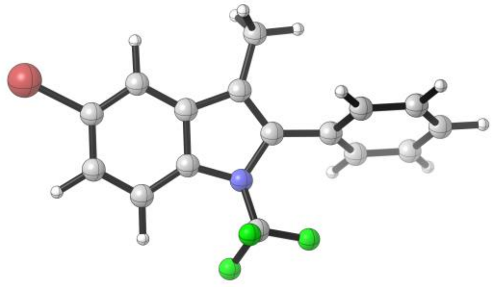

ICOPAU

C	1.906581000	-0.795274000	-0.080030000
C	0.587486000	-0.327582000	-0.019633000
C	0.345757000	1.056993000	0.112863000
C	1.386183000	1.985151000	0.200121000
C	2.684730000	1.507931000	0.144281000
C	2.927360000	0.131679000	0.003278000
C	-0.688156000	-0.981250000	-0.068560000
C	-1.635165000	0.009227000	0.032369000
N	-1.013303000	1.251200000	0.138952000
H	2.125103000	-1.852723000	-0.184922000
H	1.195658000	3.049175000	0.300716000
H	3.522038000	2.193832000	0.205096000
C	-0.907969000	-2.449999000	-0.254128000
H	-0.624976000	-3.018064000	0.640660000
H	-1.955974000	-2.672280000	-0.473598000
H	-0.303229000	-2.832051000	-1.084835000
C	-1.636417000	2.527498000	0.408731000
H	-1.573036000	3.192947000	-0.459843000
H	-2.686634000	2.375819000	0.658409000
H	-1.143346000	3.009632000	1.259080000
C	-3.102428000	-0.125566000	-0.004012000
C	-3.748637000	-1.006203000	0.869884000
C	-3.872144000	0.594689000	-0.926124000
C	-5.129755000	-1.166154000	0.821071000
C	-5.253236000	0.441815000	-0.967790000
C	-5.885681000	-0.440436000	-0.095050000
H	-3.158807000	-1.558126000	1.595667000
H	-3.378345000	1.264980000	-1.624413000
H	-5.616687000	-1.853786000	1.506046000
H	-5.836228000	1.005006000	-1.690416000
H	-6.964057000	-0.562365000	-0.130426000
Br	4.732017000	-0.465015000	-0.072957000

ICOPAU *N*-CF_3

C	2.182952000	-1.047299000	-0.000481000
C	0.854484000	-0.612983000	-0.028752000
C	0.561115000	0.753822000	-0.151430000
C	1.568986000	1.715570000	-0.215378000
C	2.883444000	1.277208000	-0.170668000
C	3.176504000	-0.089049000	-0.070597000
C	-0.394153000	-1.329511000	0.039650000
C	-1.394614000	-0.408658000	-0.036300000
N	-0.834664000	0.884075000	-0.196019000
H	2.428670000	-2.100457000	0.081707000
H	1.350756000	2.771900000	-0.299419000
H	3.693678000	1.995604000	-0.219502000
C	-0.524573000	-2.817467000	0.106972000
H	-0.126345000	-3.205305000	1.051902000
H	-1.568162000	-3.130663000	0.025516000
H	0.038887000	-3.293281000	-0.703949000
C	-1.455822000	2.089870000	0.218871000
F	-0.941703000	3.126481000	-0.457884000
F	-2.765895000	2.071848000	0.003418000
F	-1.262728000	2.338132000	1.524382000
C	-2.846016000	-0.682621000	-0.076849000
C	-3.462338000	-1.308199000	1.008647000
C	-3.601448000	-0.394305000	-1.218274000
C	-4.814041000	-1.637983000	0.957410000
C	-4.950353000	-0.719859000	-1.266570000
C	-5.559917000	-1.342949000	-0.178710000
H	-2.876797000	-1.529246000	1.896277000
H	-3.123211000	0.086197000	-2.066444000
H	-5.283467000	-2.121511000	1.808688000
H	-5.528105000	-0.491778000	-2.157134000
H	-6.614645000	-1.597778000	-0.219090000
Br	4.995402000	-0.630682000	-0.027455000

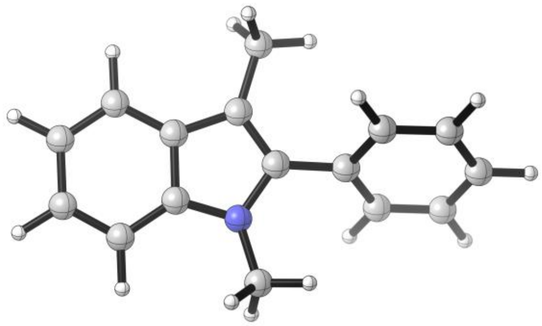

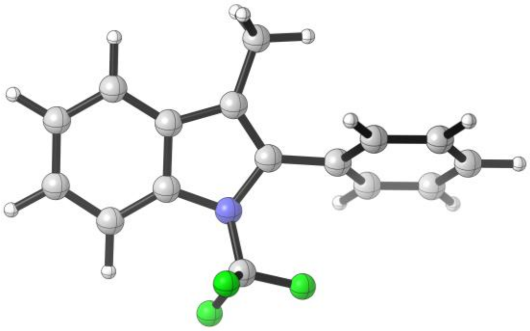

INUXIA

C	-3.114633000	1.376354000	-0.181534000
C	-1.871925000	0.735130000	-0.088037000
C	-1.831002000	-0.666897000	0.070839000
C	-2.992507000	-1.440107000	0.154069000
C	-4.207053000	-0.779372000	0.064510000
C	-4.269319000	0.616196000	-0.104512000
C	-0.513870000	1.199135000	-0.120267000
C	0.281966000	0.087743000	0.015739000
N	-0.512490000	-1.051897000	0.126516000
H	-3.168889000	2.454363000	-0.307976000
H	-2.949648000	-2.518447000	0.276165000
H	-5.129013000	-1.350499000	0.122035000
H	-5.239135000	1.099894000	-0.173652000
C	-0.084048000	2.618060000	-0.326609000
H	-0.296591000	3.238164000	0.553274000
H	0.988095000	2.686194000	-0.531879000
H	-0.616657000	3.066342000	-1.173621000
C	-0.084452000	-2.395084000	0.443768000
H	-0.241301000	-3.073327000	-0.403076000
H	0.975812000	-2.391486000	0.697499000
H	-0.646651000	-2.772492000	1.304529000
C	1.753660000	0.013306000	0.004564000
C	2.504772000	0.818454000	0.867747000
C	2.429894000	-0.833311000	-0.883168000
C	3.895251000	0.782538000	0.841273000
C	3.819262000	-0.876989000	-0.901857000
C	4.556276000	-0.067499000	-0.040651000
H	1.987110000	1.467137000	1.568113000
H	1.857603000	-1.447133000	-1.573446000
H	4.463194000	1.414449000	1.517670000
H	4.328304000	-1.536904000	-1.598163000
H	5.641560000	-0.098916000	-0.058318000

INUXIA *N*-CF_3

C	-2.992619000	1.983765000	-0.045801000
C	-1.804681000	1.245583000	-0.054843000
C	-1.852087000	-0.151968000	-0.170720000
C	-3.060925000	-0.844004000	-0.244708000
C	-4.227386000	-0.093352000	-0.217957000
C	-4.198111000	1.306482000	-0.126058000
C	-0.417991000	1.633734000	0.027763000
C	0.329351000	0.497000000	-0.032004000
N	-0.528599000	-0.621028000	-0.195093000
H	-2.965596000	3.066968000	0.030808000
H	-3.098415000	-1.922875000	-0.322673000
H	-5.183356000	-0.604662000	-0.275946000
H	-5.131615000	1.860693000	-0.116042000
C	0.071445000	3.045579000	0.087179000
H	-0.216476000	3.523280000	1.031182000
H	1.159574000	3.096488000	0.000533000
H	-0.363909000	3.640457000	-0.724139000
C	-0.235330000	-1.938377000	0.236018000
F	-0.936327000	-2.825918000	-0.485202000
F	1.053416000	-2.230851000	0.100249000
F	-0.561230000	-2.138813000	1.524262000
C	1.803440000	0.408974000	-0.071278000
C	2.556326000	0.901533000	0.996581000
C	2.465307000	-0.091703000	-1.197637000
C	3.947627000	0.892641000	0.942639000
C	3.852723000	-0.104976000	-1.248433000
C	4.597862000	0.387966000	-0.178312000
H	2.043786000	1.285691000	1.873711000
H	1.883157000	-0.469478000	-2.032775000
H	4.521821000	1.275834000	1.780797000
H	4.355919000	-0.496177000	-2.127561000
H	5.682869000	0.377962000	-0.220597000

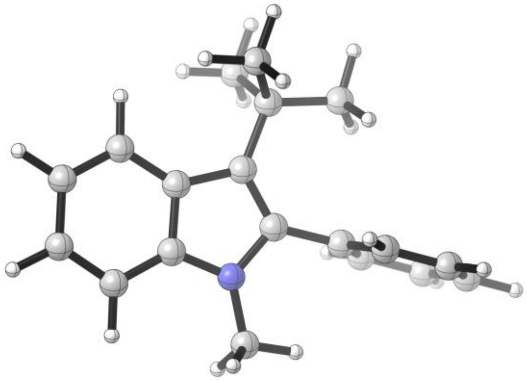

SEFTOM

C	-3.203216000	0.621447000	0.000006000
C	-1.891686000	0.114506000	0.000000000
C	-1.729697000	-1.288175000	0.000028000
C	-2.806202000	-2.180302000	0.000048000
C	-4.081749000	-1.644655000	0.000052000
C	-4.276656000	-0.252675000	0.000034000
C	-0.566192000	0.701751000	-0.000006000
C	0.312017000	-0.358613000	-0.000014000
N	-0.387404000	-1.561794000	0.000038000
H	-3.390568000	1.689953000	-0.000031000
H	-2.647919000	-3.254748000	0.000040000
H	-4.942170000	-2.307187000	0.000071000
H	-5.287745000	0.143461000	0.000041000
C	-0.286610000	2.199375000	-0.000026000
C	0.160940000	-2.897576000	-0.000182000
H	-0.164192000	-3.447020000	-0.890755000
H	1.249573000	-2.845805000	-0.000017000
H	-0.164469000	-3.447438000	0.890131000
C	1.794859000	-0.418163000	0.000000000
C	2.501708000	-0.469591000	1.204013000
C	2.501759000	-0.469582000	-1.203980000
C	3.890874000	-0.548768000	1.204950000
C	3.890926000	-0.548757000	-1.204856000
C	4.587844000	-0.586004000	0.000061000
H	1.952445000	-0.434795000	2.140656000
H	1.952540000	-0.434779000	-2.140648000
H	4.429894000	-0.579424000	2.147215000
H	4.429985000	-0.579407000	-2.147099000
H	5.672170000	-0.645088000	0.000085000
C	-0.904989000	2.832301000	1.263057000
H	-1.981535000	2.656750000	1.333024000
H	-0.442277000	2.413161000	2.163267000
H	-0.738303000	3.916287000	1.265635000
C	-0.904921000	2.832257000	-1.263149000
H	-0.442213000	2.413042000	-2.163327000
H	-1.981475000	2.656766000	-1.333126000
H	-0.738174000	3.916234000	-1.265781000
C	1.208425000	2.550363000	0.000012000
H	1.721971000	2.163247000	0.884702000
H	1.722006000	2.163331000	-0.884695000
H	1.317689000	3.641191000	0.000058000

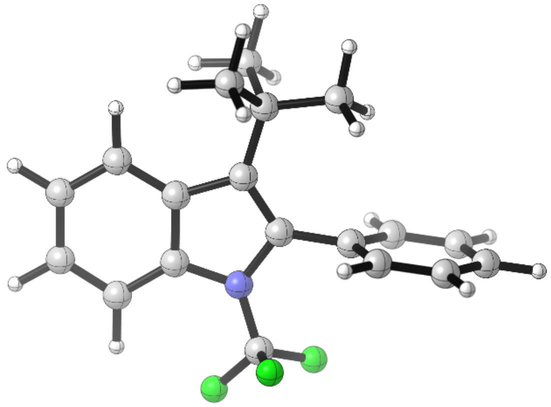

SEFTOM *N*-CF_3

C	-3.270387000	1.013267000	-0.009204000
C	-1.965498000	0.494739000	-0.037171000
C	-1.799644000	-0.901968000	-0.103632000
C	-2.887691000	-1.780449000	-0.127607000
C	-4.159098000	-1.234550000	-0.087530000
C	-4.352065000	0.152026000	-0.032288000
C	-0.646531000	1.110832000	-0.011136000
C	0.257281000	0.088577000	-0.067687000
N	-0.425401000	-1.146554000	-0.156849000
H	-3.444694000	2.081908000	0.033337000
H	-2.756571000	-2.851504000	-0.175304000
H	-5.018361000	-1.897935000	-0.102864000
H	-5.359926000	0.554668000	-0.007599000
C	-0.410555000	2.616254000	0.068413000
C	0.207237000	-2.396728000	0.044602000
F	-0.626769000	-3.402509000	-0.230343000
F	1.276398000	-2.540004000	-0.740222000
F	0.621822000	-2.557843000	1.313626000
C	1.742358000	0.100525000	-0.062317000
C	2.443164000	-0.012788000	1.138848000
C	2.450939000	0.223831000	-1.259014000
C	3.834312000	0.016511000	1.145736000
C	3.840301000	0.254116000	-1.252807000
C	4.534257000	0.153000000	-0.049180000
H	1.891921000	-0.116160000	2.068390000
H	1.902919000	0.302332000	-2.193389000
H	4.371118000	-0.065804000	2.085932000
H	4.382863000	0.356480000	-2.187757000
H	5.619853000	0.178231000	-0.043818000
C	-1.049092000	3.156937000	1.364820000
H	-2.118465000	2.944998000	1.432990000
H	-0.566575000	2.708336000	2.239923000
H	-0.916829000	4.243624000	1.422441000
C	-1.041201000	3.293912000	-1.165518000
H	-0.556051000	2.939627000	-2.081540000
H	-2.110861000	3.092724000	-1.259806000
H	-0.906429000	4.380271000	-1.106922000
C	1.070325000	3.024559000	0.097410000
H	1.600151000	2.600261000	0.954326000
H	1.600502000	2.725066000	-0.810135000
H	1.129702000	4.116342000	0.173750000

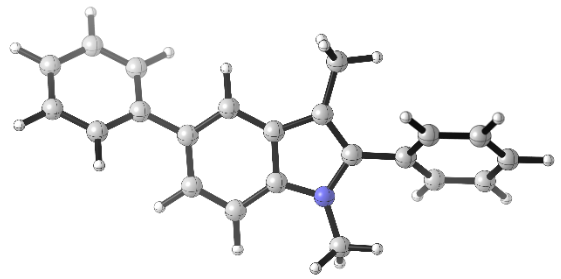

28 *N*-CH$_3$

C	1.494703000	-0.600234000	0.007508000
C	0.152451000	-0.205353000	0.012859000
C	-0.164760000	1.168642000	-0.023838000
C	0.827585000	2.151667000	-0.052589000
C	2.145943000	1.732085000	-0.052175000
C	2.497896000	0.362765000	-0.024578000
C	-1.087865000	-0.926874000	0.054659000
C	-2.087545000	0.015202000	0.037303000
N	-1.532699000	1.292983000	-0.015727000
H	1.754312000	-1.654591000	0.058253000
H	0.581486000	3.208648000	-0.092030000
H	2.937035000	2.474666000	-0.103410000
C	-1.227895000	-2.417027000	0.052117000
H	-0.918217000	-2.854655000	1.009359000
H	-2.261297000	-2.721764000	-0.136072000
H	-0.598929000	-2.865266000	-0.726037000
C	-2.221841000	2.556861000	0.111688000
H	-2.191611000	3.123569000	-0.826137000
H	-3.263502000	2.379812000	0.380504000
H	-1.756013000	3.157141000	0.900238000
C	-3.545100000	-0.200445000	0.023399000
C	-4.145348000	-1.009916000	0.993873000
C	-4.352060000	0.369712000	-0.969508000
C	-5.515912000	-1.247465000	0.970219000
C	-5.723107000	0.140723000	-0.986575000
C	-6.308892000	-0.670265000	-0.017394000
H	-3.527824000	-1.444008000	1.774580000
H	-3.893881000	0.982266000	-1.741277000
H	-5.966149000	-1.878484000	1.730766000
H	-6.334496000	0.587958000	-1.764777000
H	-7.379312000	-0.852054000	-0.033188000
C	3.928208000	-0.037829000	-0.027699000
C	4.368342000	-1.119567000	-0.799327000
C	4.871013000	0.652611000	0.742892000
C	5.705434000	-1.502026000	-0.797416000
C	6.209537000	0.275041000	0.741933000
C	6.632647000	-0.805248000	-0.027472000
H	3.655425000	-1.651307000	-1.423586000
H	4.545204000	1.480421000	1.366886000
H	6.025670000	-2.341414000	-1.408118000
H	6.922942000	0.821376000	1.352403000
H	7.677444000	-1.101553000	-0.027533000

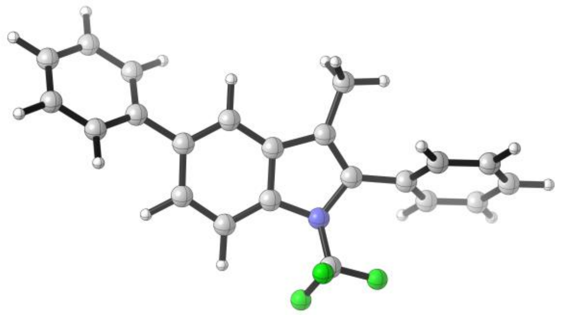

28 *N*-CF$_3$

C	1.834517000	-0.838110000	0.039889000
C	0.483359000	-0.489128000	-0.005775000
C	0.109245000	0.845483000	-0.217553000
C	1.062176000	1.854085000	-0.354834000
C	2.396631000	1.486854000	-0.291082000
C	2.803483000	0.150341000	-0.101126000
C	-0.722640000	-1.269041000	0.127647000
C	-1.776257000	-0.416395000	-0.000933000
N	-1.293479000	0.893534000	-0.256615000
H	2.126434000	-1.869726000	0.216805000
H	0.784271000	2.887314000	-0.517017000
H	3.155690000	2.252325000	-0.422210000
C	-0.764036000	-2.754254000	0.295742000
H	-0.347365000	-3.052565000	1.265035000
H	-1.785933000	-3.136358000	0.234415000
H	-0.167463000	-3.248163000	-0.480054000
C	-1.974618000	2.084244000	0.098794000
F	-1.538038000	3.105402000	-0.652505000
F	-3.286667000	1.978482000	-0.081897000
F	-1.770442000	2.426883000	1.382038000
C	-3.208648000	-0.777149000	-0.018341000
C	-3.782607000	-1.388417000	1.098214000
C	-3.985757000	-0.586991000	-1.166048000
C	-5.111971000	-1.801663000	1.071048000
C	-5.312666000	-0.994937000	-1.190515000
C	-5.879196000	-1.604208000	-0.071914000
H	-3.181574000	-1.531947000	1.991404000
H	-3.540836000	-0.117555000	-2.038194000
H	-5.547693000	-2.273261000	1.946675000
H	-5.906776000	-0.842424000	-2.086539000
H	-6.916754000	-1.923800000	-0.093703000
C	4.247635000	-0.192986000	-0.050731000
C	4.733391000	-1.337365000	-0.692967000
C	5.154346000	0.617038000	0.642017000
C	6.084093000	-1.664464000	-0.641779000
C	6.505858000	0.292997000	0.691134000
C	6.976070000	-0.849785000	0.050012000
H	4.047021000	-1.963375000	-1.256649000
H	4.791416000	1.496976000	1.166138000
H	6.442141000	-2.553753000	-1.152382000
H	7.192428000	0.931231000	1.239764000
H	8.031215000	-1.103449000	0.088697000

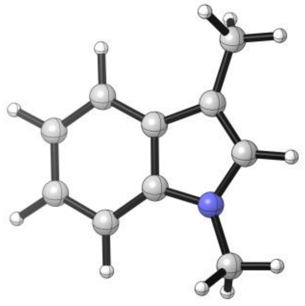

3-Methylindole *N*-CH_3

C	1.237630000	-1.566220000	0.000030000
C	0.098610000	-0.749660000	0.000000000
C	0.261460000	0.655010000	-0.000060000
C	1.521630000	1.259760000	-0.000040000
C	2.628470000	0.426730000	-0.000010000
C	2.489210000	-0.973750000	0.000030000
C	-1.315830000	-1.014530000	0.000010000
C	-1.924940000	0.207110000	-0.000040000
N	-0.989950000	1.224060000	-0.000080000
H	1.137940000	-2.648430000	0.000060000
H	1.632350000	2.340350000	-0.000050000
H	3.623140000	0.862770000	0.000010000
H	3.379670000	-1.595330000	0.000070000
H	-2.980760000	0.446560000	-0.000010000
C	-1.970700000	-2.359680000	0.000010000
H	-1.684810000	-2.944350000	0.882700000
H	-3.061330000	-2.268550000	-0.000020000
H	-1.684760000	-2.944360000	-0.882660000
C	-1.260880000	2.639860000	0.000090000
H	-0.838680000	3.119530000	-0.890190000
H	-2.341330000	2.796360000	-0.000520000
H	-0.839700000	3.119220000	0.891020000

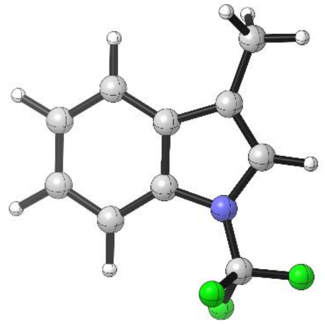

3-Methylindole *N*-CF_3

C	-2.758040000	-0.169980000	0.033620000
C	-1.470960000	0.378130000	-0.012420000
C	-0.358630000	-0.482320000	-0.064810000
C	-0.485840000	-1.871110000	-0.060200000
C	-1.771370000	-2.386920000	-0.005450000
C	-2.897390000	-1.547860000	0.038050000
C	-0.978370000	1.737290000	-0.015860000
C	0.374440000	1.660650000	-0.066190000
N	0.776750000	0.323240000	-0.120450000
H	-3.629880000	0.477060000	0.069000000
H	0.379580000	-2.522370000	-0.098190000
H	-1.908590000	-3.463830000	0.001020000
H	-3.888400000	-1.989510000	0.076170000
H	1.112940000	2.447560000	-0.086010000
C	-1.815490000	2.975650000	0.020610000
H	-2.444170000	3.000230000	0.918190000
H	-1.193260000	3.875120000	0.020970000
H	-2.483080000	3.028000000	-0.847230000
C	2.099590000	-0.137590000	0.012920000
F	2.361230000	-1.112130000	-0.868200000
F	2.957500000	0.863020000	-0.194830000
F	2.346810000	-0.645410000	1.230540000

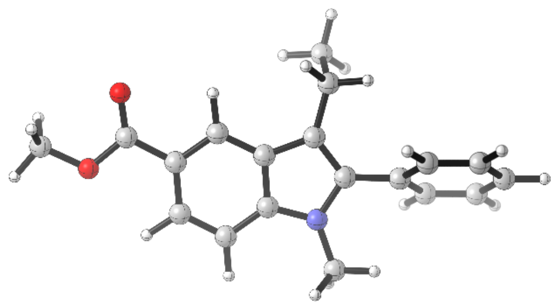

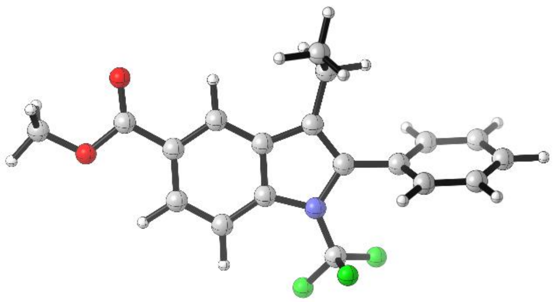

HOMBAP

C	-1.916296000	0.730806000	0.133544000
C	-0.613561000	0.231228000	0.109629000
C	-0.412697000	-1.163351000	-0.002911000
C	-1.479899000	-2.065801000	-0.078476000
C	-2.760425000	-1.547856000	-0.050673000
C	-2.983018000	-0.156966000	0.053307000
C	0.684414000	0.844739000	0.184927000
C	1.598337000	-0.175299000	0.111038000
N	0.936269000	-1.398798000	-0.007247000
H	-2.118860000	1.794115000	0.217453000
H	-1.314230000	-3.135475000	-0.162778000
H	-3.613503000	-2.213480000	-0.110997000
C	0.951506000	2.320342000	0.208212000
H	1.987416000	2.507372000	0.509217000
H	0.315755000	2.792774000	0.967906000
C	1.523218000	-2.719760000	0.020258000
H	1.386697000	-3.230965000	-0.939282000
H	2.590728000	-2.639383000	0.225675000
H	1.059488000	-3.320761000	0.809426000
C	3.071188000	-0.088504000	0.102822000
C	3.748163000	0.503560000	1.173834000
C	3.813886000	-0.571639000	-0.981064000
C	5.134585000	0.617675000	1.158282000
C	5.200370000	-0.466937000	-0.991567000
C	5.863913000	0.130621000	0.077279000
H	3.177089000	0.866854000	2.023178000
H	3.294664000	-1.019204000	-1.824310000
H	5.646425000	1.081351000	1.996258000
H	5.763292000	-0.843770000	-1.840336000
H	6.946317000	0.216386000	0.066951000
C	-4.351990000	0.415719000	0.083173000
O	-4.605776000	1.598018000	0.169208000
O	-5.310834000	-0.527333000	0.003779000
C	-6.646576000	-0.035158000	0.028647000
H	-6.826912000	0.635763000	-0.815213000
H	-7.285997000	-0.915260000	-0.040445000
H	-6.841223000	0.507110000	0.957495000
C	0.694689000	2.980997000	-1.152695000
H	1.358355000	2.556980000	-1.913363000
H	-0.337040000	2.818577000	-1.481115000
H	0.871693000	4.060633000	-1.104277000

HOMBAP *N*-CF_3

C	2.212625000	-0.972394000	-0.167833000
C	0.893590000	-0.520389000	-0.162363000
C	0.625415000	0.860037000	-0.068623000
C	1.657090000	1.799599000	0.032600000
C	2.957447000	1.328268000	0.032594000
C	3.242504000	-0.046811000	-0.070503000
C	-0.364722000	-1.219511000	-0.227897000
C	-1.348199000	-0.281199000	-0.166795000
N	-0.760457000	1.002682000	-0.095999000
H	2.450645000	-2.028831000	-0.242140000
H	1.462433000	2.859639000	0.115135000
H	3.777781000	2.031661000	0.113988000
C	-0.530751000	-2.709202000	-0.244023000
H	0.172634000	-3.142853000	-0.965564000
H	-1.535991000	-2.965771000	-0.592659000
C	-1.483899000	2.212675000	0.024570000
F	-0.729617000	3.252314000	-0.341059000
F	-2.570964000	2.204410000	-0.744946000
F	-1.887527000	2.440072000	1.286772000
C	-2.809123000	-0.509501000	-0.128727000
C	-3.542545000	-0.265719000	1.036107000
C	-3.459782000	-1.033614000	-1.248399000
C	-4.906306000	-0.533198000	1.074434000
C	-4.822834000	-1.307590000	-1.205921000
C	-5.548654000	-1.055376000	-0.045269000
H	-3.040214000	0.133267000	1.911557000
H	-2.889484000	-1.219425000	-2.153775000
H	-5.467399000	-0.339489000	1.983580000
H	-5.318757000	-1.714172000	-2.082092000
H	-6.613281000	-1.266437000	-0.012521000
C	4.635924000	-0.565701000	-0.075144000
O	4.931812000	-1.737634000	-0.157740000
O	5.551834000	0.413527000	0.023399000
C	6.908583000	-0.022428000	0.024395000
H	7.143082000	-0.552604000	-0.902011000
H	7.508259000	0.883635000	0.108510000
H	7.098456000	-0.687652000	0.870432000
C	-0.296726000	-3.330092000	1.139252000
H	0.702991000	-3.090209000	1.515856000
H	-1.027772000	-2.945273000	1.857637000
H	-0.394197000	-4.419686000	1.099164000

6. NMR

Carbamoyl Azides

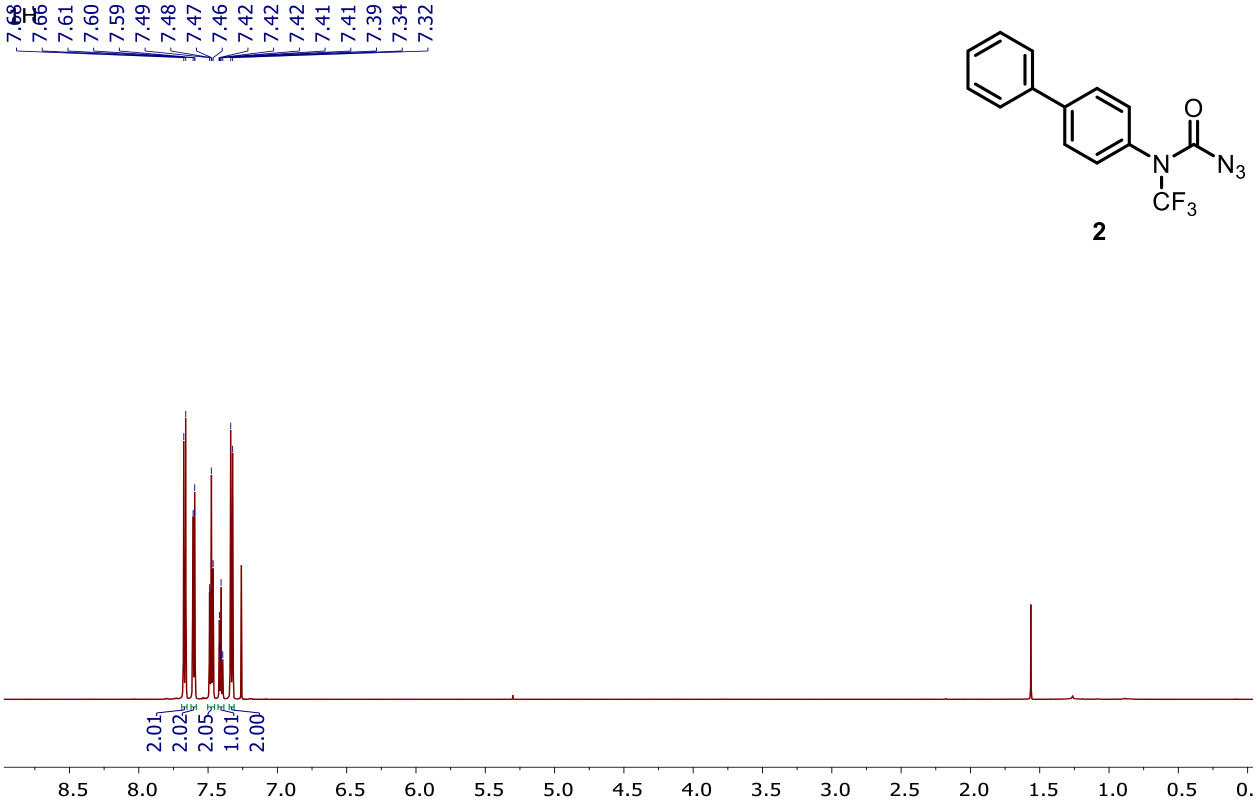

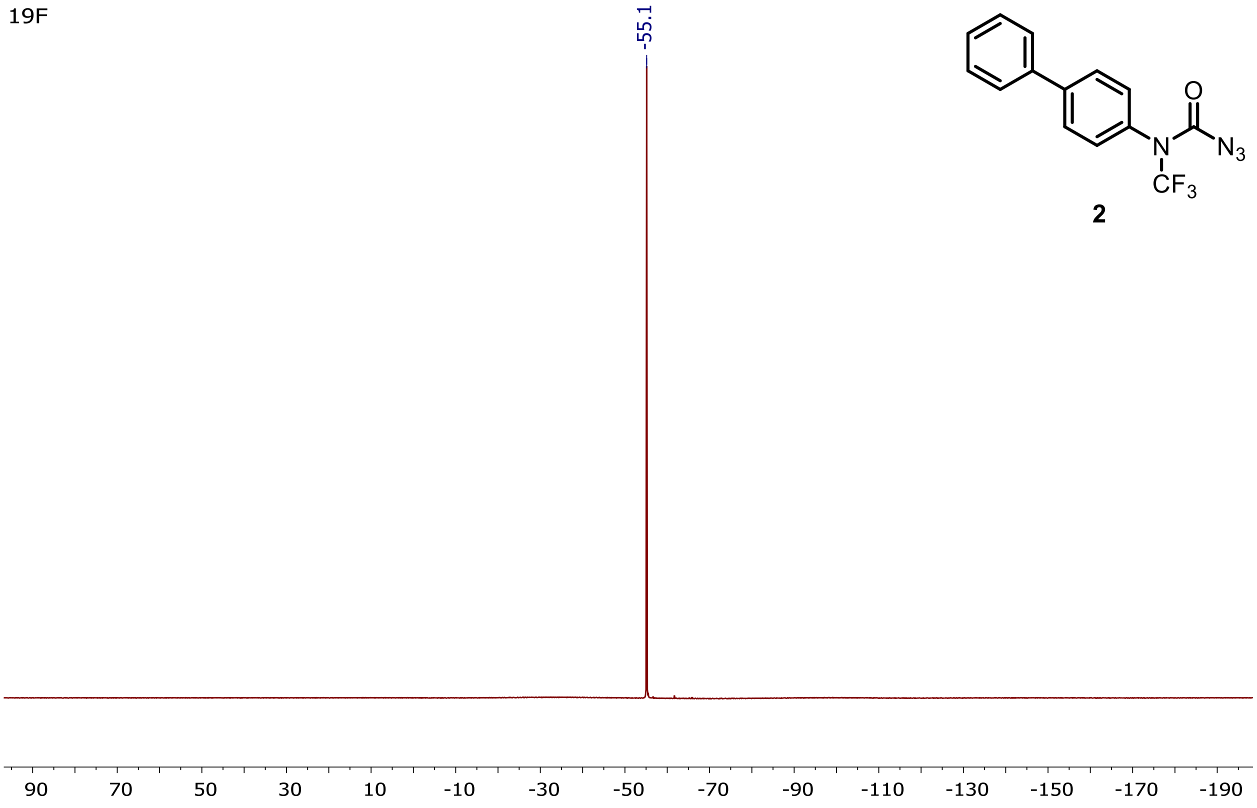

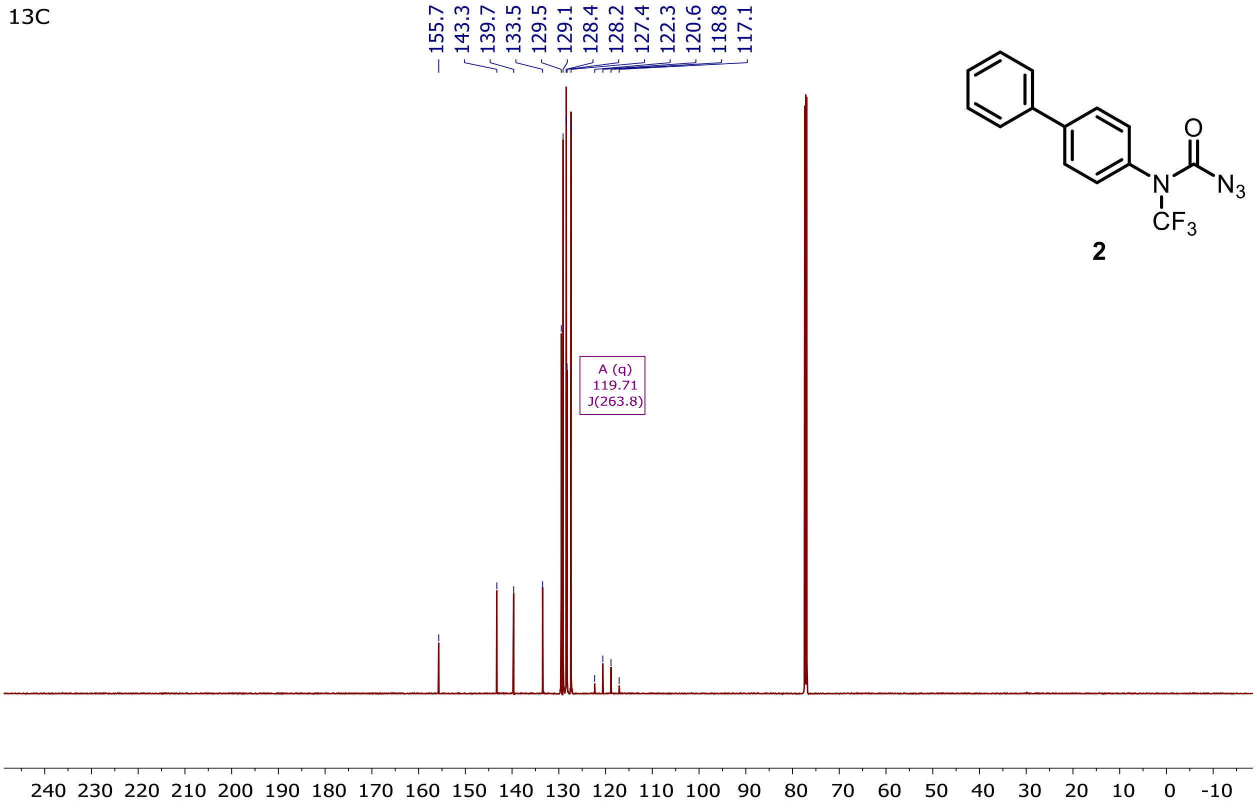
13C
155.7
143.3
139.7
133.5
129.5
129.1
128.4
128.2
127.4
122.3
120.6
118.8
117.1
A (q)
119.71
J(263.8)
240 230 220 210 200 190 180 170 160 150 140 130 120 110 100 90 80 70 60 50 40 30 20 10 0 -10
O
N
N3
CF3

2

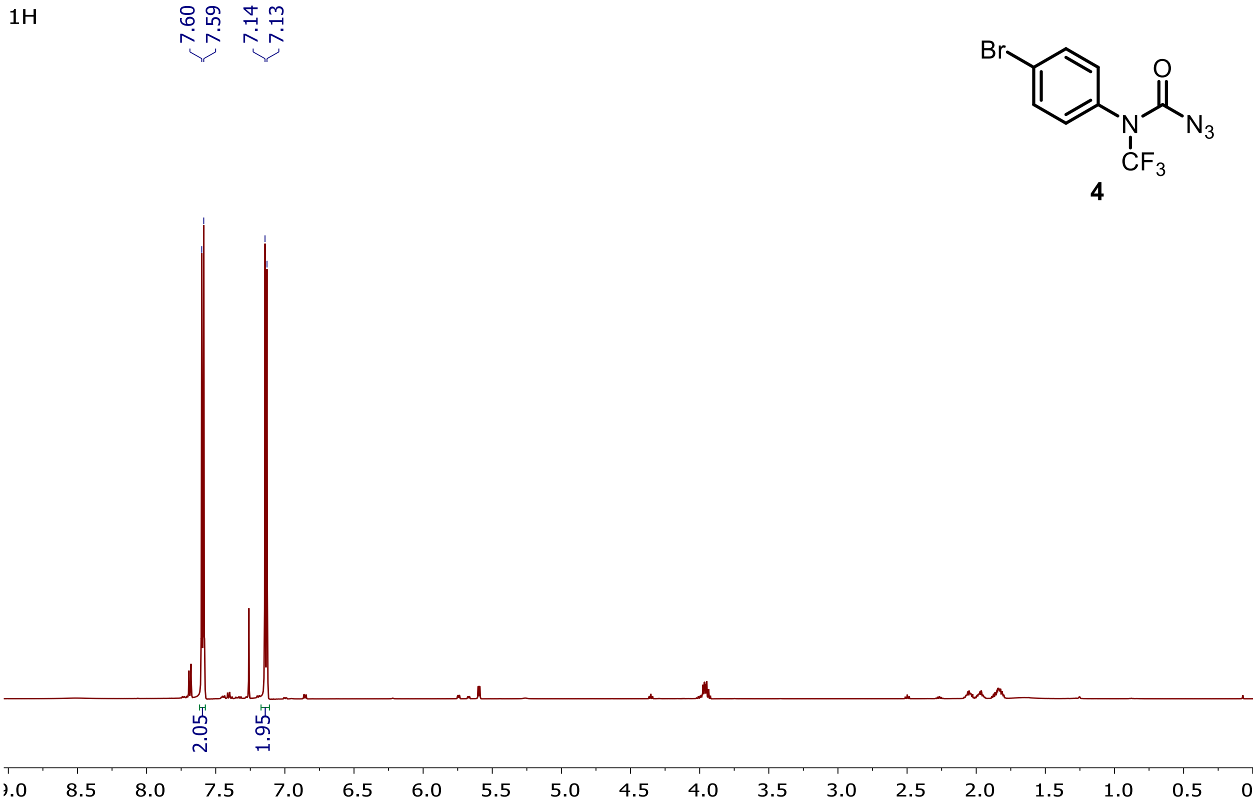
1H
7.60
7.59
7.14
7.13
2.05
1.95
8.5 8.0 7.5 7.0 6.5 6.0 5.5 5.0 4.5 4.0 3.5 3.0 2.5 2.0 1.5 1.0 0.5 0
Br
O
N
N3
CF3

4

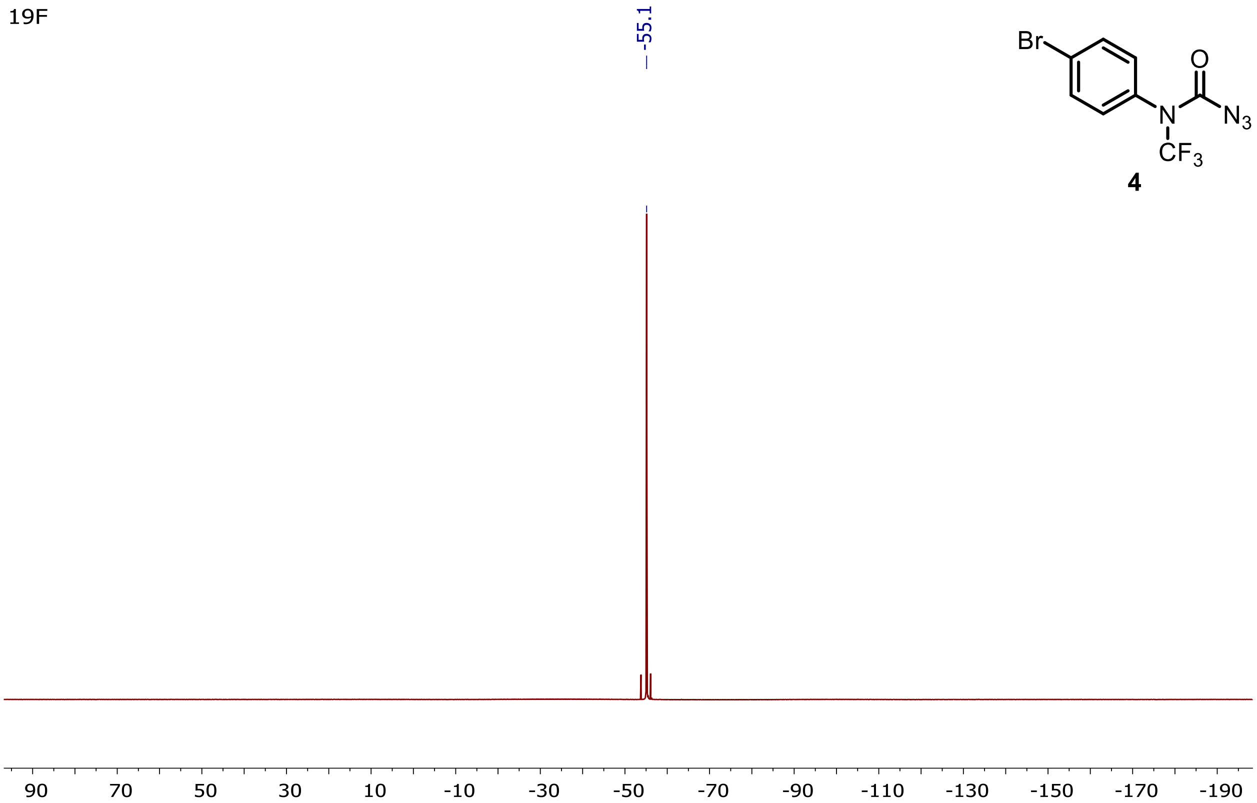
19F
-55.1
Br
O
N
N3
CF3
90 70 50 30 10 -10 -30 -50 -70 -90 -110 -130 -150 -170 -190

4

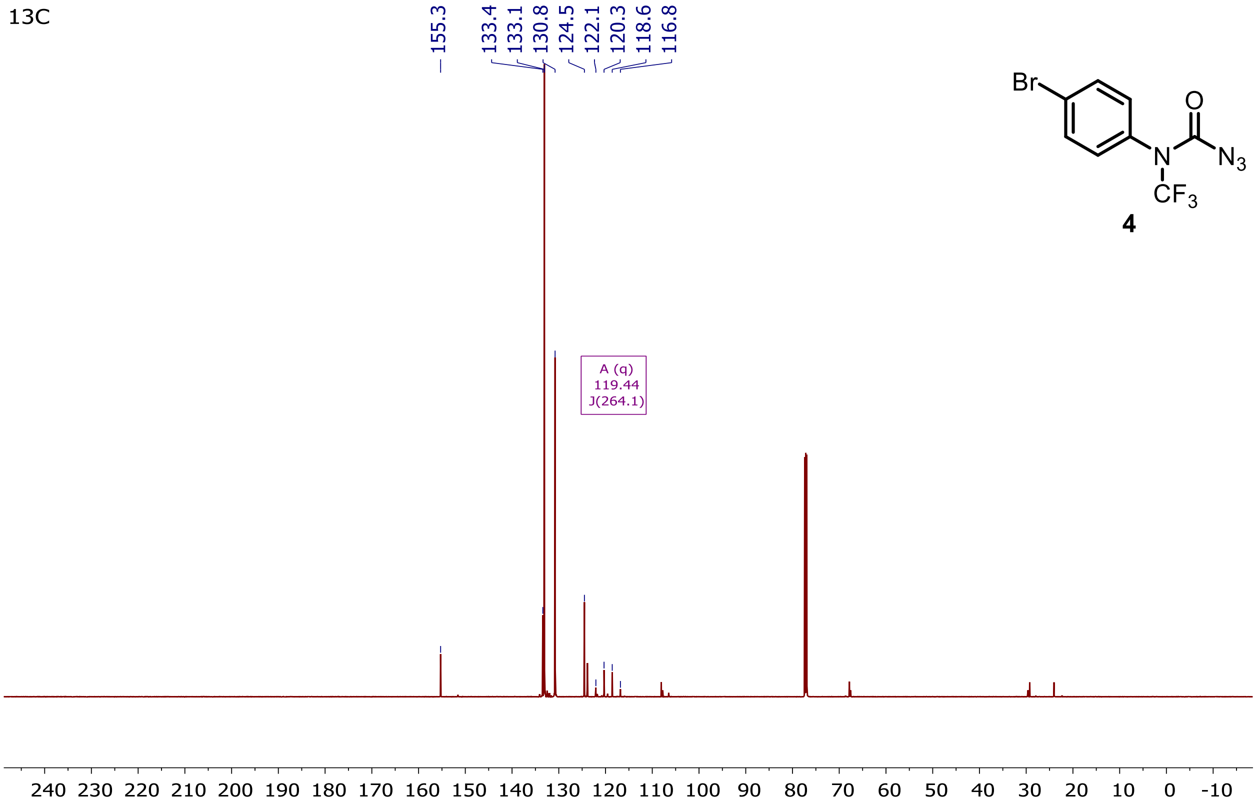
13C
155.3
133.4
133.1
130.8
124.5
122.1
120.3
118.6
116.8
Br
O
N
N3
CF3
A (q)
119.44
J(264.1)
240 230 220 210 200 190 180 170 160 150 140 130 120 110 100 90 80 70 60 50 40 30 20 10 0 -10

4

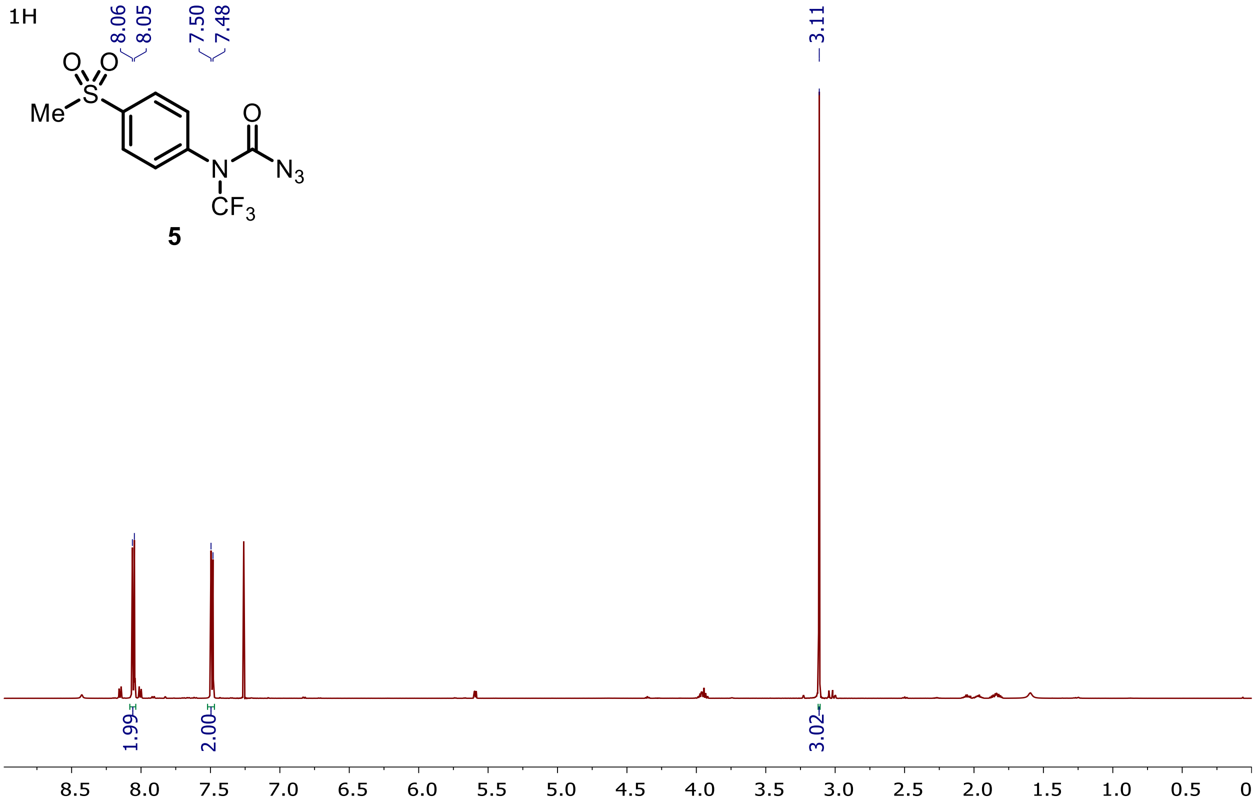
1H
8.06
8.05
7.50
7.48
3.11
O
O
S
Me
O
N
N3
CF3
5
1.99
2.00
3.02
8.5
8.0
7.5
7.0
6.5
6.0
5.5
5.0
4.5
4.0
3.5
3.0
2.5
2.0
1.5
1.0
0.5
0

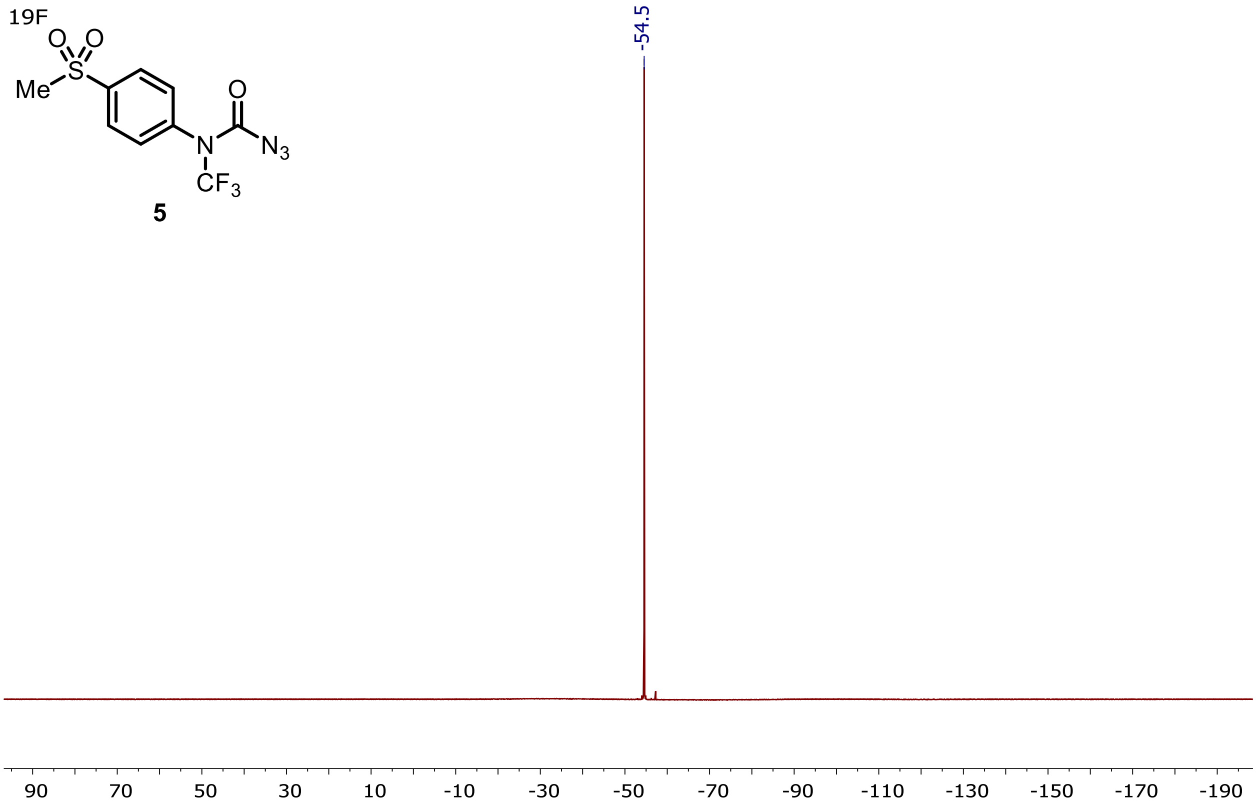
19F
-54.5
O
O
S
Me
O
N
N3
CF3
5
90
70
50
30
10
-10
-30
-50
-70
-90
-110
-130
-150
-170
-190

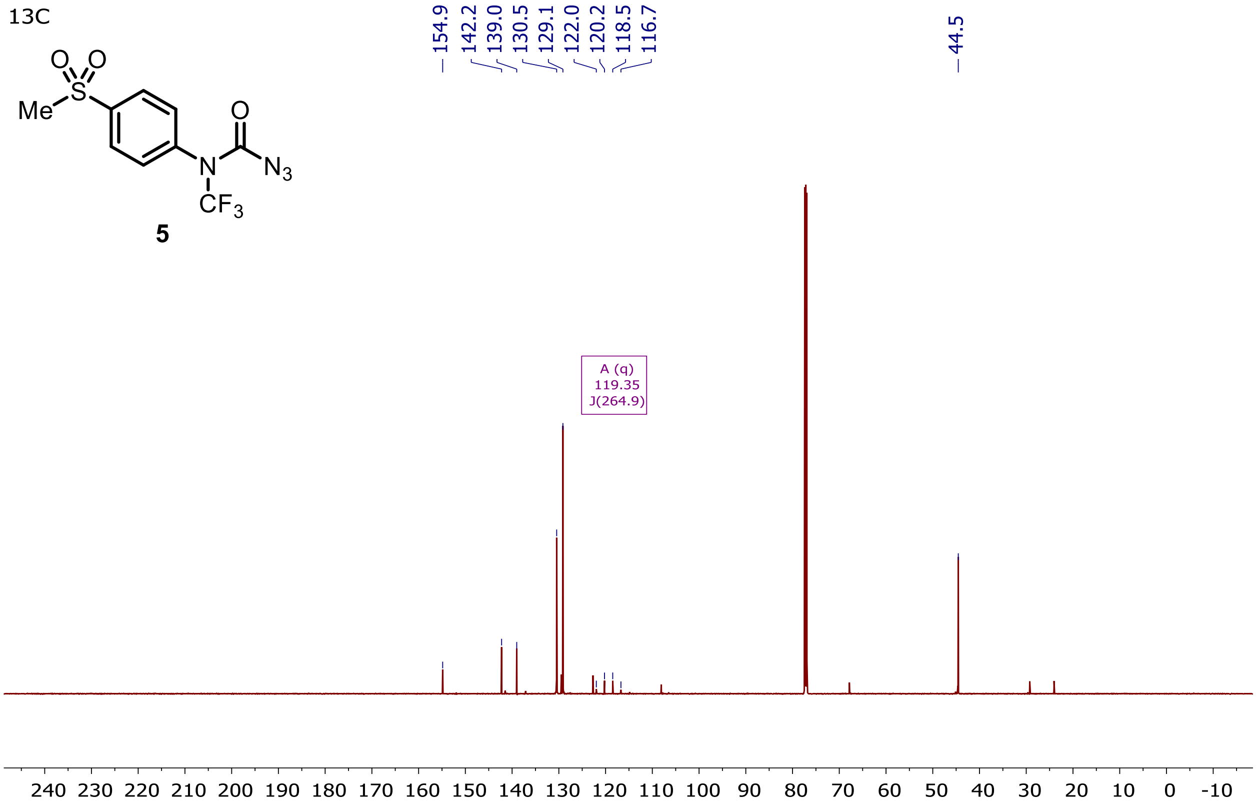

13C
O O
Me S
O
N N3
CF3
5
154.9
142.2
139.0
130.5
129.1
122.0
120.2
118.5
116.7
44.5
A (q)
119.35
J(264.9)
240 230 220 210 200 190 180 170 160 150 140 130 120 110 100 90 80 70 60 50 40 30 20 10 0 -10

Hydrazines

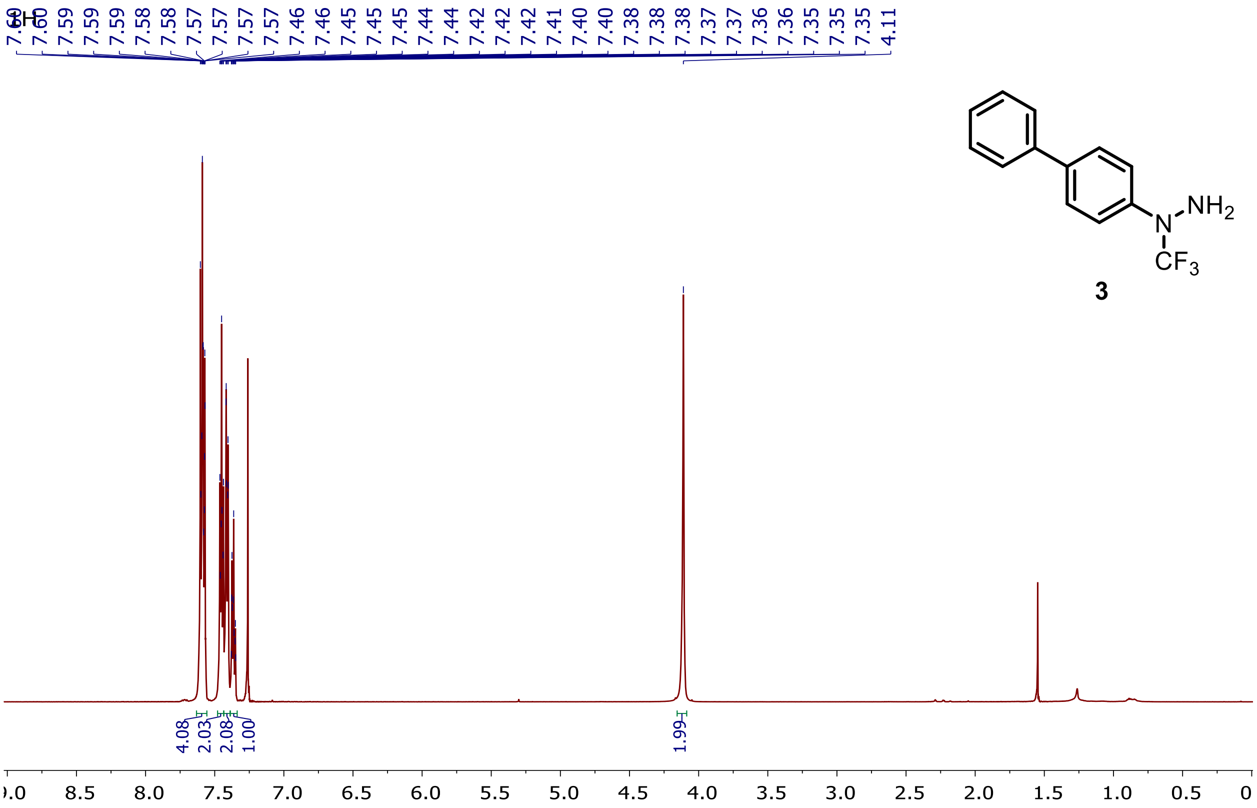
1H
7.60 7.60 7.59 7.59 7.59 7.58 7.58 7.57 7.57 7.57 7.57 7.46 7.46 7.45 7.45 7.45 7.44 7.44 7.42 7.42 7.42 7.41 7.40 7.40 7.38 7.38 7.38 7.37 7.37 7.36 7.36 7.35 7.35 7.35 4.11
4.08 2.03 2.08 1.00 1.99
NH2
N
CF3
3

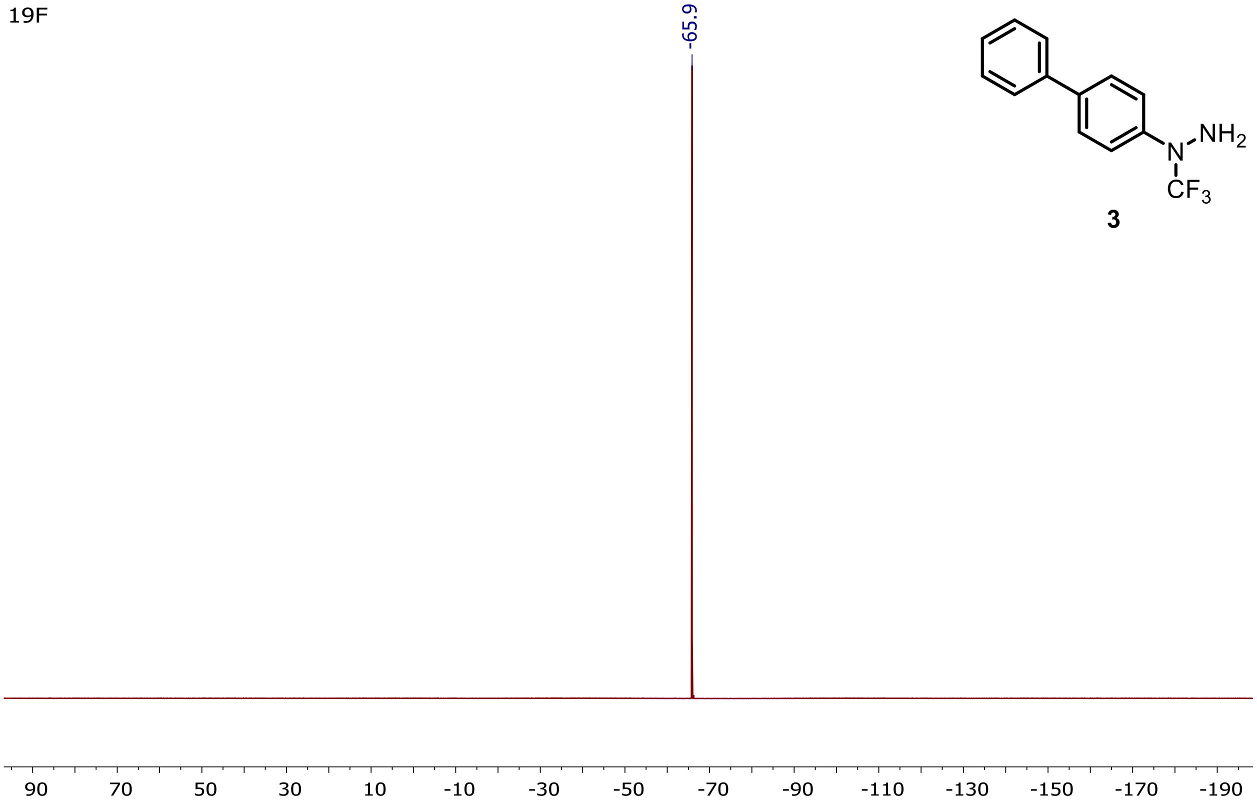
19F
-65.9
NH2
N
CF3
3
90 70 50 30 10 -10 -30 -50 -70 -90 -110 -130 -150 -170 -190

13C

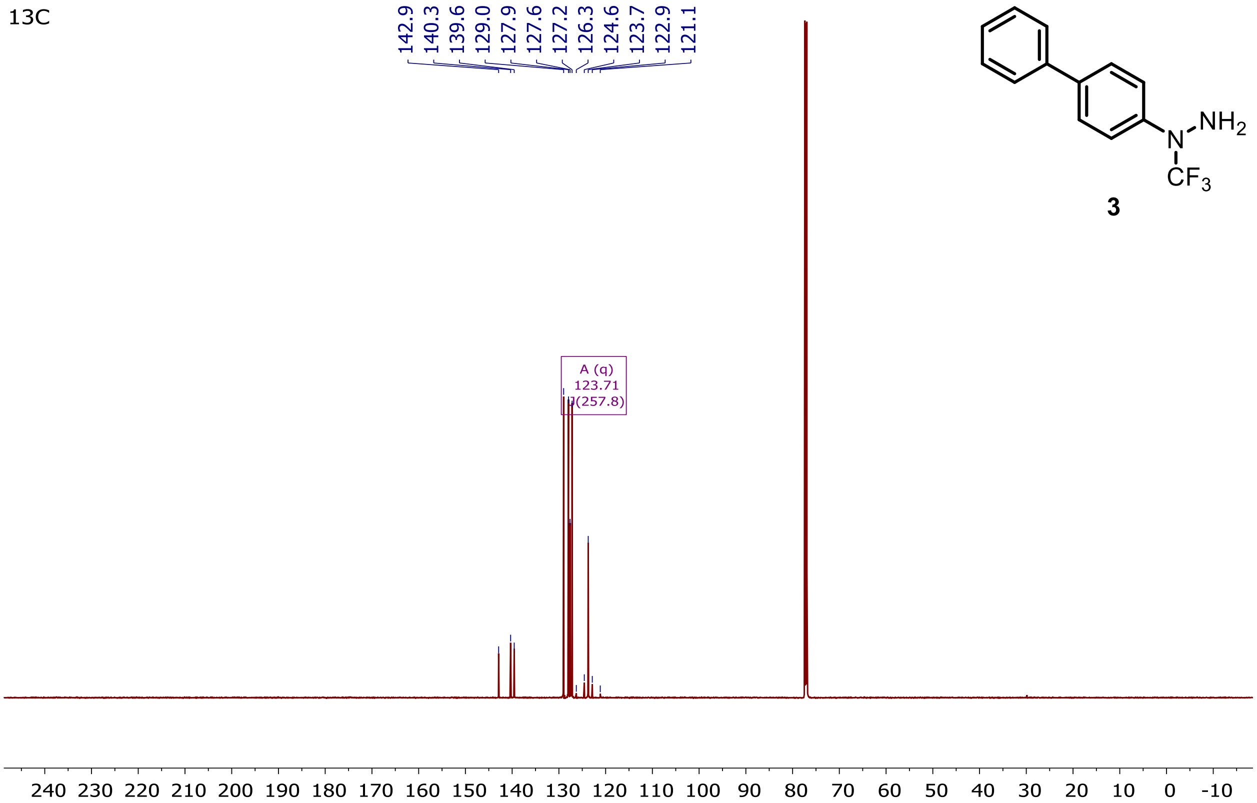
142.9
140.3
139.6
129.0
127.9
127.6
127.2
126.3
124.6
123.7
122.9
121.1
A (q)
123.71
J(257.8)
240 230 220 210 200 190 180 170 160 150 140 130 120 110 100 90 80 70 60 50 40 30 20 10 0 -10
NH2
N
CF3

3

1H

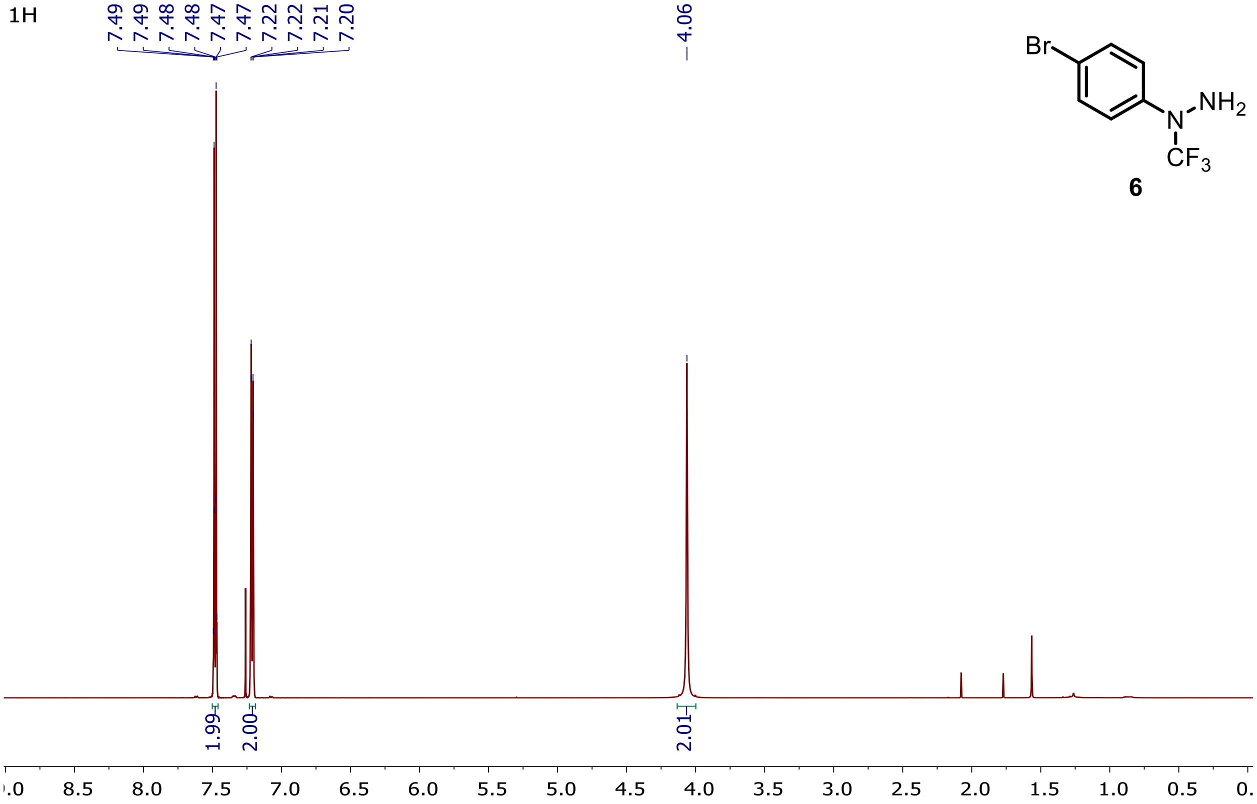
7.49
7.49
7.48
7.48
7.47
7.47
7.22
7.22
7.21
7.20
4.06
1.99
2.00
2.01
8.5 8.0 7.5 7.0 6.5 6.0 5.5 5.0 4.5 4.0 3.5 3.0 2.5 2.0 1.5 1.0 0.5
Br
NH2
N
CF3

6

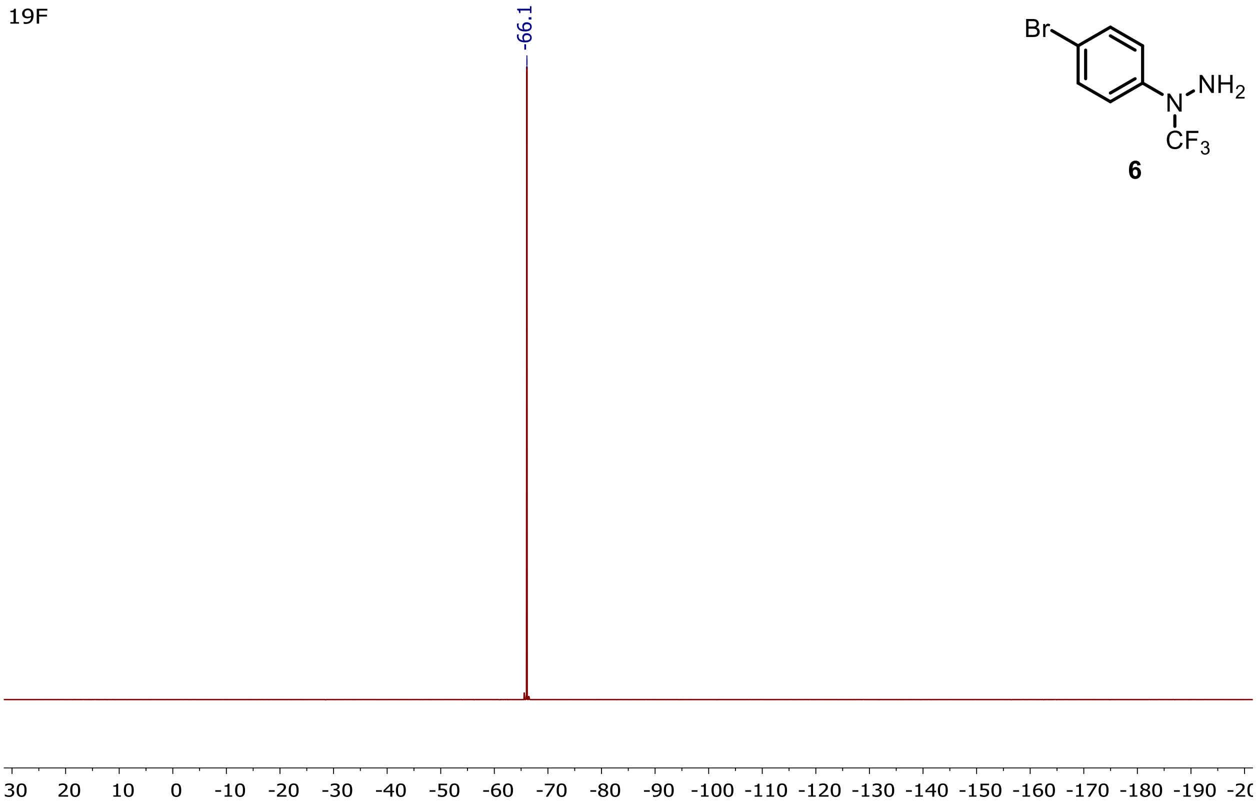
19F
-66.1
Br
NH2
N
CF3
6
30 20 10 0 -10 -20 -30 -40 -50 -60 -70 -80 -90 -100 -110 -120 -130 -140 -150 -160 -170 -180 -190 -20

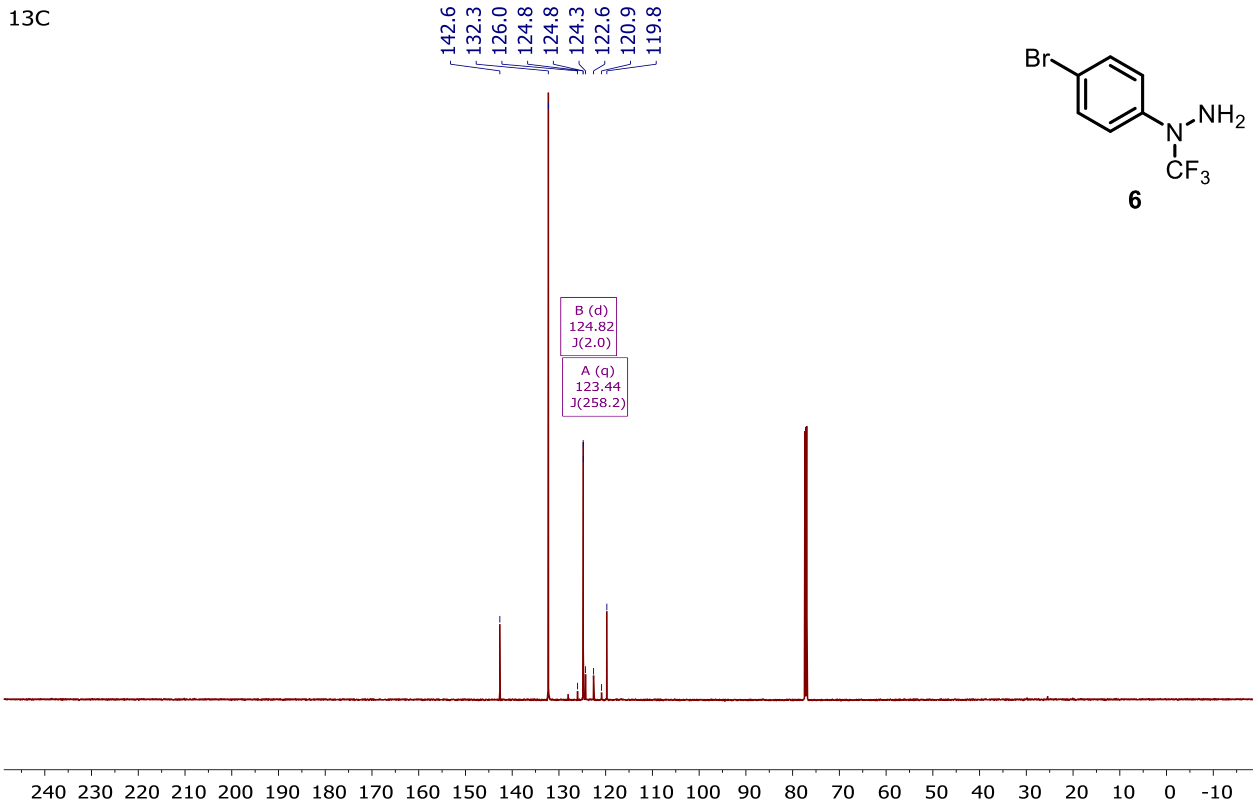
13C
142.6
132.3
126.0
124.8
124.8
124.3
122.6
120.9
119.8
B (d)
124.82
J(2.0)
A (q)
123.44
J(258.2)
Br
NH2
N
CF3
6
240 230 220 210 200 190 180 170 160 150 140 130 120 110 100 90 80 70 60 50 40 30 20 10 0 -10

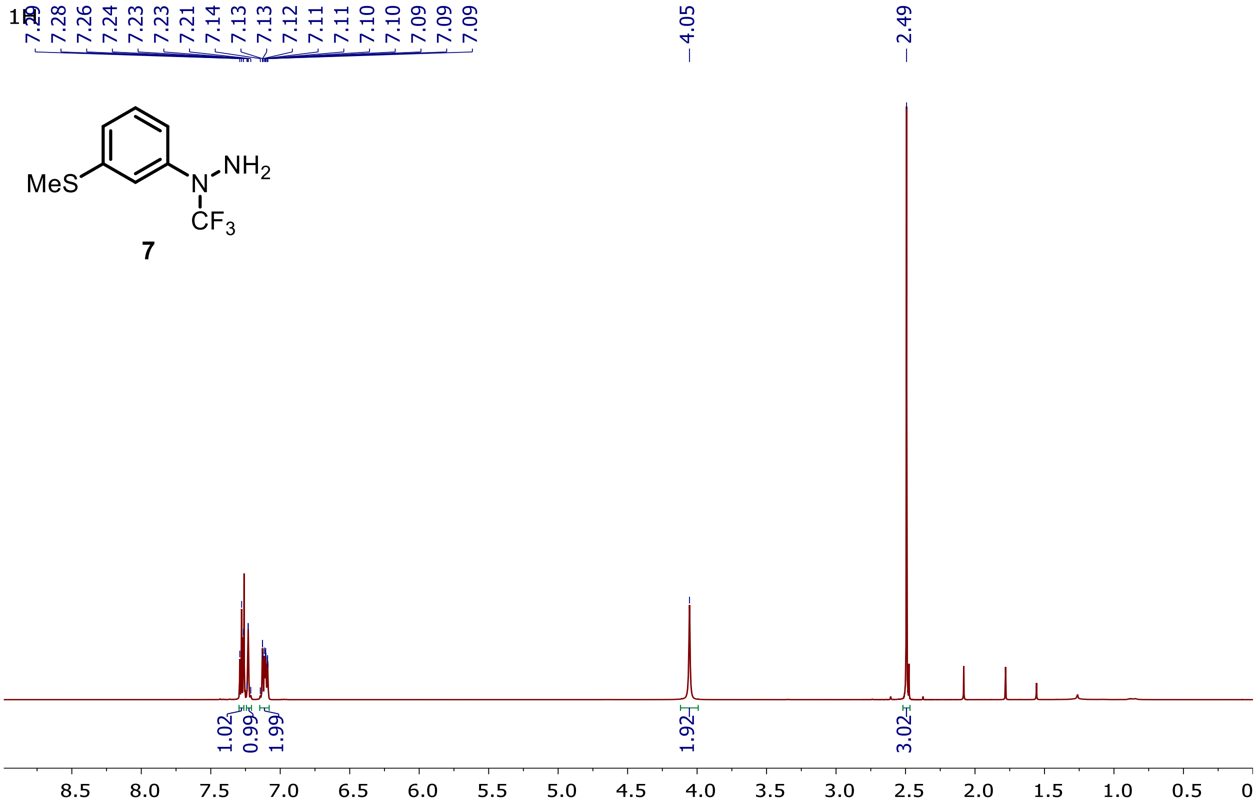
1H
7.29 7.28 7.26 7.24 7.23 7.23 7.21 7.14 7.13 7.13 7.12 7.11 7.11 7.10 7.10 7.09 7.09 7.09
4.05
2.49
MeS
NH2
N
CF3
7
1.02 0.99 1.99
1.92
3.02
8.5 8.0 7.5 7.0 6.5 6.0 5.5 5.0 4.5 4.0 3.5 3.0 2.5 2.0 1.5 1.0 0.5 0

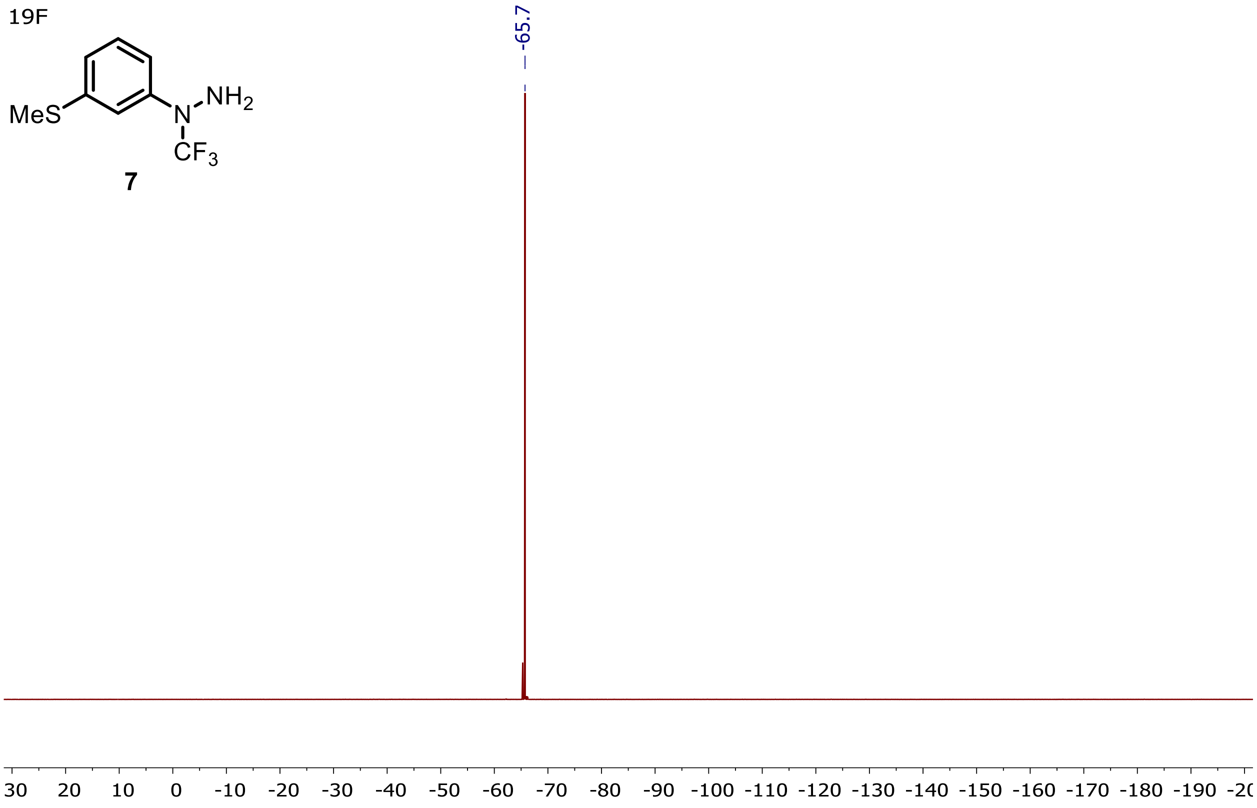
19F
-65.7
MeS
NH2
N
CF3
7
30 20 10 0 -10 -20 -30 -40 -50 -60 -70 -80 -90 -100 -110 -120 -130 -140 -150 -160 -170 -180 -190

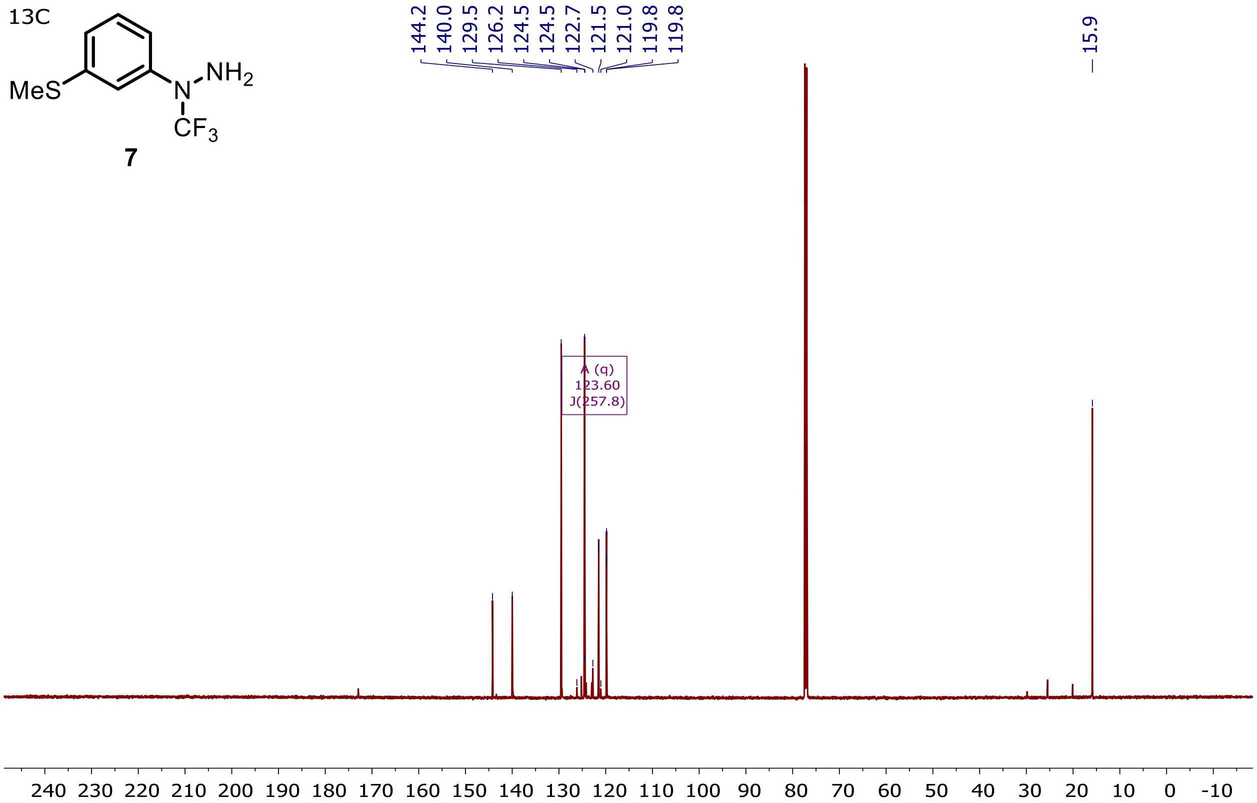
13C
MeS
N
NH$_2$
CF$_3$
7
144.2
140.0
129.5
126.2
124.5
124.5
122.7
121.5
121.0
119.8
119.8
15.9
A (q)
123.60
J(257.8)
240 230 220 210 200 190 180 170 160 150 140 130 120 110 100 90 80 70 60 50 40 30 20 10 0 -10

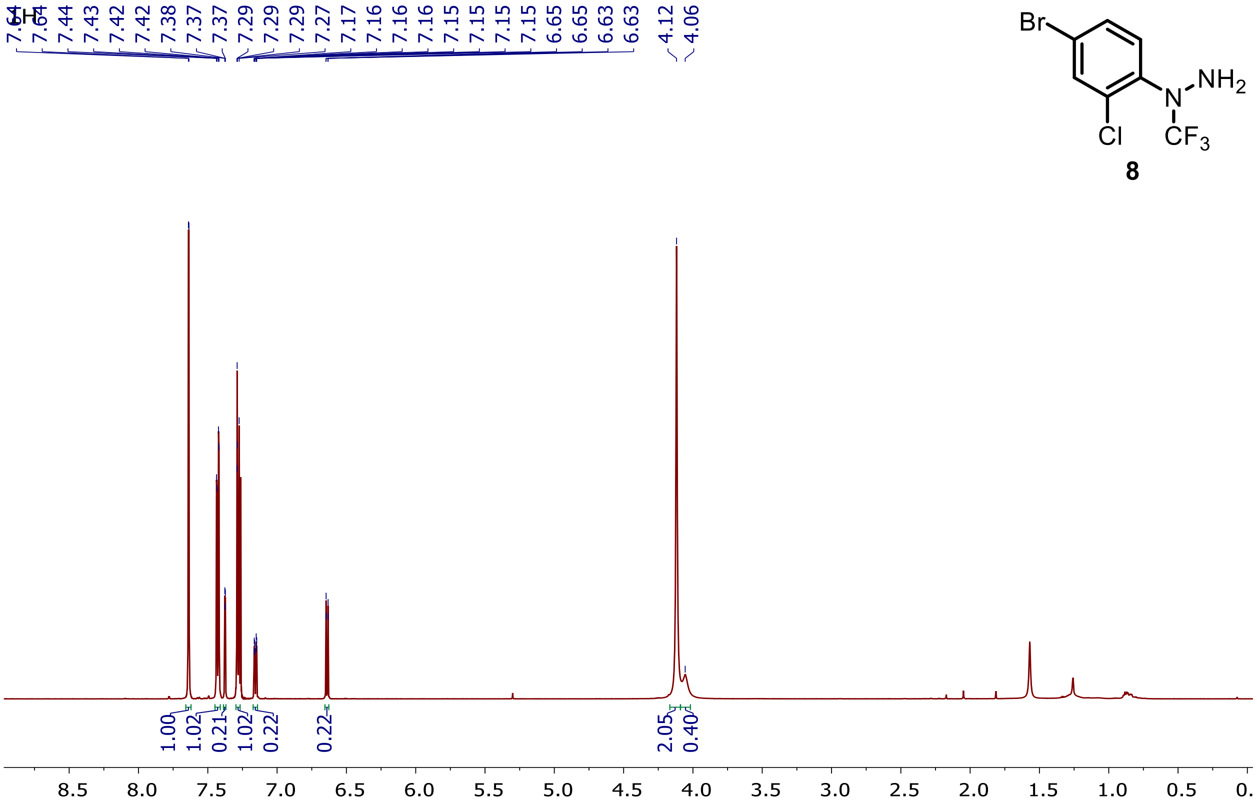
1H
7.64
7.64
7.44
7.43
7.42
7.42
7.38
7.37
7.37
7.29
7.29
7.29
7.27
7.17
7.16
7.16
7.15
7.15
7.15
7.15
6.65
6.65
6.63
6.63
4.12
4.06
Br
N
NH$_2$
Cl
CF$_3$
8
1.00
1.02
0.21
1.02
0.22
0.22
2.05
0.40
8.5 8.0 7.5 7.0 6.5 6.0 5.5 5.0 4.5 4.0 3.5 3.0 2.5 2.0 1.5 1.0 0.5 0.

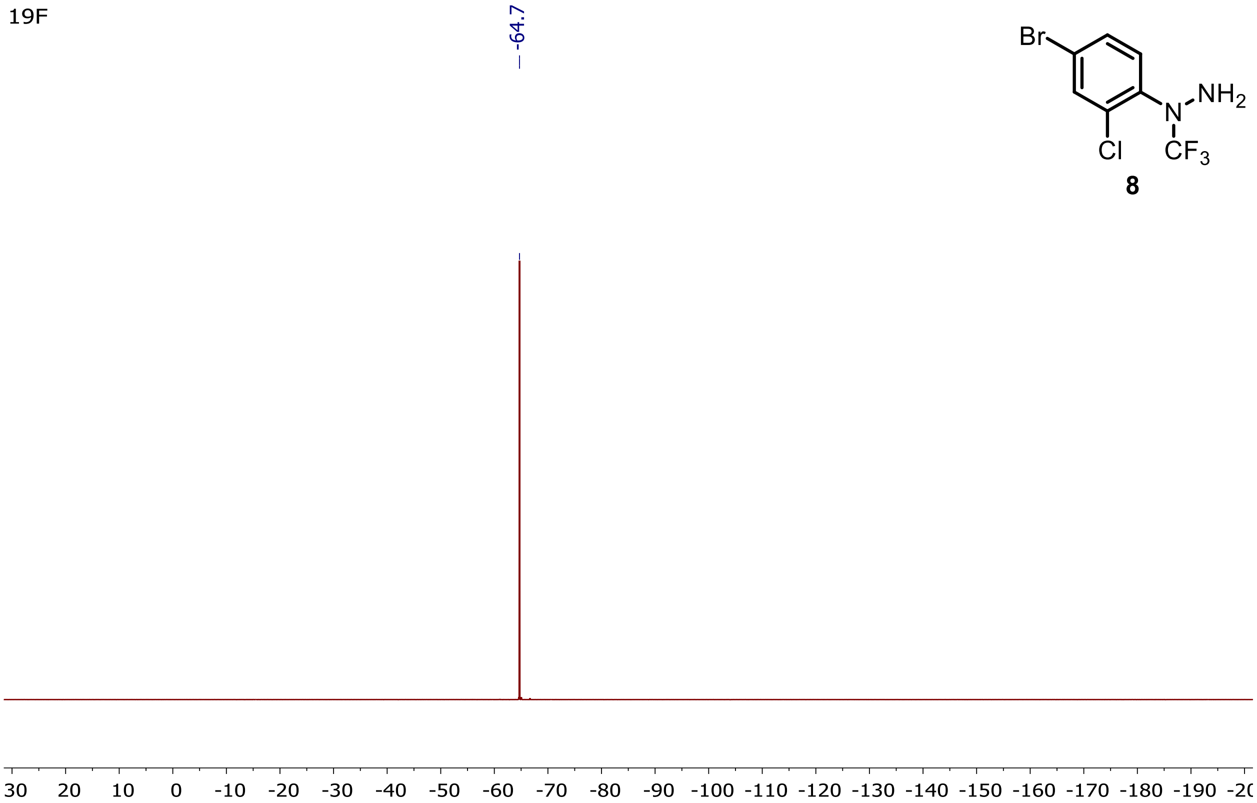
19F
-64.7
Br
NH2
N
Cl
CF3
8
30 20 10 0 -10 -20 -30 -40 -50 -60 -70 -80 -90 -100 -110 -120 -130 -140 -150 -160 -170 -180 -190

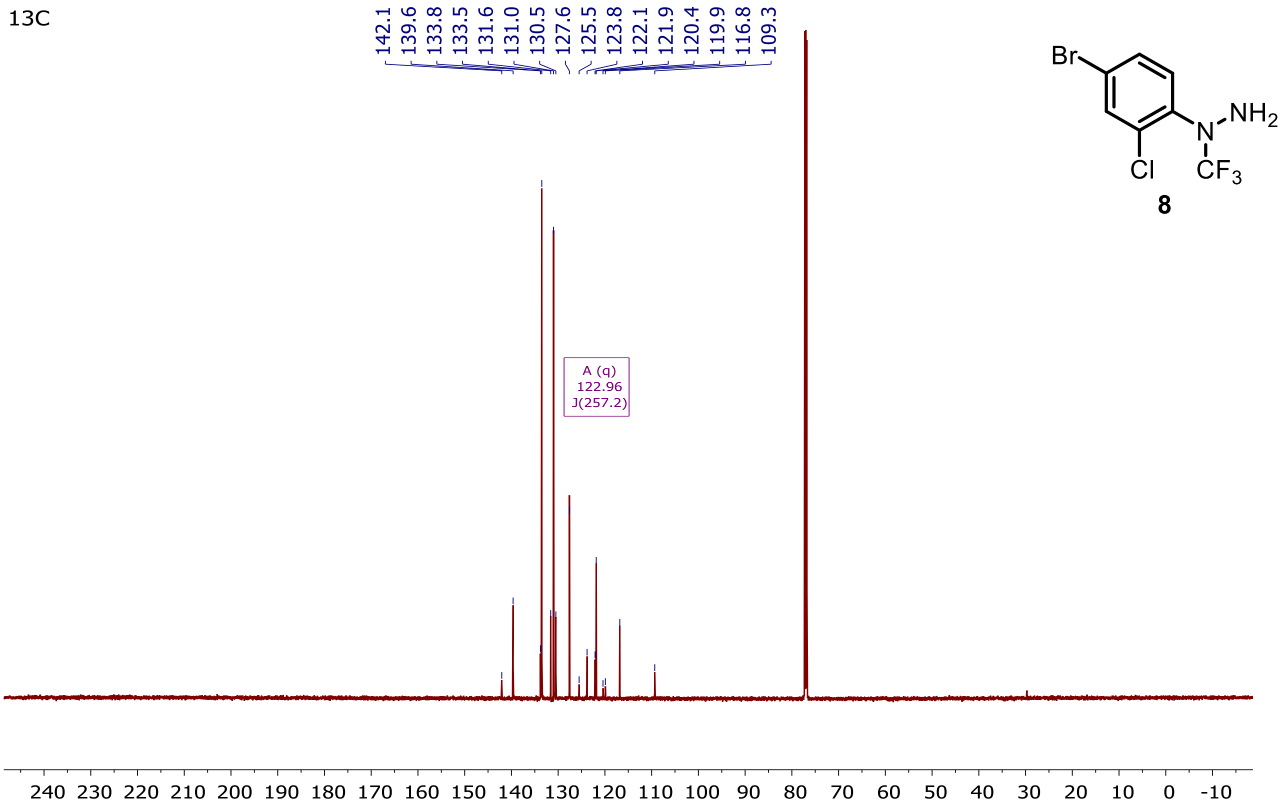
13C
142.1
139.6
133.8
133.5
131.6
131.0
130.5
127.6
125.5
123.8
122.1
121.9
120.4
119.9
116.8
109.3
A (q)
122.96
J(257.2)
Br
NH2
N
Cl
CF3
8
240 230 220 210 200 190 180 170 160 150 140 130 120 110 100 90 80 70 60 50 40 30 20 10 0 -10

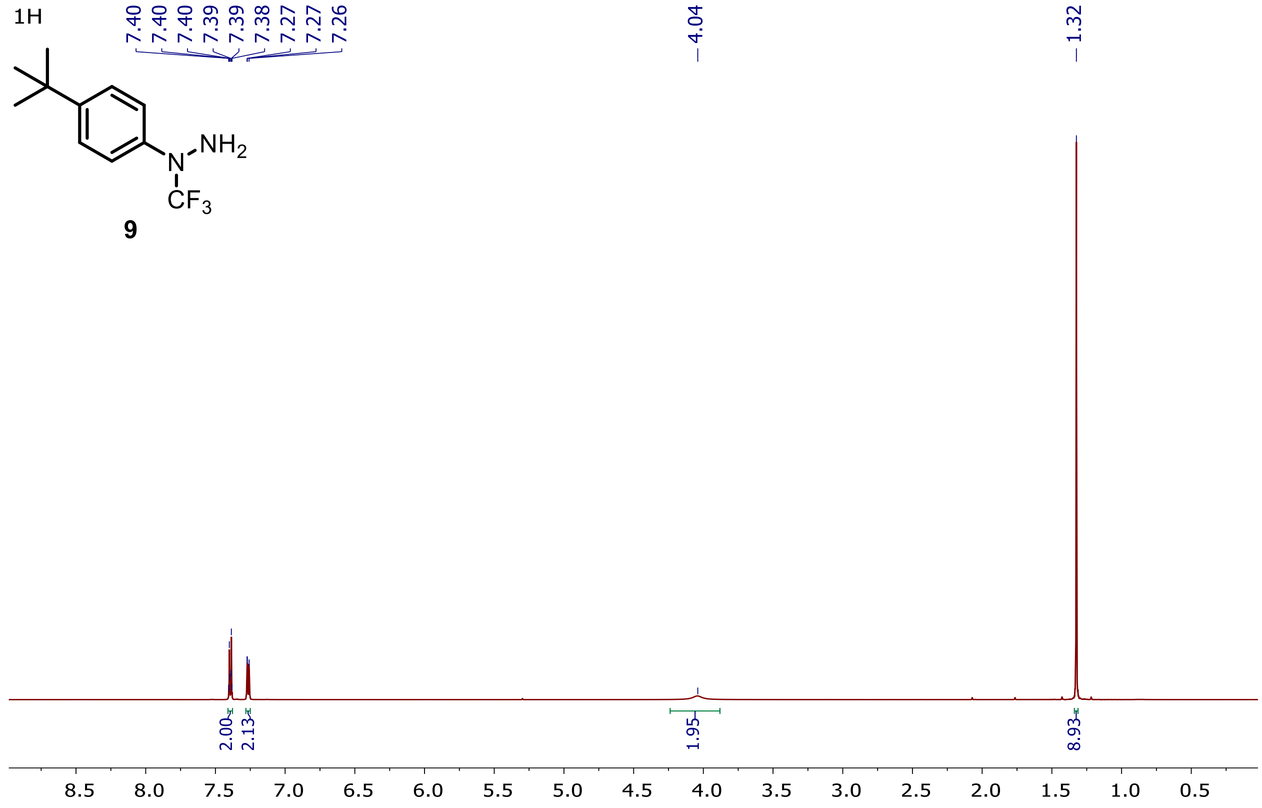
1H
7.40
7.40
7.40
7.39
7.39
7.38
7.27
7.27
7.26
4.04
1.32
NH2
N
CF3
9
2.00
2.13
1.95
8.93
8.5
8.0
7.5
7.0
6.5
6.0
5.5
5.0
4.5
4.0
3.5
3.0
2.5
2.0
1.5
1.0
0.5

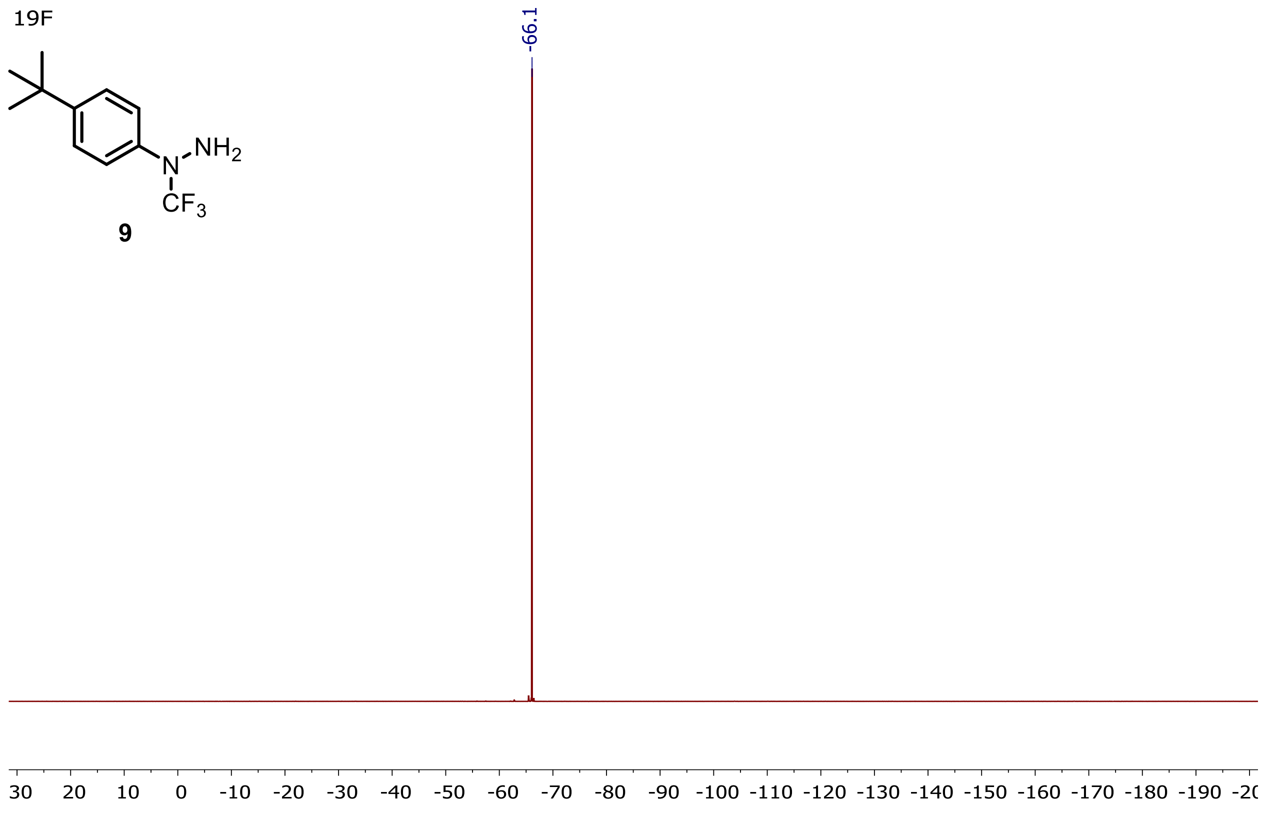
19F
-66.1
NH2
N
CF3
9
30 20 10 0 -10 -20 -30 -40 -50 -60 -70 -80 -90 -100 -110 -120 -130 -140 -150 -160 -170 -180 -190

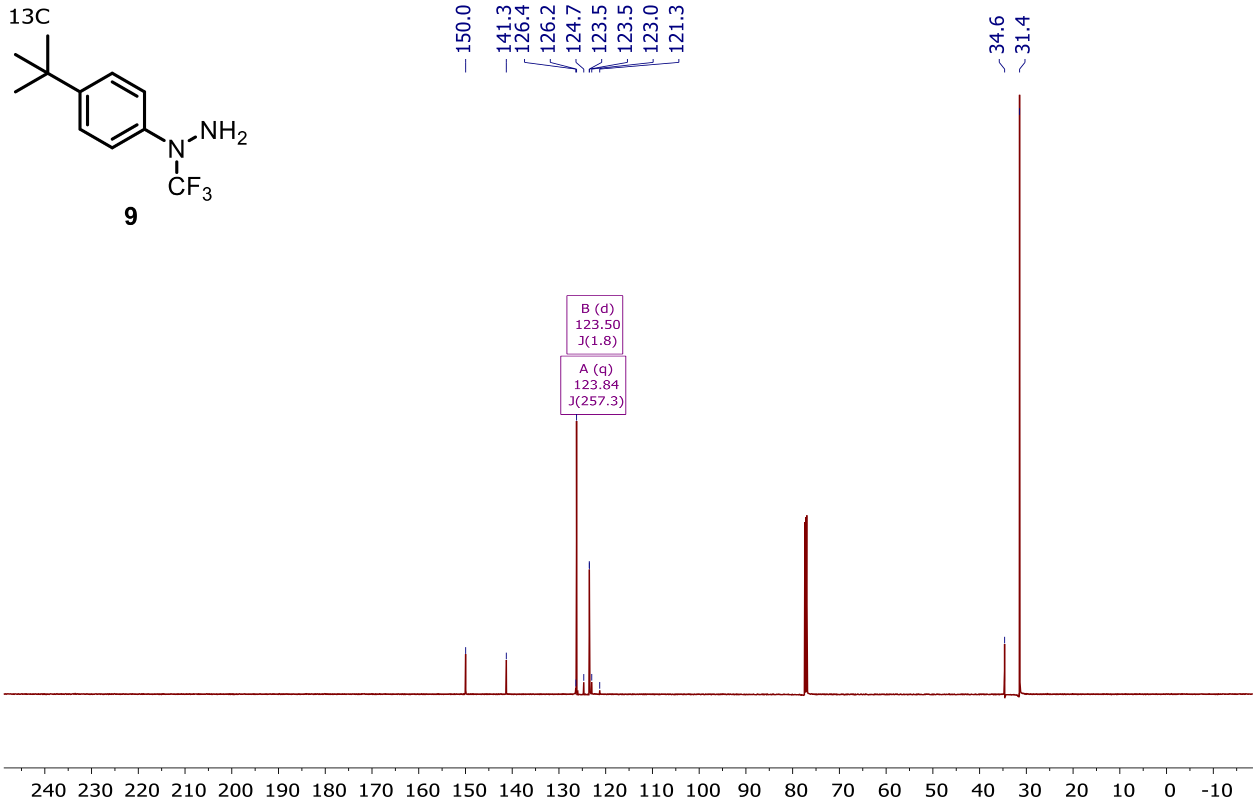
13C
NH2
CF3
9
150.0
141.3
126.4
126.2
124.7
123.5
123.5
123.0
121.3
34.6
31.4
B (d)
123.50
J(1.8)
A (q)
123.84
J(257.3)
240 230 220 210 200 190 180 170 160 150 140 130 120 110 100 90 80 70 60 50 40 30 20 10 0 -10

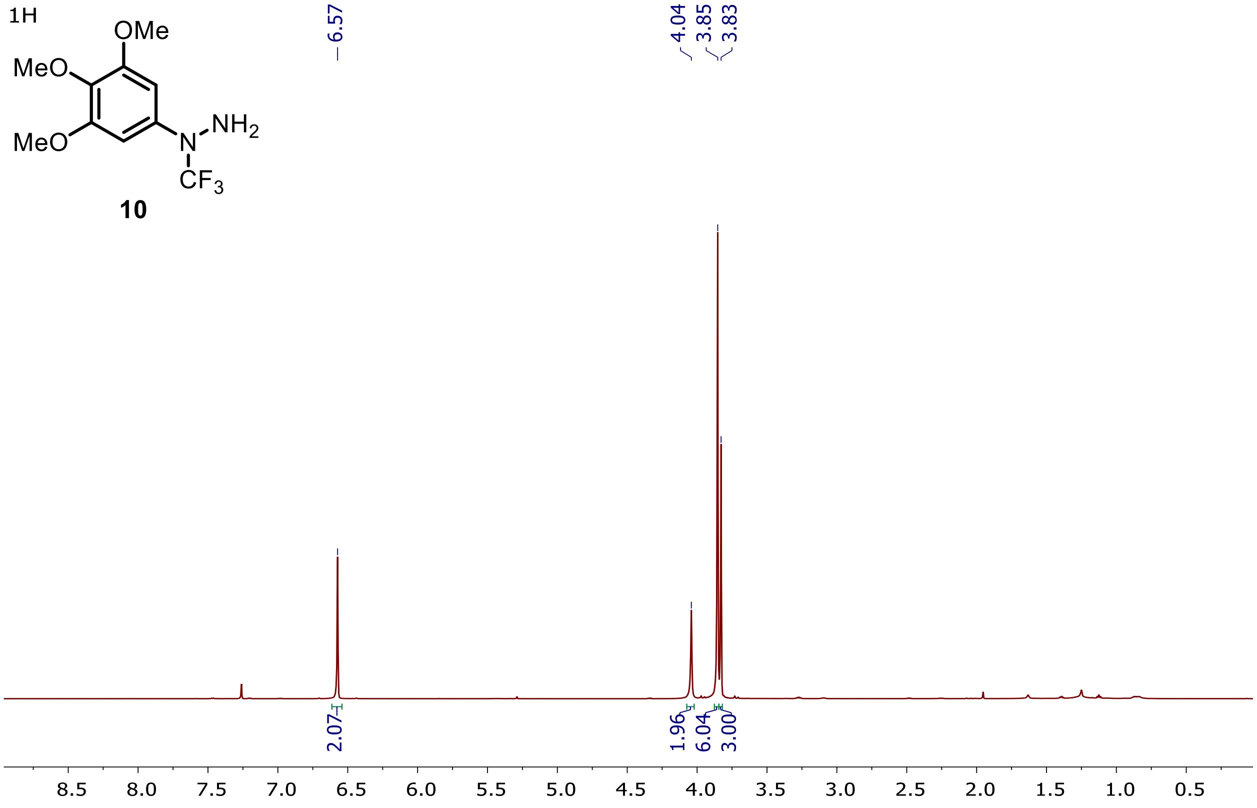
1H
OMe
MeO
MeO
NH2
CF3
10
6.57
4.04
3.85
3.83
2.07
1.96
6.04
3.00
8.5 8.0 7.5 7.0 6.5 6.0 5.5 5.0 4.5 4.0 3.5 3.0 2.5 2.0 1.5 1.0 0.5

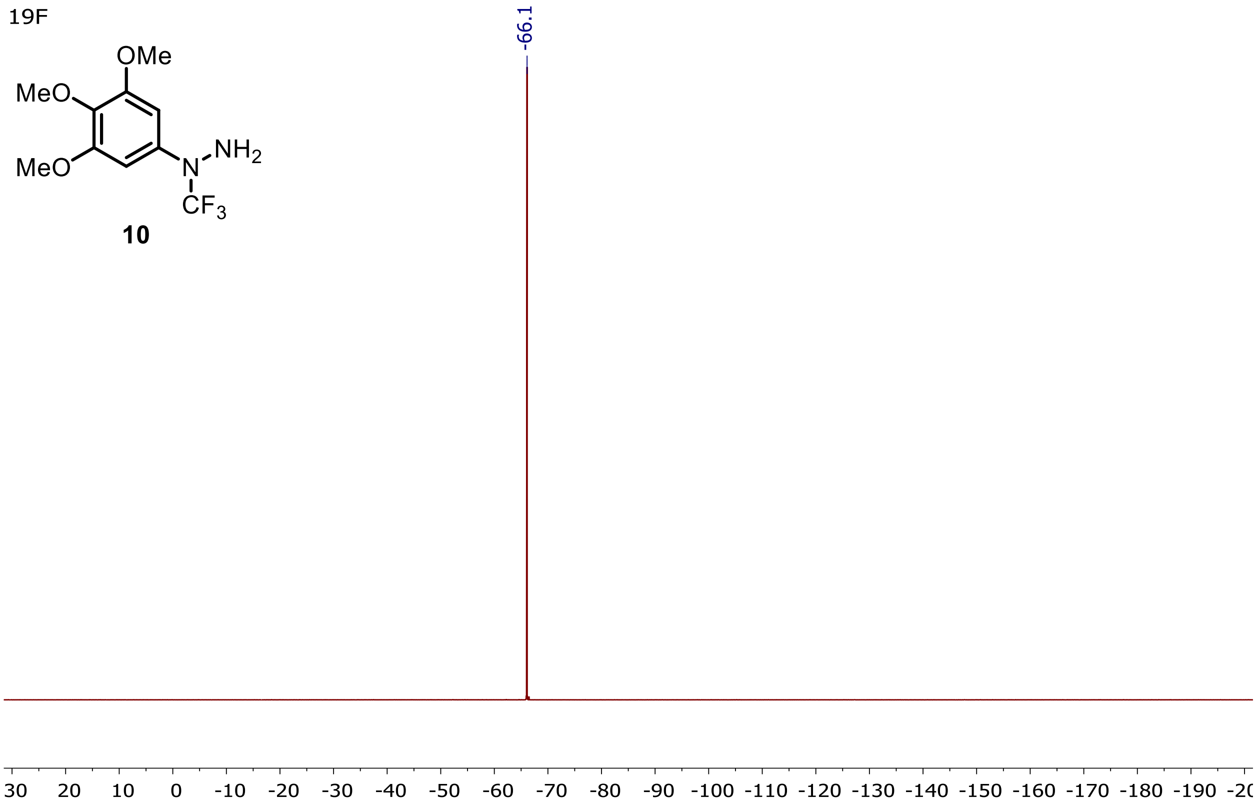
19F
OMe
MeO
MeO
NH2
N
CF3
10
-66.1
30 20 10 0 -10 -20 -30 -40 -50 -60 -70 -80 -90 -100 -110 -120 -130 -140 -150 -160 -170 -180 -190

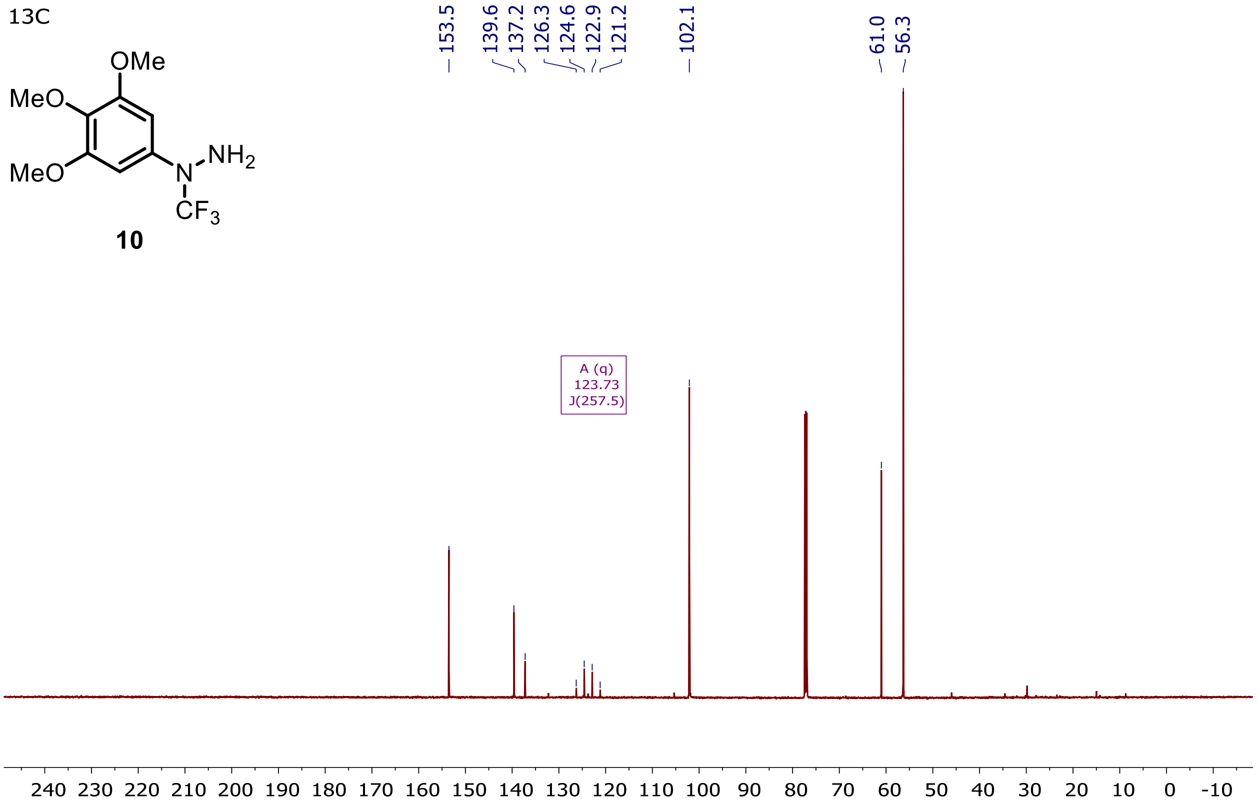
13C
OMe
MeO
MeO
NH2
N
CF3
10
153.5
139.6
137.2
126.3
124.6
122.9
121.2
102.1
61.0
56.3
A (q)
123.73
J(257.5)
240 230 220 210 200 190 180 170 160 150 140 130 120 110 100 90 80 70 60 50 40 30 20 10 0 -10

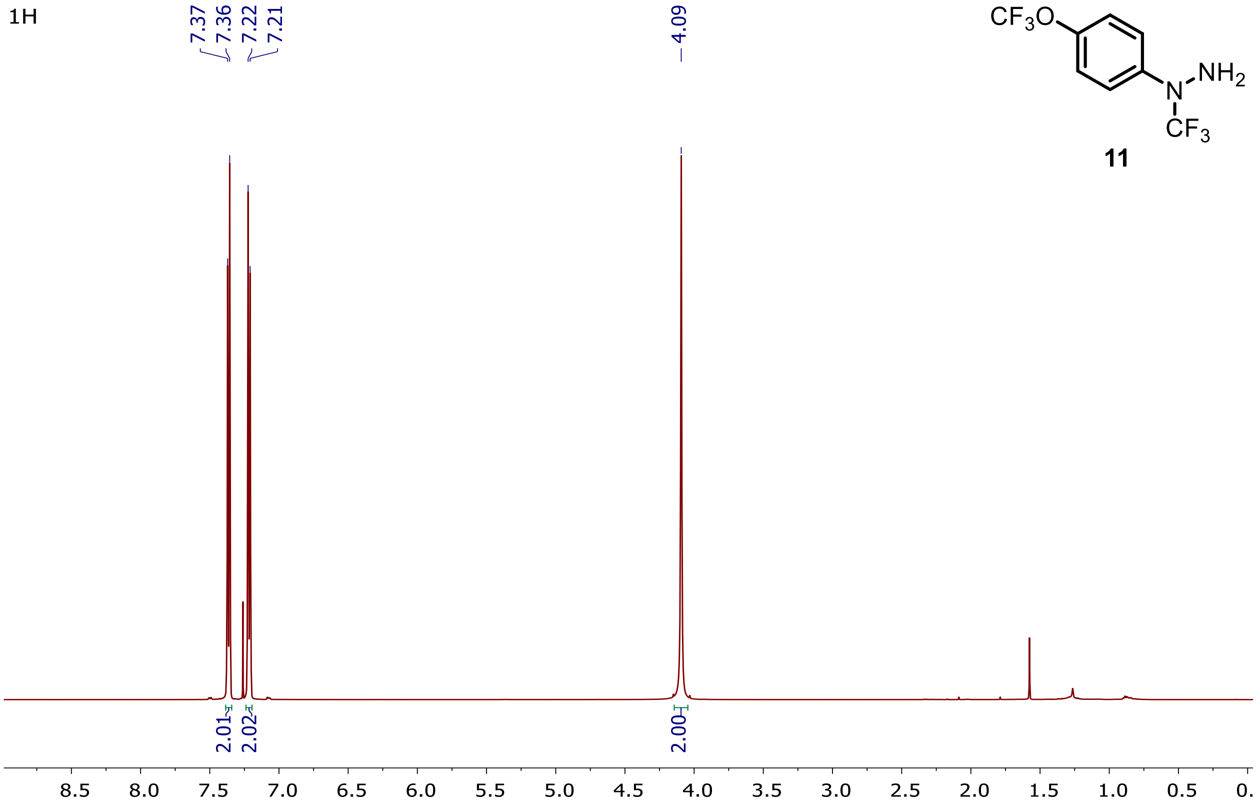
1H
7.37
7.36
7.22
7.21
4.09
CF3O
NH2
N
CF3
2.01
2.02
2.00
8.5 8.0 7.5 7.0 6.5 6.0 5.5 5.0 4.5 4.0 3.5 3.0 2.5 2.0 1.5 1.0 0.5 0.

11

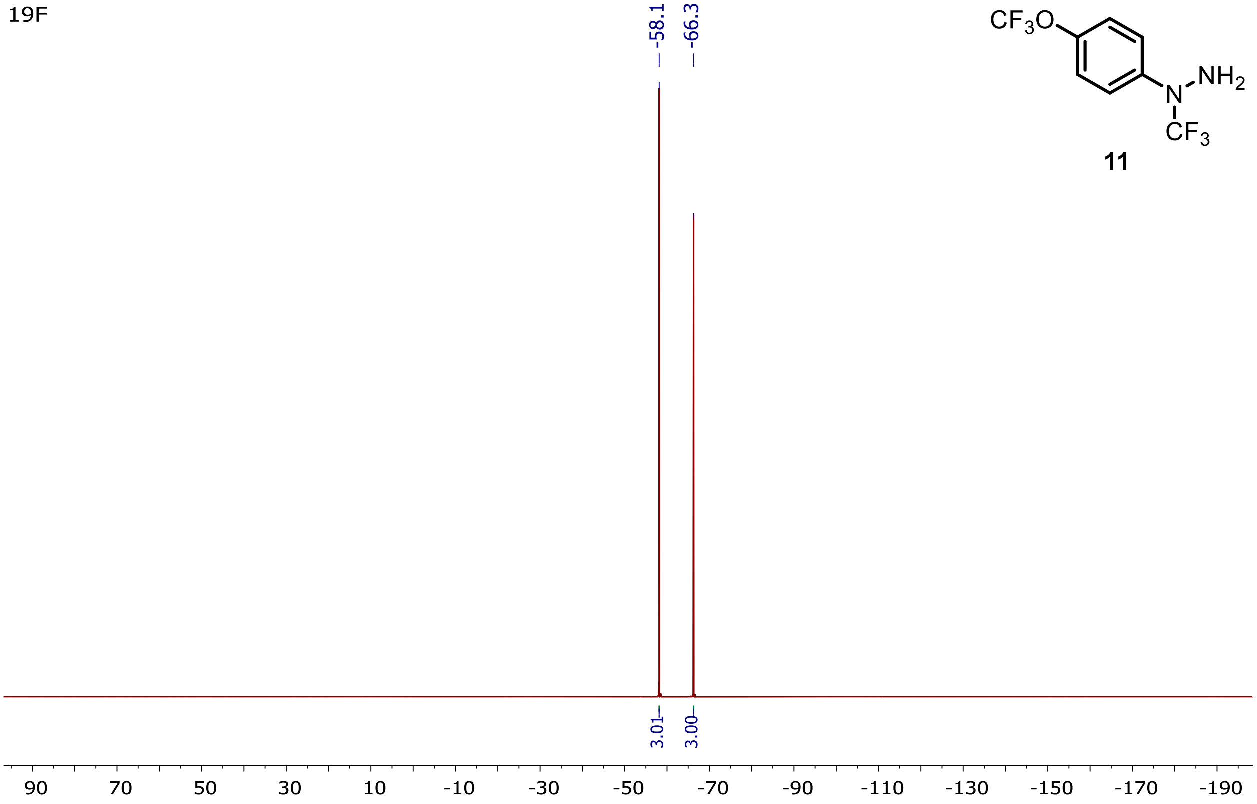
19F
-58.1
-66.3
CF3O
NH2
N
CF3
3.01
3.00
90 70 50 30 10 -10 -30 -50 -70 -90 -110 -130 -150 -170 -190

11

13C

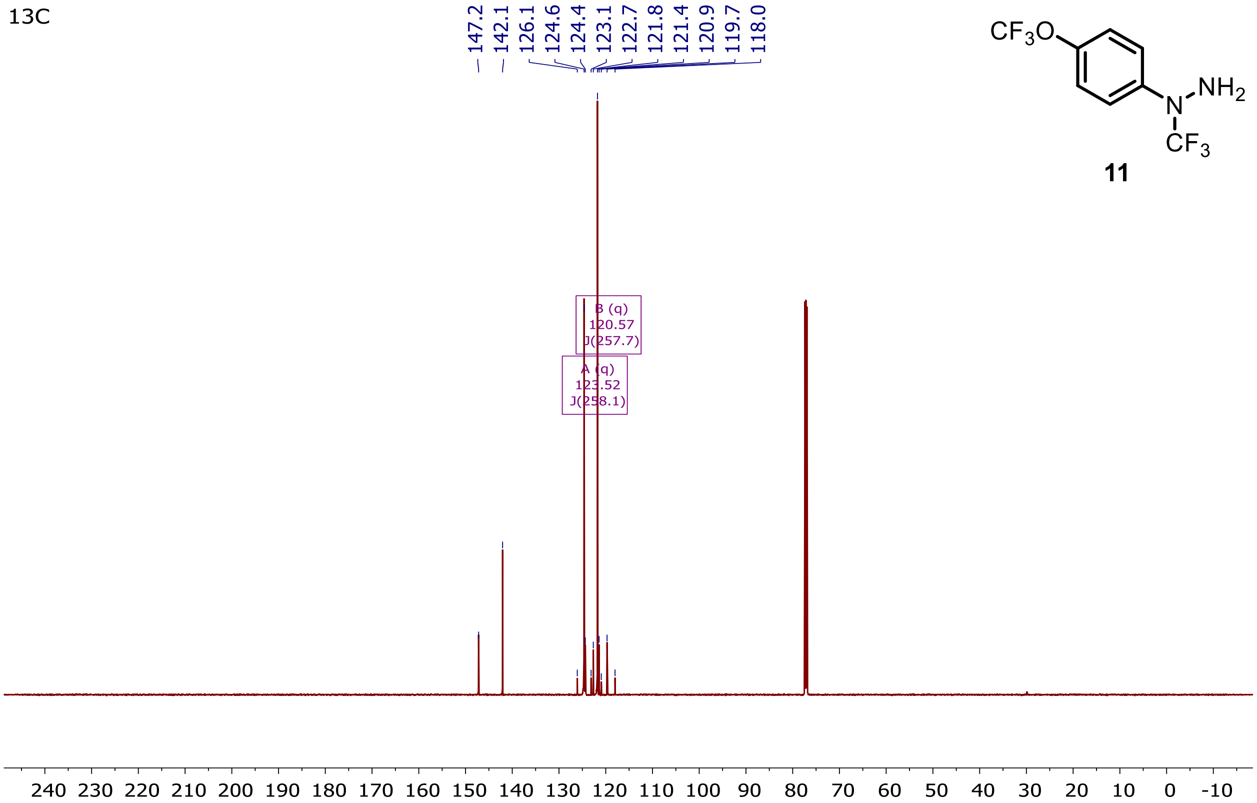
147.2
142.1
126.1
124.6
124.4
123.1
122.7
121.8
121.4
120.9
119.7
118.0
B (q)
120.57
J(257.7)
A (q)
123.52
J(258.1)
240 230 220 210 200 190 180 170 160 150 140 130 120 110 100 90 80 70 60 50 40 30 20 10 0 -10
CF3O
NH2
N
CF3

11

1H

12

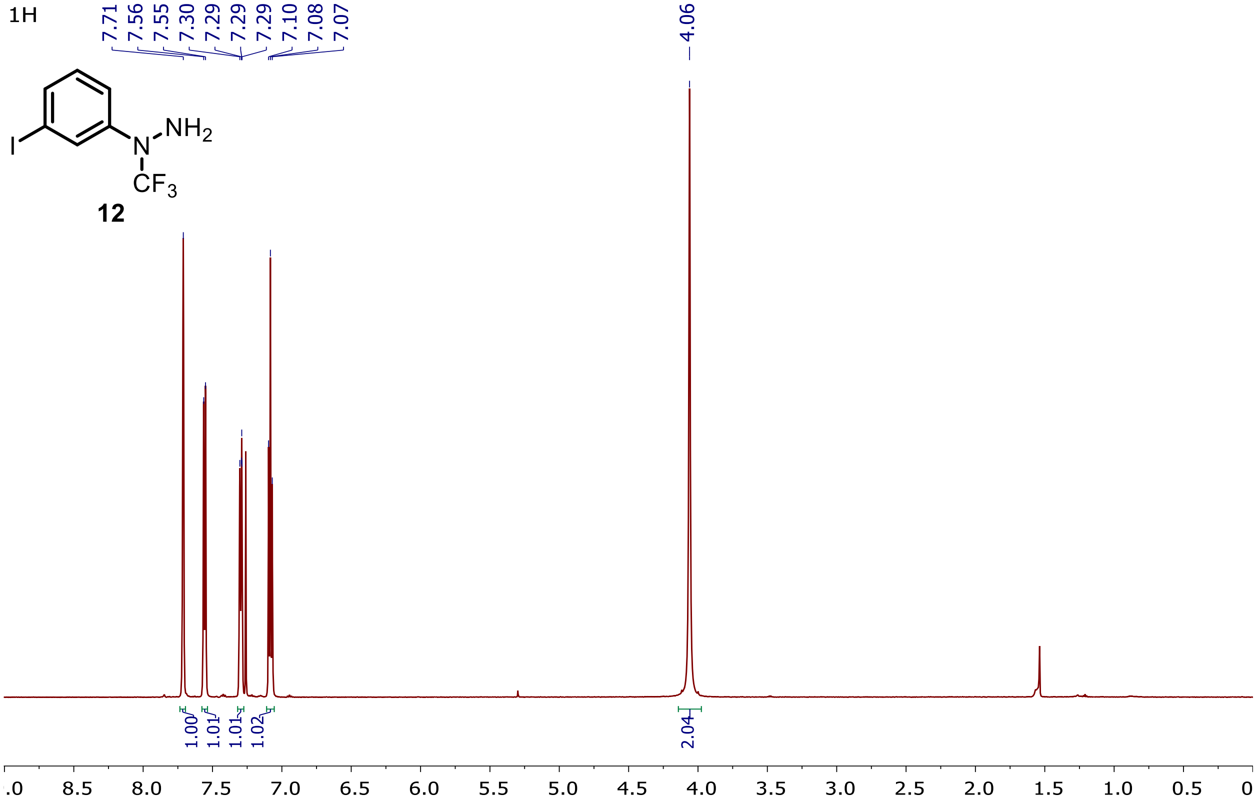
I
NH2
N
CF3
7.71
7.56
7.55
7.30
7.29
7.29
7.29
7.10
7.08
7.07
4.06
1.00
1.01
1.01
1.02
2.04
.0 8.5 8.0 7.5 7.0 6.5 6.0 5.5 5.0 4.5 4.0 3.5 3.0 2.5 2.0 1.5 1.0 0.5 0

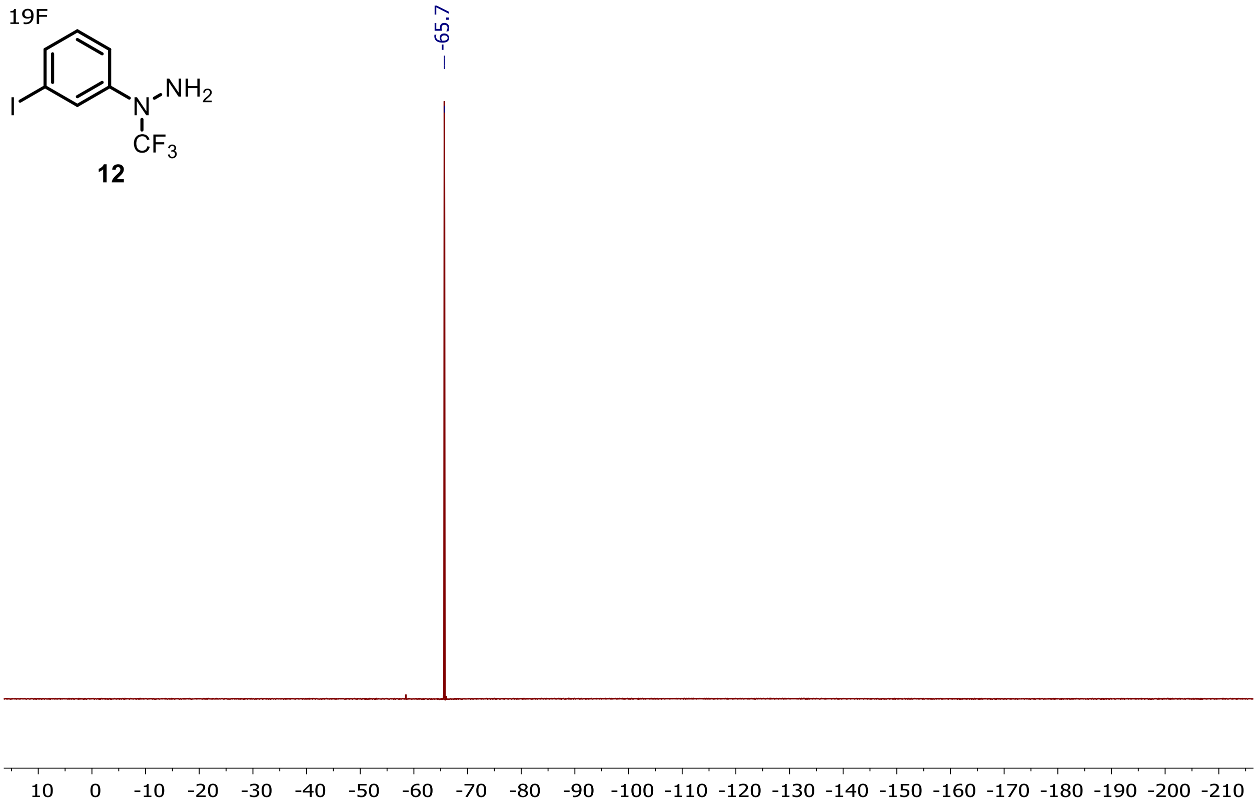

19F
I
NH2
N
CF3
12
-65.7
10 0 -10 -20 -30 -40 -50 -60 -70 -80 -90 -100 -110 -120 -130 -140 -150 -160 -170 -180 -190 -200 -210

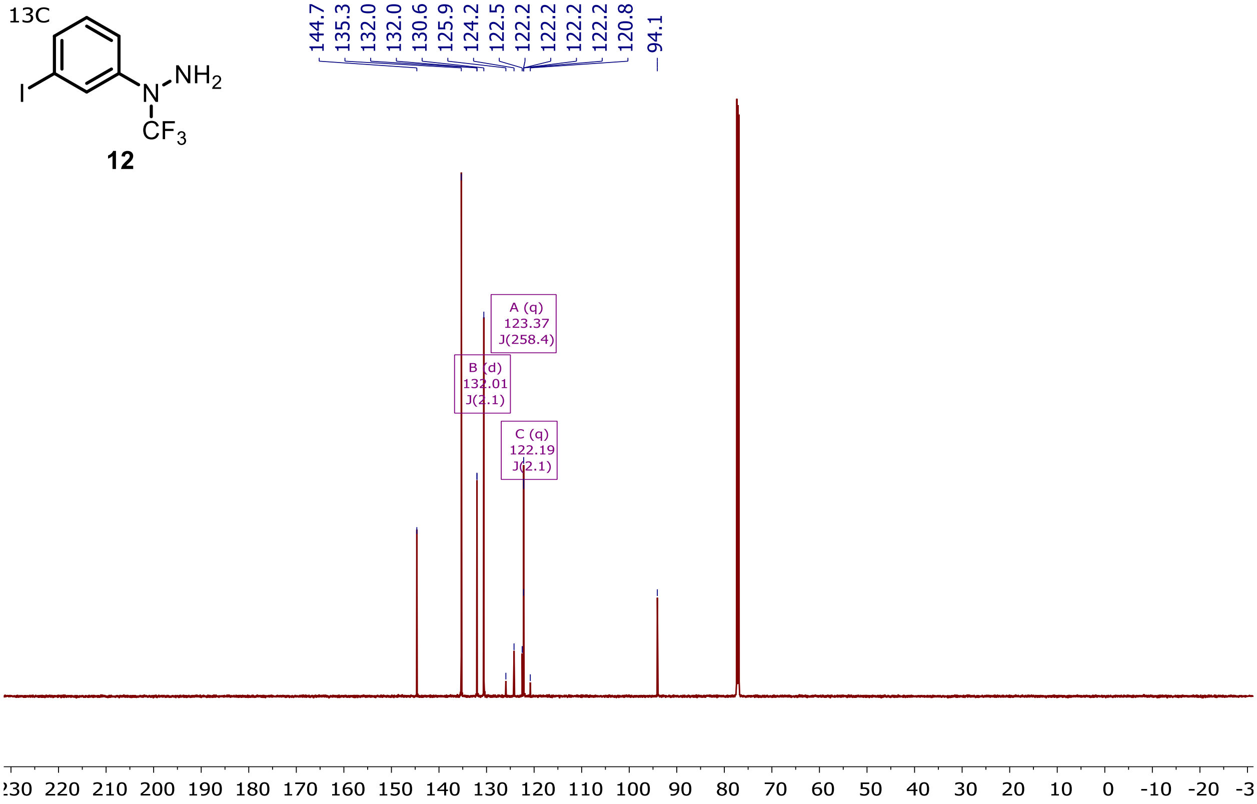

13C
I
NH2
N
CF3
12
144.7
135.3
132.0
132.0
130.6
125.9
124.2
122.5
122.2
122.2
122.2
122.2
120.8
94.1
A (q)
123.37
J(258.4)
B (d)
132.01
J(2.1)
C (q)
122.19
J(2.1)
230 220 210 200 190 180 170 160 150 140 130 120 110 100 90 80 70 60 50 40 30 20 10 0 -10 -20 -3

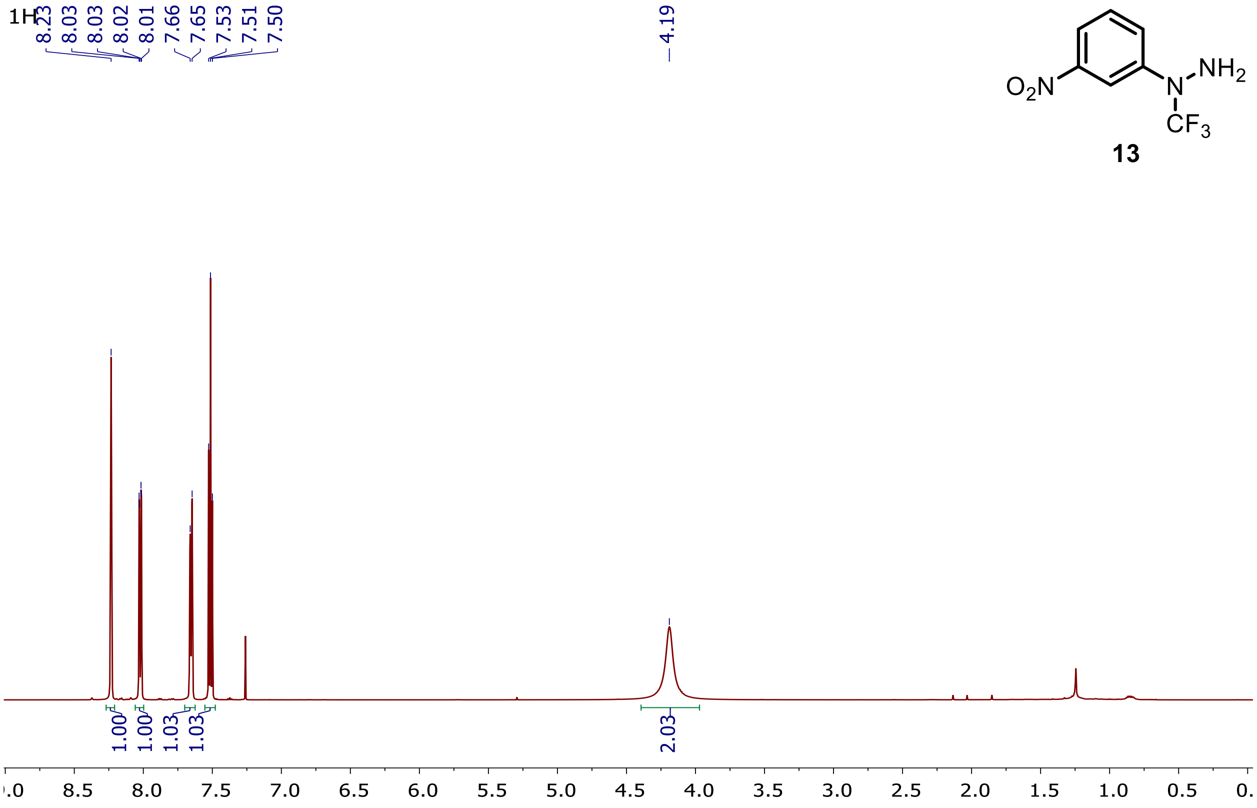
1H
8.23
8.03
8.03
8.02
8.01
7.66
7.65
7.53
7.51
7.50
4.19
O2N
NH2
N
CF3
13
1.00
1.00
1.03
1.03
2.03
8.5
8.0
7.5
7.0
6.5
6.0
5.5
5.0
4.5
4.0
3.5
3.0
2.5
2.0
1.5
1.0
0.5

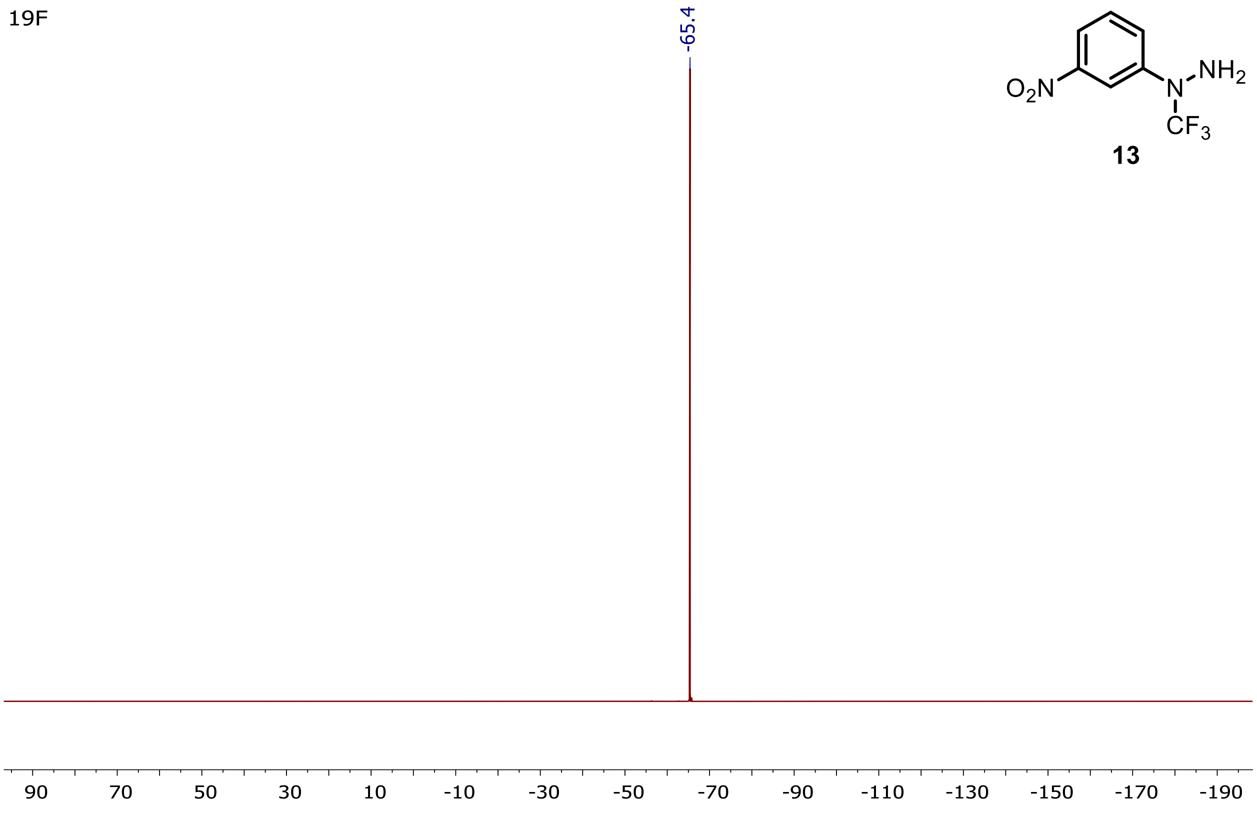
19F
-65.4
O2N
NH2
N
CF3
13
90
70
50
30
10
-10
-30
-50
-70
-90
-110
-130
-150
-170
-190

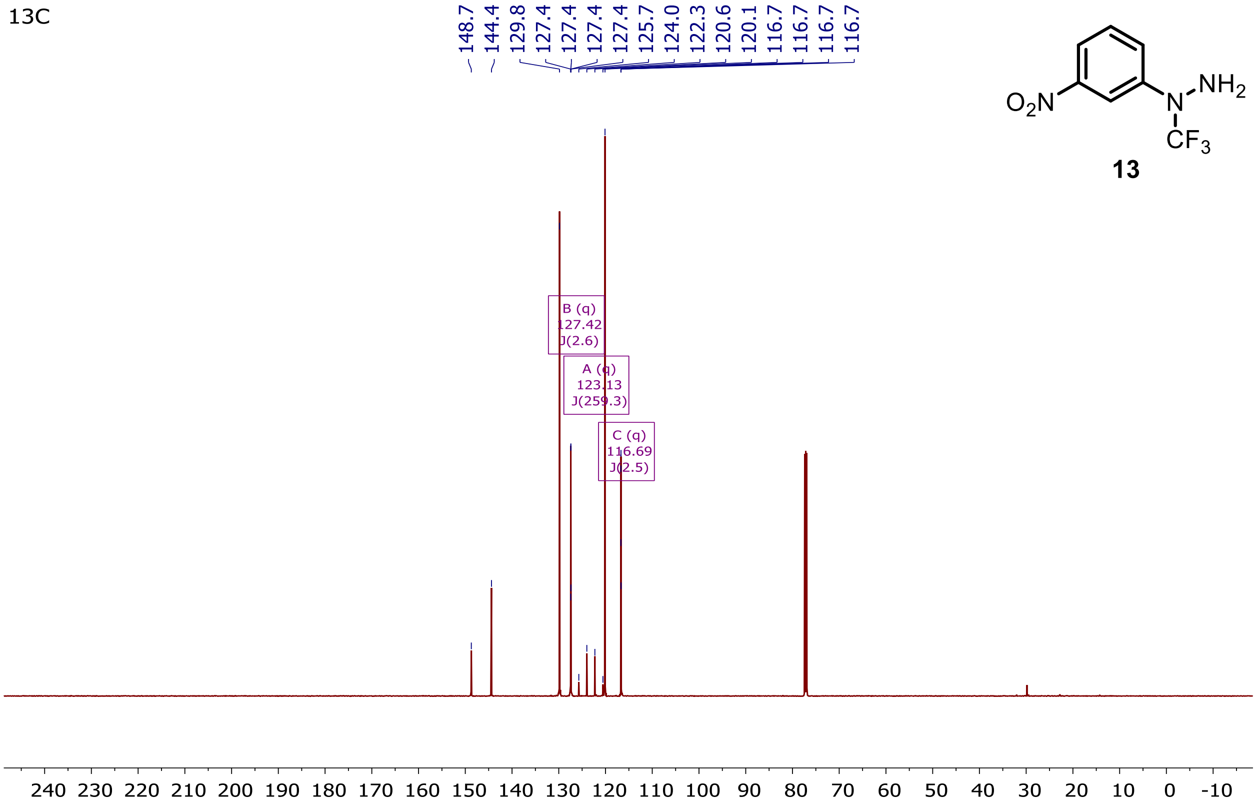
13C
148.7
144.4
129.8
127.4
127.4
127.4
127.4
125.7
124.0
122.3
120.6
120.1
116.7
116.7
116.7
116.7
B (q)
127.42
J(2.6)
A (q)
123.13
J(259.3)
C (q)
116.69
J(2.5)
240 230 220 210 200 190 180 170 160 150 140 130 120 110 100 90 80 70 60 50 40 30 20 10 0 -10
O_2N
NH_2
N
CF_3

13

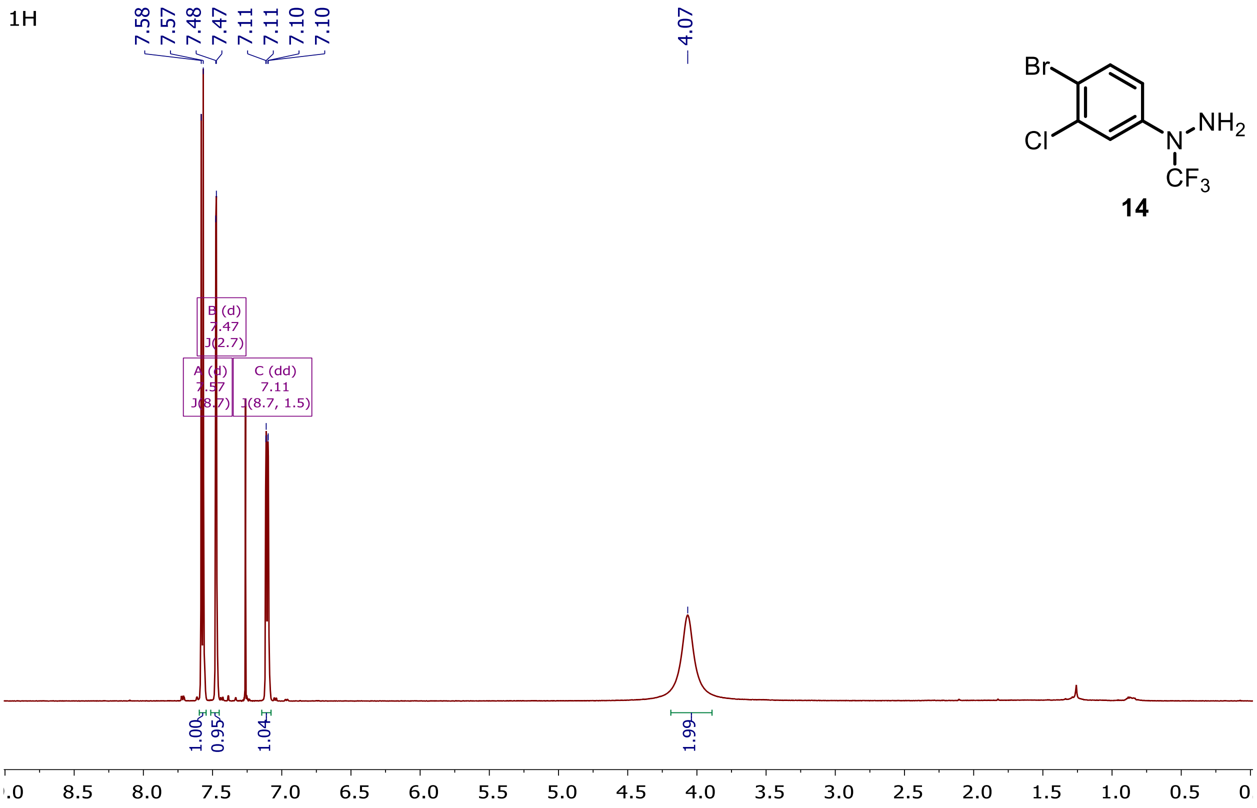
1H
7.58
7.57
7.48
7.47
7.11
7.11
7.10
7.10
4.07
B (d)
7.47
J(2.7)
A (d)
7.57
J(8.7)
C (dd)
7.11
J(8.7, 1.5)
1.00
0.95
1.04
1.99
8.5 8.0 7.5 7.0 6.5 6.0 5.5 5.0 4.5 4.0 3.5 3.0 2.5 2.0 1.5 1.0 0.5 0
Br
Cl
NH_2
N
CF_3

14

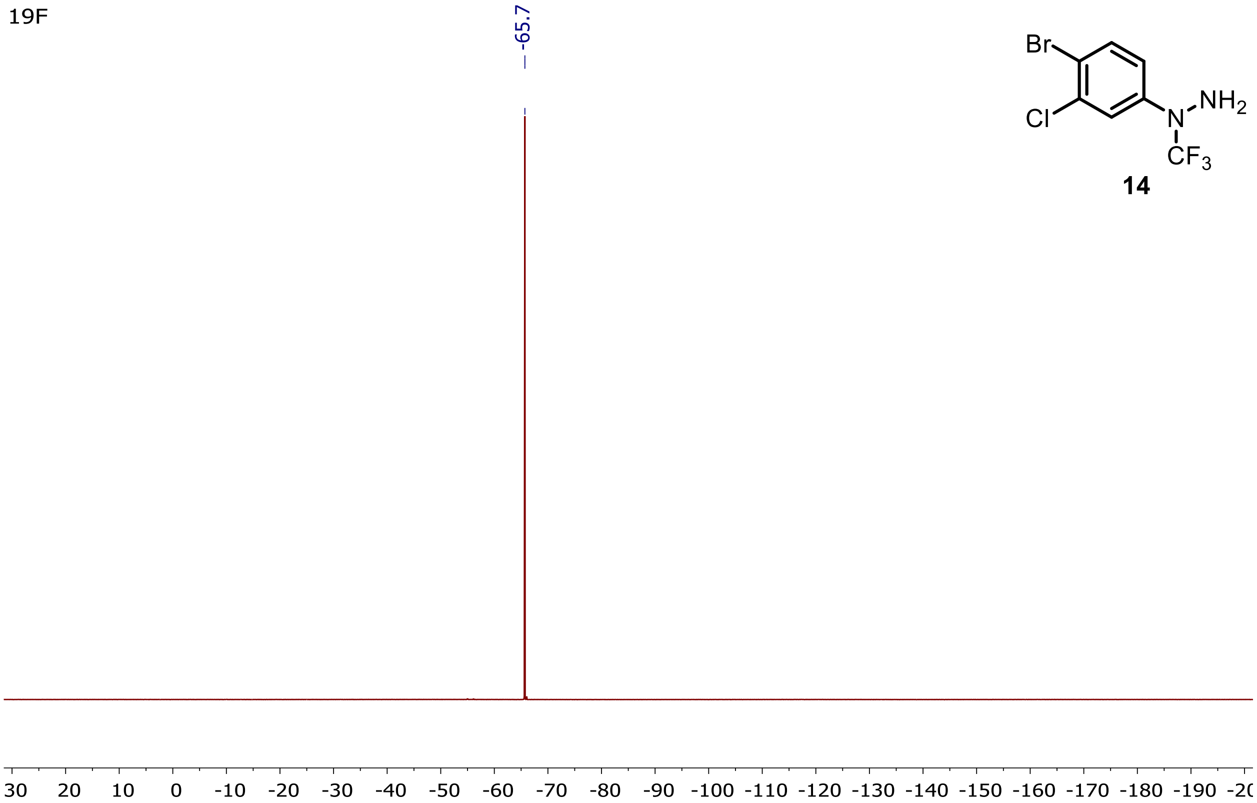
19F
-65.7
Br
Cl
NH2
N
CF3
14
30 20 10 0 -10 -20 -30 -40 -50 -60 -70 -80 -90 -100 -110 -120 -130 -140 -150 -160 -170 -180 -190

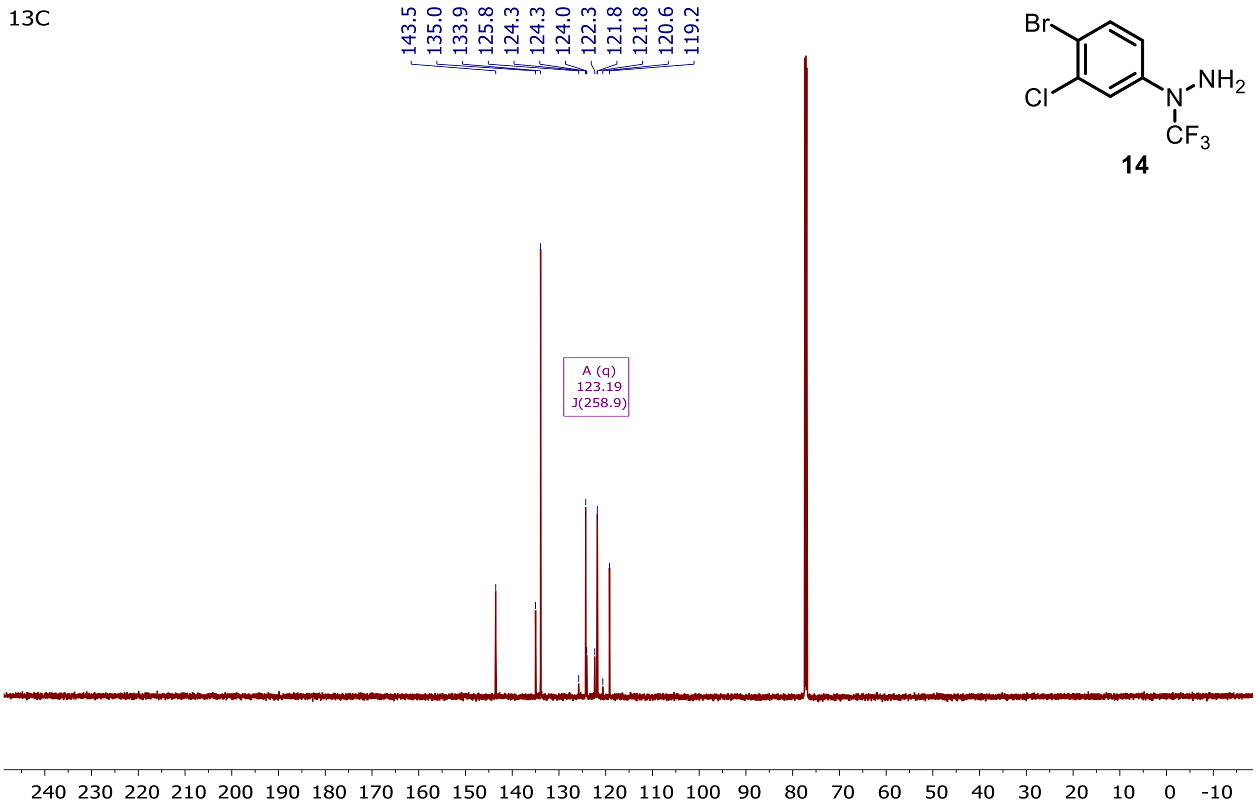
13C
143.5
135.0
133.9
125.8
124.3
124.3
124.0
122.3
121.8
121.8
120.6
119.2
A (q)
123.19
J(258.9)
Br
Cl
NH2
N
CF3
14
240 230 220 210 200 190 180 170 160 150 140 130 120 110 100 90 80 70 60 50 40 30 20 10 0 -10

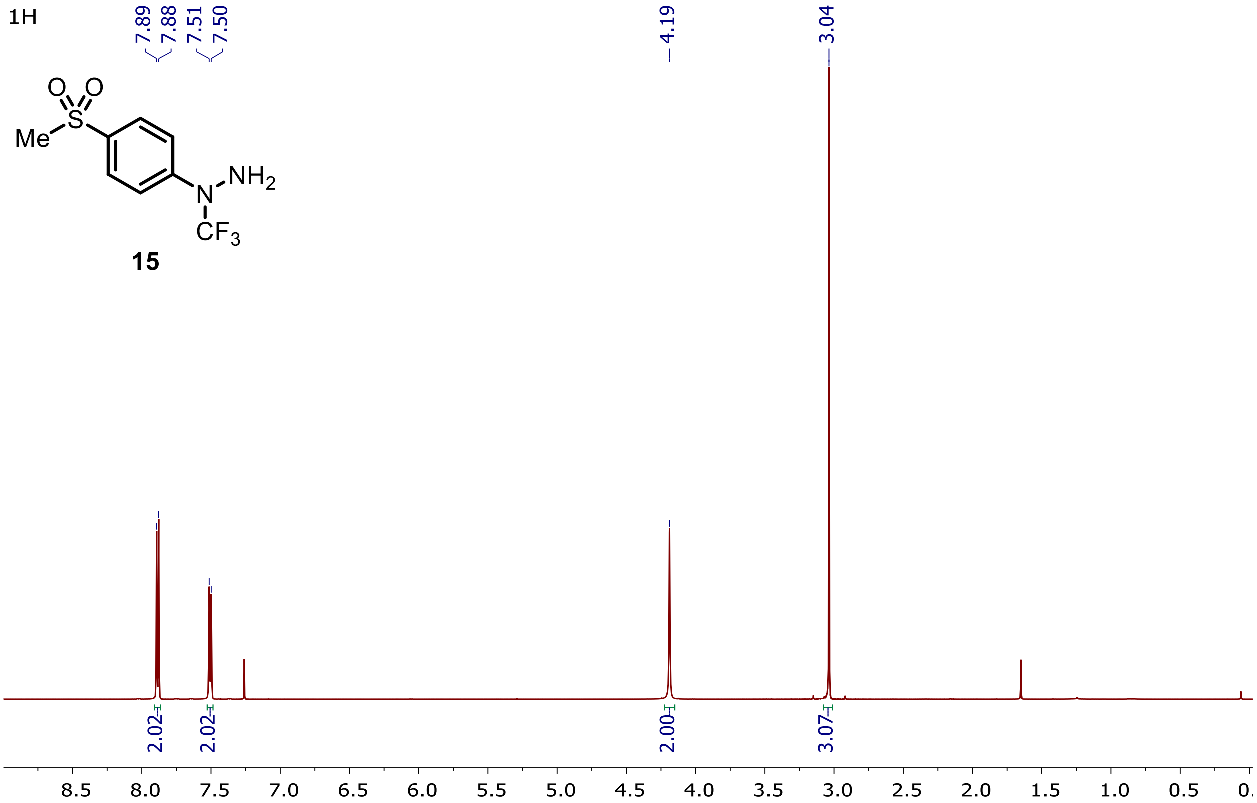

1H
7.89
7.88
7.51
7.50
4.19
3.04
O
O
S
Me
NH2
N
CF3
15
2.02
2.02
2.00
3.07
8.5 8.0 7.5 7.0 6.5 6.0 5.5 5.0 4.5 4.0 3.5 3.0 2.5 2.0 1.5 1.0 0.5

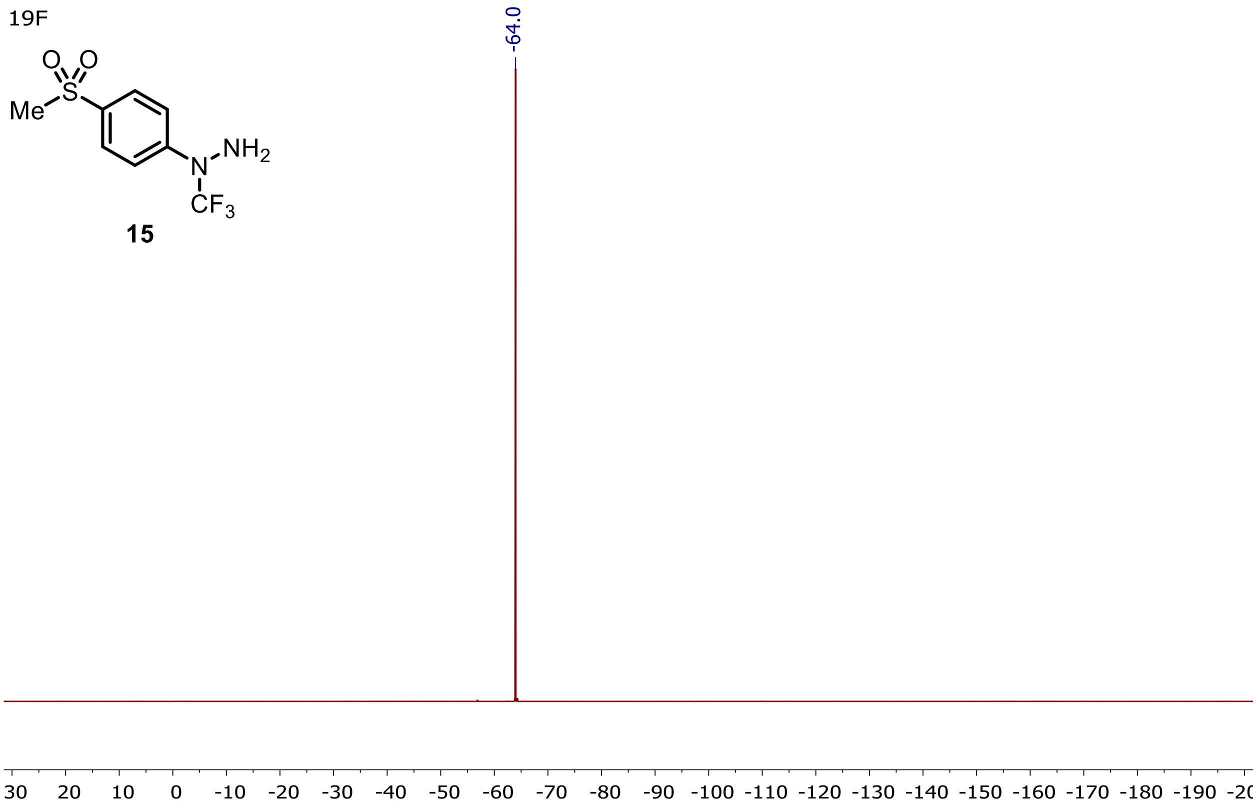

19F
-64.0
O
O
S
Me
NH2
N
CF3
15
30 20 10 0 -10 -20 -30 -40 -50 -60 -70 -80 -90 -100 -110 -120 -130 -140 -150 -160 -170 -180 -190

13C

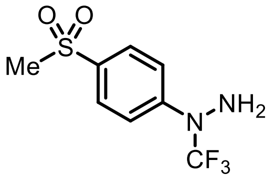

15

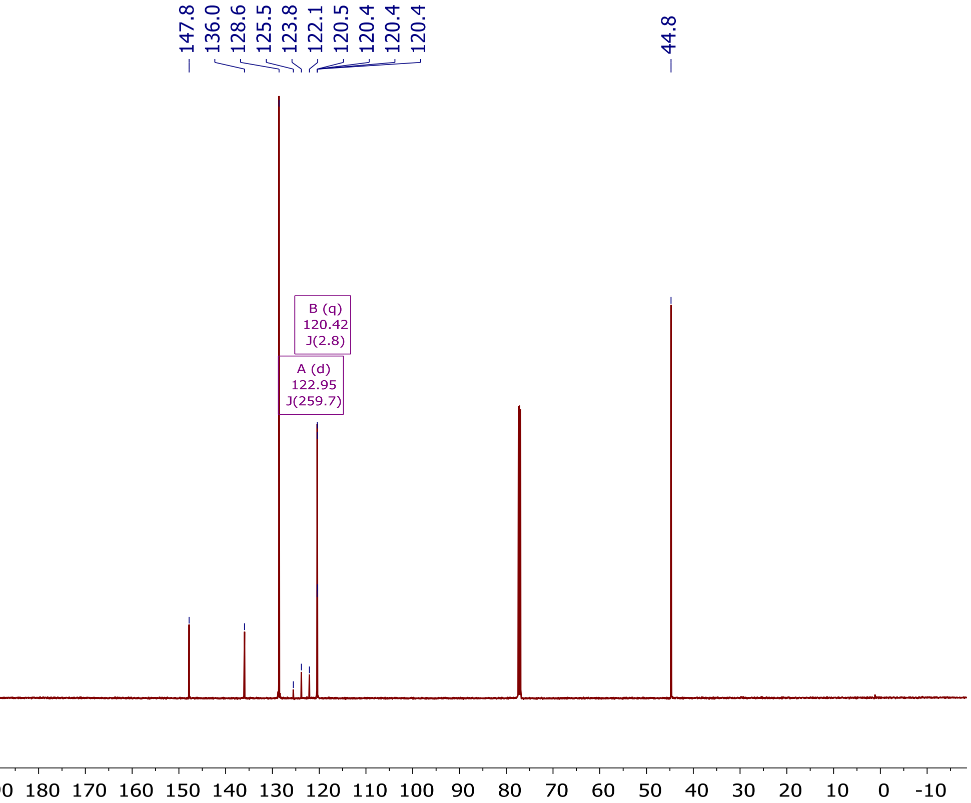

147.8
136.0
128.6
125.5
123.8
122.1
120.5
120.4
120.4
120.4
44.8
B (q)
120.42
J(2.8)
A (d)
122.95
J(259.7)
240 230 220 210 200 190 180 170 160 150 140 130 120 110 100 90 80 70 60 50 40 30 20 10 0 -10

Derivatized hydrazines

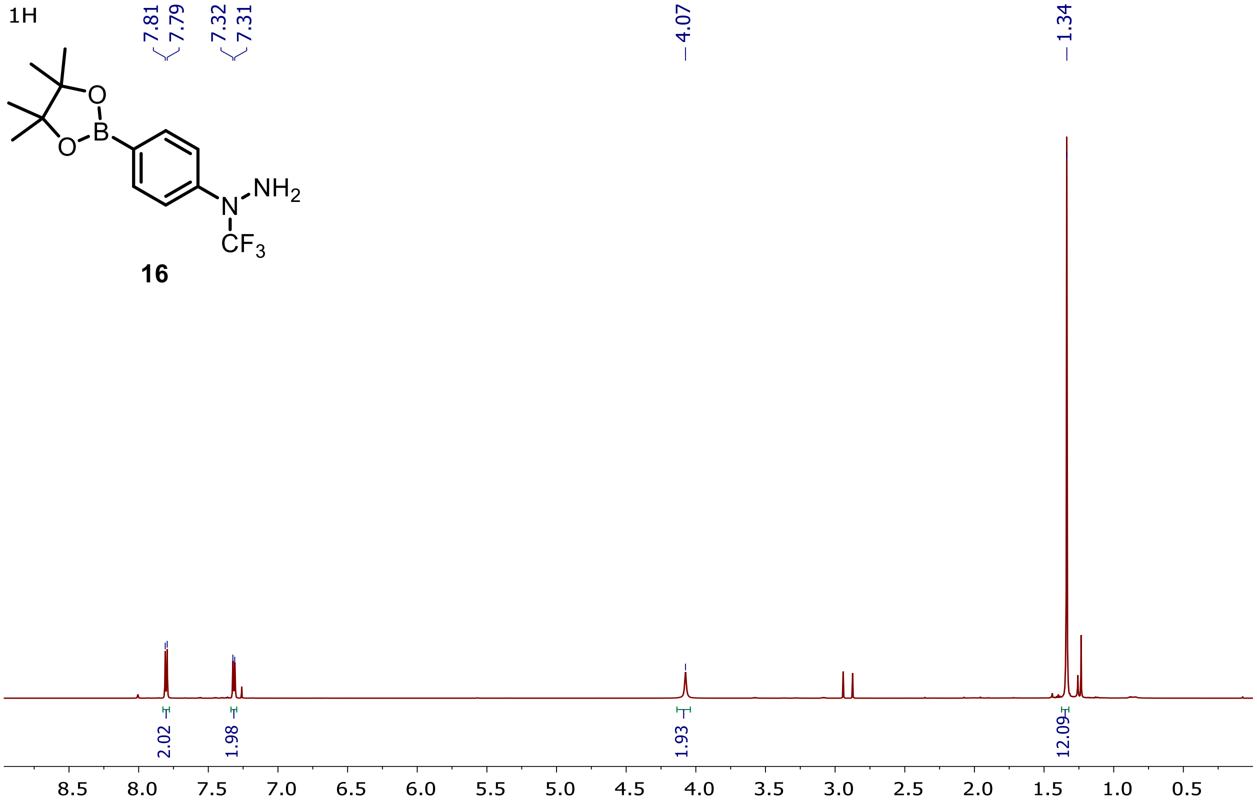

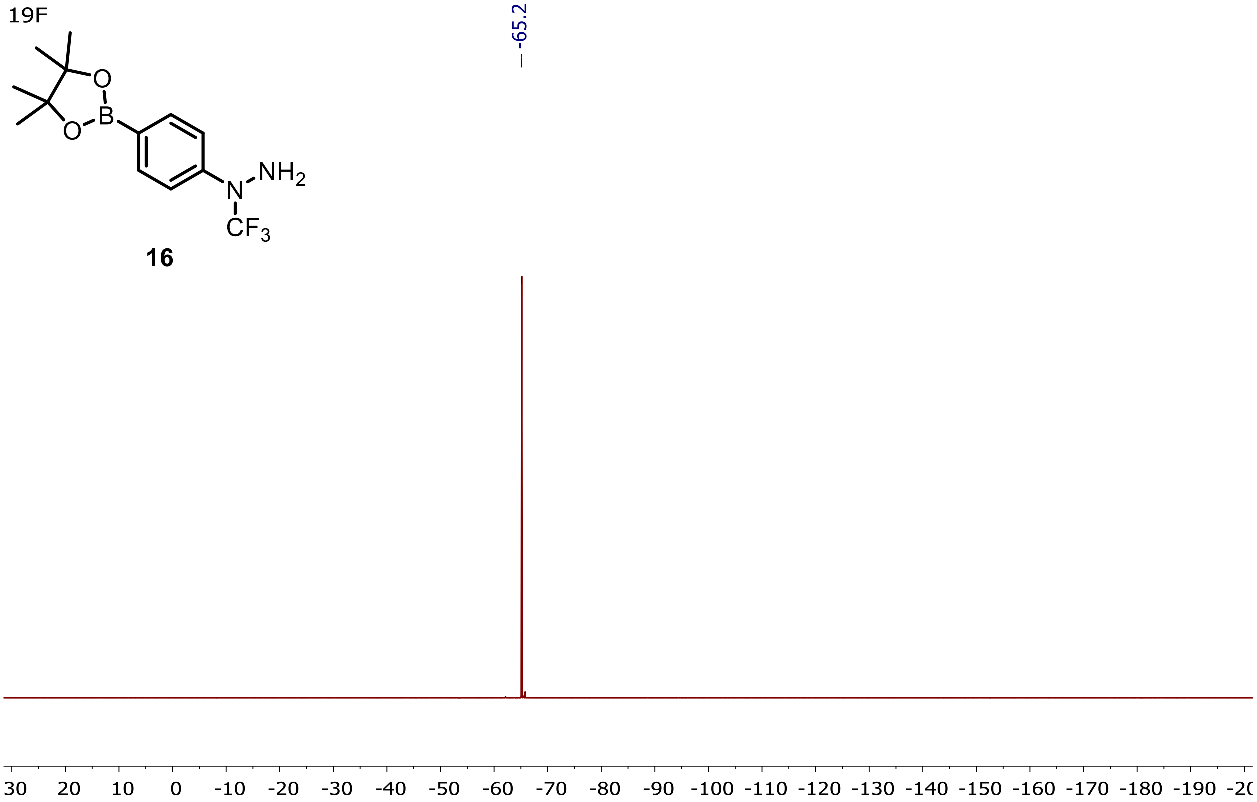

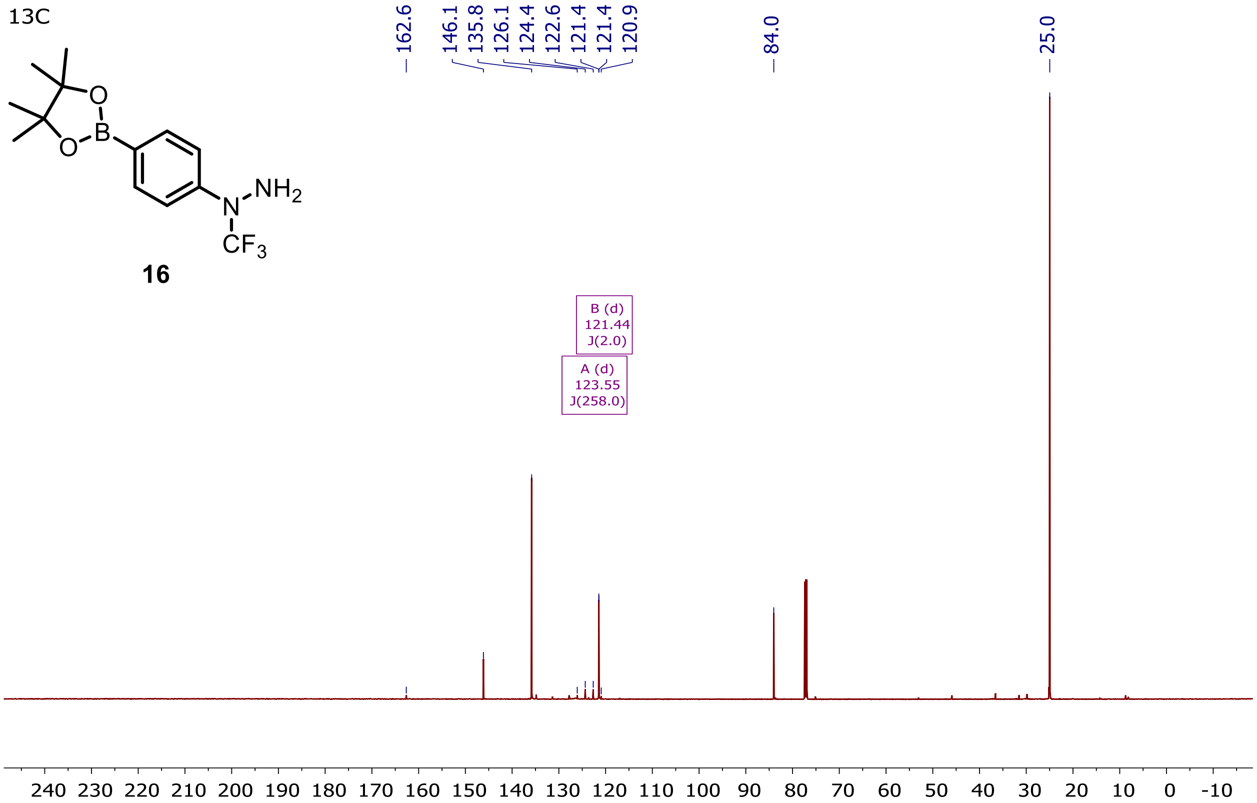
13C
162.6
146.1
135.8
126.1
124.4
122.6
121.4
121.4
120.9
84.0
25.0
NH2
CF3
O
B
N
16
B (d)
121.44
J(2.0)
A (d)
123.55
J(258.0)
240 230 220 210 200 190 180 170 160 150 140 130 120 110 100 90 80 70 60 50 40 30 20 10 0 -10

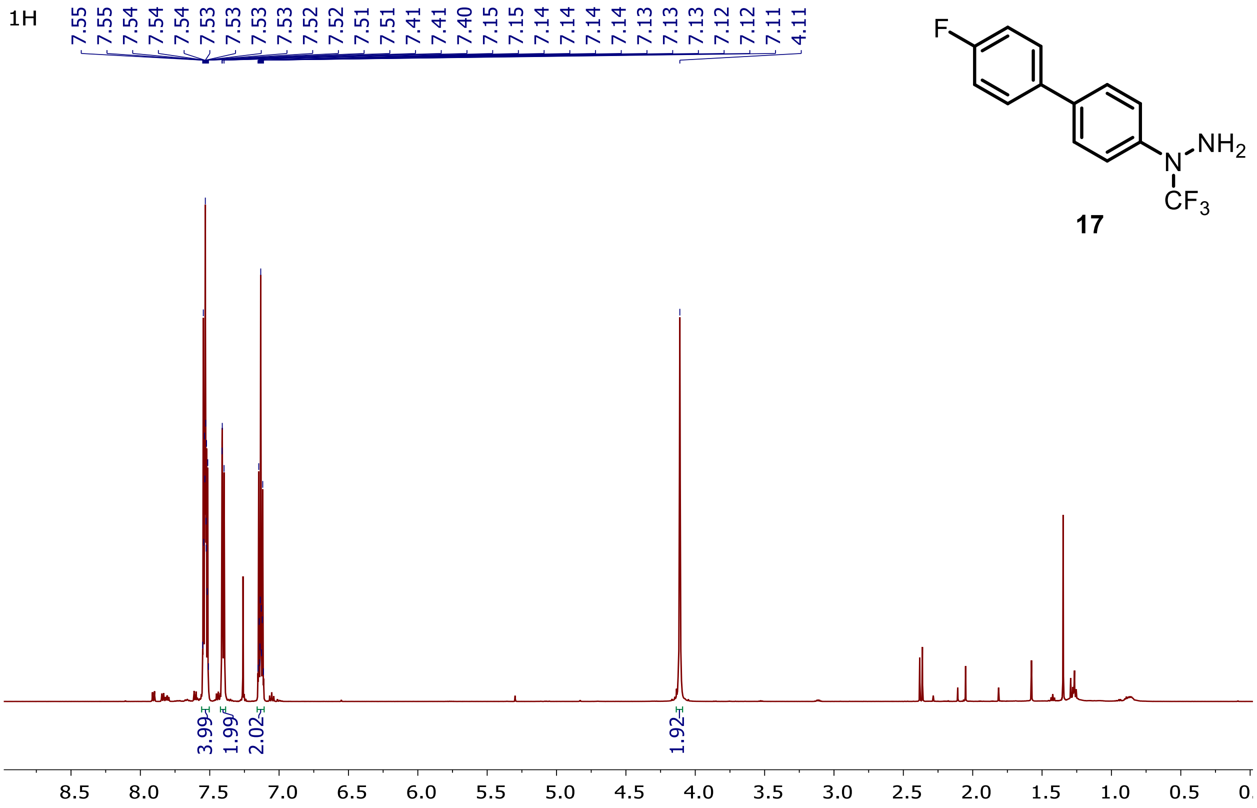
1H
7.55
7.55
7.54
7.54
7.54
7.53
7.53
7.53
7.53
7.52
7.52
7.51
7.51
7.41
7.41
7.40
7.15
7.15
7.14
7.14
7.14
7.14
7.13
7.13
7.13
7.12
7.12
7.11
4.11
F
NH2
N
CF3
17
3.99
1.99
2.02
1.92
8.5 8.0 7.5 7.0 6.5 6.0 5.5 5.0 4.5 4.0 3.5 3.0 2.5 2.0 1.5 1.0 0.5 0.

19F

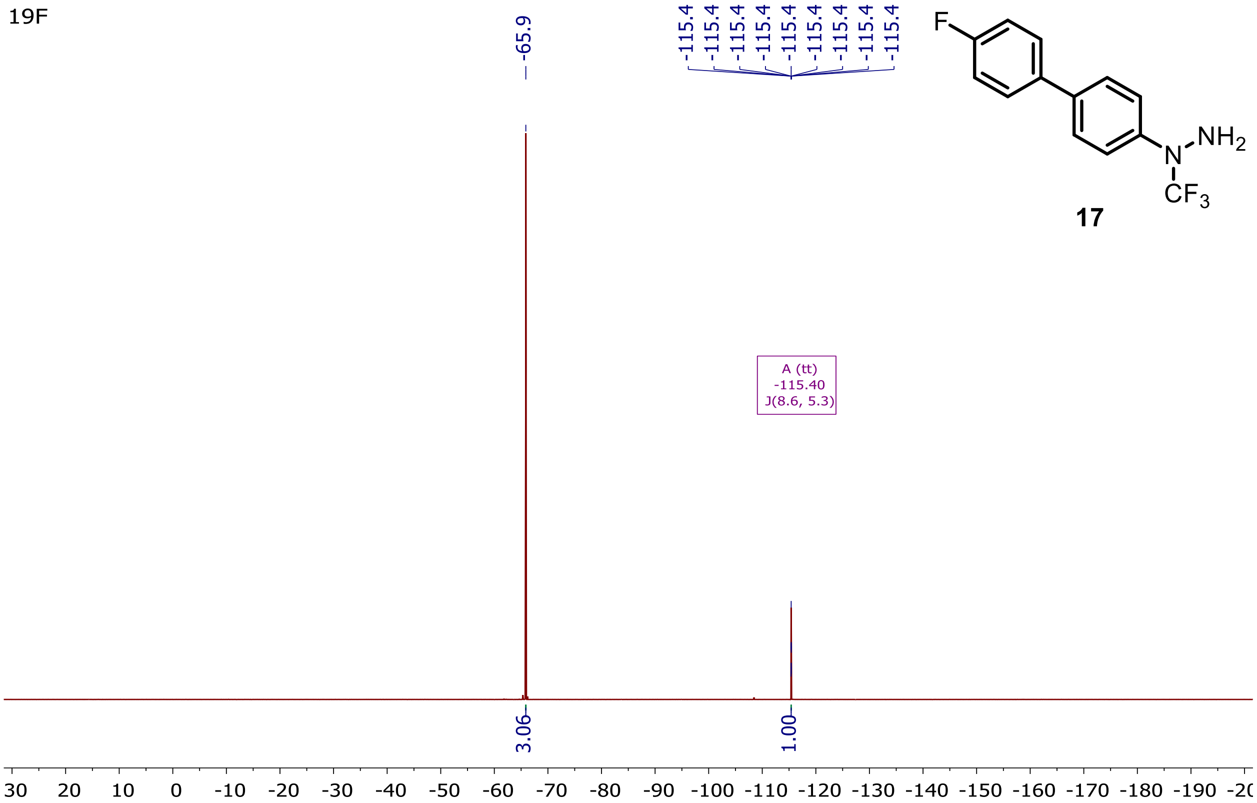

-65.9
-115.4
A (tt)
-115.40
J(8.6, 5.3)
3.06
1.00
30 20 10 0 -10 -20 -30 -40 -50 -60 -70 -80 -90 -100 -110 -120 -130 -140 -150 -160 -170 -180 -190
F
NH2
N
CF3
17

13C

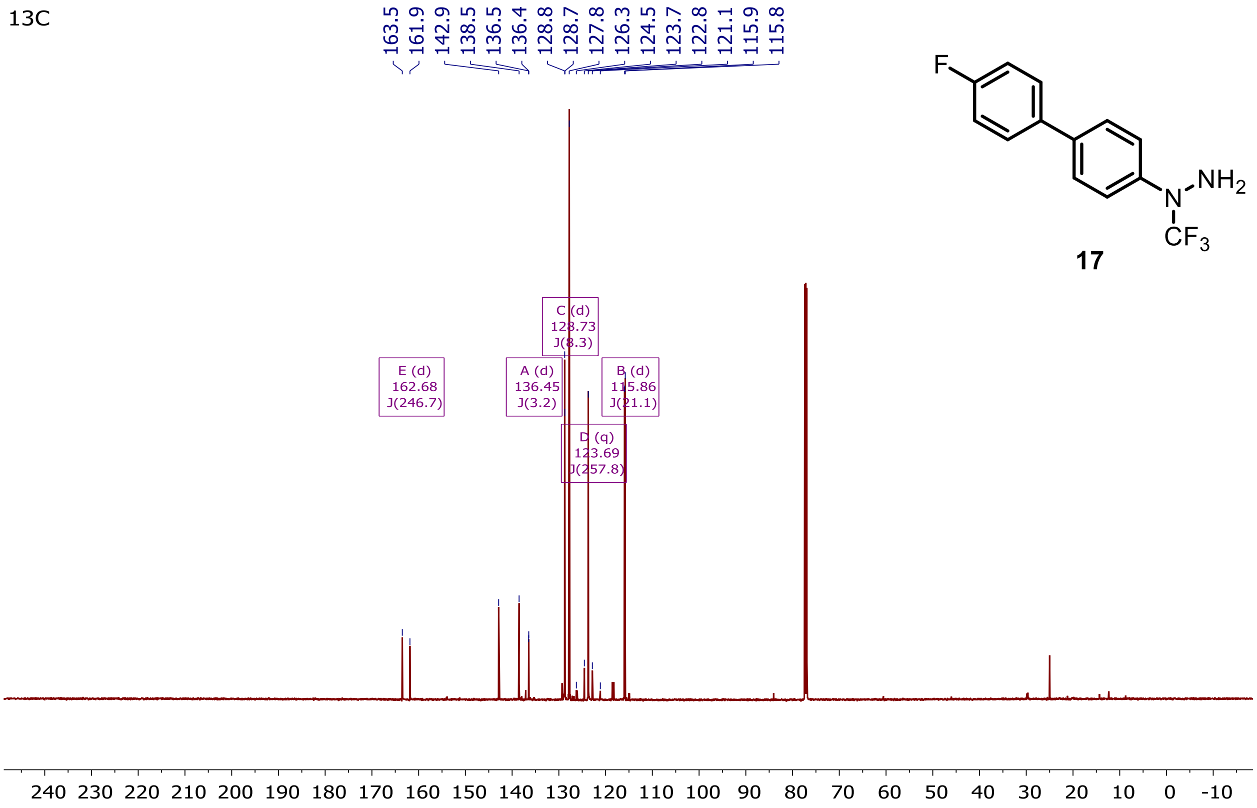

163.5
161.9
142.9
138.5
136.5
136.4
128.8
128.7
127.8
126.3
124.5
123.7
122.8
121.1
115.9
115.8
E (d)
162.68
J(246.7)
A (d)
136.45
J(3.2)
C (d)
128.73
J(8.3)
B (d)
115.86
J(21.1)
D (q)
123.69
J(257.8)
240 230 220 210 200 190 180 170 160 150 140 130 120 110 100 90 80 70 60 50 40 30 20 10 0 -10
F
NH2
N
CF3
17

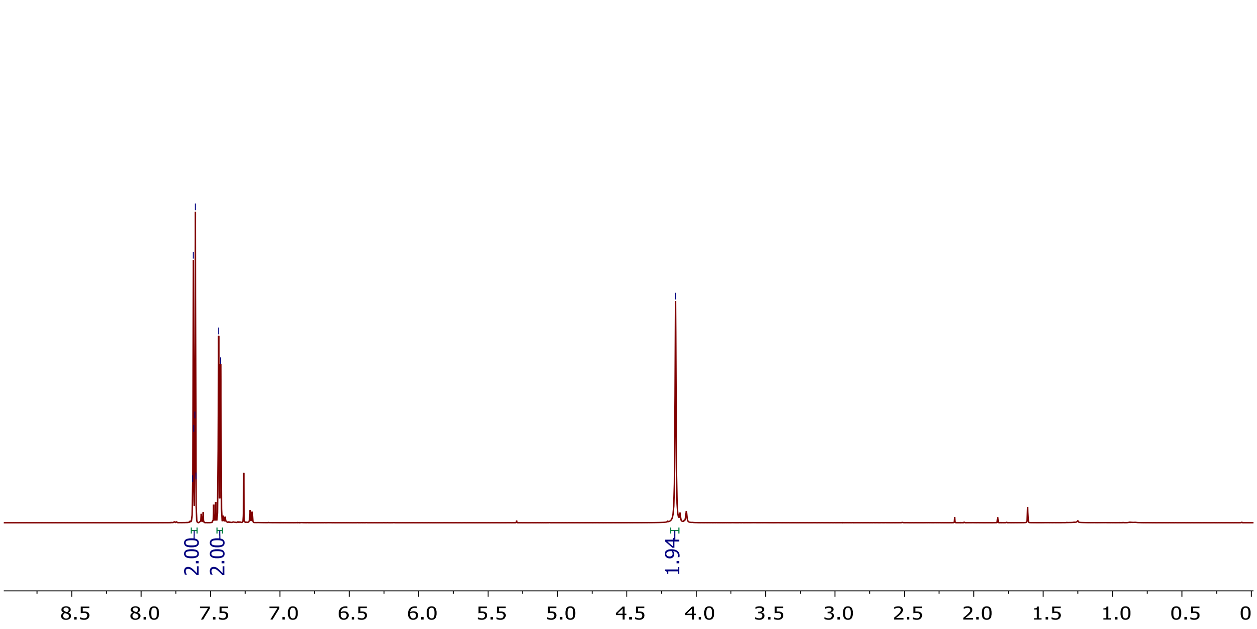
1H
7.63
7.62
7.62
7.61
7.61
7.60
7.44
7.43
4.15
2.00
2.00
1.94
8.5 8.0 7.5 7.0 6.5 6.0 5.5 5.0 4.5 4.0 3.5 3.0 2.5 2.0 1.5 1.0 0.5 0

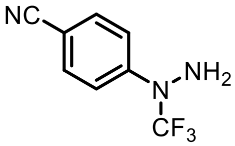
NC
NH2
N
CF3

18

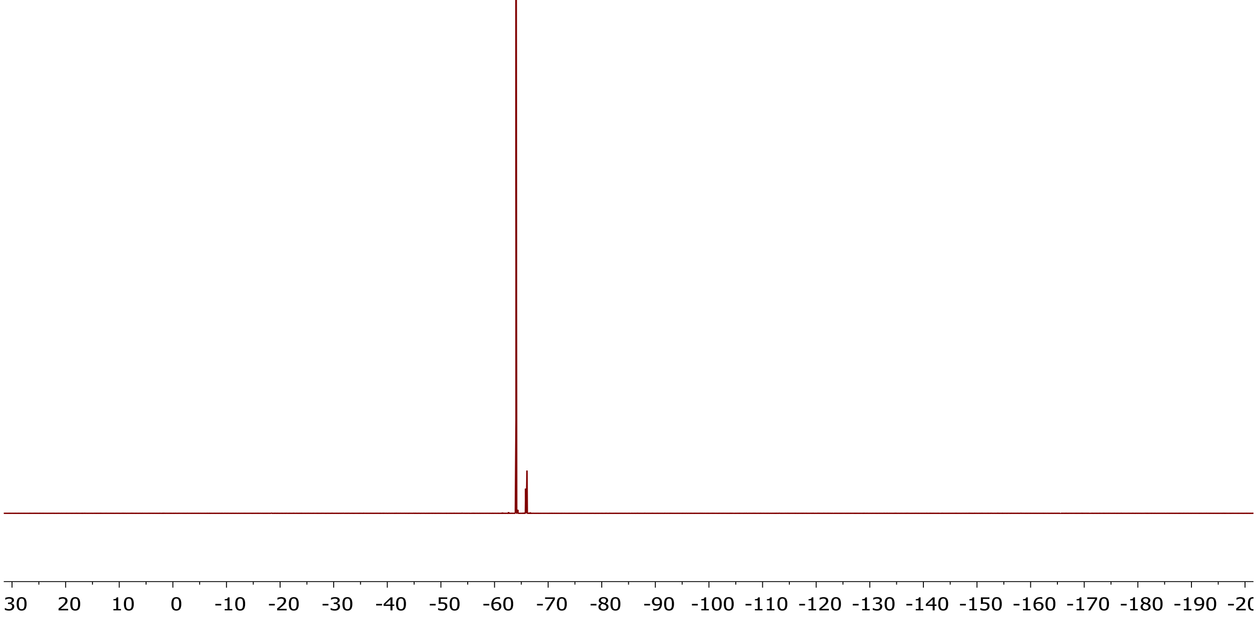
19F
-64.0
30 20 10 0 -10 -20 -30 -40 -50 -60 -70 -80 -90 -100 -110 -120 -130 -140 -150 -160 -170 -180 -190 -20

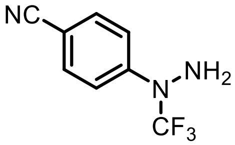
NC
NH2
N
CF3

18

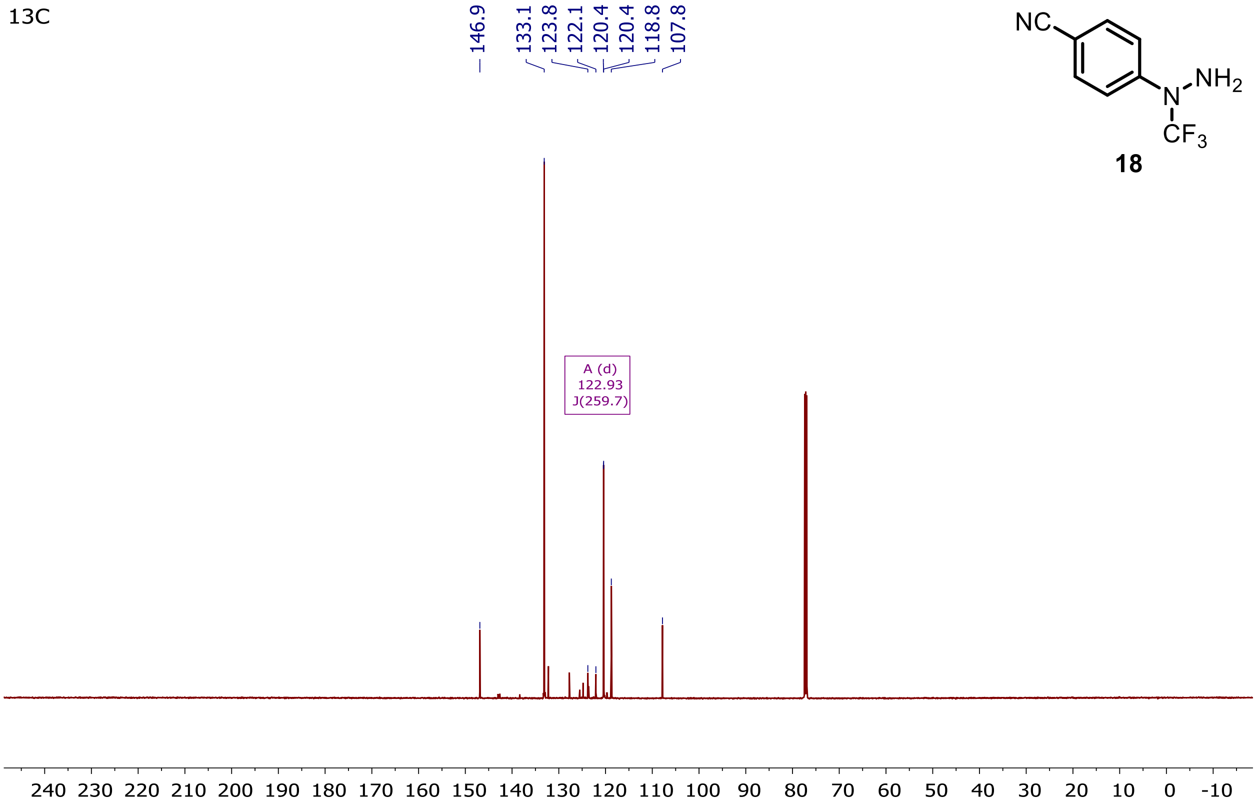
13C
146.9
133.1
123.8
122.1
120.4
120.4
118.8
107.8
A (d)
122.93
J(259.7)
240 230 220 210 200 190 180 170 160 150 140 130 120 110 100 90 80 70 60 50 40 30 20 10 0 -10
NC
NH2
N
CF3

18

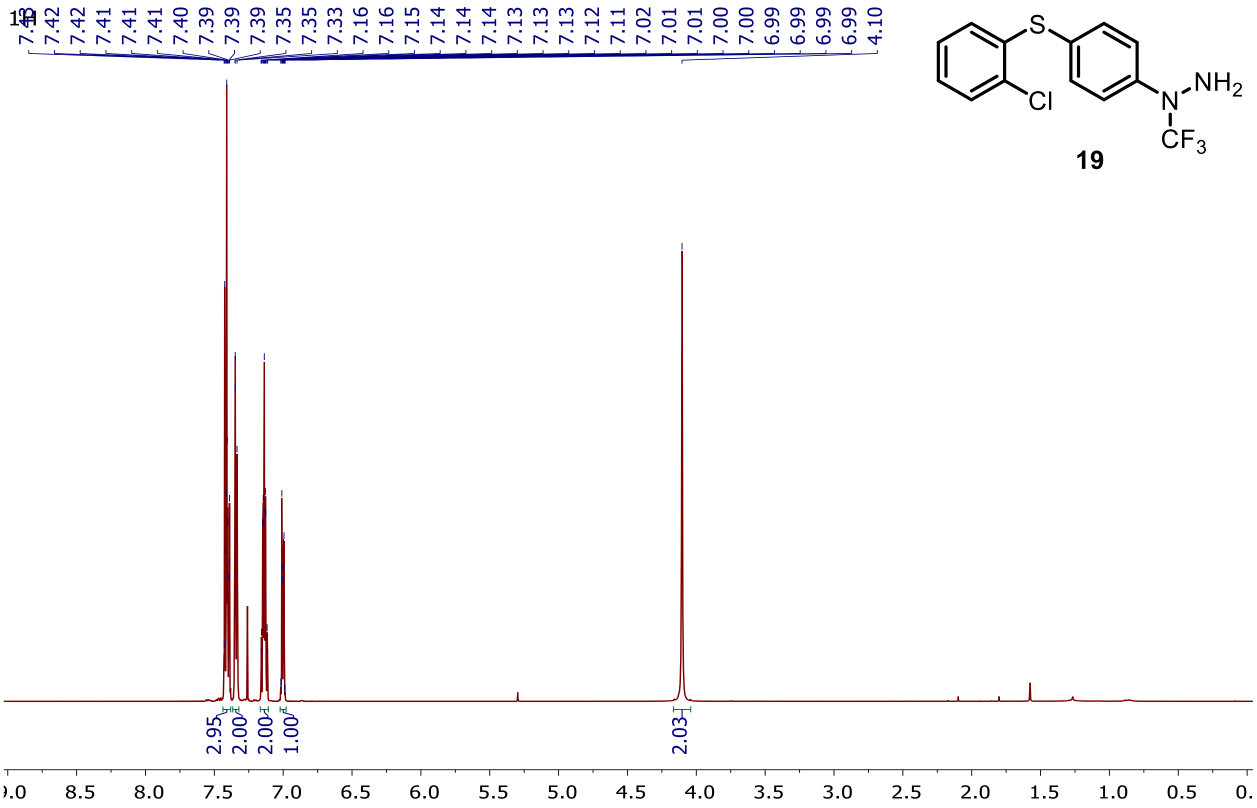
1H
7.42
7.42
7.41
7.41
7.40
7.39
7.39
7.39
7.35
7.35
7.33
7.16
7.16
7.15
7.14
7.14
7.14
7.13
7.13
7.13
7.12
7.11
7.02
7.01
7.01
7.00
7.00
6.99
6.99
6.99
6.99
4.10
2.95
2.00
2.00
1.00
2.03
8.5 8.0 7.5 7.0 6.5 6.0 5.5 5.0 4.5 4.0 3.5 3.0 2.5 2.0 1.5 1.0 0.5
S
Cl
NH2
N
CF3

19

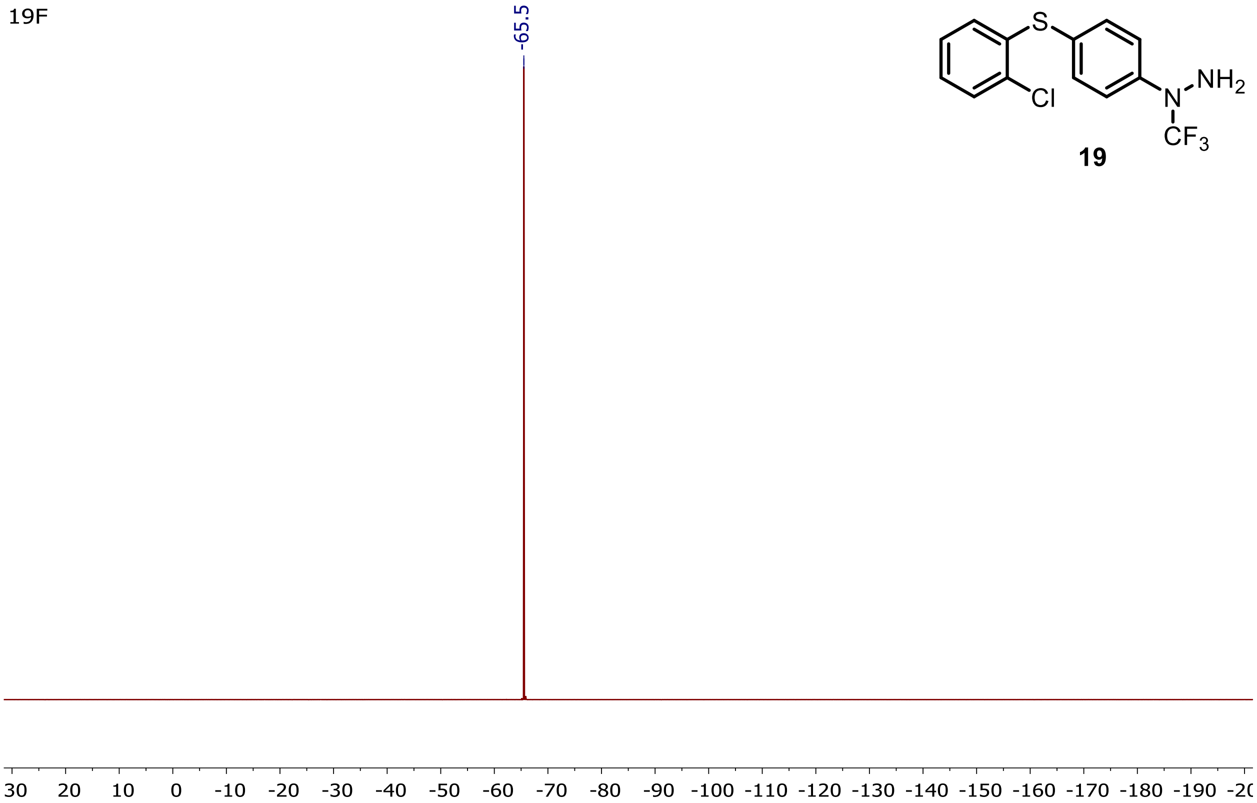
19F
-65.5
S
Cl
NH2
N
CF3
19
30 20 10 0 -10 -20 -30 -40 -50 -60 -70 -80 -90 -100 -110 -120 -130 -140 -150 -160 -170 -180 -190 -20

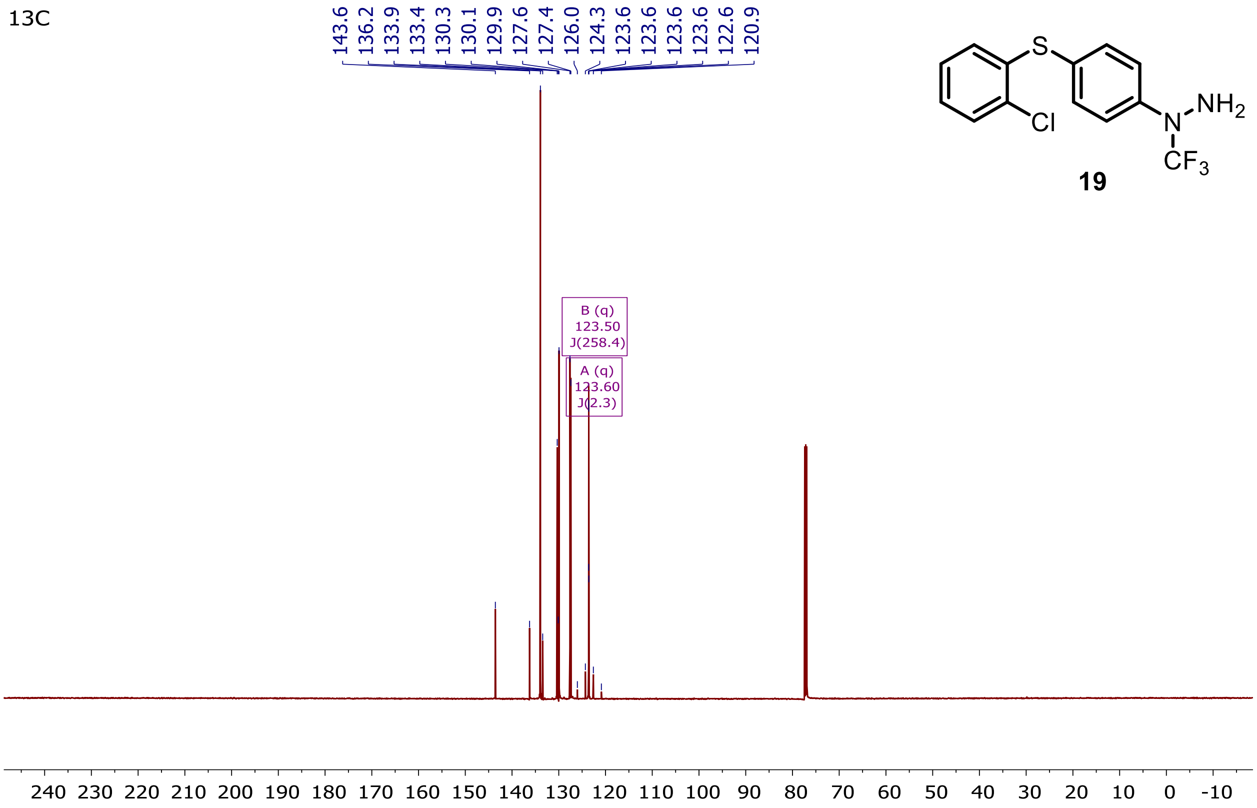
13C
143.6
136.2
133.9
133.4
130.3
130.1
129.9
127.6
127.4
126.0
124.3
123.6
123.6
123.6
123.6
122.6
120.9
S
Cl
NH2
N
CF3
19
B (q)
123.50
J(258.4)
A (q)
123.60
J(2.3)
240 230 220 210 200 190 180 170 160 150 140 130 120 110 100 90 80 70 60 50 40 30 20 10 0 -10

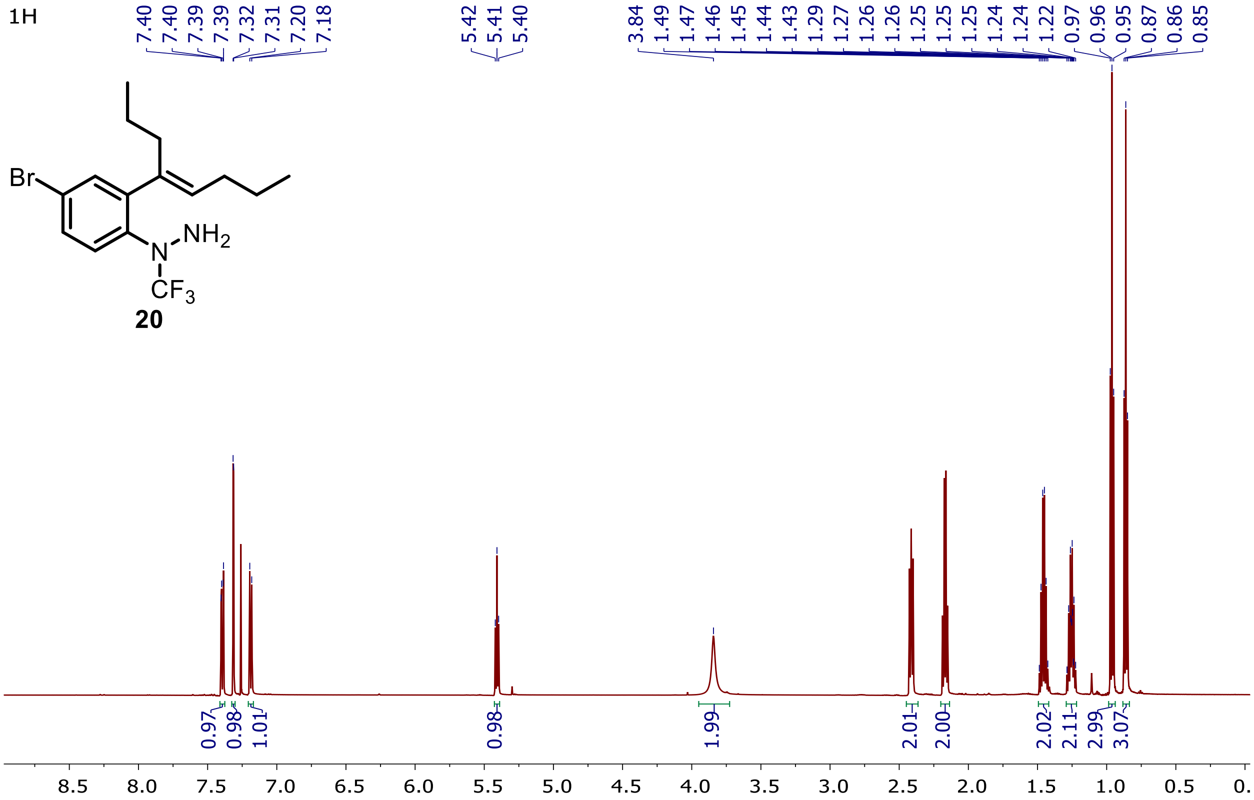
1H
7.40
7.40
7.39
7.39
7.32
7.31
7.20
7.18
5.42
5.41
5.40
3.84
1.49
1.47
1.46
1.45
1.44
1.43
1.29
1.27
1.26
1.26
1.25
1.25
1.25
1.24
1.24
1.22
0.97
0.96
0.95
0.87
0.86
0.85
Br
NH_2
N
CF_3
20
0.97
0.98
1.01
0.98
1.99
2.01
2.00
2.02
2.11
2.99
3.07
8.5 8.0 7.5 7.0 6.5 6.0 5.5 5.0 4.5 4.0 3.5 3.0 2.5 2.0 1.5 1.0 0.5 0.

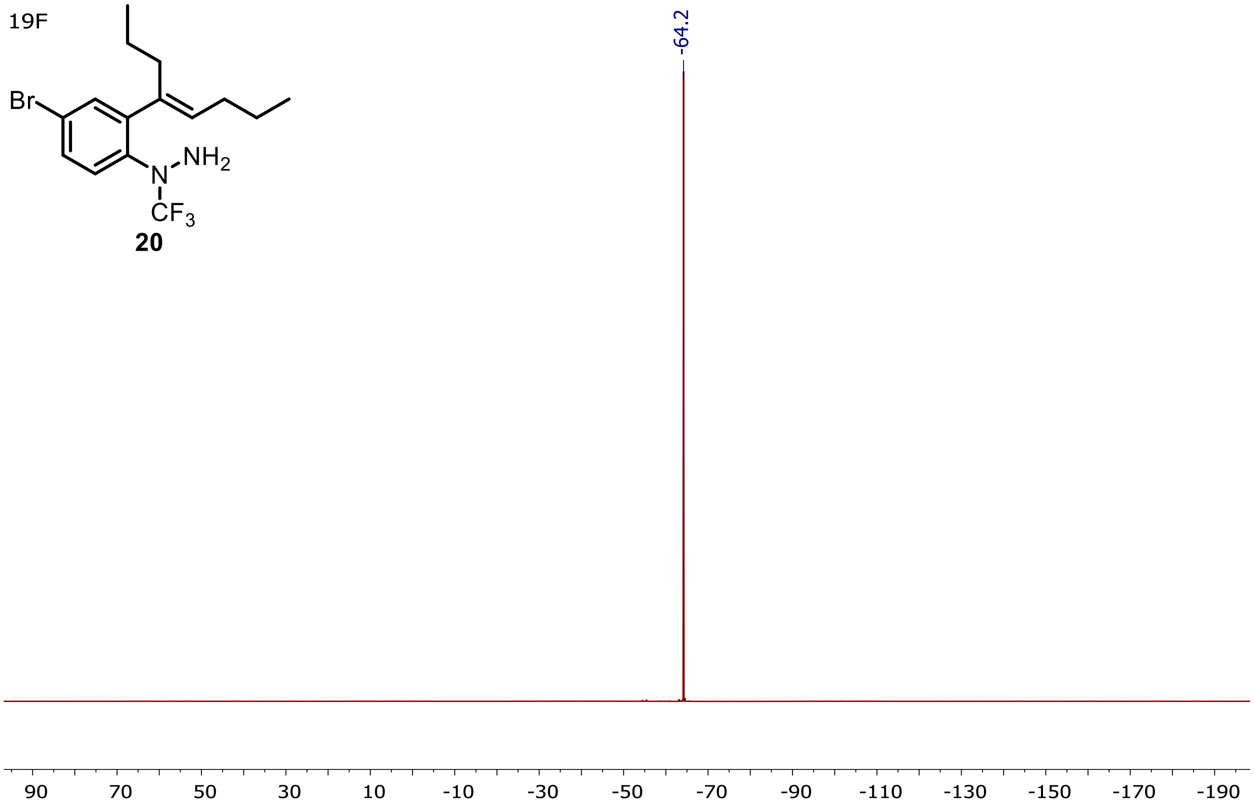
19F
-64.2
Br
NH_2
N
CF_3
20
90 70 50 30 10 -10 -30 -50 -70 -90 -110 -130 -150 -170 -190

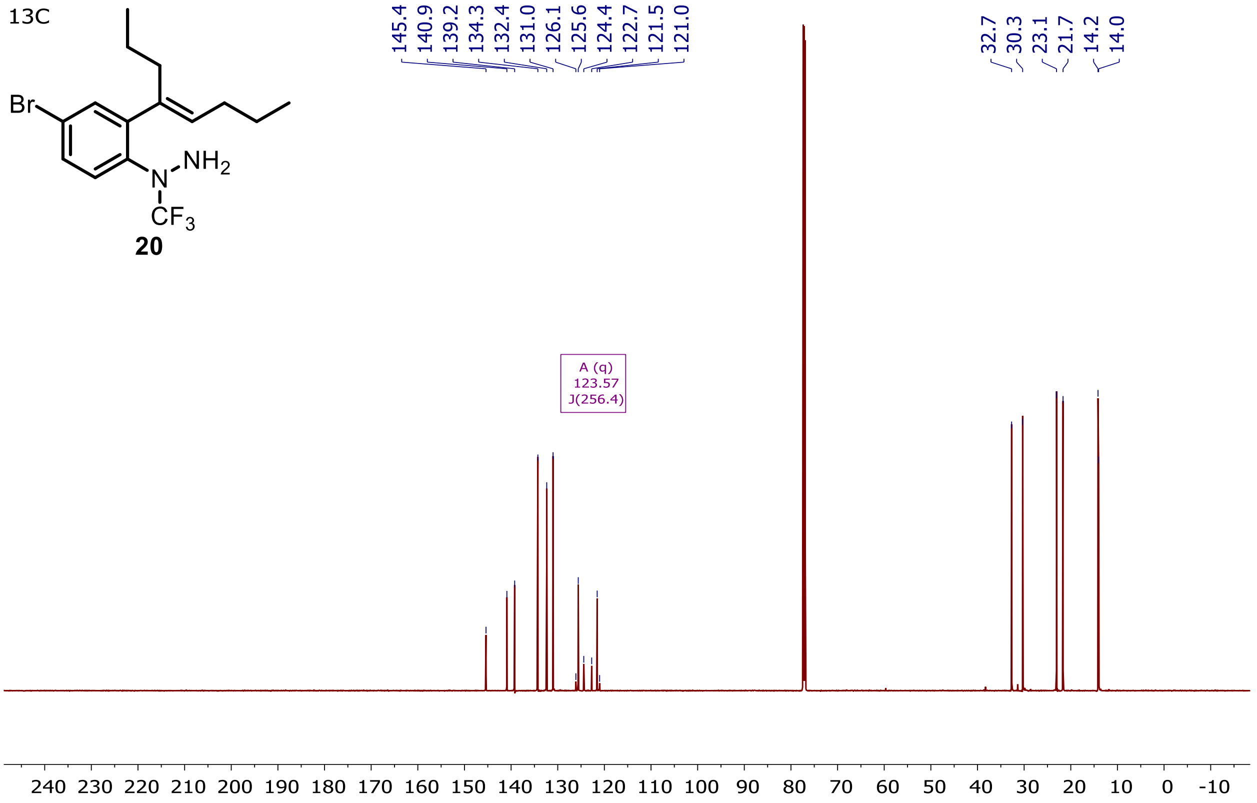
13C
Br
NH2
N
CF3
20
145.4
140.9
139.2
134.3
132.4
131.0
126.1
125.6
124.4
122.7
121.5
121.0
32.7
30.3
23.1
21.7
14.2
14.0
A (q)
123.57
J(256.4)
240 230 220 210 200 190 180 170 160 150 140 130 120 110 100 90 80 70 60 50 40 30 20 10 0 -10

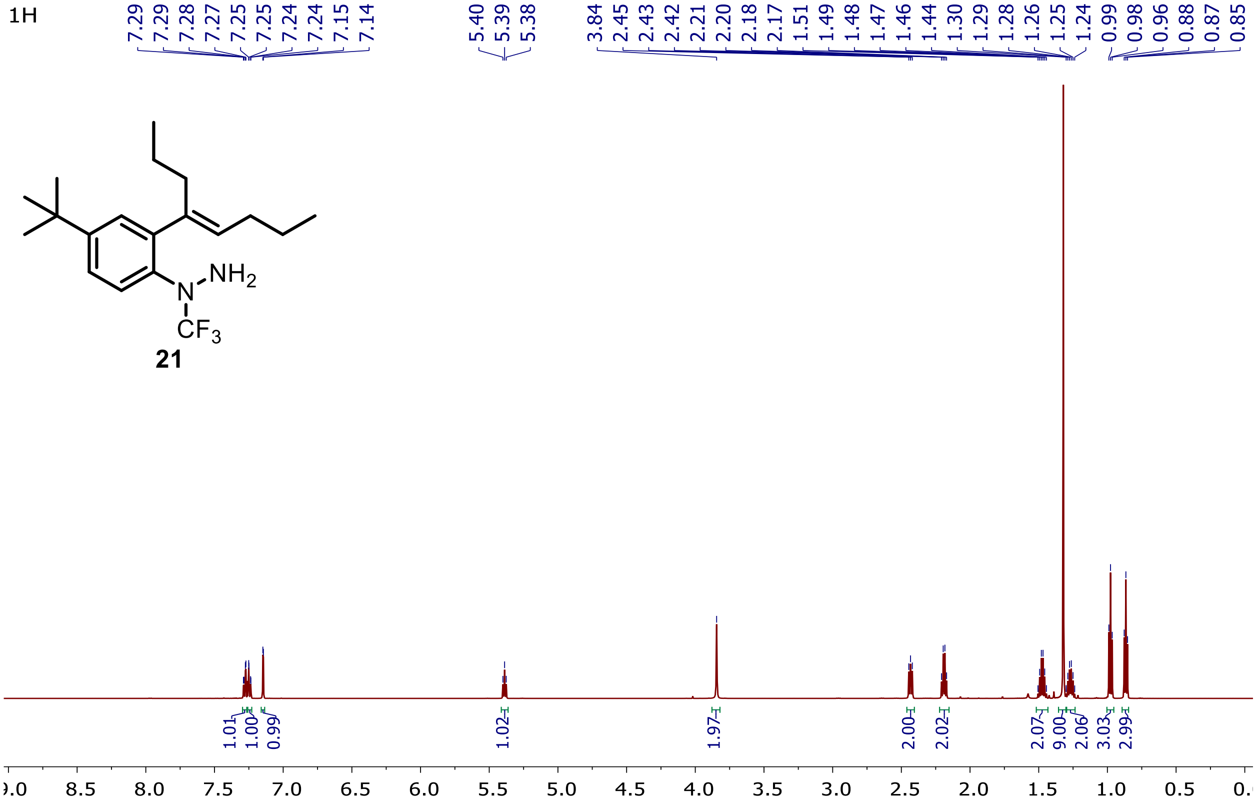
1H
NH2
N
CF3
21
7.29
7.29
7.28
7.27
7.25
7.25
7.24
7.24
7.15
7.14
5.40
5.39
5.38
3.84
2.45
2.43
2.42
2.21
2.20
2.18
2.17
1.51
1.49
1.48
1.47
1.46
1.44
1.30
1.29
1.28
1.26
1.25
1.24
0.99
0.98
0.96
0.88
0.87
0.85
1.01
1.00
0.99
1.02
1.97
2.00
2.02
2.07
9.00
2.06
3.03
2.99
8.5 8.0 7.5 7.0 6.5 6.0 5.5 5.0 4.5 4.0 3.5 3.0 2.5 2.0 1.5 1.0 0.5

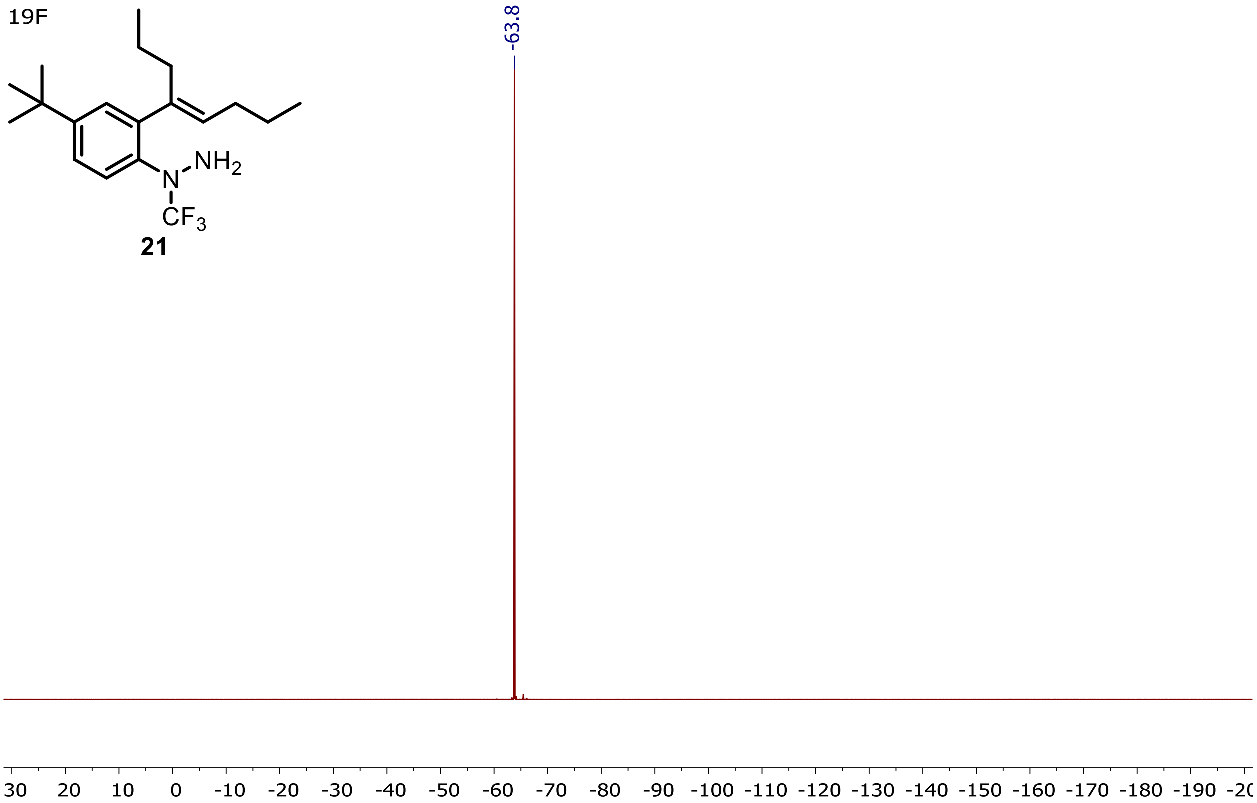
19F
NH2
N
CF3
21
-63.8
30 20 10 0 -10 -20 -30 -40 -50 -60 -70 -80 -90 -100 -110 -120 -130 -140 -150 -160 -170 -180 -190

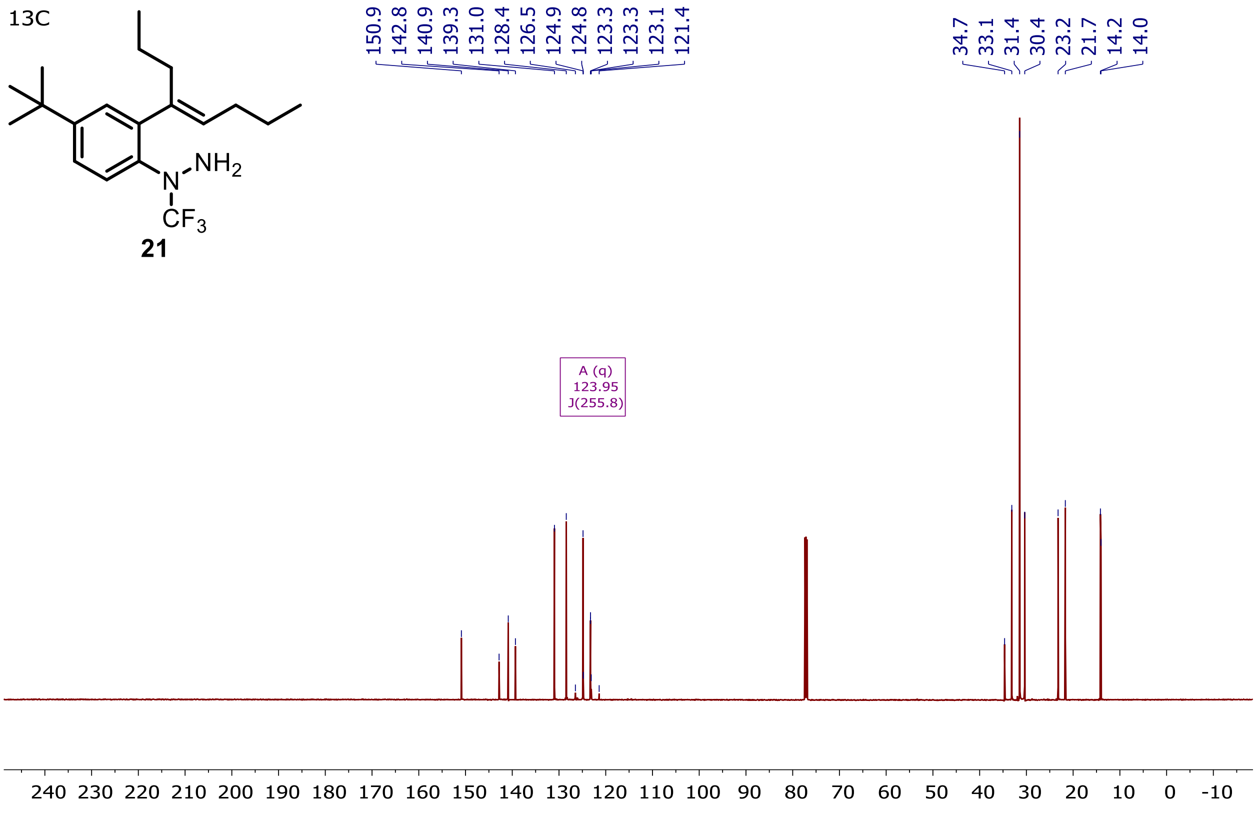
13C
NH2
N
CF3
21
150.9 142.8 140.9 139.3 131.0 128.4 126.5 124.9 124.8 123.3 123.3 123.1 121.4
34.7 33.1 31.4 30.4 23.2 21.7 14.2 14.0
A (q)
123.95
J(255.8)
240 230 220 210 200 190 180 170 160 150 140 130 120 110 100 90 80 70 60 50 40 30 20 10 0 -10

Indoles

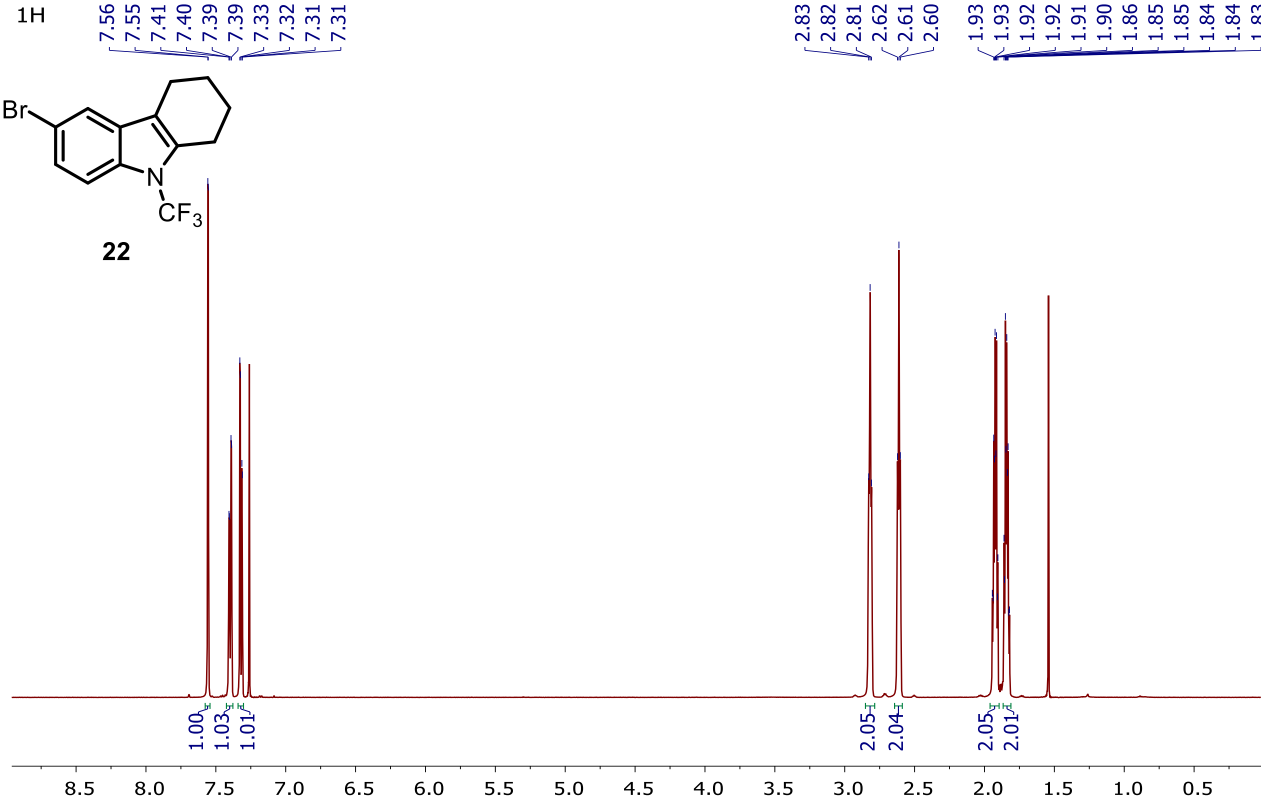
1H
7.56
7.55
7.41
7.40
7.39
7.39
7.33
7.32
7.31
7.31
2.83
2.82
2.81
2.62
2.61
2.60
1.93
1.93
1.92
1.92
1.91
1.90
1.86
1.85
1.85
1.84
1.84
Br
N
CF3
22
1.00
1.03
1.01
2.05
2.04
2.05
2.01
8.5 8.0 7.5 7.0 6.5 6.0 5.5 5.0 4.5 4.0 3.5 3.0 2.5 2.0 1.5 1.0 0.5

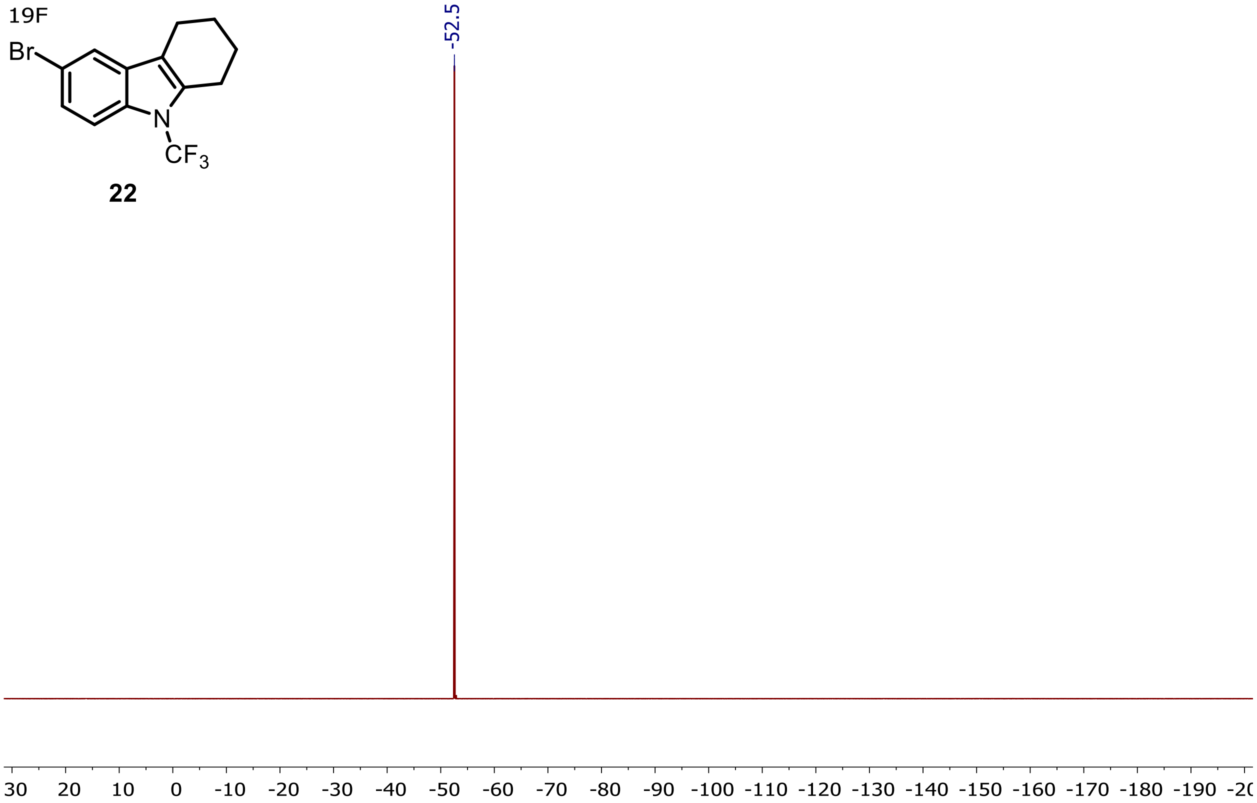
19F
Br
N
CF3
22
-52.5
30 20 10 0 -10 -20 -30 -40 -50 -60 -70 -80 -90 -100 -110 -120 -130 -140 -150 -160 -170 -180 -190

13C

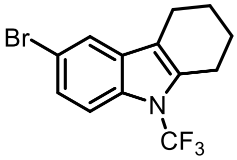

22

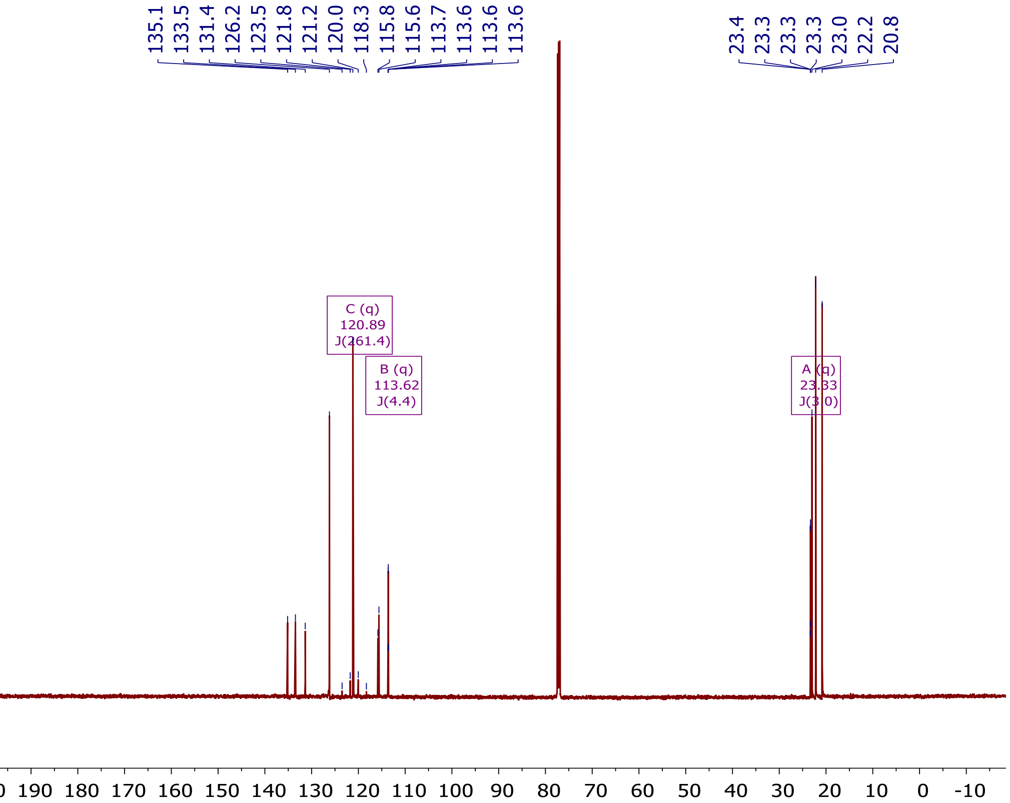

135.1
133.5
131.4
126.2
123.5
121.8
121.2
120.0
118.3
115.8
115.6
113.7
113.6
113.6
113.6
23.4
23.3
23.3
23.3
23.0
22.2
20.8
C (q)
120.89
J(261.4)
B (q)
113.62
J(4.4)
A (q)
23.33
J(3.0)
240 230 220 210 200 190 180 170 160 150 140 130 120 110 100 90 80 70 60 50 40 30 20 10 0 -10

1H

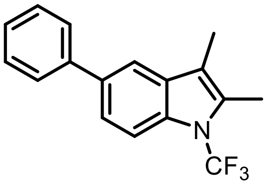

27

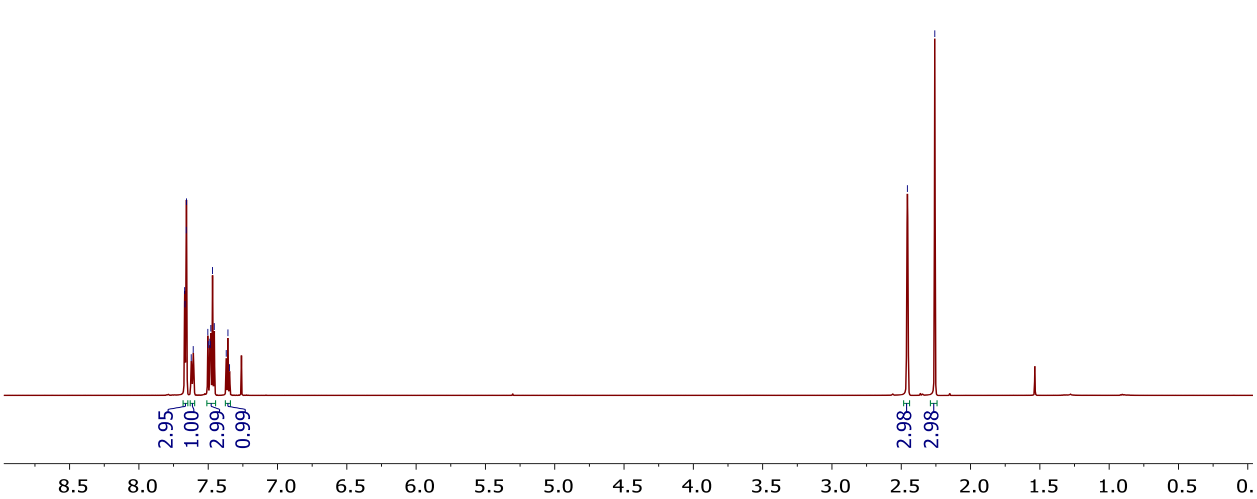

7.67
7.67
7.66
7.66
7.62
7.61
7.50
7.50
7.49
7.49
7.48
7.47
7.46
7.37
7.36
7.35
2.46
2.26
2.95
1.00
2.99
0.99
2.98
2.98
8.5 8.0 7.5 7.0 6.5 6.0 5.5 5.0 4.5 4.0 3.5 3.0 2.5 2.0 1.5 1.0 0.5 0.

19F

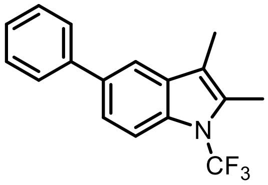

27

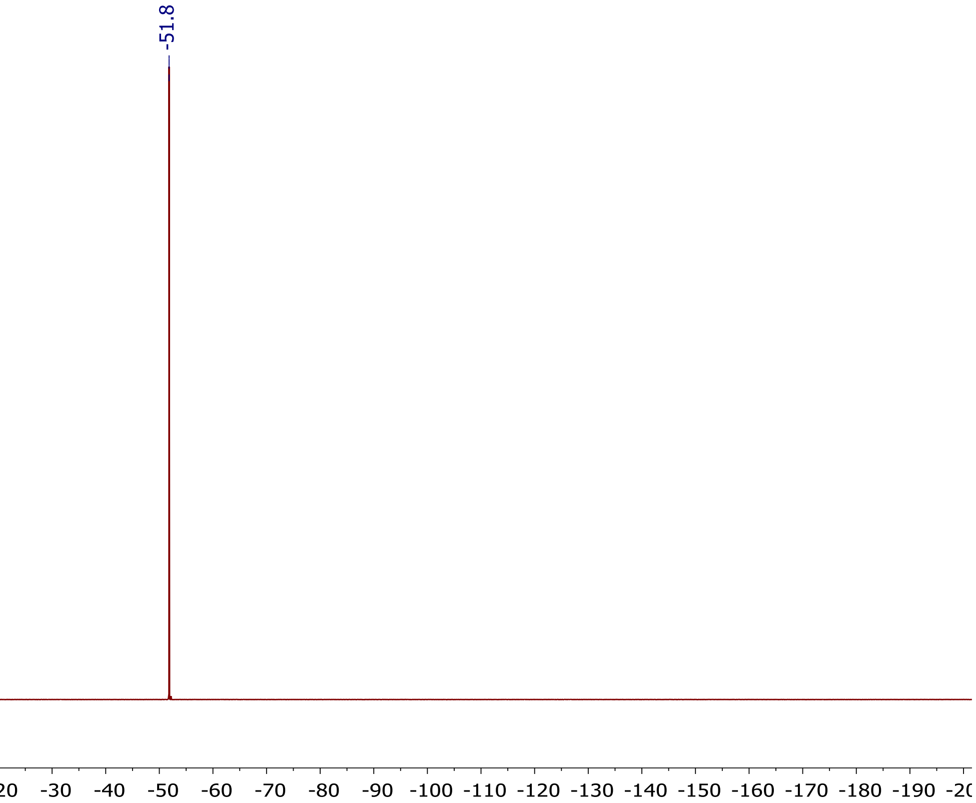
-51.8
30 20 10 0 -10 -20 -30 -40 -50 -60 -70 -80 -90 -100 -110 -120 -130 -140 -150 -160 -170 -180 -190 -2(

13C

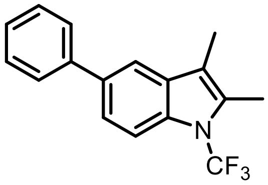

27

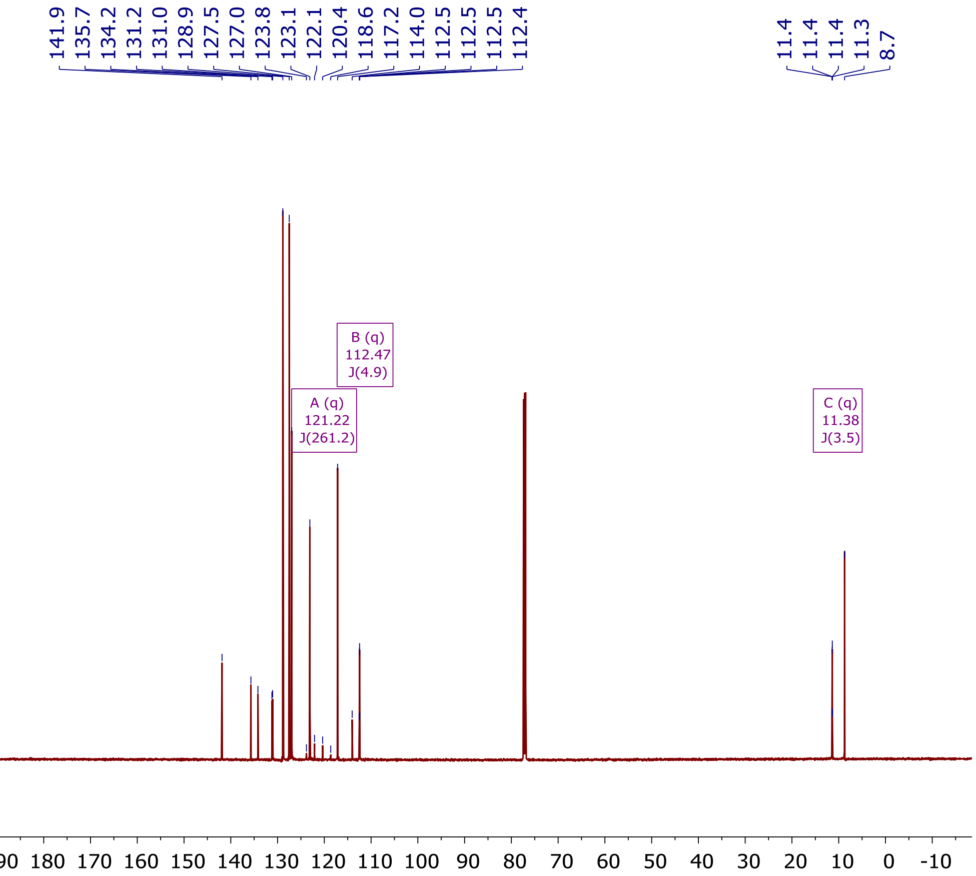
141.9 135.7 134.2 131.2 131.0 128.9 127.5 127.0 123.8 123.1 122.1 120.4 118.6 117.2 114.0 112.5 112.5 112.5 112.4
11.4 11.4 11.4 11.3 8.7
B (q)
112.47
J(4.9)
A (q)
121.22
J(261.2)
C (q)
11.38
J(3.5)
240 230 220 210 200 190 180 170 160 150 140 130 120 110 100 90 80 70 60 50 40 30 20 10 0 -10

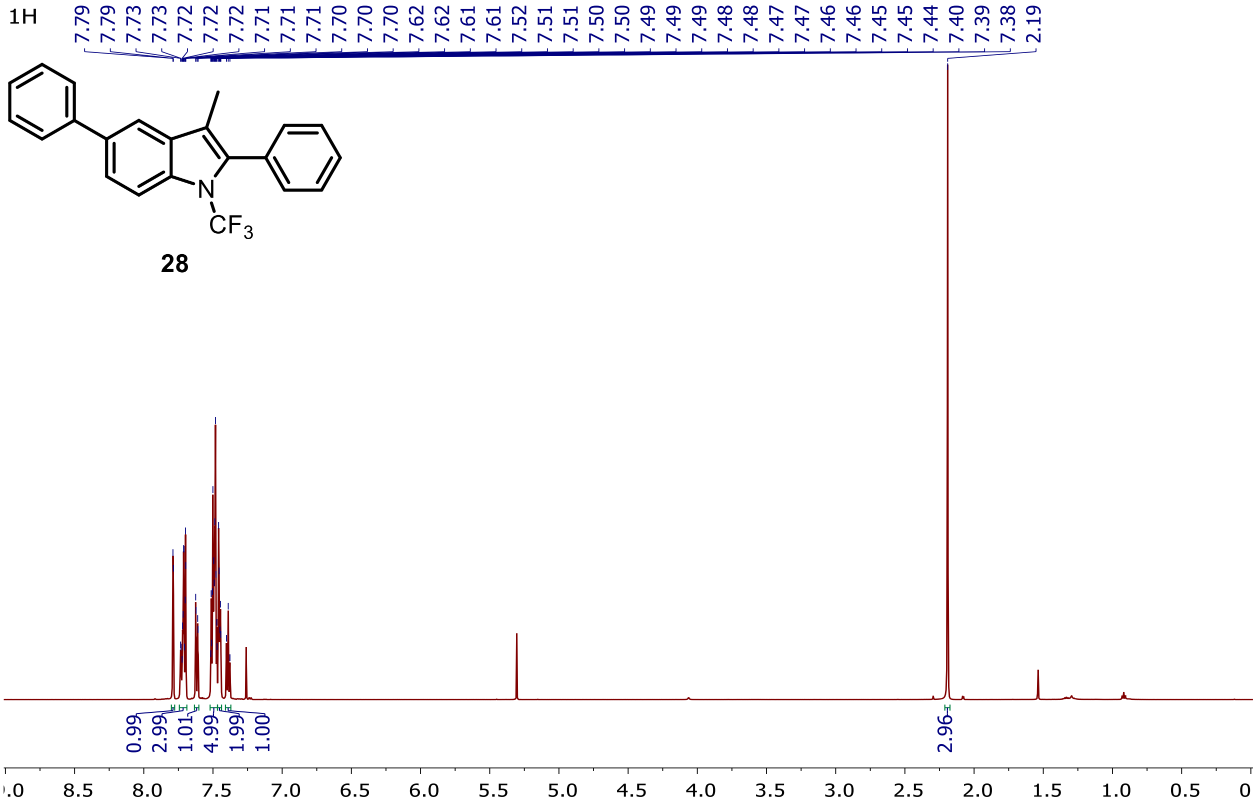
1H
7.79 7.79 7.73 7.73 7.72 7.72 7.72 7.71 7.71 7.71 7.70 7.70 7.70 7.62 7.62 7.61 7.61 7.52 7.51 7.51 7.50 7.50 7.49 7.49 7.49 7.48 7.48 7.47 7.47 7.46 7.46 7.45 7.45 7.44 7.40 7.39 7.38 2.19
N
CF_3
28
0.99 2.99 1.01 4.99 1.99 1.00
2.96
8.5 8.0 7.5 7.0 6.5 6.0 5.5 5.0 4.5 4.0 3.5 3.0 2.5 2.0 1.5 1.0 0.5 0

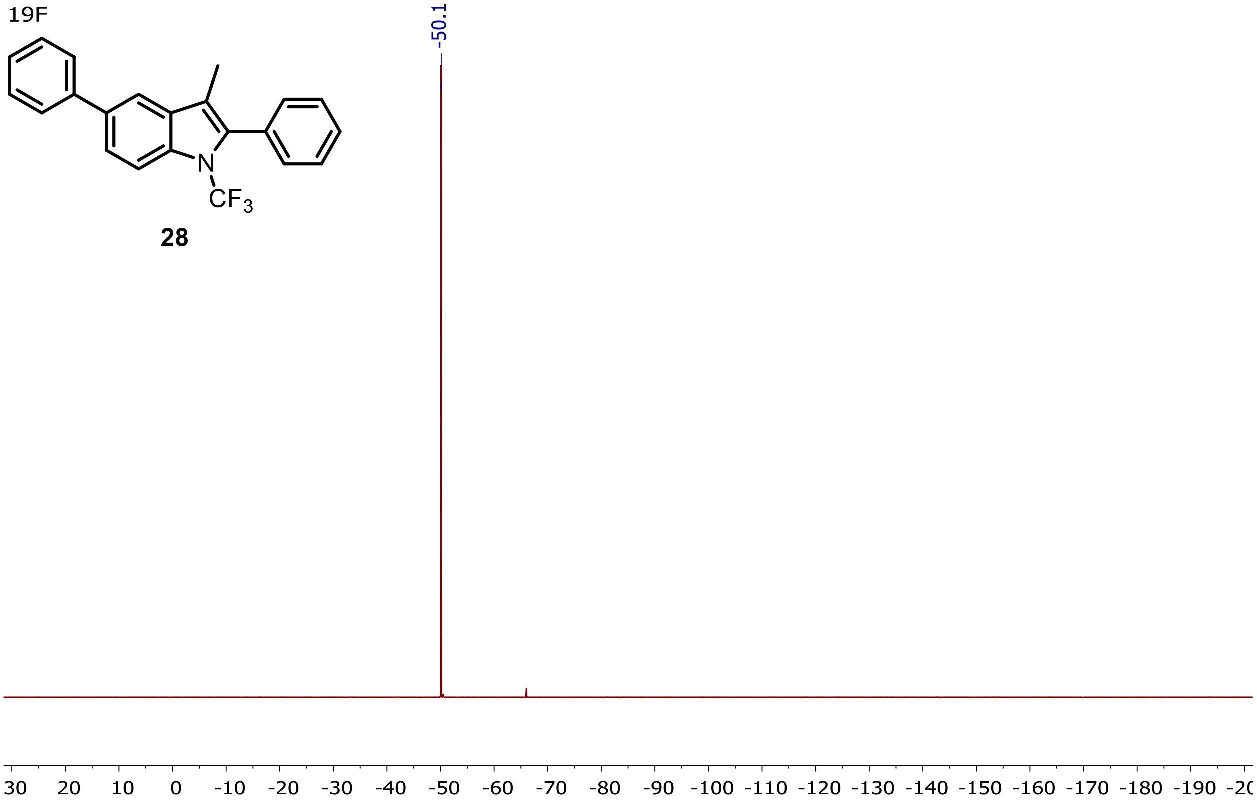
19F
-50.1
N
CF_3
28
30 20 10 0 -10 -20 -30 -40 -50 -60 -70 -80 -90 -100 -110 -120 -130 -140 -150 -160 -170 -180 -190

13C

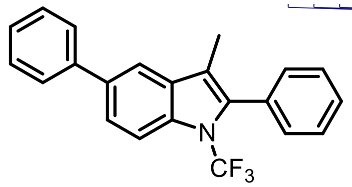

N
CF3

28

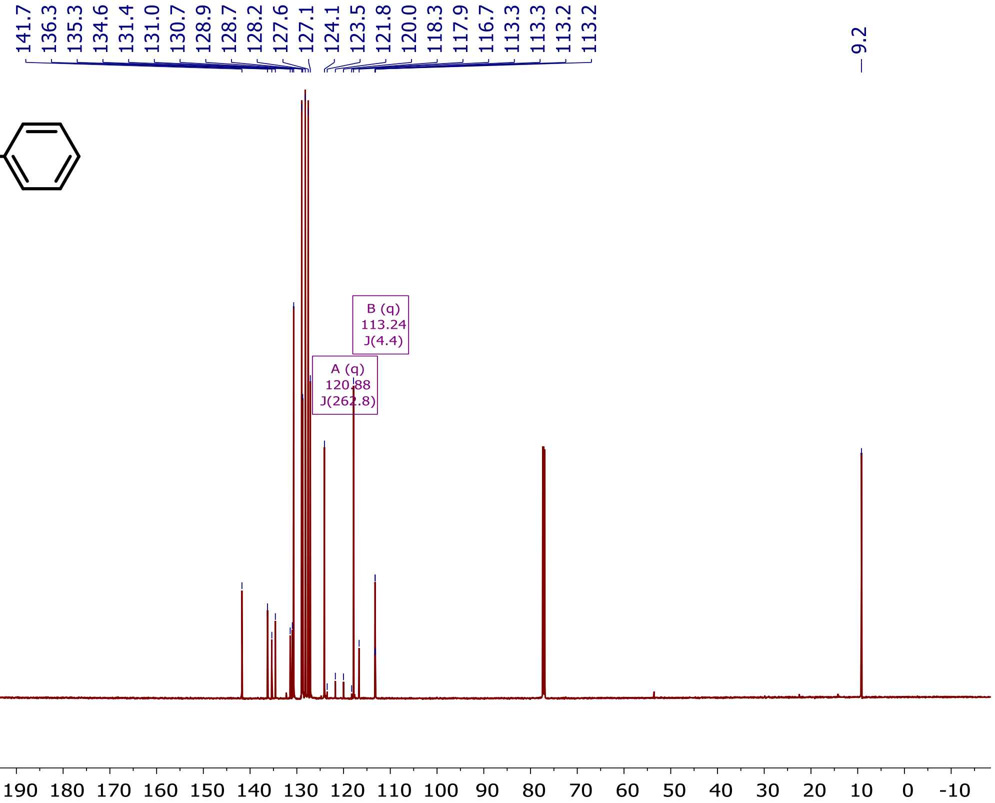

141.7
136.3
135.3
134.6
131.4
131.0
130.7
128.9
128.7
128.2
127.6
127.1
124.1
123.5
121.8
120.0
118.3
117.9
116.7
113.3
113.3
113.2
113.2
9.2
B (q)
113.24
J(4.4)
A (q)
120.88
J(262.8)
240 230 220 210 200 190 180 170 160 150 140 130 120 110 100 90 80 70 60 50 40 30 20 10 0 -10

Derivatized indoles

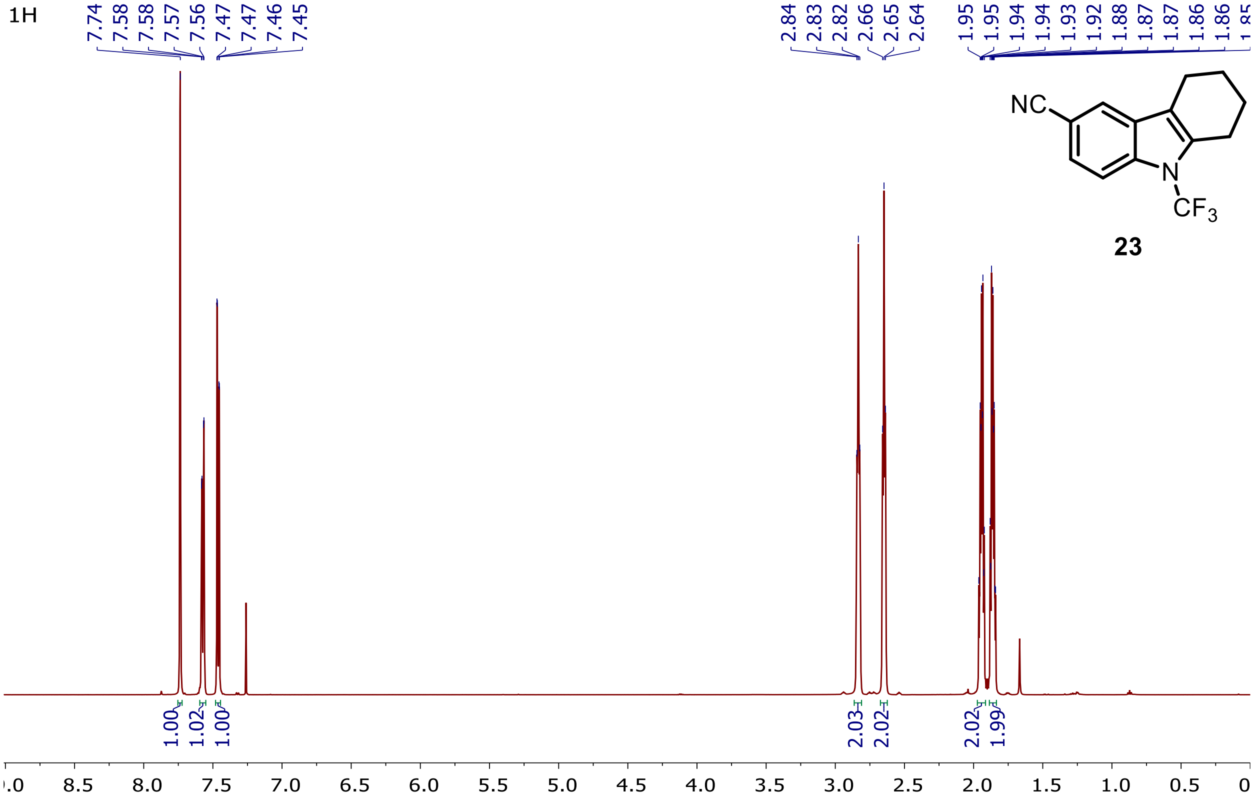

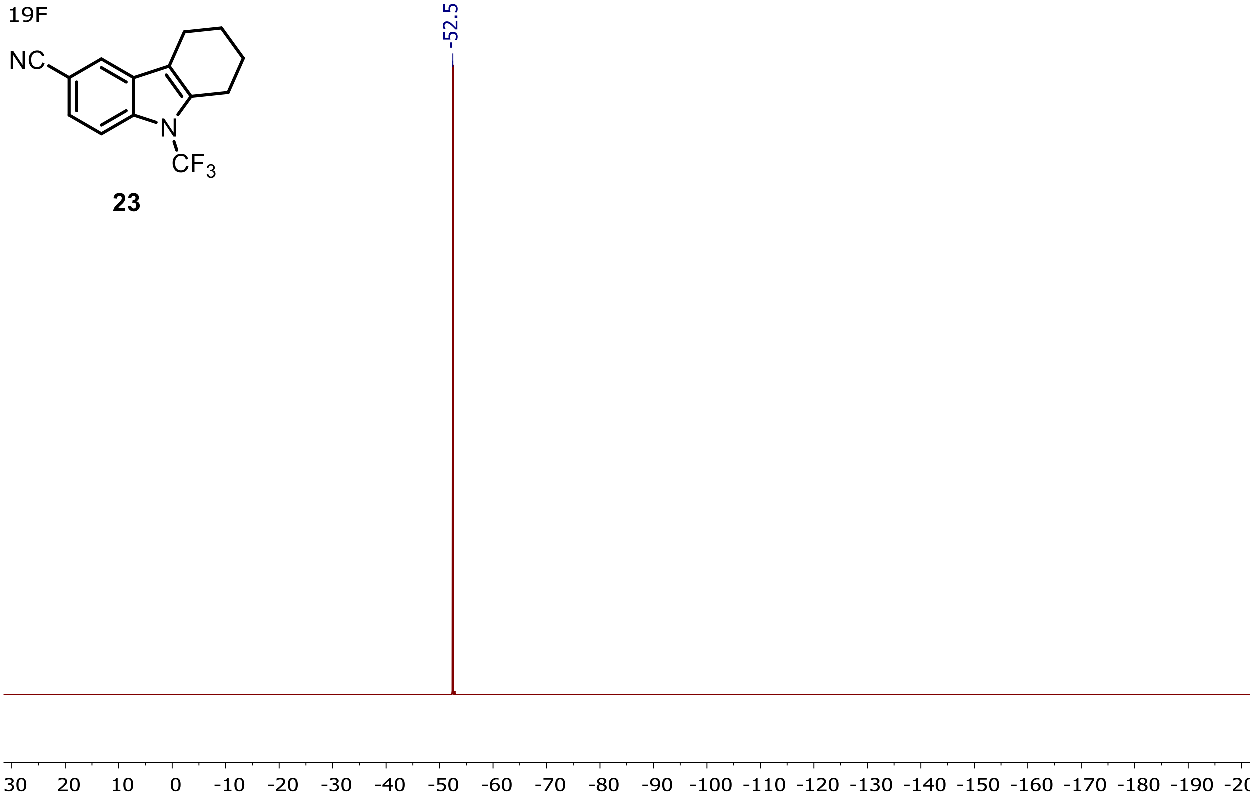

13C

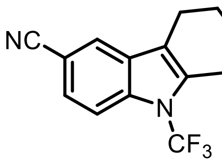

NC
N
CF3

23

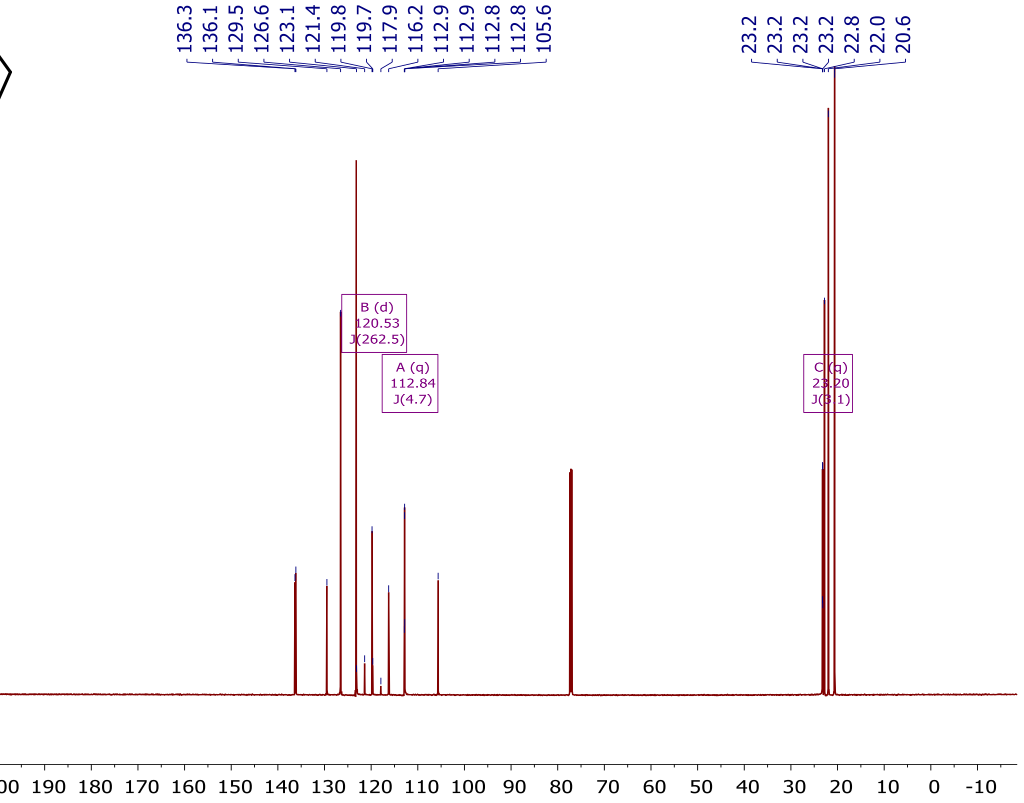

136.3
136.1
129.5
126.6
123.1
121.4
119.8
119.7
117.9
116.2
112.9
112.9
112.8
112.8
105.6
23.2
23.2
23.2
23.2
22.8
22.0
20.6
B (d)
120.53
J(262.5)
A (q)
112.84
J(4.7)
C (q)
23.20
J(3.1)
240 230 220 210 200 190 180 170 160 150 140 130 120 110 100 90 80 70 60 50 40 30 20 10 0 -10

1H

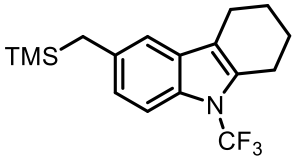

TMS
N
CF3

24

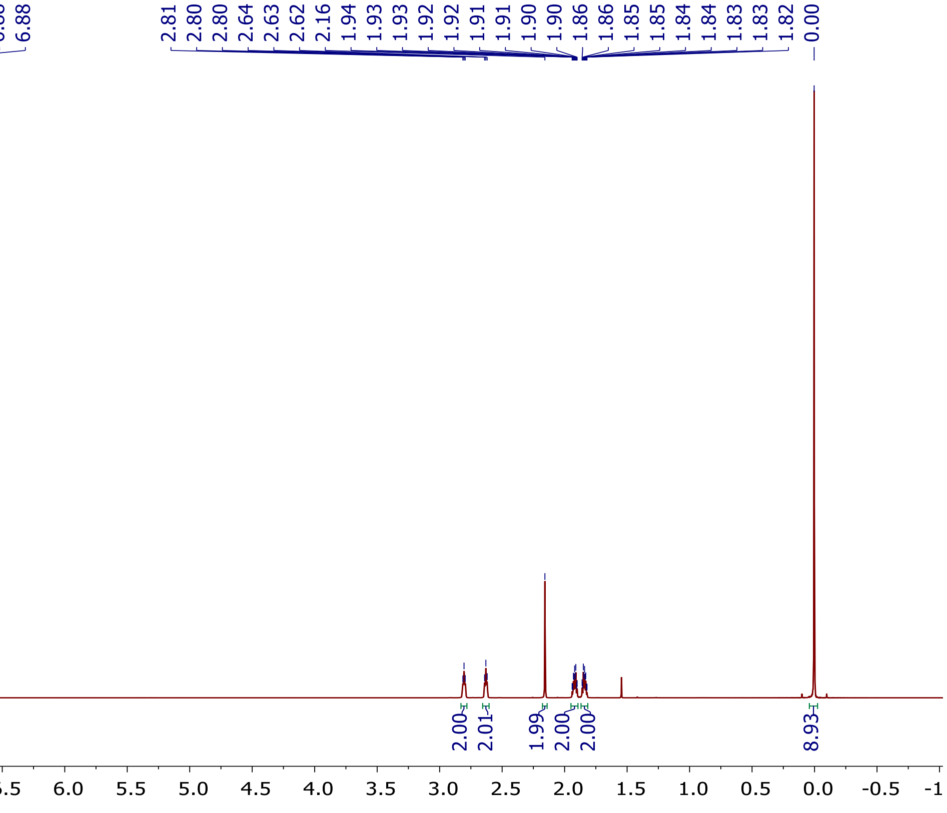

7.40
7.39
7.38
7.38
7.03
6.90
6.89
6.88
6.88
2.81
2.80
2.80
2.64
2.63
2.62
2.16
1.94
1.93
1.93
1.92
1.92
1.91
1.91
1.90
1.90
1.86
1.86
1.85
1.85
1.84
1.84
1.83
1.83
1.82
0.00
1.01
1.00
1.02
2.00
2.01
1.99
2.00
2.00
8.93
8.5 8.0 7.5 7.0 6.5 6.0 5.5 5.0 4.5 4.0 3.5 3.0 2.5 2.0 1.5 1.0 0.5 0.0 -0.5 -1

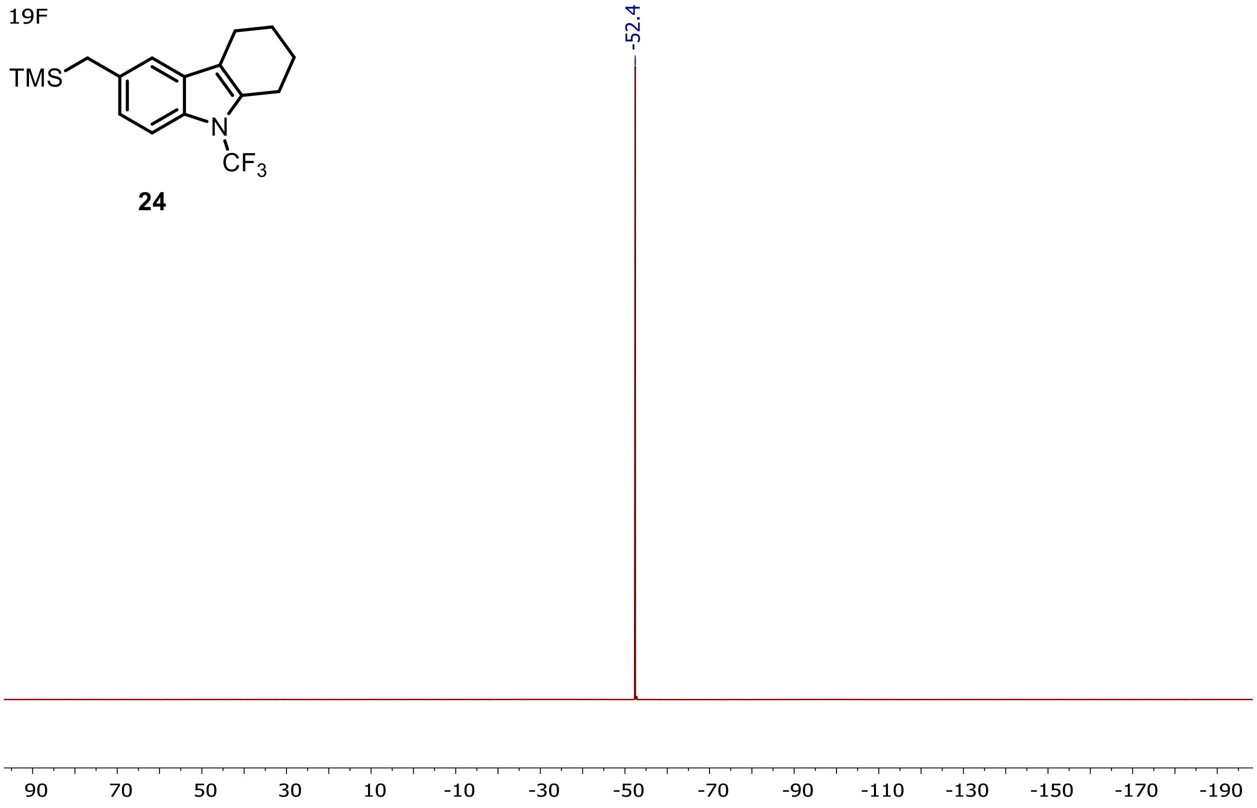

19F
TMS
N
CF3
24
-52.4
90 70 50 30 10 -10 -30 -50 -70 -90 -110 -130 -150 -170 -190

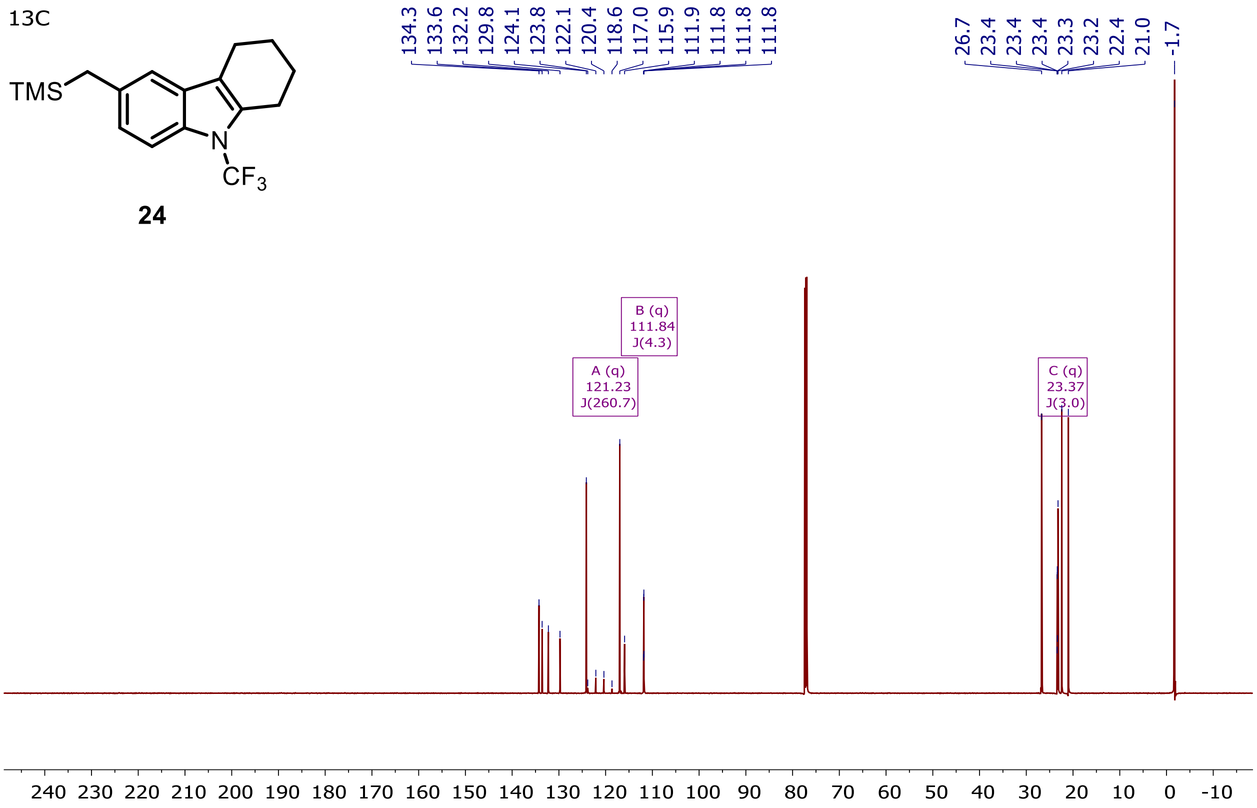

13C
TMS
N
CF3
24
134.3 133.6 132.2 129.8 124.1 123.8 122.1 120.4 118.6 117.0 115.9 111.9 111.8 111.8 111.8
26.7 23.4 23.4 23.4 23.3 23.2 22.4 21.0
-1.7
A (q) 121.23 J(260.7)
B (q) 111.84 J(4.3)
C (q) 23.37 J(3.0)
240 230 220 210 200 190 180 170 160 150 140 130 120 110 100 90 80 70 60 50 40 30 20 10 0 -10

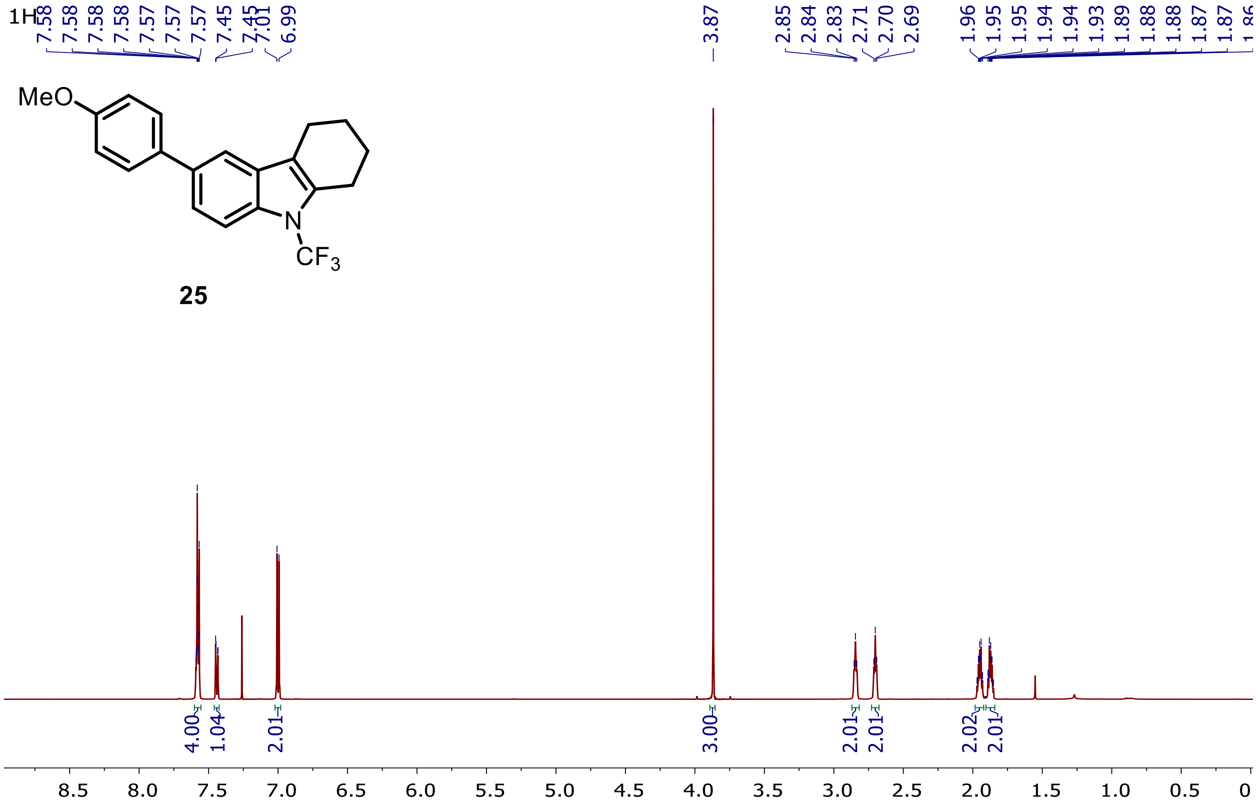
1H
7.58
7.58
7.58
7.58
7.57
7.57
7.57
7.45
7.45
7.01
6.99
3.87
2.85
2.84
2.83
2.71
2.70
2.69
1.96
1.95
1.95
1.94
1.94
1.93
1.89
1.88
1.88
1.87
1.87
MeO
N
CF3
25
4.00
1.04
2.01
3.00
2.01
2.01
2.02
2.01
8.5
8.0
7.5
7.0
6.5
6.0
5.5
5.0
4.5
4.0
3.5
3.0
2.5
2.0
1.5
1.0
0.5
0

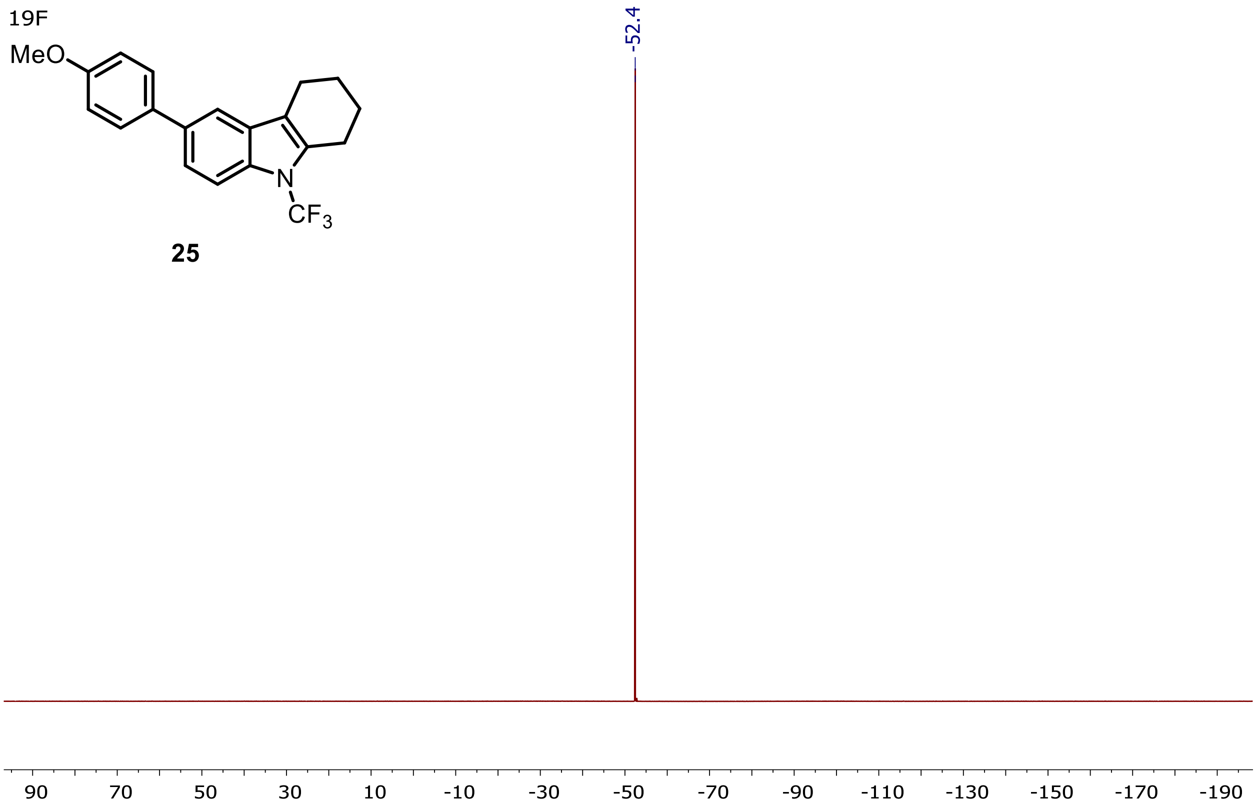
19F
-52.4
MeO
N
CF3
25
90
70
50
30
10
-10
-30
-50
-70
-90
-110
-130
-150
-170
-190

13C

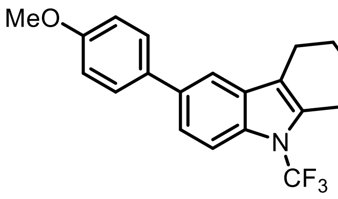

25

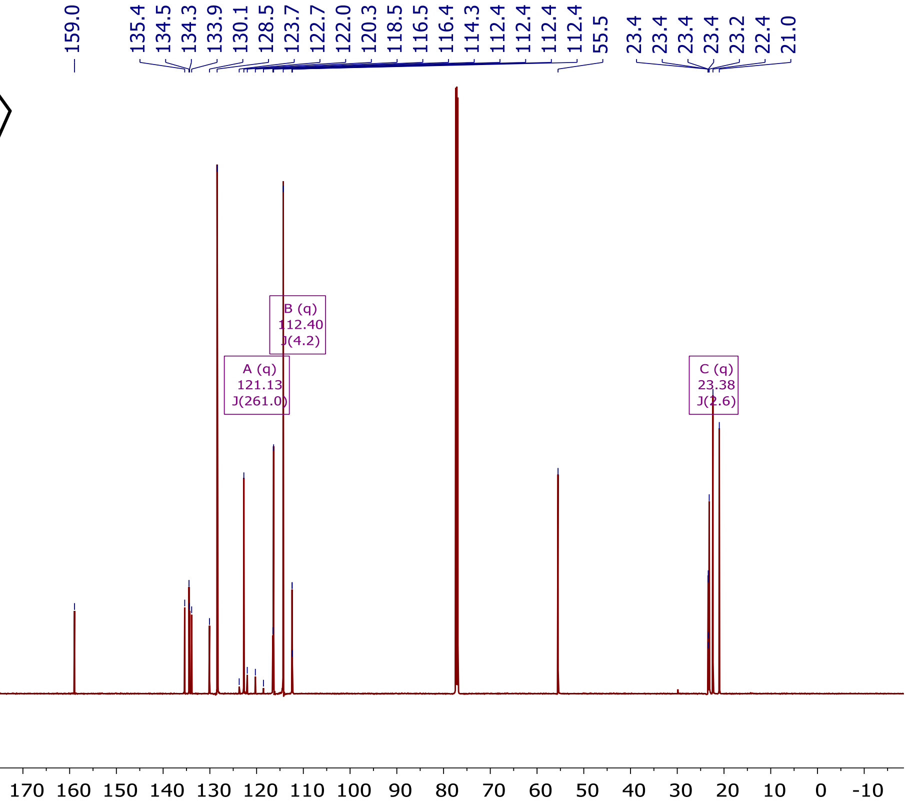

159.0
135.4
134.5
134.3
133.9
130.1
128.5
123.7
122.7
122.0
120.3
118.5
116.5
116.4
114.3
112.4
112.4
112.4
112.4
55.5
23.4
23.4
23.4
23.4
23.2
22.4
21.0
A (q)
121.13
J(261.0)
B (q)
112.40
J(4.2)
C (q)
23.38
J(2.6)
240 230 220 210 200 190 180 170 160 150 140 130 120 110 100 90 80 70 60 50 40 30 20 10 0 -10

1H

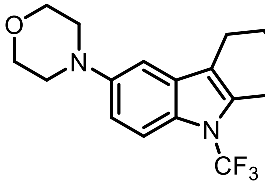

26

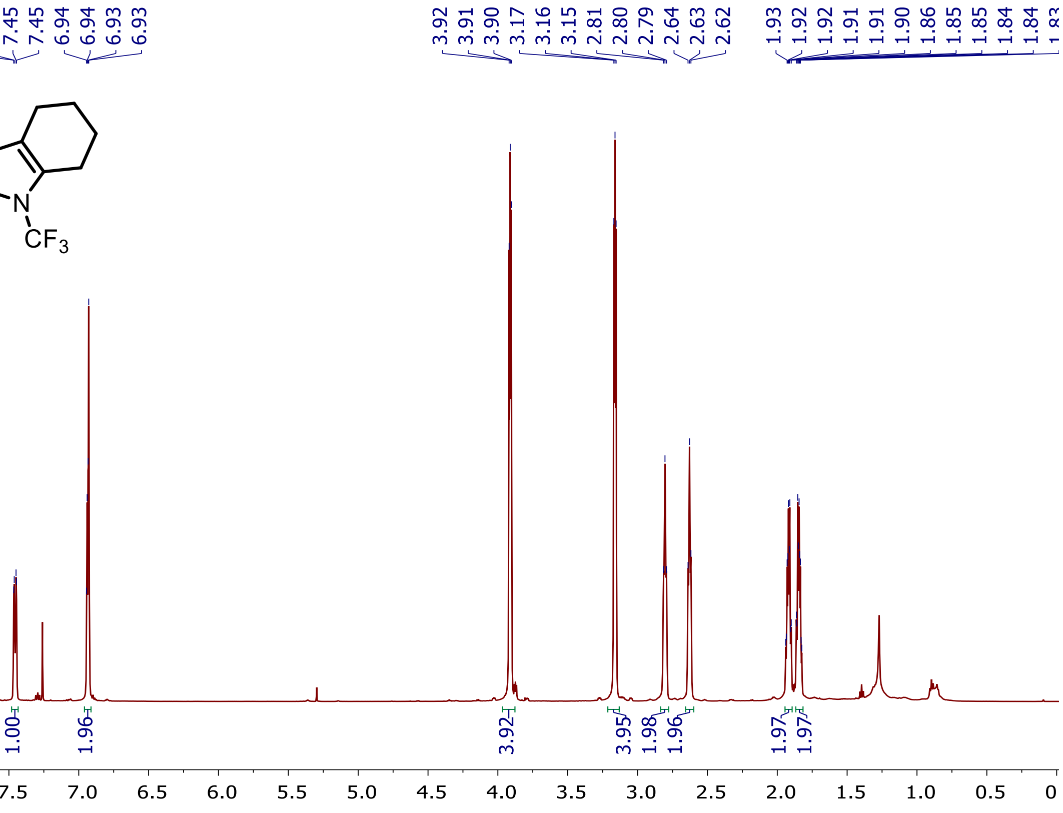

7.47
7.46
7.45
7.45
6.94
6.94
6.93
6.93
3.92
3.91
3.90
3.17
3.16
3.15
2.81
2.80
2.79
2.64
2.63
2.62
1.93
1.92
1.92
1.91
1.91
1.90
1.86
1.85
1.85
1.84
1.84
1.00
1.96
3.92
3.95
1.98
1.96
1.97
1.97
8.5 8.0 7.5 7.0 6.5 6.0 5.5 5.0 4.5 4.0 3.5 3.0 2.5 2.0 1.5 1.0 0.5 0

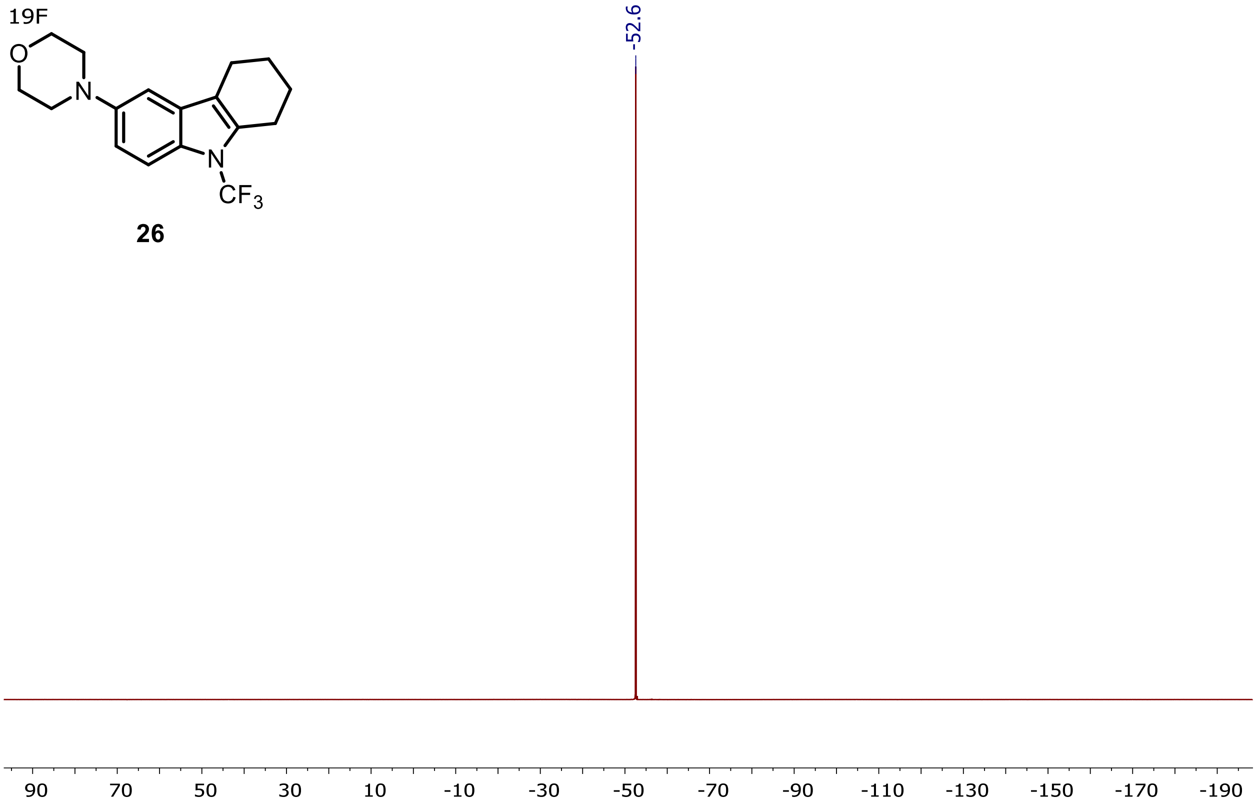
19F
26
-52.6

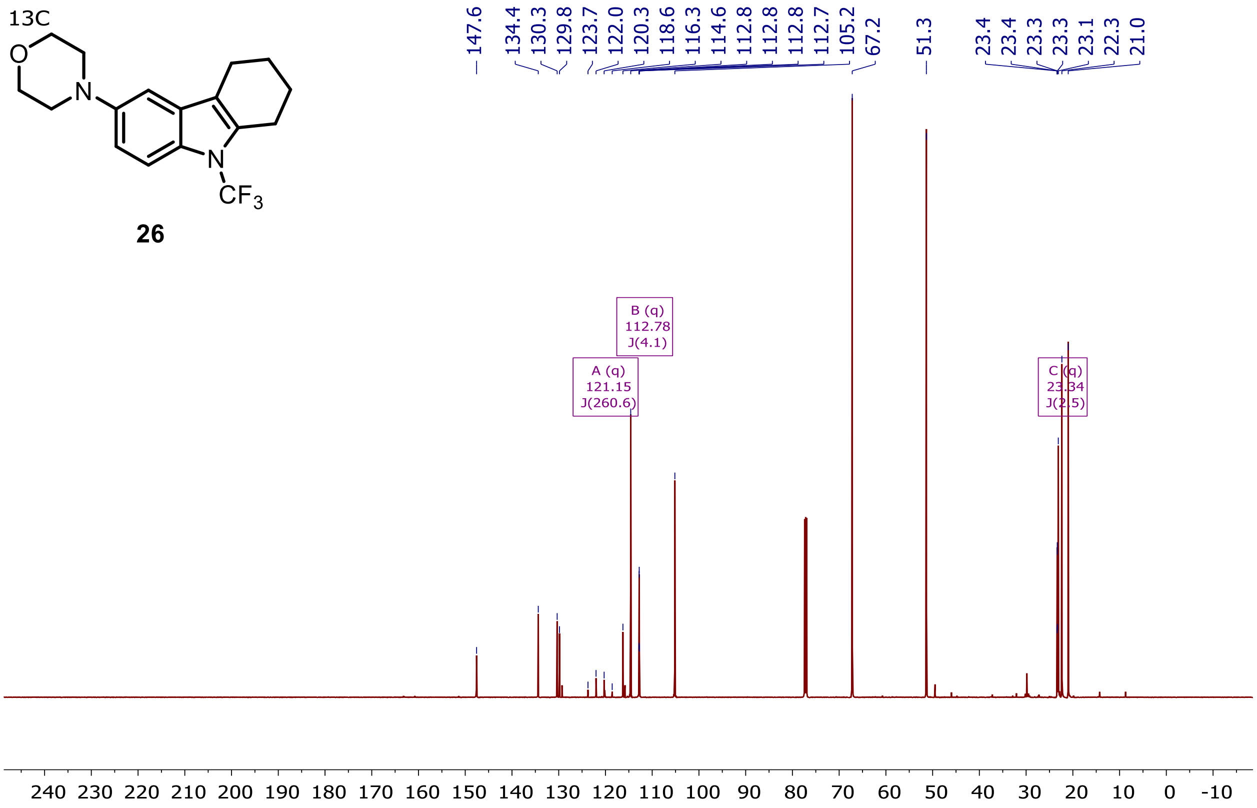
13C
26
147.6
134.4
130.3
129.8
123.7
122.0
120.3
118.6
116.3
114.6
112.8
112.8
112.8
112.7
105.2
67.2
51.3
23.4
23.4
23.3
23.3
23.1
22.3
21.0
B (q)
112.78
J(4.1)
A (q)
121.15
J(260.6)
C (q)
23.34
J(2.5)

N-Substituted Hydrazines-CF_3

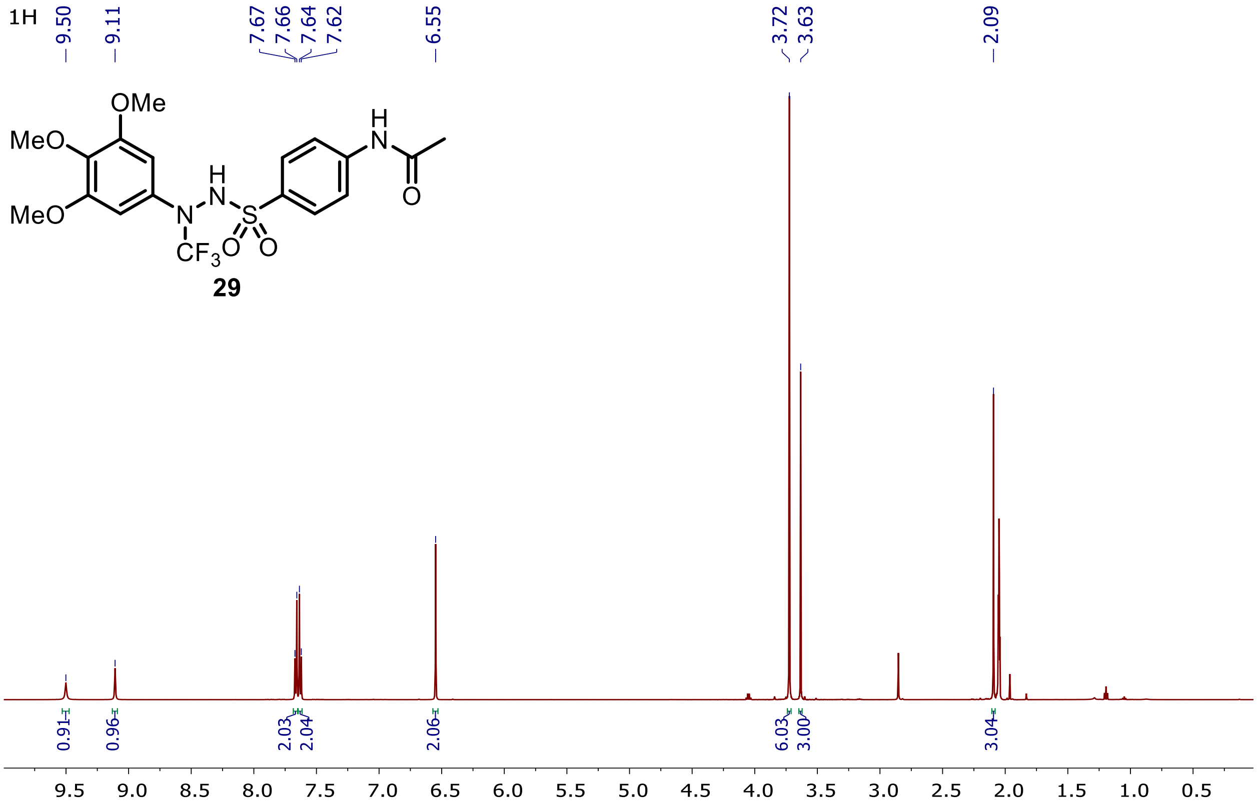

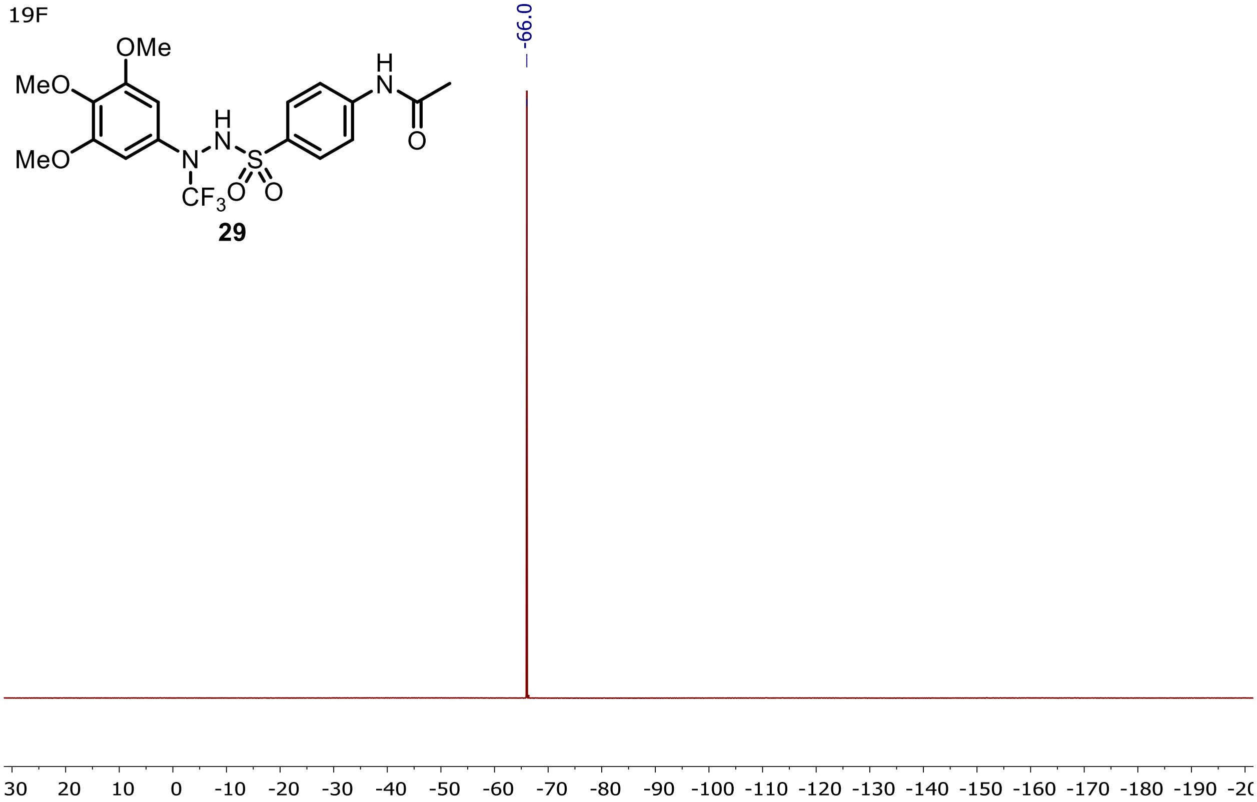

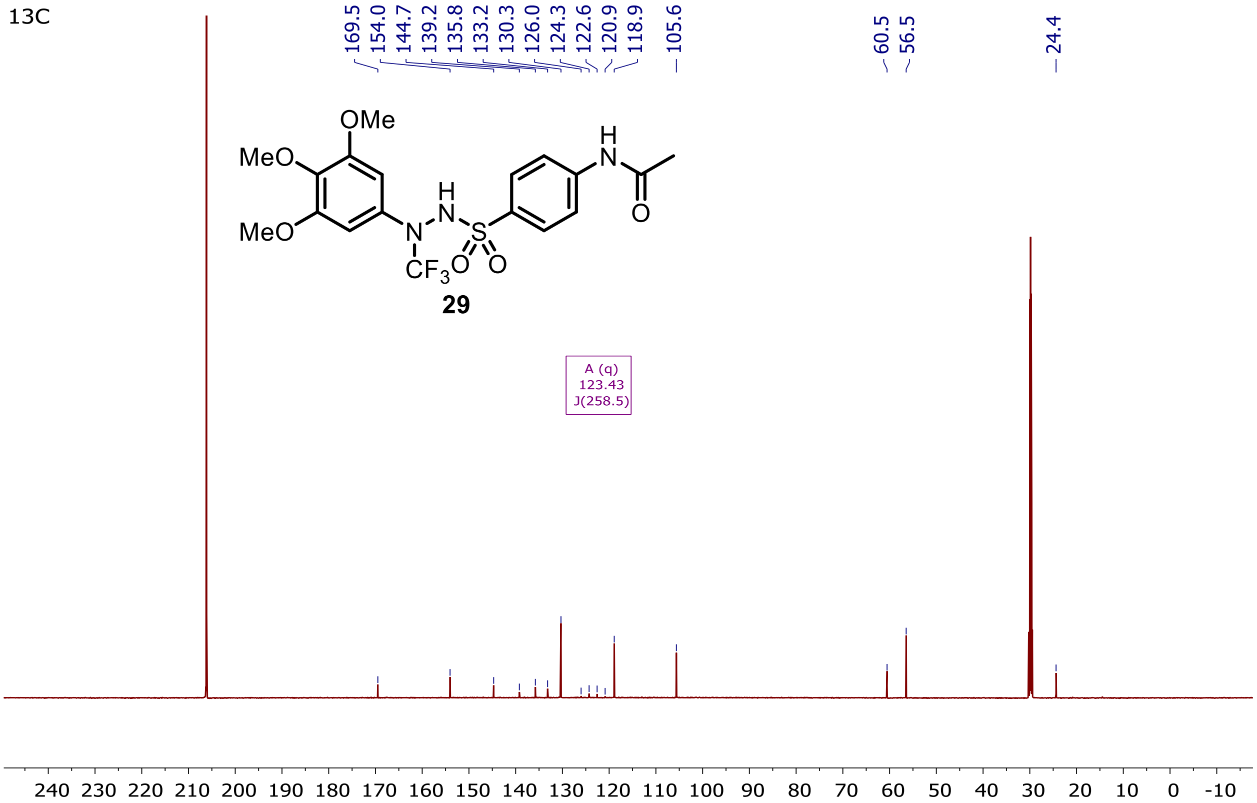
13C
169.5
154.0
144.7
139.2
135.8
133.2
130.3
126.0
124.3
122.6
120.9
118.9
105.6
60.5
56.5
24.4
OMe
MeO
MeO
H
N
N
S
O
O
CF3
29
A (q)
123.43
J(258.5)
240 230 220 210 200 190 180 170 160 150 140 130 120 110 100 90 80 70 60 50 40 30 20 10 0 -10

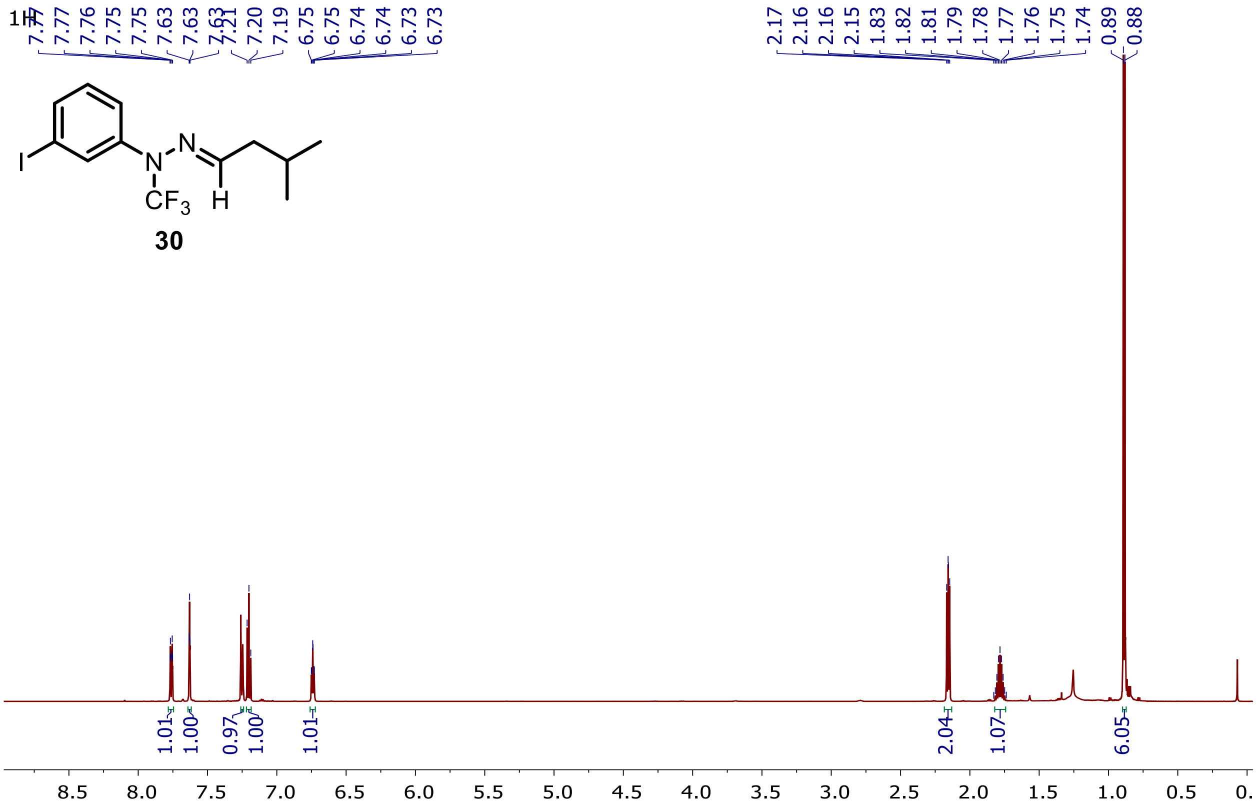
1H
7.77
7.77
7.76
7.75
7.75
7.63
7.63
7.21
7.20
7.19
6.75
6.75
6.74
6.74
6.73
6.73
2.17
2.16
2.16
2.15
1.83
1.82
1.81
1.79
1.78
1.77
1.76
1.75
1.74
0.89
0.88
I
N
N
CF3
H
30
1.01
1.00
0.97
1.00
1.01
2.04
1.07
6.05
8.5 8.0 7.5 7.0 6.5 6.0 5.5 5.0 4.5 4.0 3.5 3.0 2.5 2.0 1.5 1.0 0.5 0.

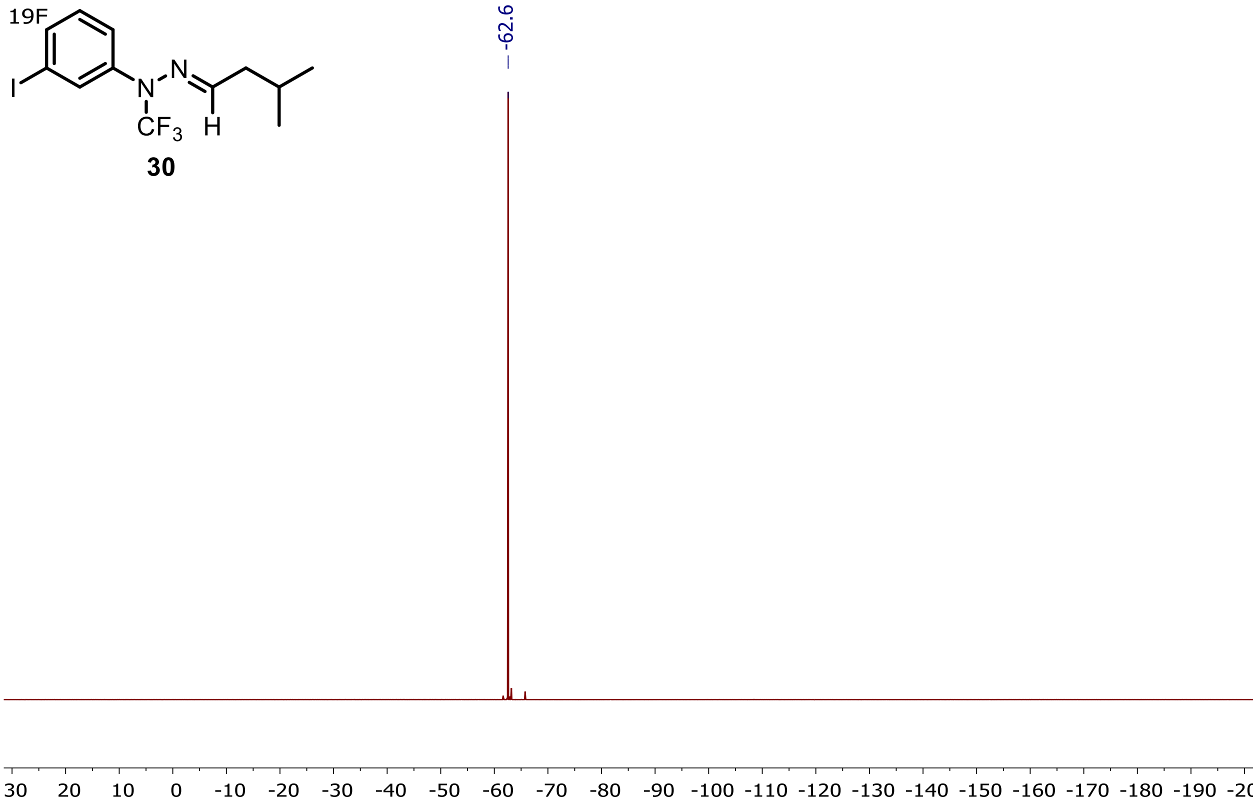
19F
I
N
N
CF3
H
30
-62.6
30 20 10 0 -10 -20 -30 -40 -50 -60 -70 -80 -90 -100 -110 -120 -130 -140 -150 -160 -170 -180 -190 -2(

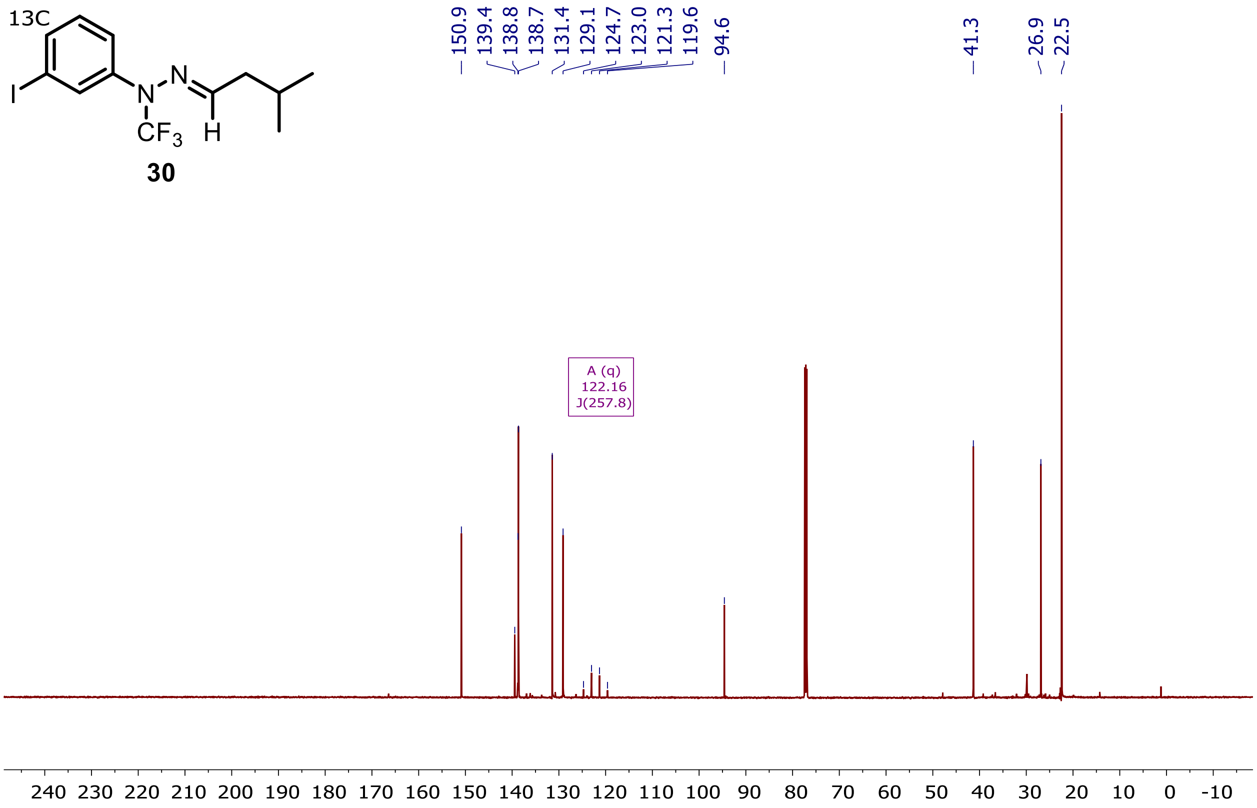
13C
I
N
N
CF3
H
30
150.9
139.4
138.8
138.7
131.4
129.1
124.7
123.0
121.3
119.6
94.6
41.3
26.9
22.5
A (q)
122.16
J(257.8)
240 230 220 210 200 190 180 170 160 150 140 130 120 110 100 90 80 70 60 50 40 30 20 10 0 -10

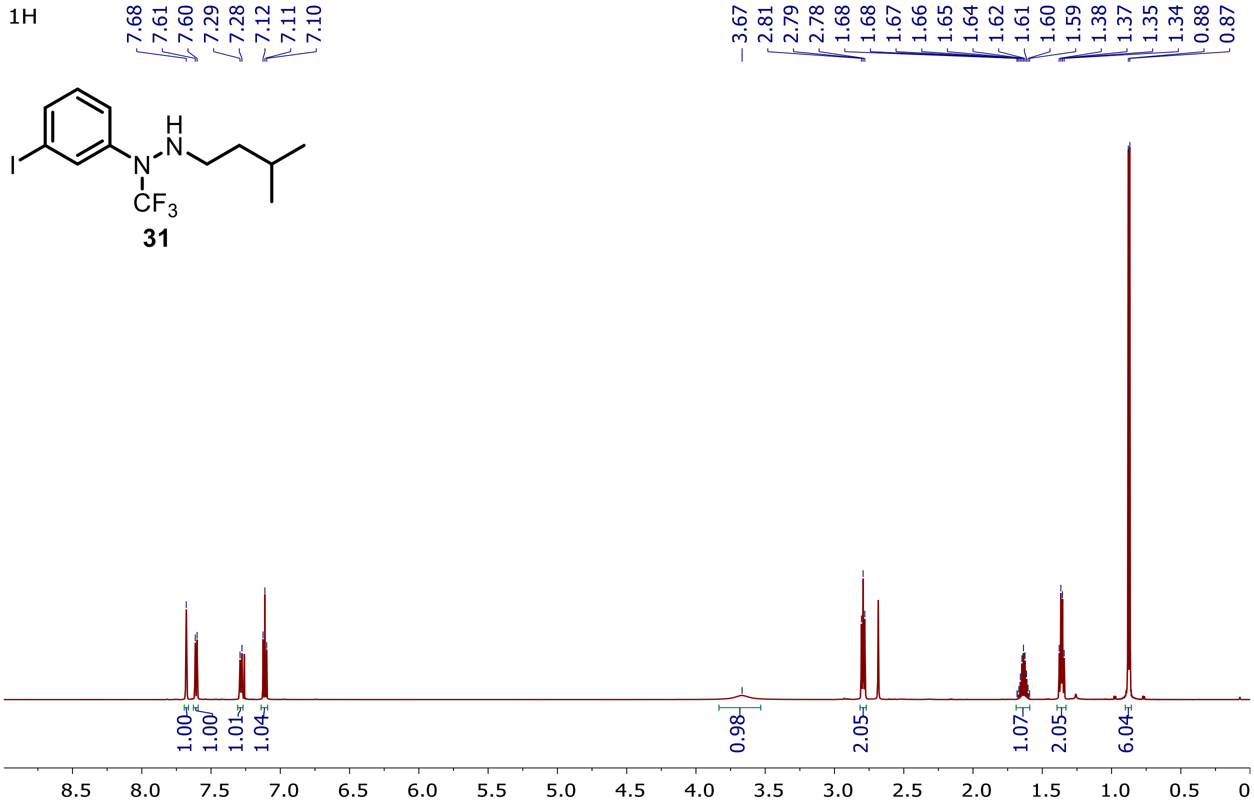

1H
31
CF3
7.68 7.61 7.60 7.29 7.28 7.12 7.11 7.10
3.67 2.81 2.79 2.78 1.68 1.68 1.67 1.66 1.65 1.64 1.62 1.61 1.60 1.59 1.38 1.37 1.35 1.34 0.88 0.87
1.00 1.00 1.01 1.04 0.98 2.05 1.07 2.05 6.04
8.5 8.0 7.5 7.0 6.5 6.0 5.5 5.0 4.5 4.0 3.5 3.0 2.5 2.0 1.5 1.0 0.5 0

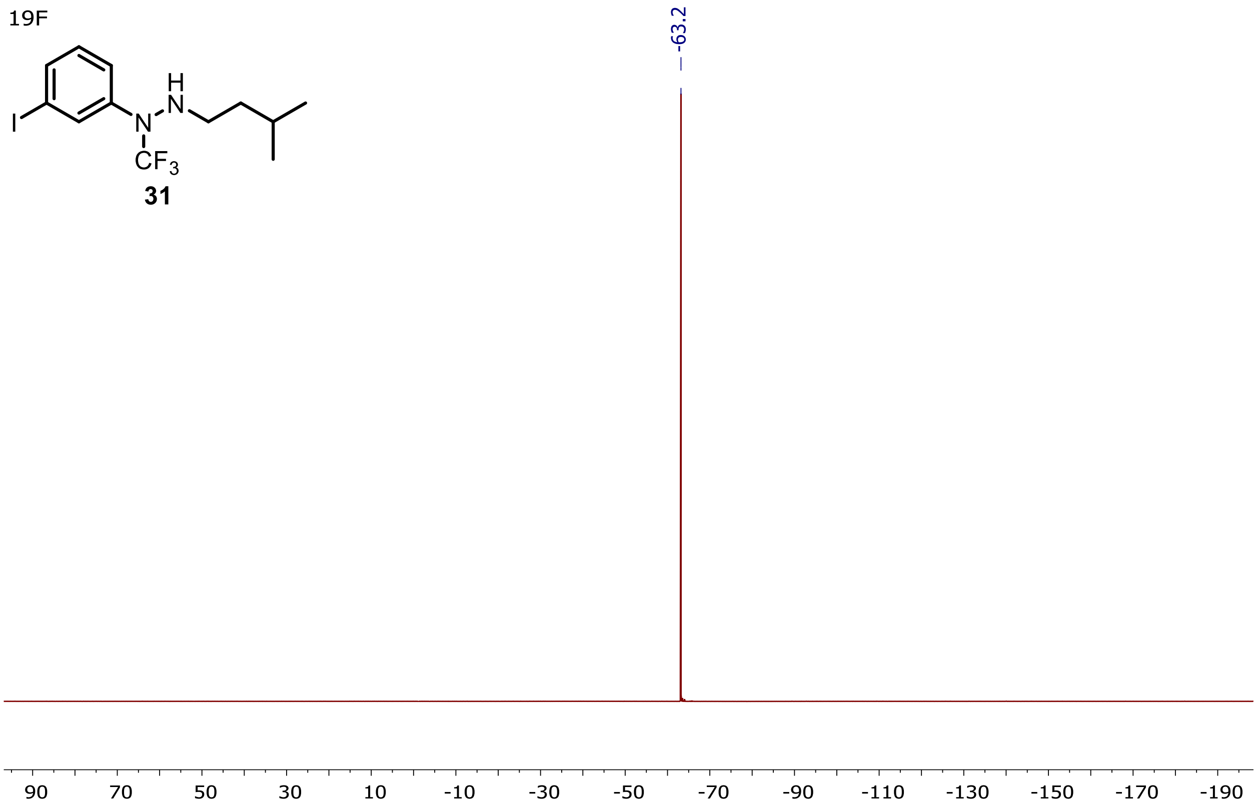

19F
31
CF3
-63.2
90 70 50 30 10 -10 -30 -50 -70 -90 -110 -130 -150 -170 -190

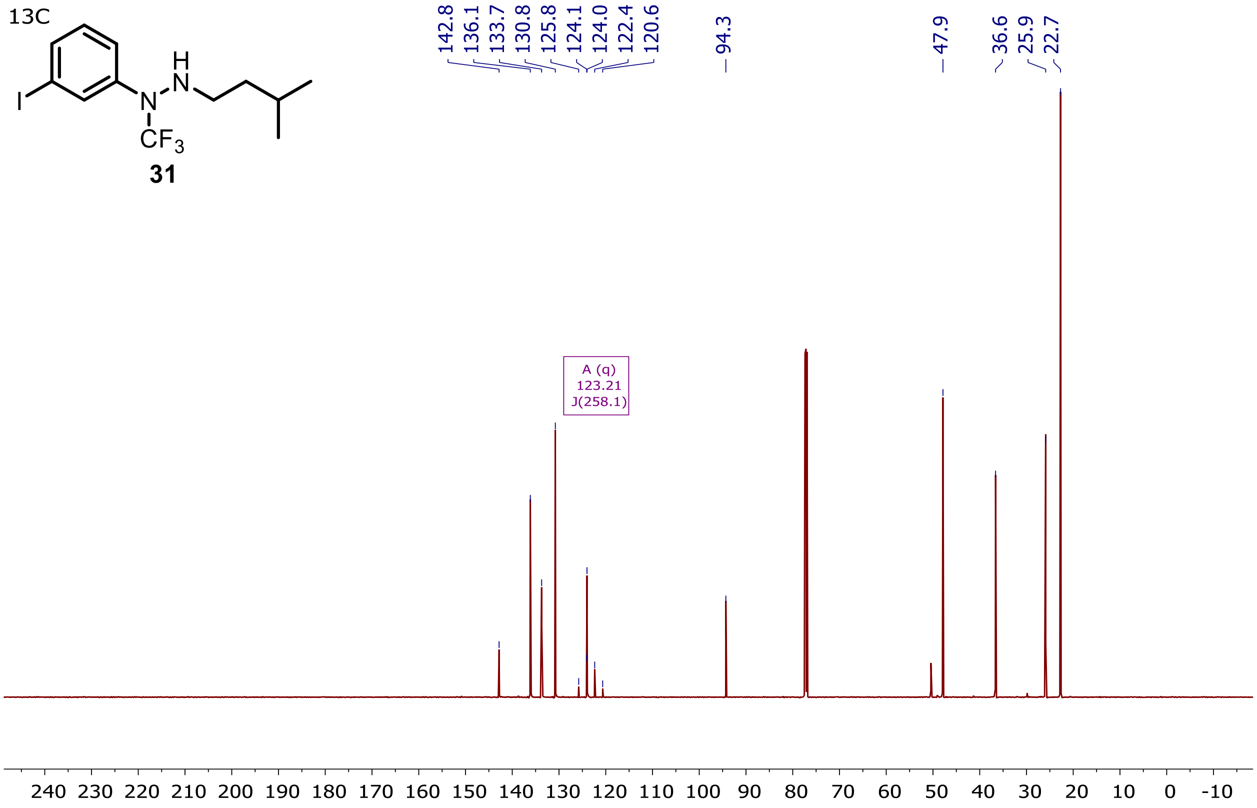
13C
31
142.8
136.1
133.7
130.8
125.8
124.1
124.0
122.4
120.6
94.3
47.9
36.6
25.9
22.7
A (q)
123.21
J(258.1)
240 230 220 210 200 190 180 170 160 150 140 130 120 110 100 90 80 70 60 50 40 30 20 10 0 -10

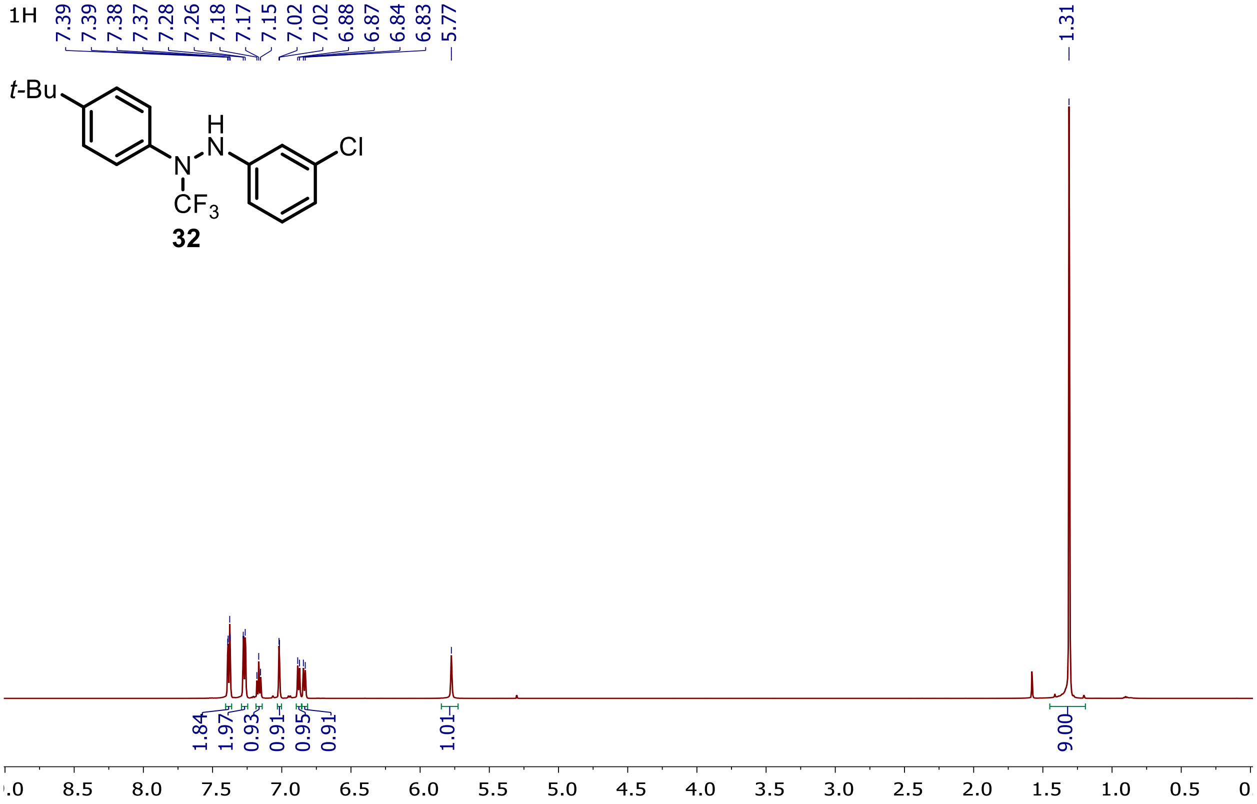
1H
t-Bu
32
7.39
7.39
7.38
7.37
7.28
7.26
7.18
7.17
7.15
7.02
7.02
6.88
6.87
6.84
6.83
5.77
1.31
1.84
1.97
0.93
0.91
0.95
0.91
1.01
9.00
8.5 8.0 7.5 7.0 6.5 6.0 5.5 5.0 4.5 4.0 3.5 3.0 2.5 2.0 1.5 1.0 0.5 0

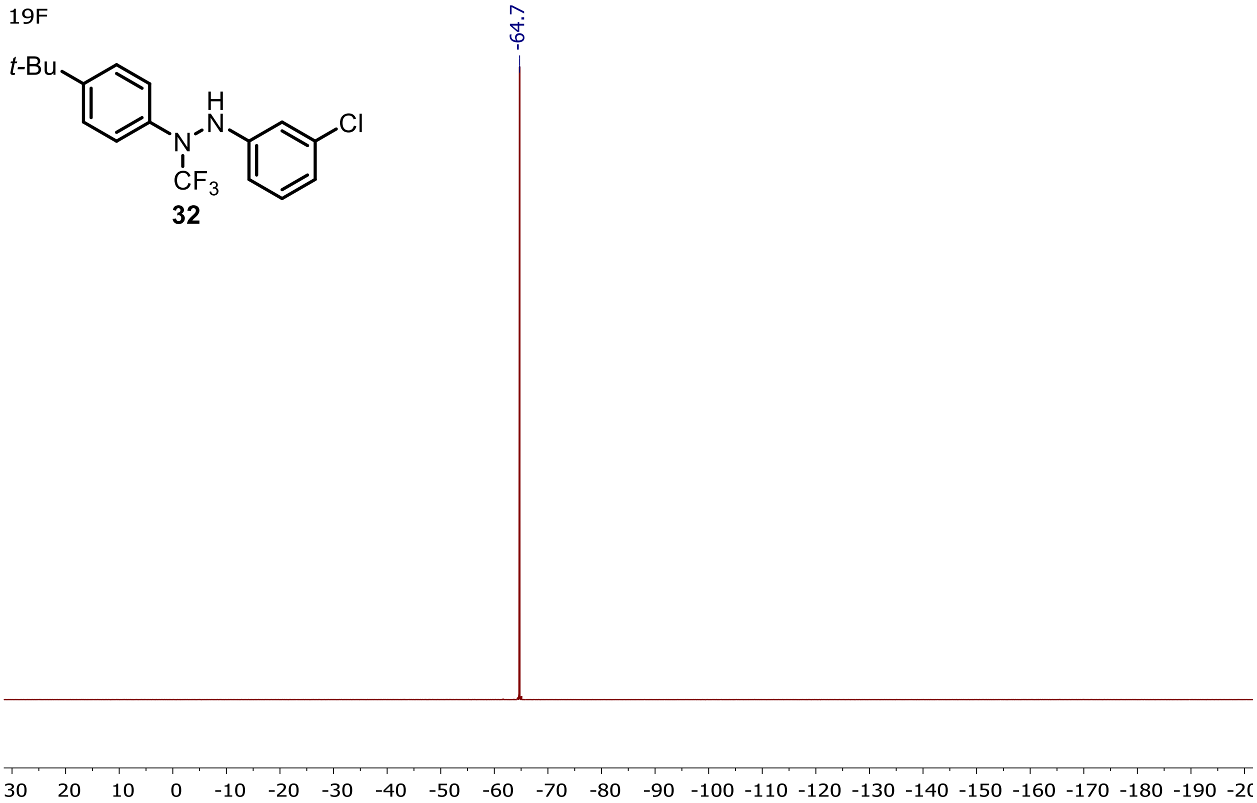
19F
t-Bu
H
N
N
Cl
CF3
32
-64.7
30 20 10 0 -10 -20 -30 -40 -50 -60 -70 -80 -90 -100 -110 -120 -130 -140 -150 -160 -170 -180 -190

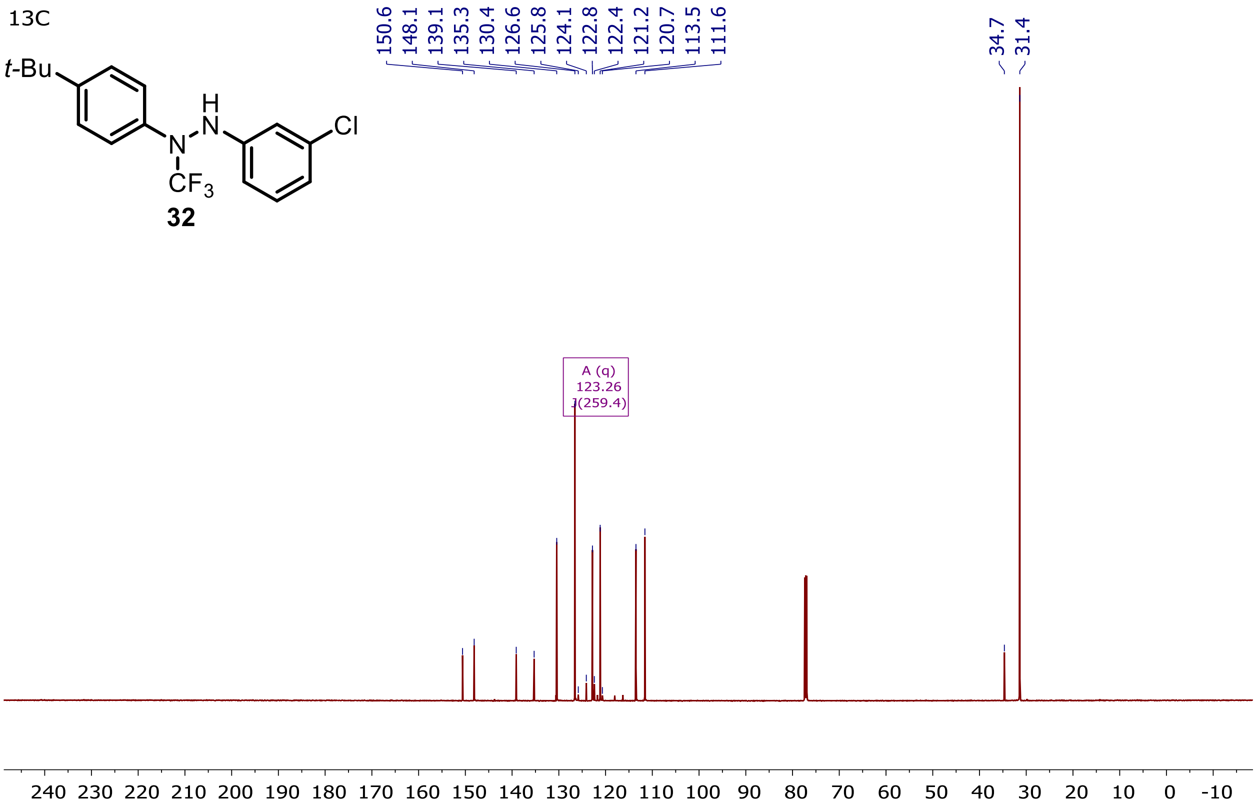
13C
t-Bu
H
N
N
Cl
CF3
32
150.6
148.1
139.1
135.3
130.4
126.6
125.8
124.1
122.8
122.4
121.2
120.7
113.5
111.6
34.7
31.4
A (q)
123.26
J(259.4)
240 230 220 210 200 190 180 170 160 150 140 130 120 110 100 90 80 70 60 50 40 30 20 10 0 -10

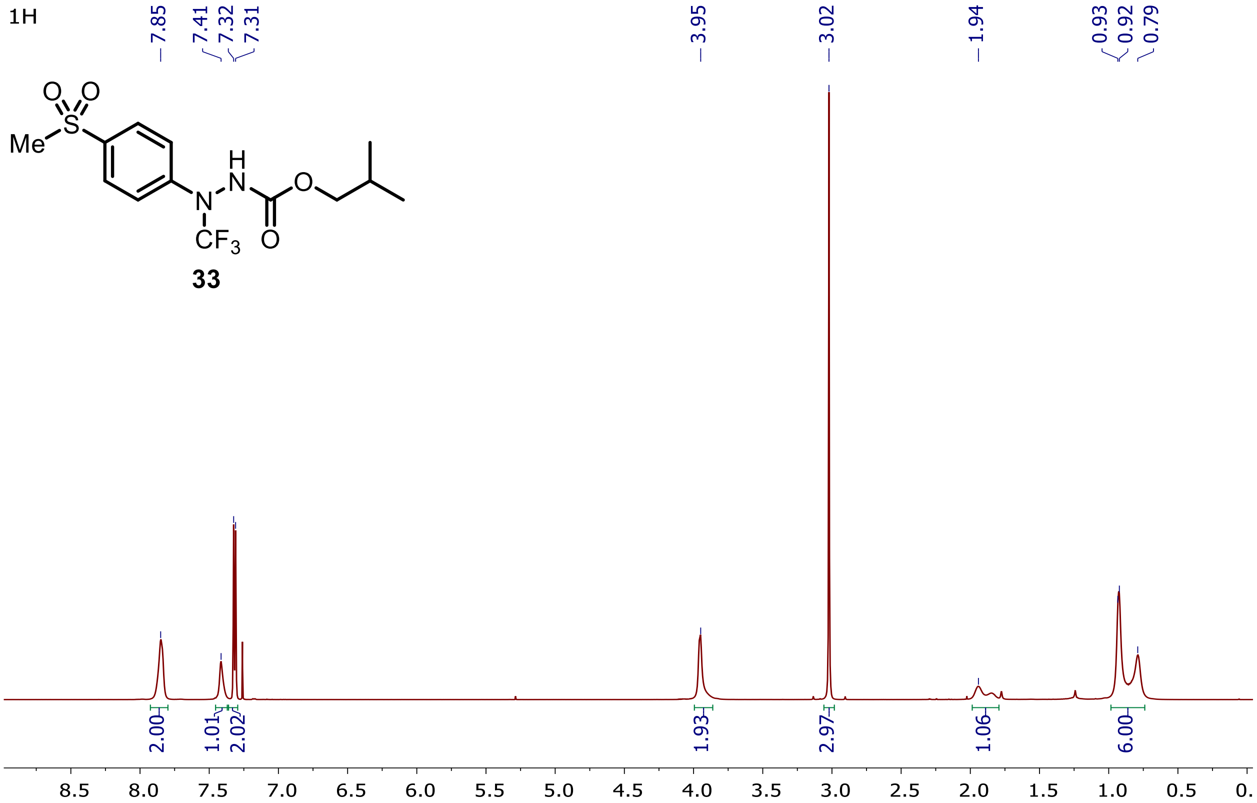
1H
7.85
7.41
7.32
7.31
3.95
3.02
1.94
0.93
0.92
0.79
O
O
S
Me
H
N
N
O
CF3
O
2.00
1.01
2.02
1.93
2.97
1.06
6.00
8.5 8.0 7.5 7.0 6.5 6.0 5.5 5.0 4.5 4.0 3.5 3.0 2.5 2.0 1.5 1.0 0.5 0.

33

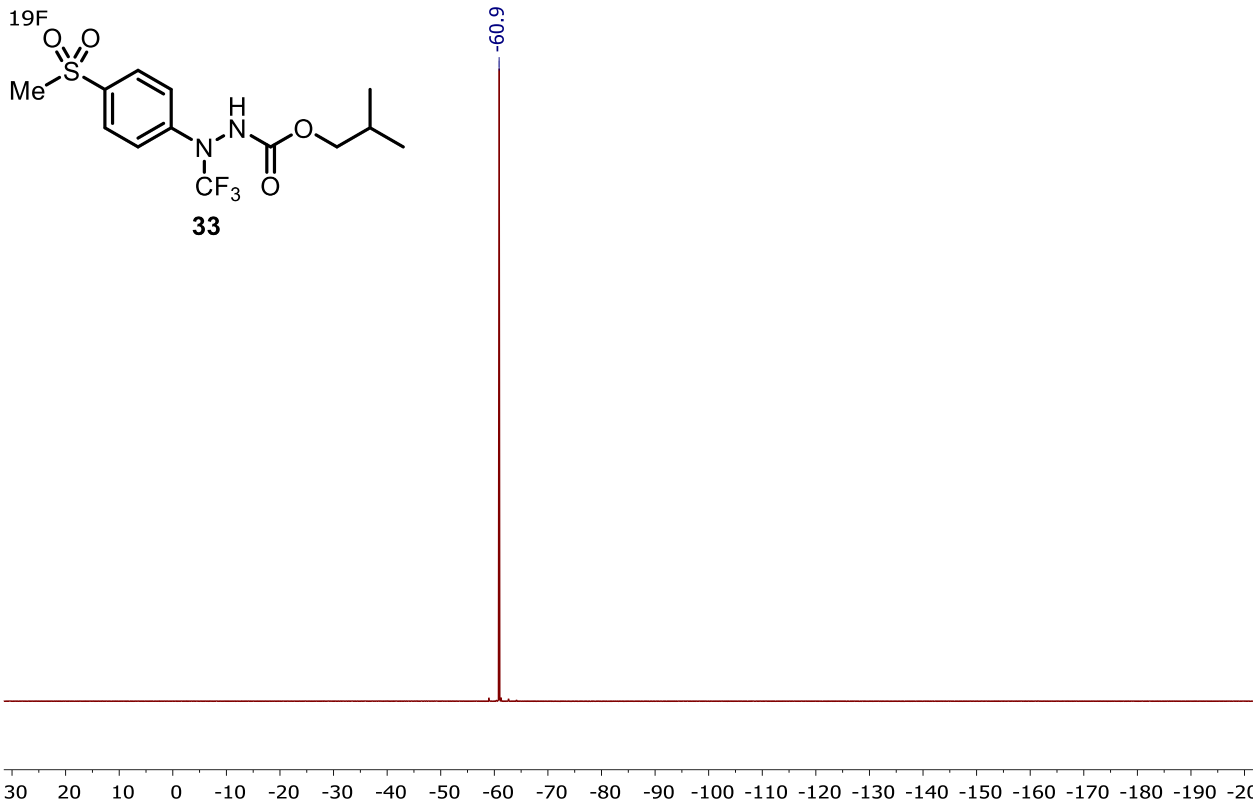
19F
-60.9
O
O
S
Me
H
N
N
O
CF3
O
30 20 10 0 -10 -20 -30 -40 -50 -60 -70 -80 -90 -100 -110 -120 -130 -140 -150 -160 -170 -180 -190 -20

33

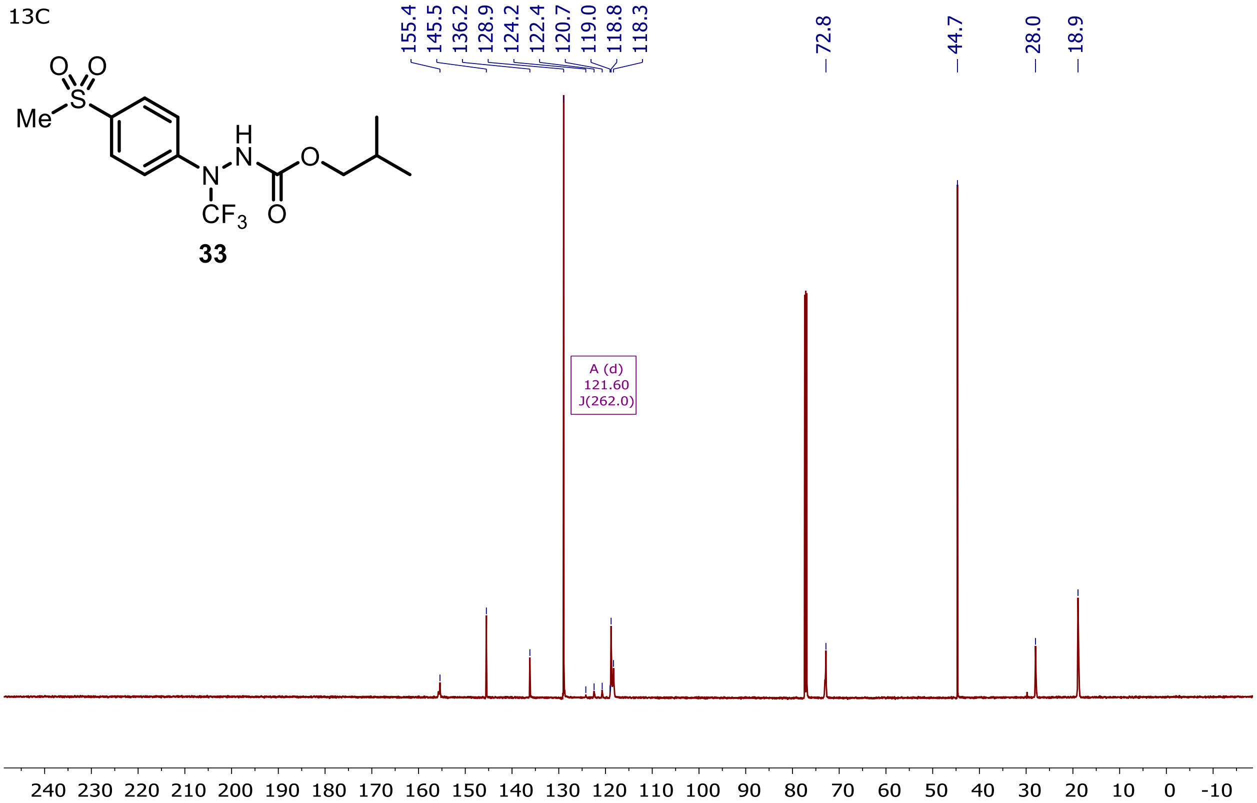

13C
155.4
145.5
136.2
128.9
124.2
122.4
120.7
119.0
118.8
118.3
72.8
44.7
28.0
18.9
Me
S
O
O
H
N
N
O
O
CF3
33
A (d)
121.60
J(262.0)
240 230 220 210 200 190 180 170 160 150 140 130 120 110 100 90 80 70 60 50 40 30 20 10 0 -10

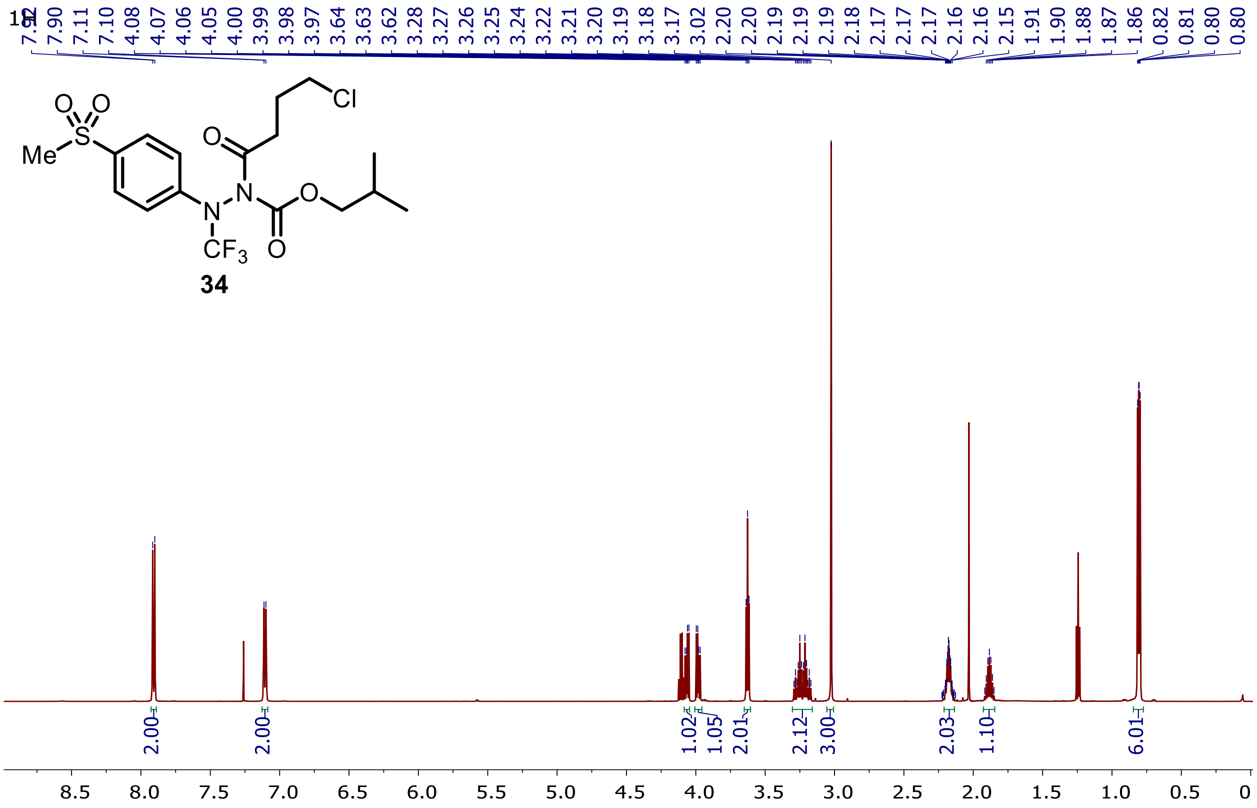

1H
7.92
7.90
7.11
7.10
4.08
4.07
4.06
4.05
4.00
3.99
3.98
3.97
3.64
3.63
3.62
3.28
3.27
3.26
3.25
3.24
3.22
3.21
3.20
3.19
3.18
3.17
3.02
2.20
2.20
2.19
2.19
2.19
2.18
2.17
2.17
2.17
2.16
2.16
2.15
1.91
1.90
1.88
1.87
1.86
0.82
0.81
0.80
0.80
Cl
O
Me
S
O
O
N
N
O
O
CF3
34
2.00
2.00
1.02
1.05
2.01
2.12
3.00
2.03
1.10
6.01
8.5 8.0 7.5 7.0 6.5 6.0 5.5 5.0 4.5 4.0 3.5 3.0 2.5 2.0 1.5 1.0 0.5 0

19F

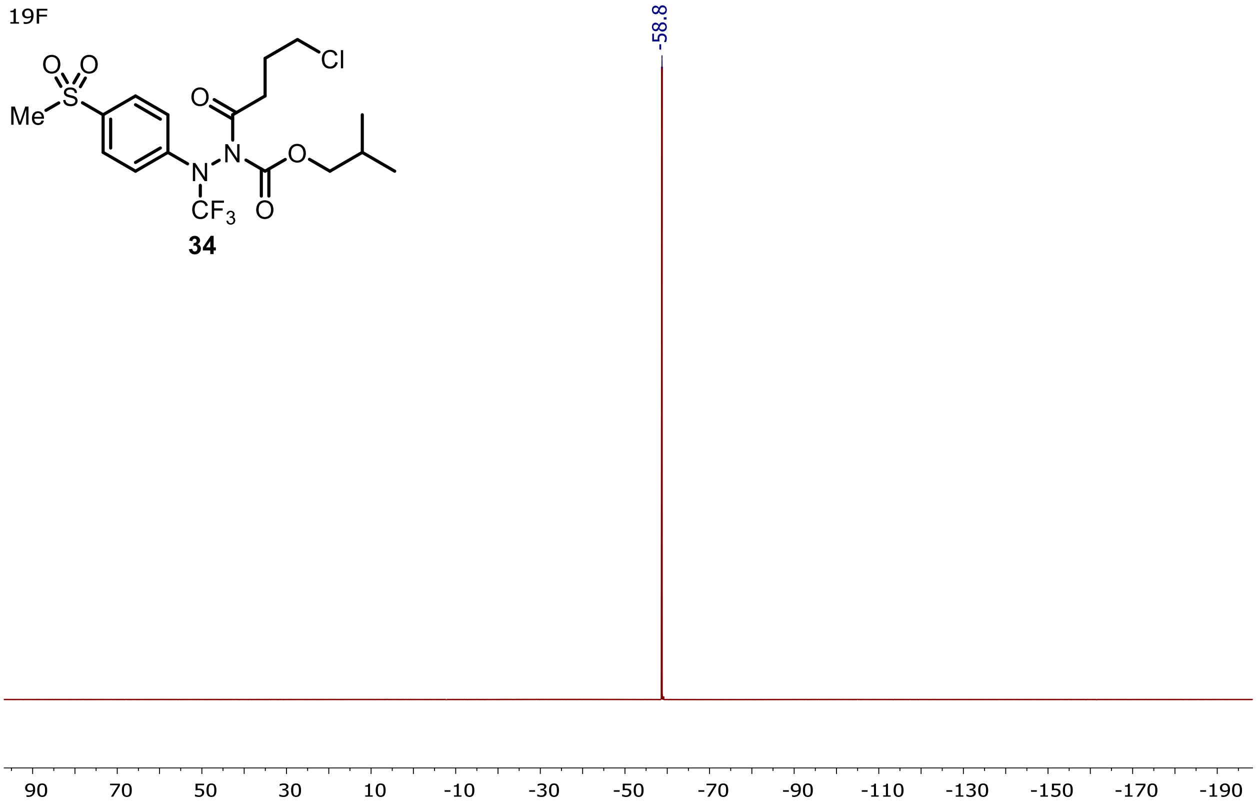

-58.8
O O
S
Me
O
Cl
N
N
O
CF3
O
34
90 70 50 30 10 -10 -30 -50 -70 -90 -110 -130 -150 -170 -190

13C

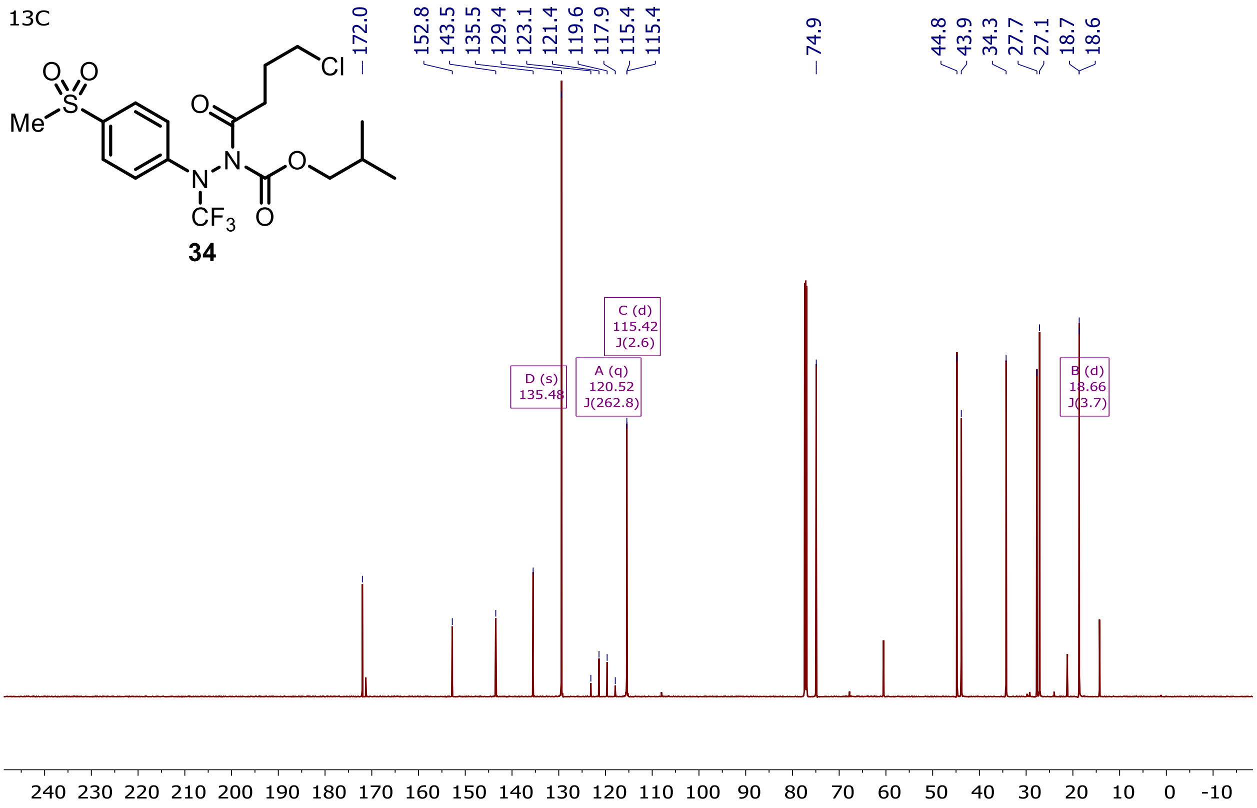

172.0
152.8
143.5
135.5
129.4
123.1
121.4
119.6
117.9
115.4
115.4
74.9
44.8
43.9
34.3
27.7
27.1
18.7
18.6
O O
S
Me
O
Cl
N
N
O
CF3
O
34
C (d)
115.42
J(2.6)
D (s)
135.48
A (q)
120.52
J(262.8)
B (d)
18.66
J(3.7)
240 230 220 210 200 190 180 170 160 150 140 130 120 110 100 90 80 70 60 50 40 30 20 10 0 -10

Carbamic fluorides

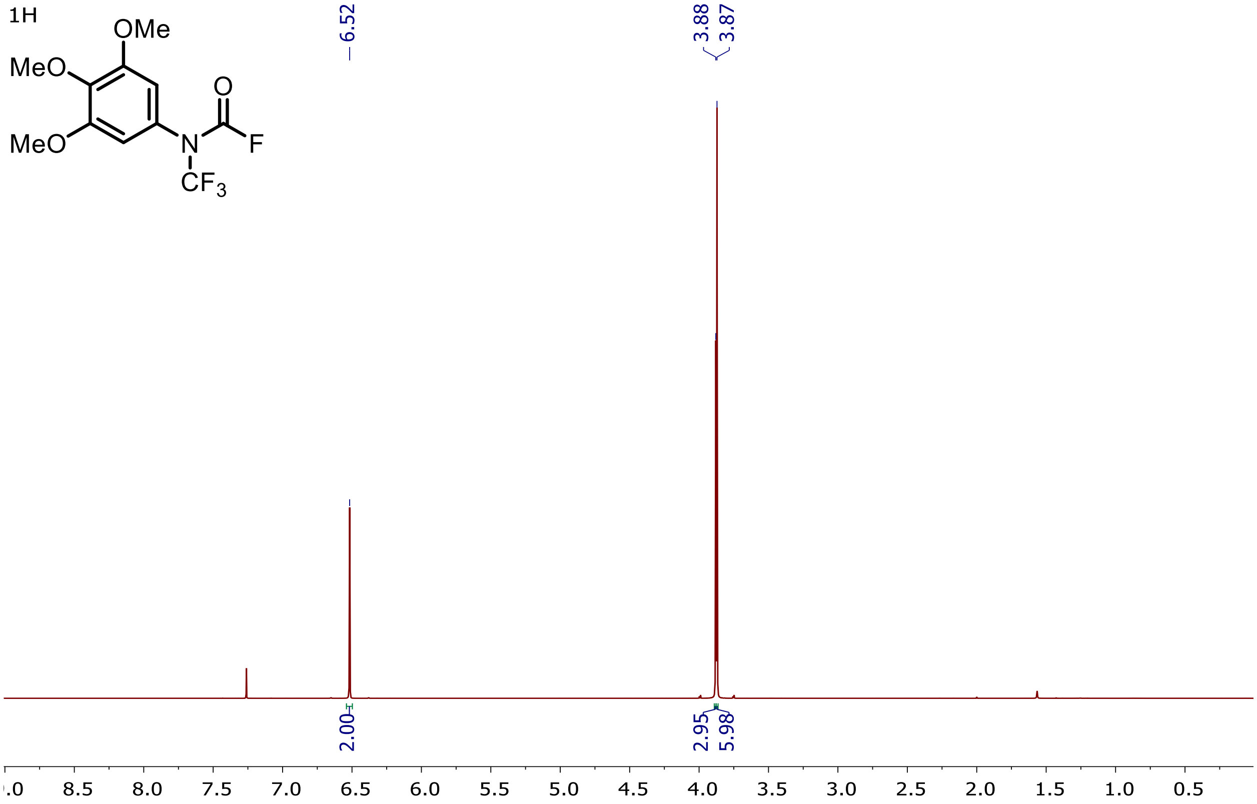

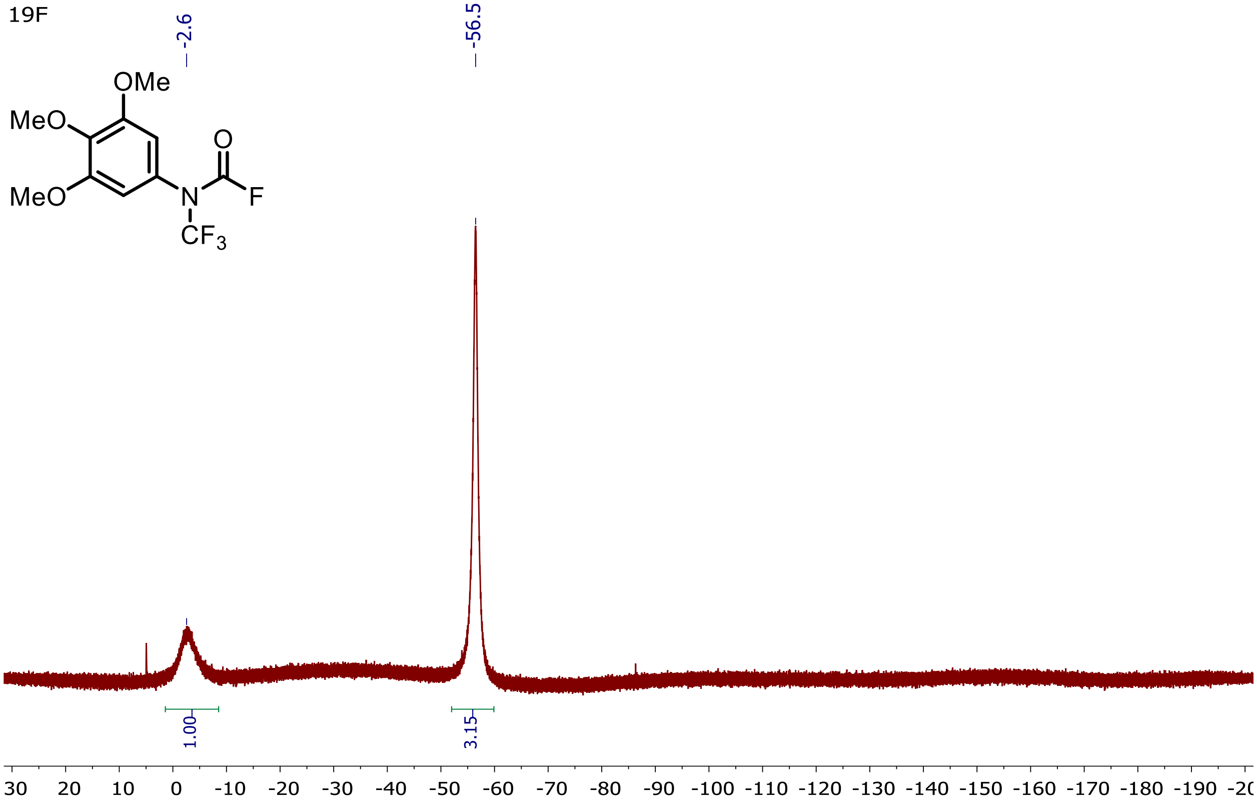

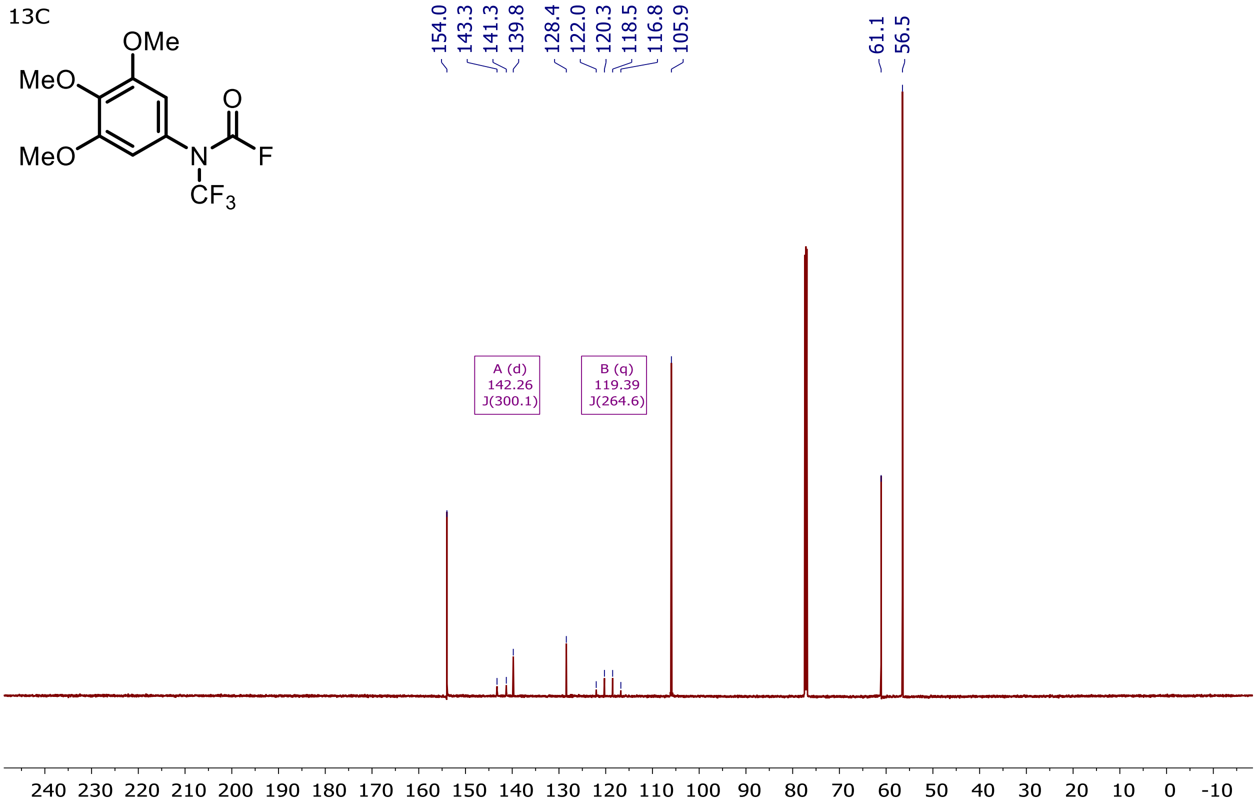
13C
OMe
MeO
MeO
O
N
F
CF3
154.0
143.3
141.3
139.8
128.4
122.0
120.3
118.5
116.8
105.9
61.1
56.5
A (d)
142.26
J(300.1)
B (q)
119.39
J(264.6)
240 230 220 210 200 190 180 170 160 150 140 130 120 110 100 90 80 70 60 50 40 30 20 10 0 -10

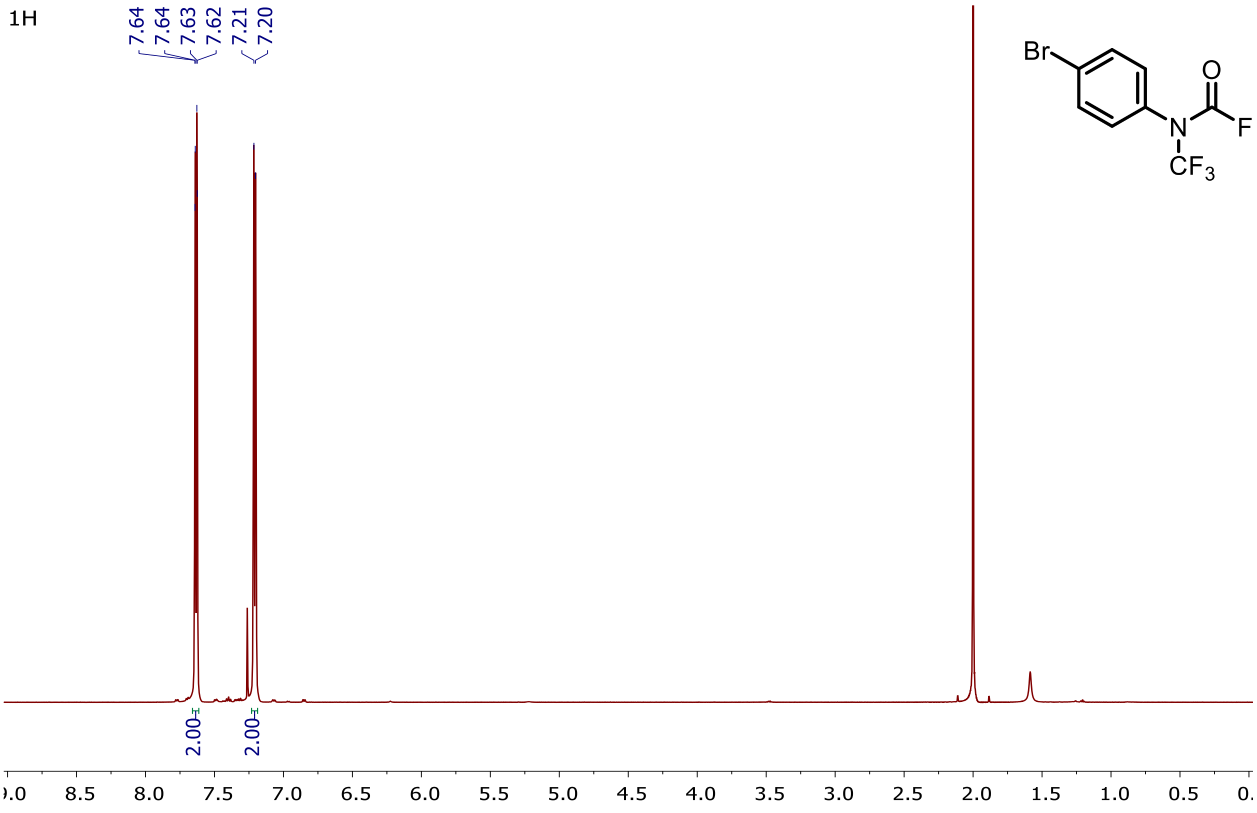
1H
7.64
7.64
7.63
7.62
7.21
7.20
Br
O
N
F
CF3
2.00
2.00
8.5 8.0 7.5 7.0 6.5 6.0 5.5 5.0 4.5 4.0 3.5 3.0 2.5 2.0 1.5 1.0 0.5

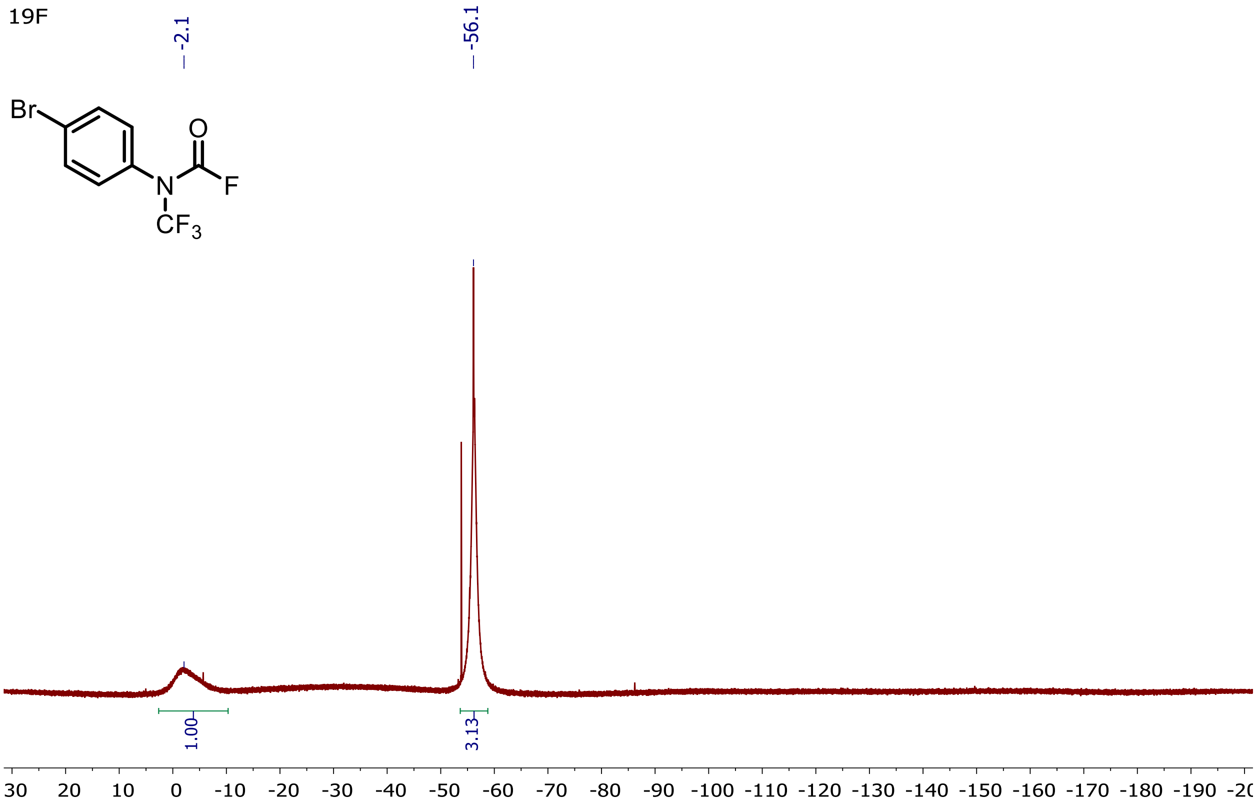

19F
-2.1
-56.1
Br
O
N
F
CF3
1.00
3.13
30 20 10 0 -10 -20 -30 -40 -50 -60 -70 -80 -90 -100 -110 -120 -130 -140 -150 -160 -170 -180 -190

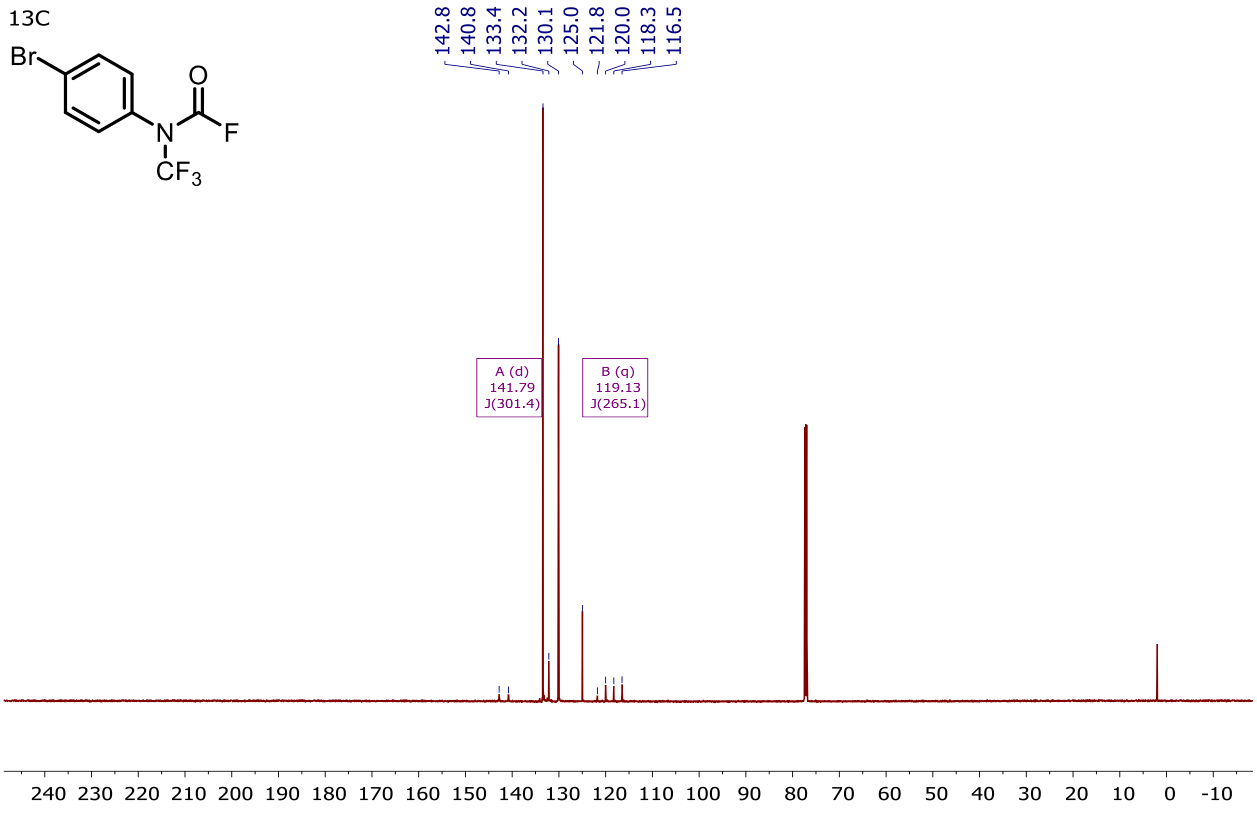

13C
142.8
140.8
133.4
132.2
130.1
125.0
121.8
120.0
118.3
116.5
Br
O
N
F
CF3
A (d)
141.79
J(301.4)
B (q)
119.13
J(265.1)
240 230 220 210 200 190 180 170 160 150 140 130 120 110 100 90 80 70 60 50 40 30 20 10 0 -10

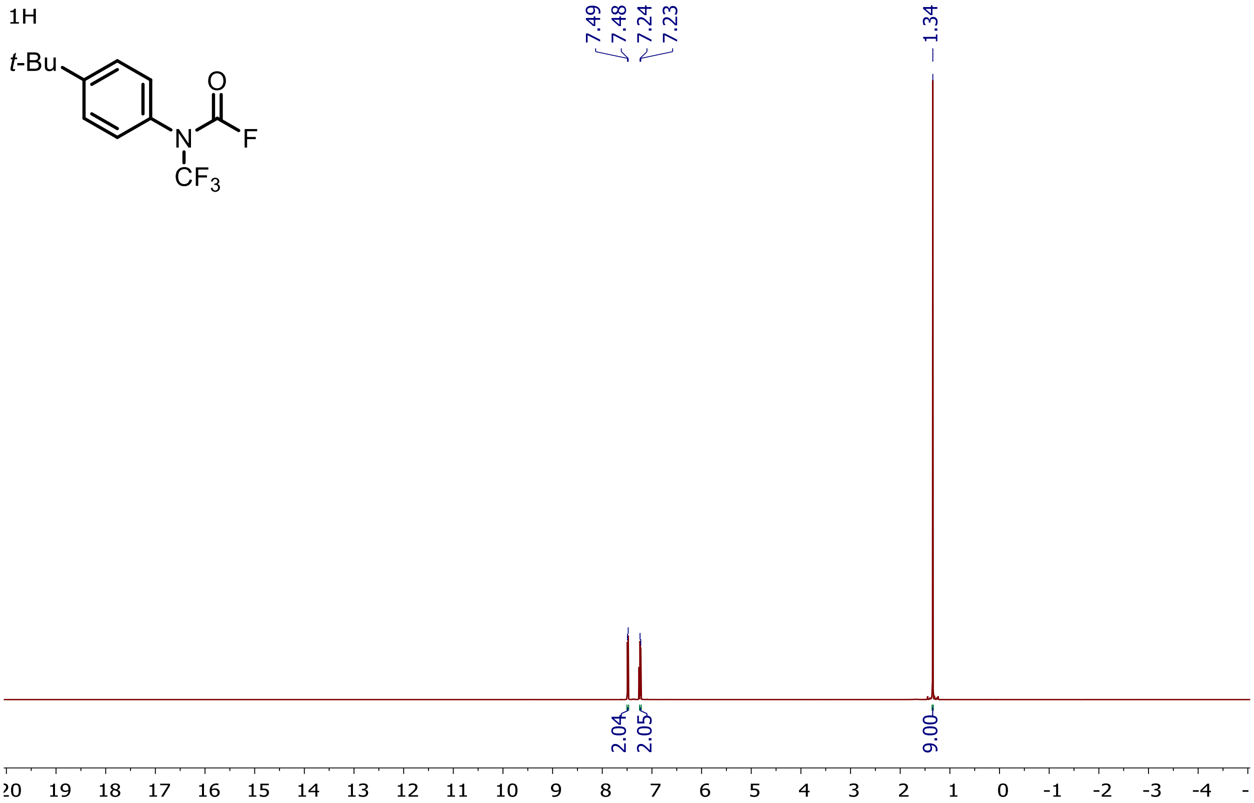
1H
t-Bu
O
F
N
CF3
7.49
7.48
7.24
7.23
1.34
2.04
2.05
9.00
20 19 18 17 16 15 14 13 12 11 10 9 8 7 6 5 4 3 2 1 0 -1 -2 -3 -4

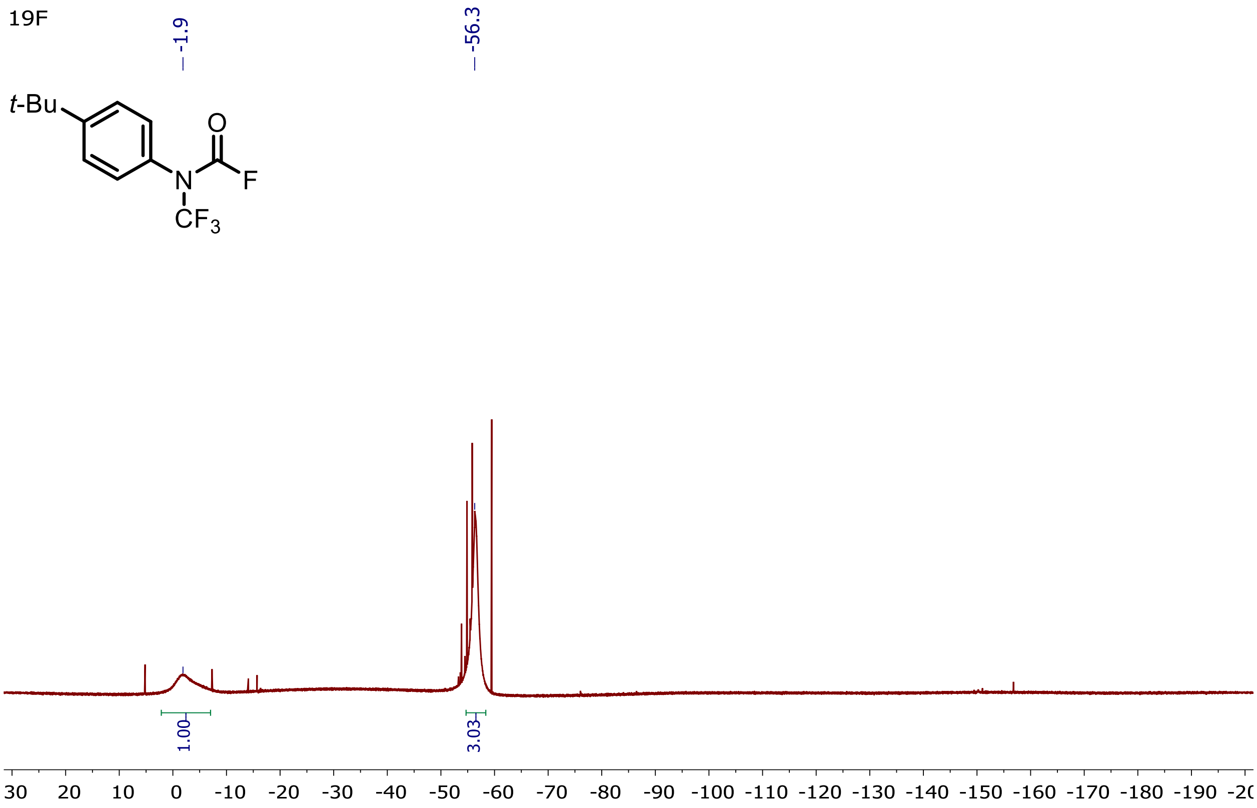
19F
-1.9
-56.3
t-Bu
O
F
N
CF3
1.00
3.03
30 20 10 0 -10 -20 -30 -40 -50 -60 -70 -80 -90 -100 -110 -120 -130 -140 -150 -160 -170 -180 -190

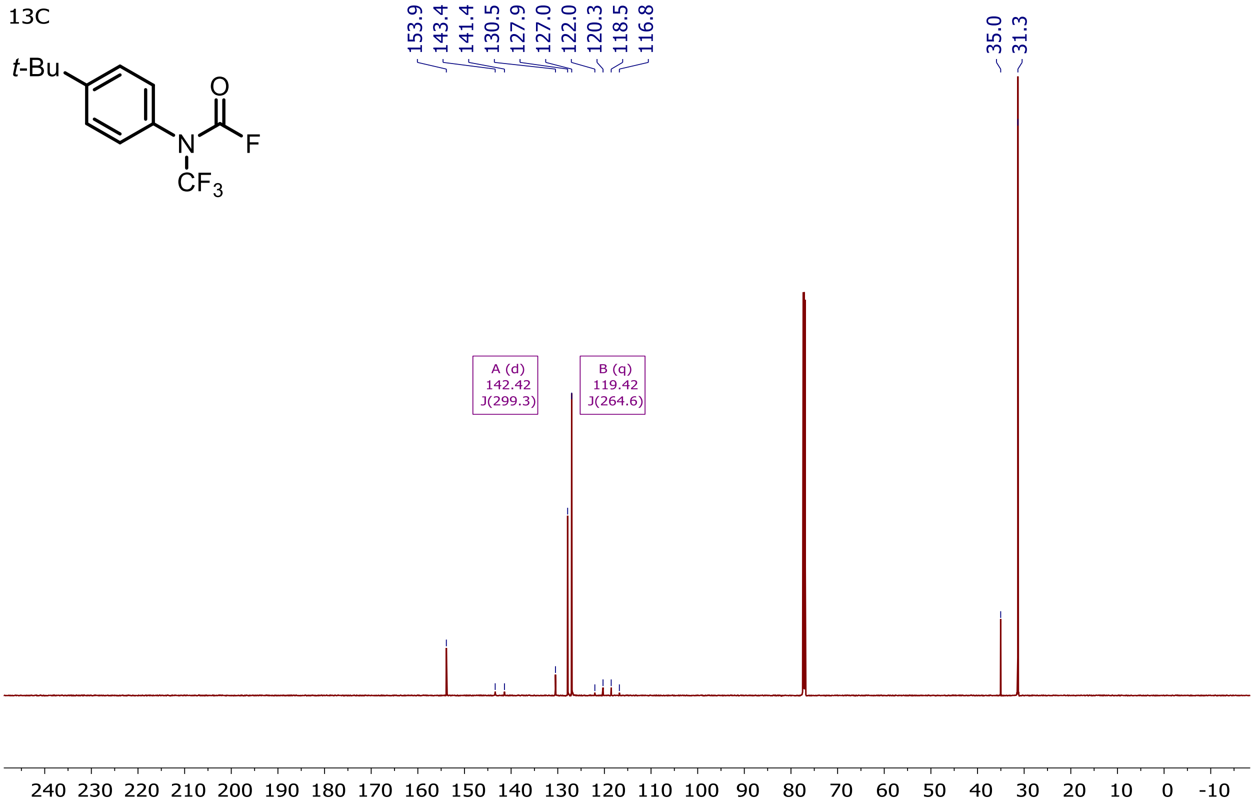
13C
t-Bu
O
N
F
CF3
153.9
143.4
141.4
130.5
127.9
127.0
122.0
120.3
118.5
116.8
35.0
31.3
A (d)
142.42
J(299.3)
B (q)
119.42
J(264.6)
240 230 220 210 200 190 180 170 160 150 140 130 120 110 100 90 80 70 60 50 40 30 20 10 0 -10

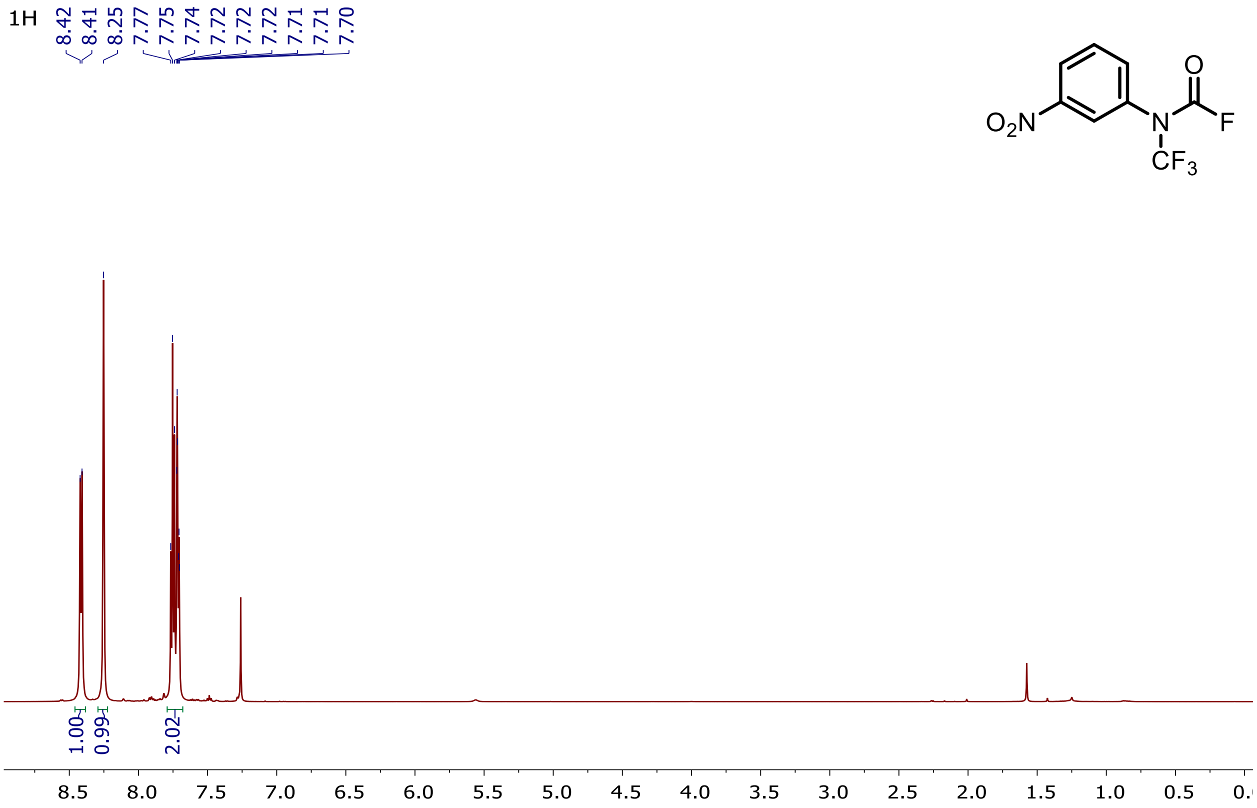
1H
8.42
8.41
8.25
7.77
7.75
7.74
7.72
7.72
7.72
7.71
7.71
7.70
O2N
O
N
F
CF3
1.00
0.99
2.02
8.5 8.0 7.5 7.0 6.5 6.0 5.5 5.0 4.5 4.0 3.5 3.0 2.5 2.0 1.5 1.0 0.5

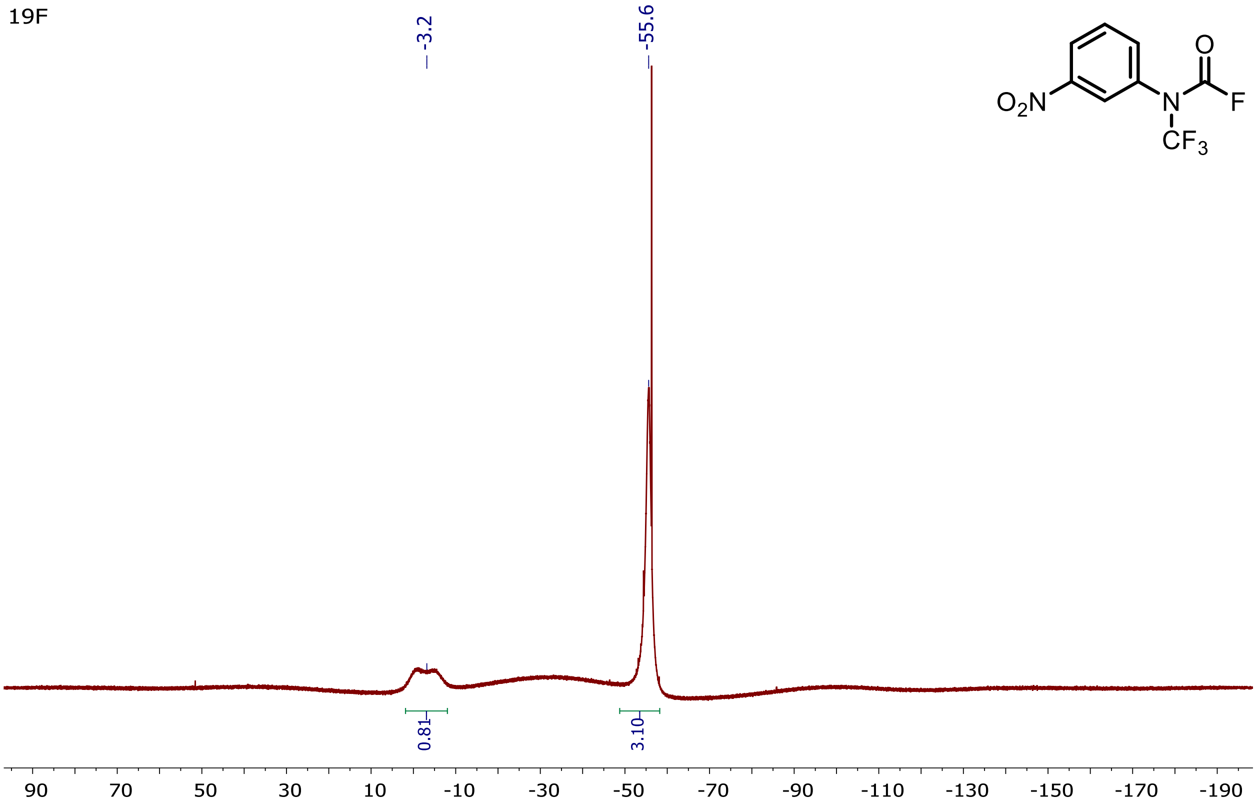

19F
-3.2
-55.6
O2N
N
O
F
CF3
0.81
3.10
90 70 50 30 10 -10 -30 -50 -70 -90 -110 -130 -150 -170 -190

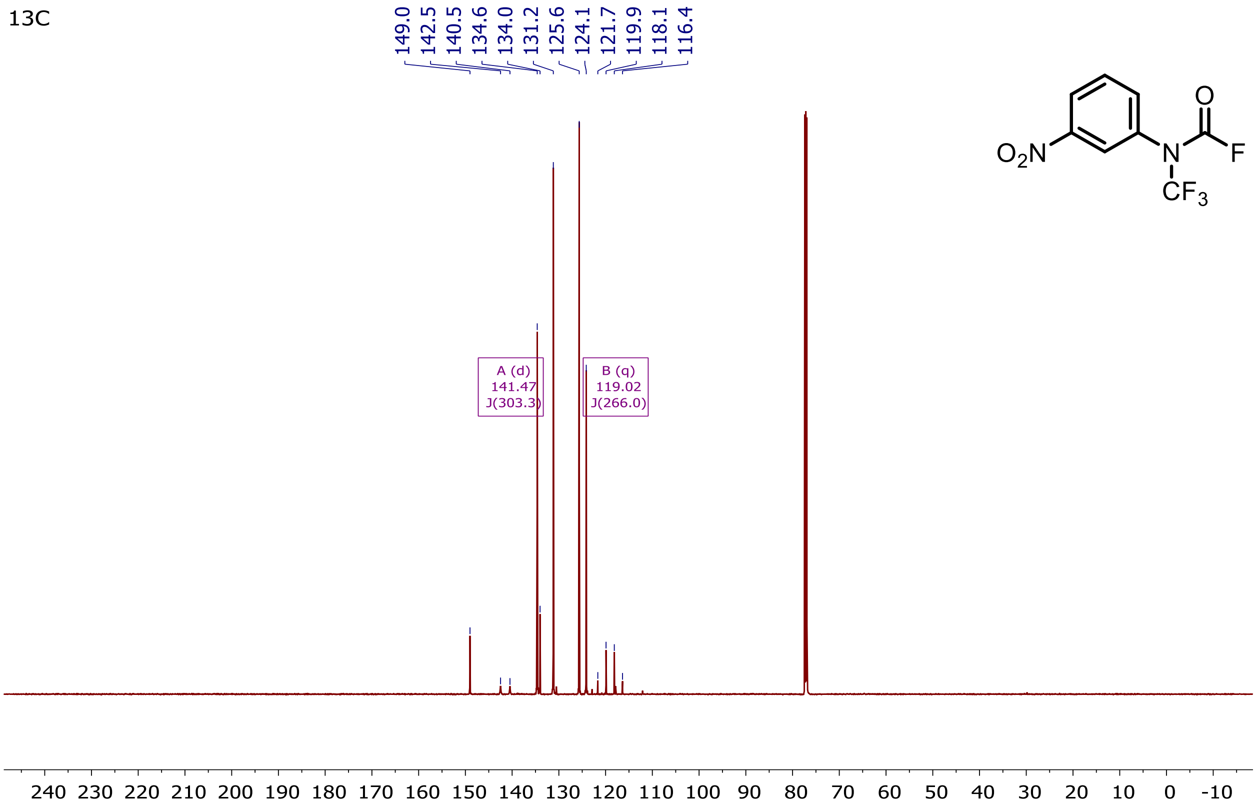

13C
149.0
142.5
140.5
134.6
134.0
131.2
125.6
124.1
121.7
119.9
118.1
116.4
O2N
N
O
F
CF3
A (d)
141.47
J(303.3)
B (q)
119.02
J(266.0)
240 230 220 210 200 190 180 170 160 150 140 130 120 110 100 90 80 70 60 50 40 30 20 10 0 -10

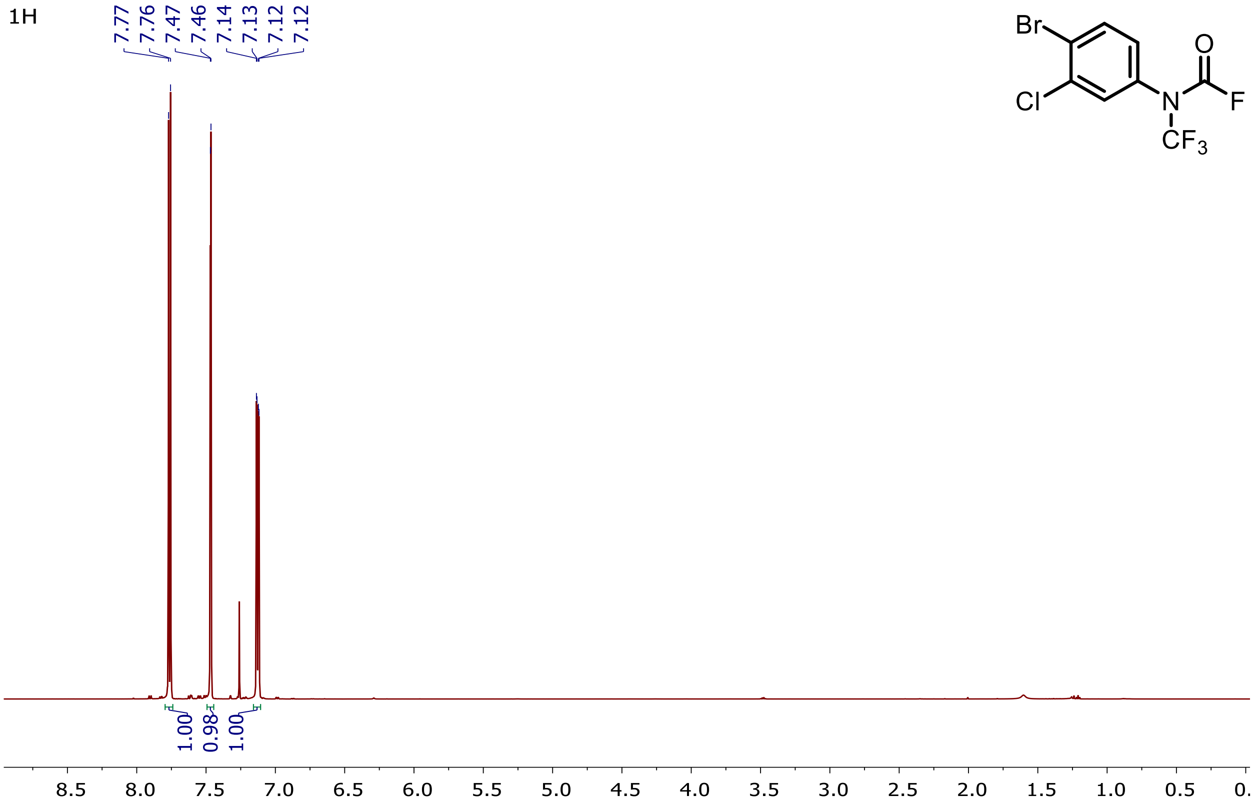
1H
7.77
7.76
7.47
7.46
7.14
7.13
7.12
7.12
1.00
0.98
1.00
8.5 8.0 7.5 7.0 6.5 6.0 5.5 5.0 4.5 4.0 3.5 3.0 2.5 2.0 1.5 1.0 0.5 0.
Br
Cl
O
F
N
CF_3

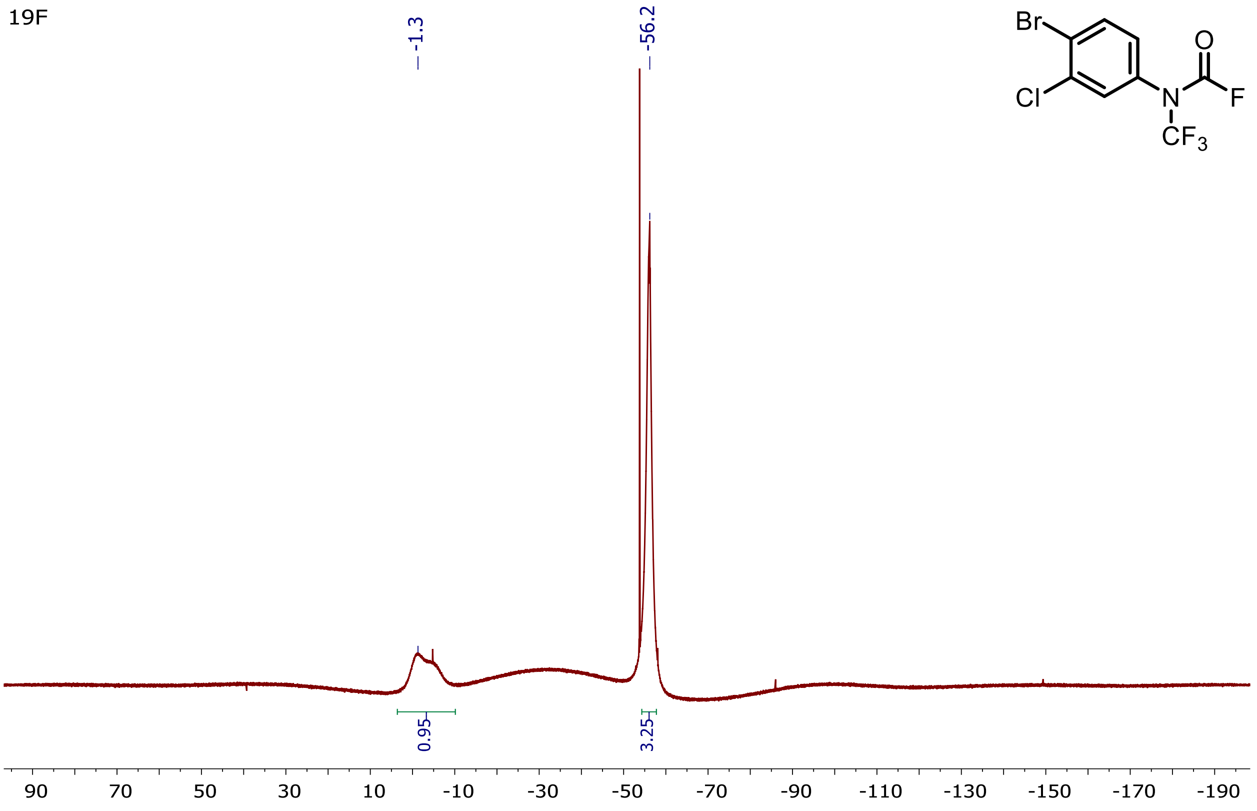
19F
-1.3
-56.2
0.95
3.25
90 70 50 30 10 -10 -30 -50 -70 -90 -110 -130 -150 -170 -190
Br
Cl
O
F
N
CF_3

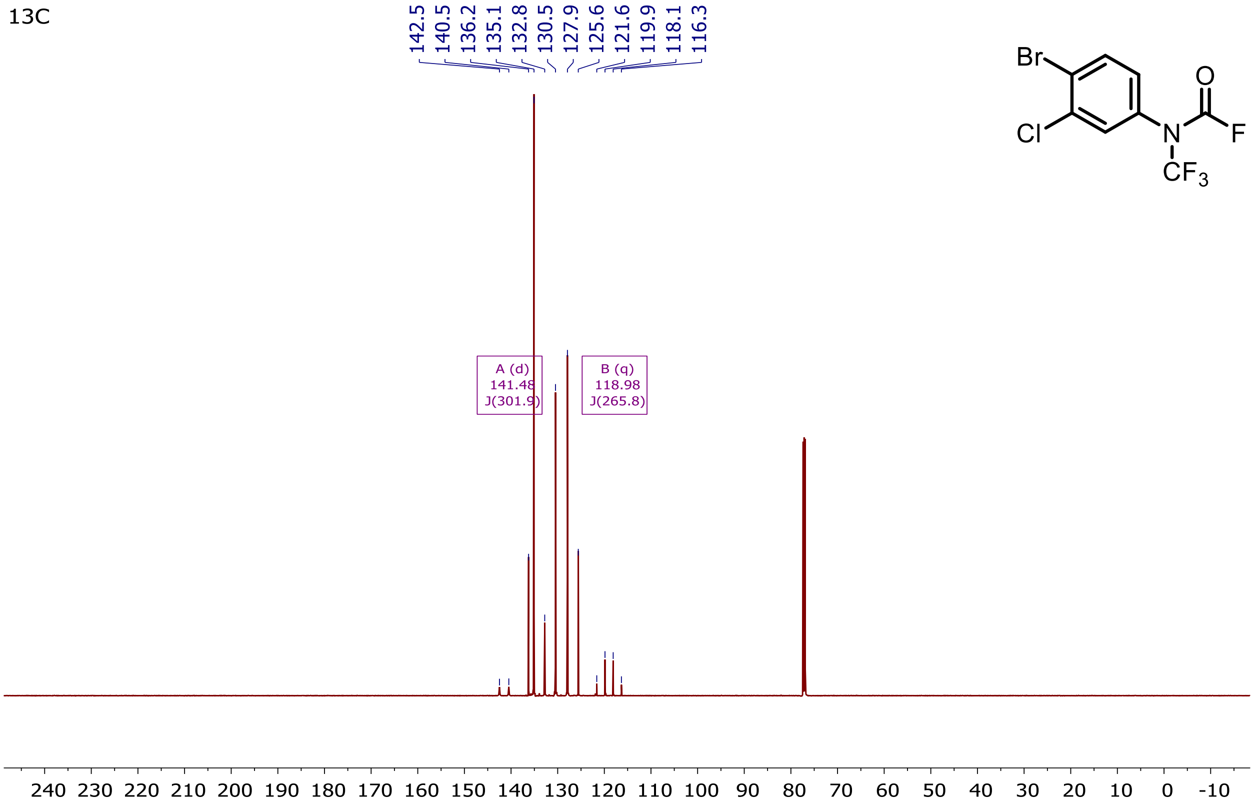
13C
142.5
140.5
136.2
135.1
132.8
130.5
127.9
125.6
121.6
119.9
118.1
116.3
Br
Cl
O
N
F
CF3
A (d)
141.48
J(301.9)
B (q)
118.98
J(265.8)
240 230 220 210 200 190 180 170 160 150 140 130 120 110 100 90 80 70 60 50 40 30 20 10 0 -10

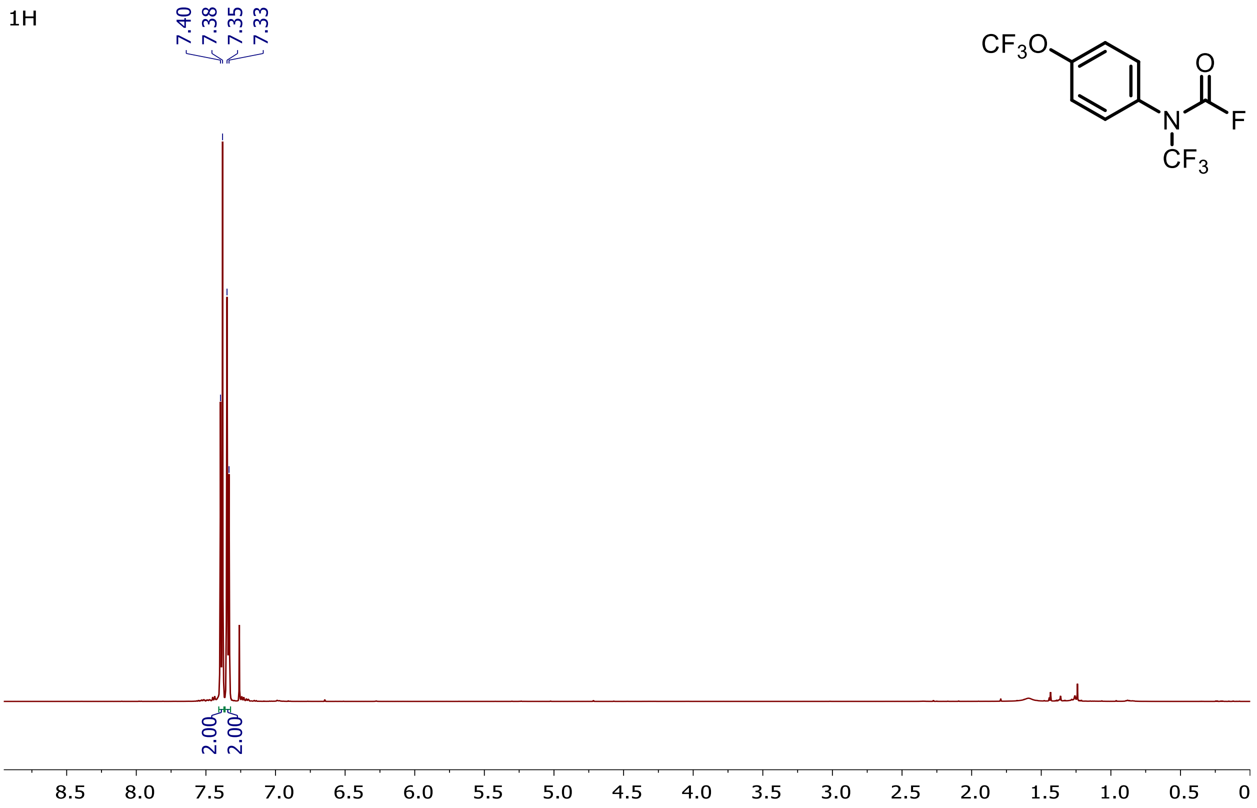
1H
7.40
7.38
7.35
7.33
CF3O
O
N
F
CF3
2.00
2.00
8.5 8.0 7.5 7.0 6.5 6.0 5.5 5.0 4.5 4.0 3.5 3.0 2.5 2.0 1.5 1.0 0.5 0

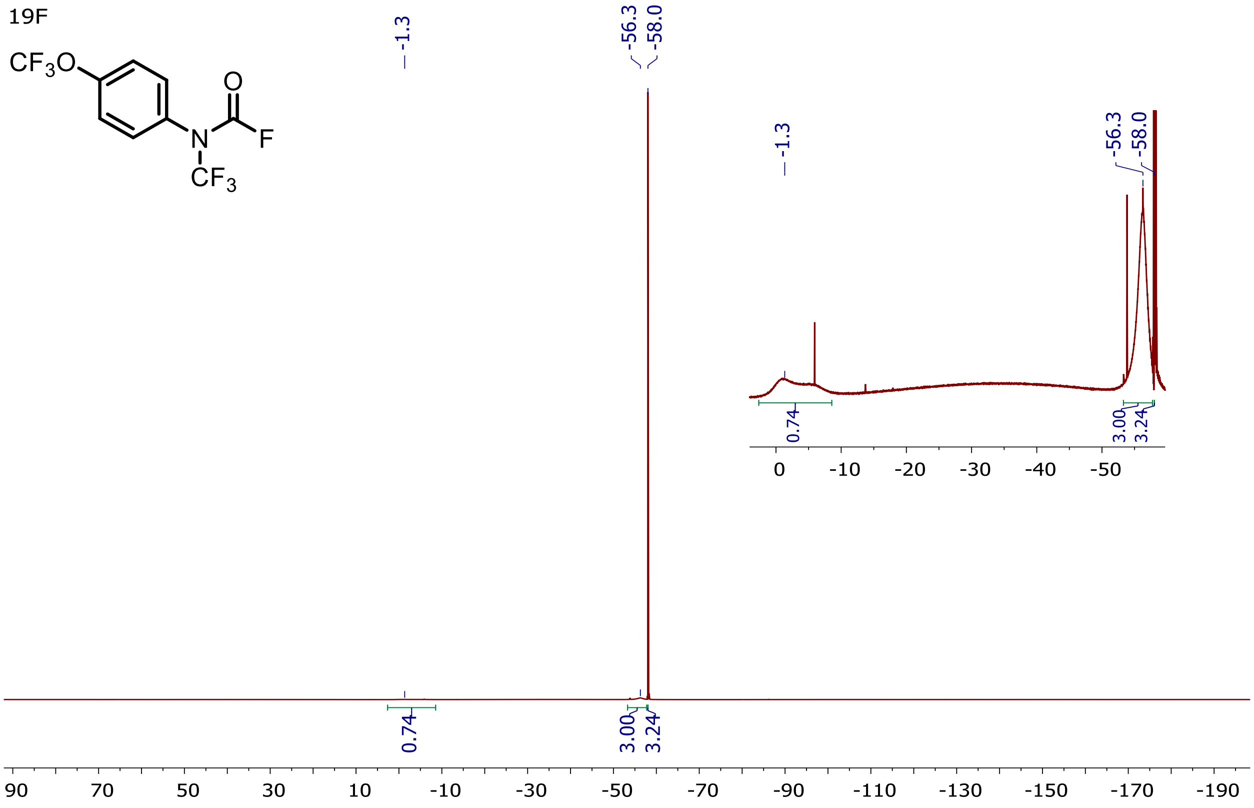
19F
CF3O
O
N
F
CF3
-1.3
-56.3
-58.0
0.74
3.00
3.24
90 70 50 30 10 -10 -30 -50 -70 -90 -110 -130 -150 -170 -190
0 -10 -20 -30 -40 -50

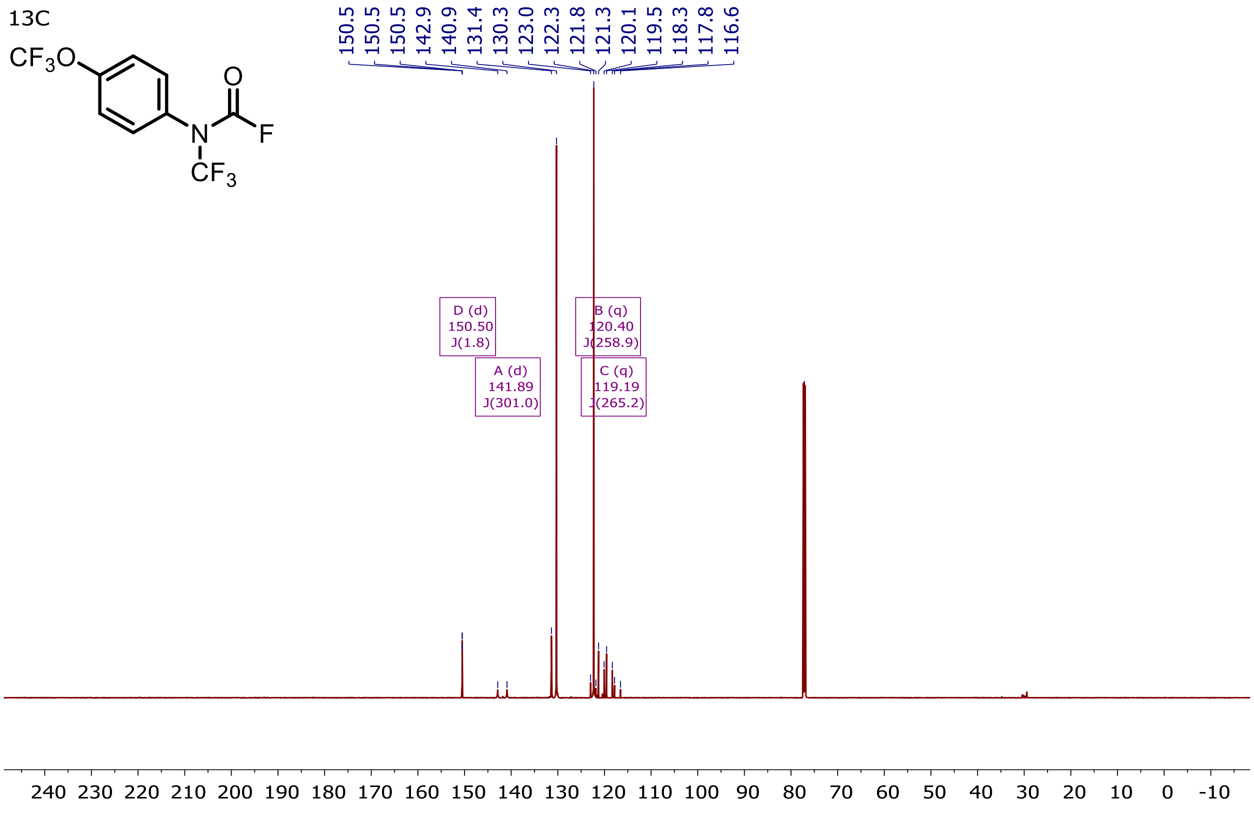
13C
CF3O
O
N
F
CF3
150.5
150.5
150.5
142.9
140.9
131.4
130.3
123.0
122.3
121.8
121.3
120.1
119.5
118.3
117.8
116.6
D (d)
150.50
J(1.8)
A (d)
141.89
J(301.0)
B (q)
120.40
J(258.9)
C (q)
119.19
J(265.2)
240 230 220 210 200 190 180 170 160 150 140 130 120 110 100 90 80 70 60 50 40 30 20 10 0 -10

Isothiocyanates

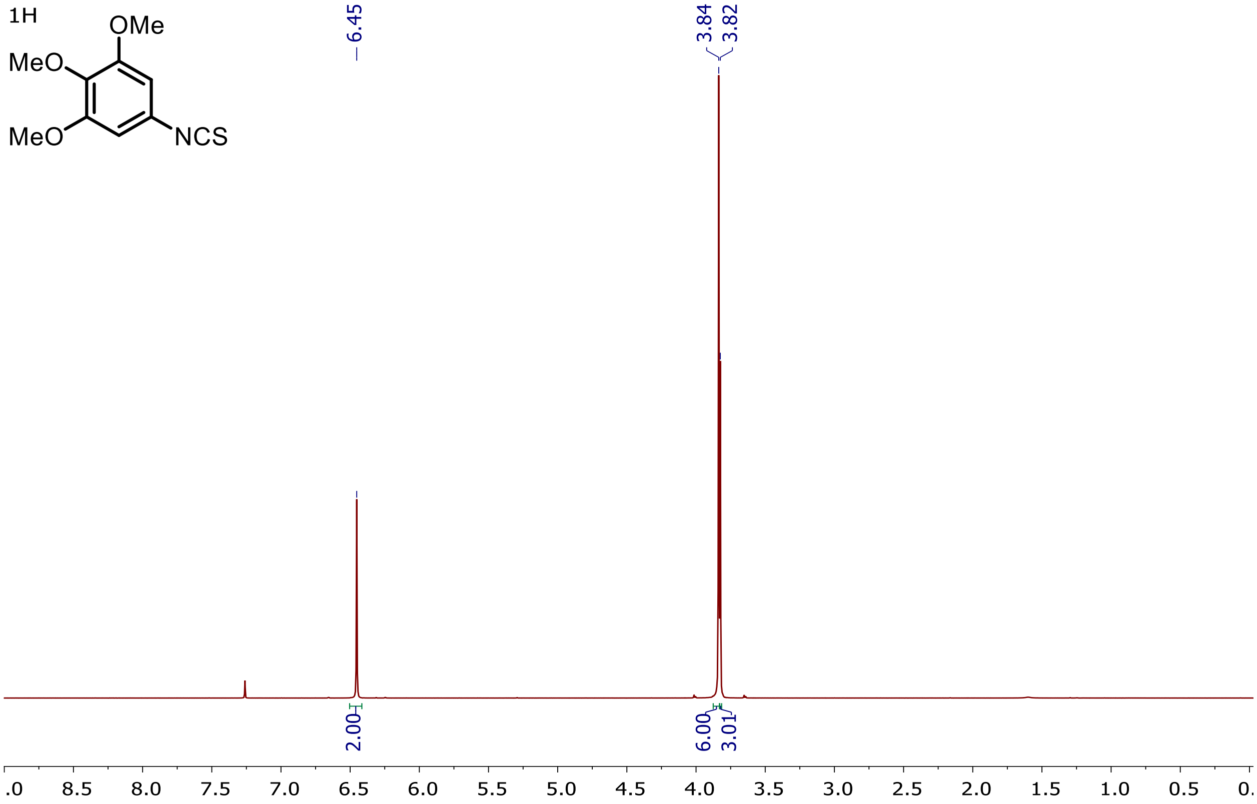

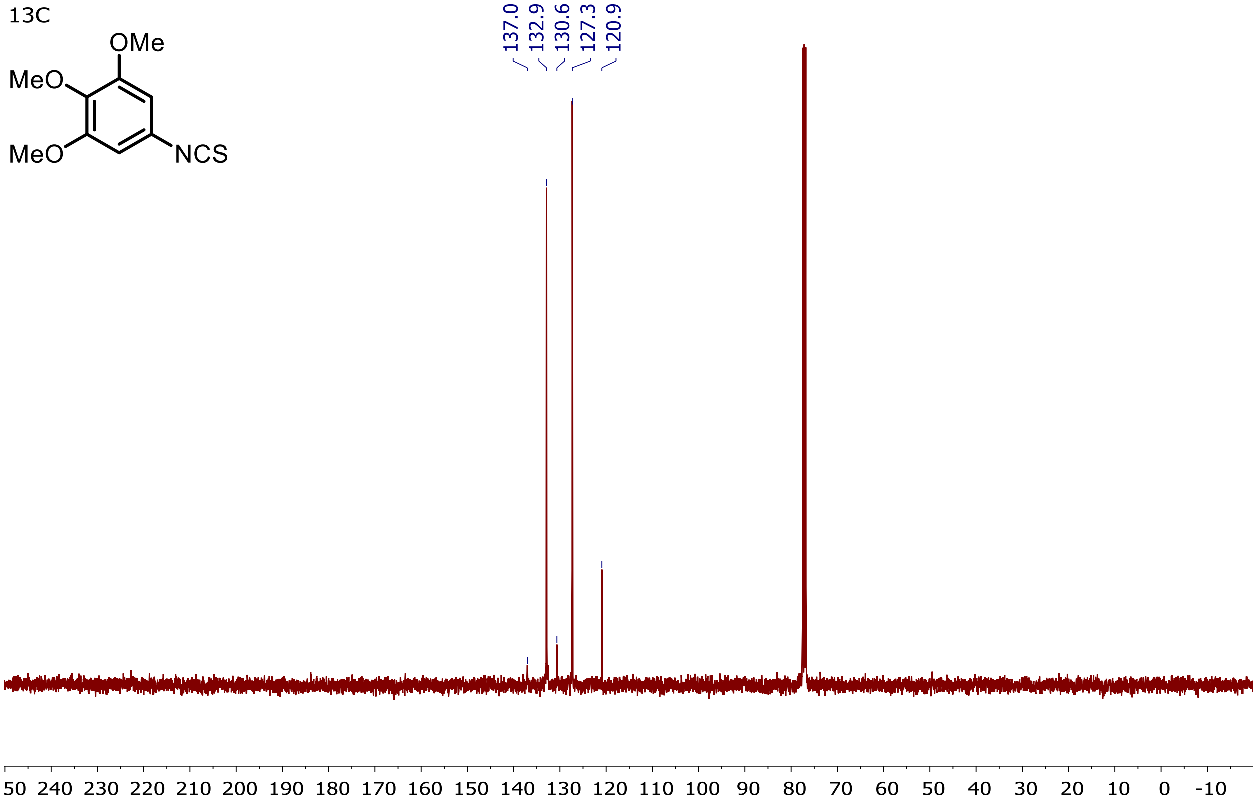

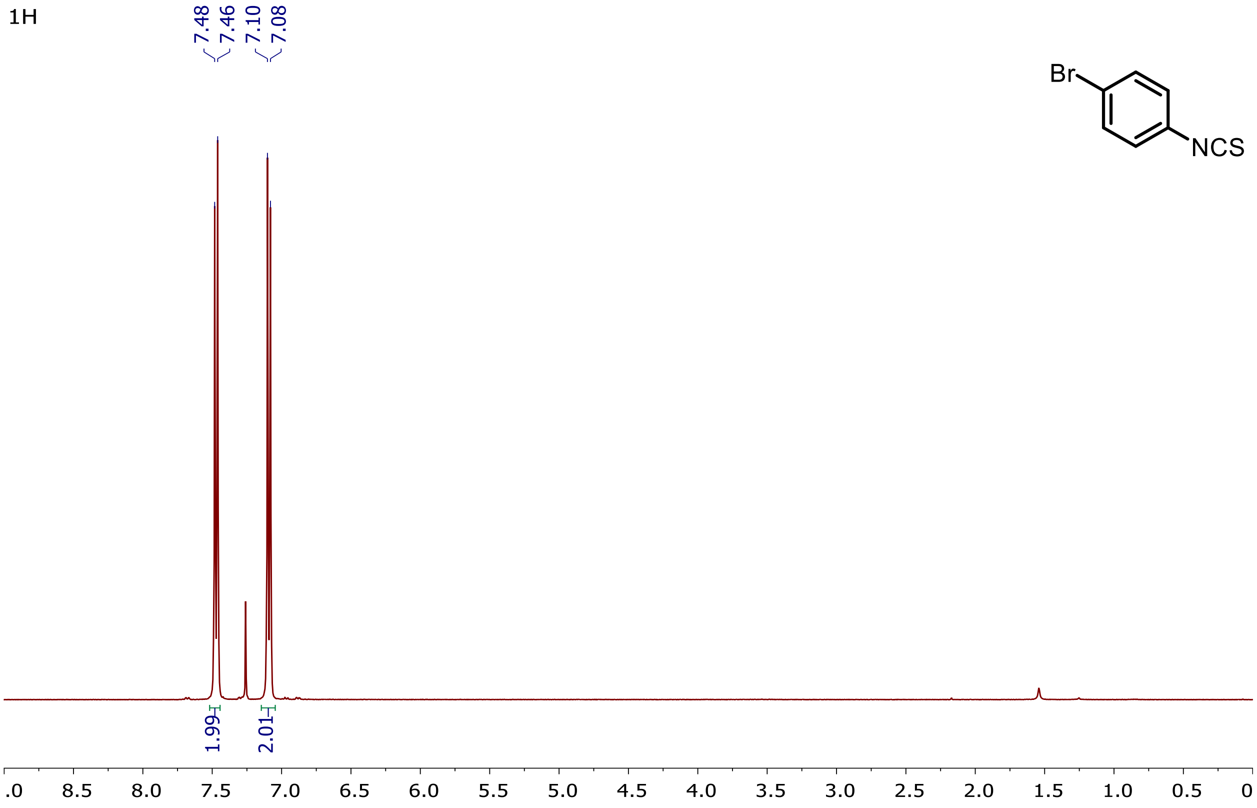

1H
7.48
7.46
7.10
7.08
1.99
2.01
Br
NCS
.0
8.5
8.0
7.5
7.0
6.5
6.0
5.5
5.0
4.5
4.0
3.5
3.0
2.5
2.0
1.5
1.0
0.5
0

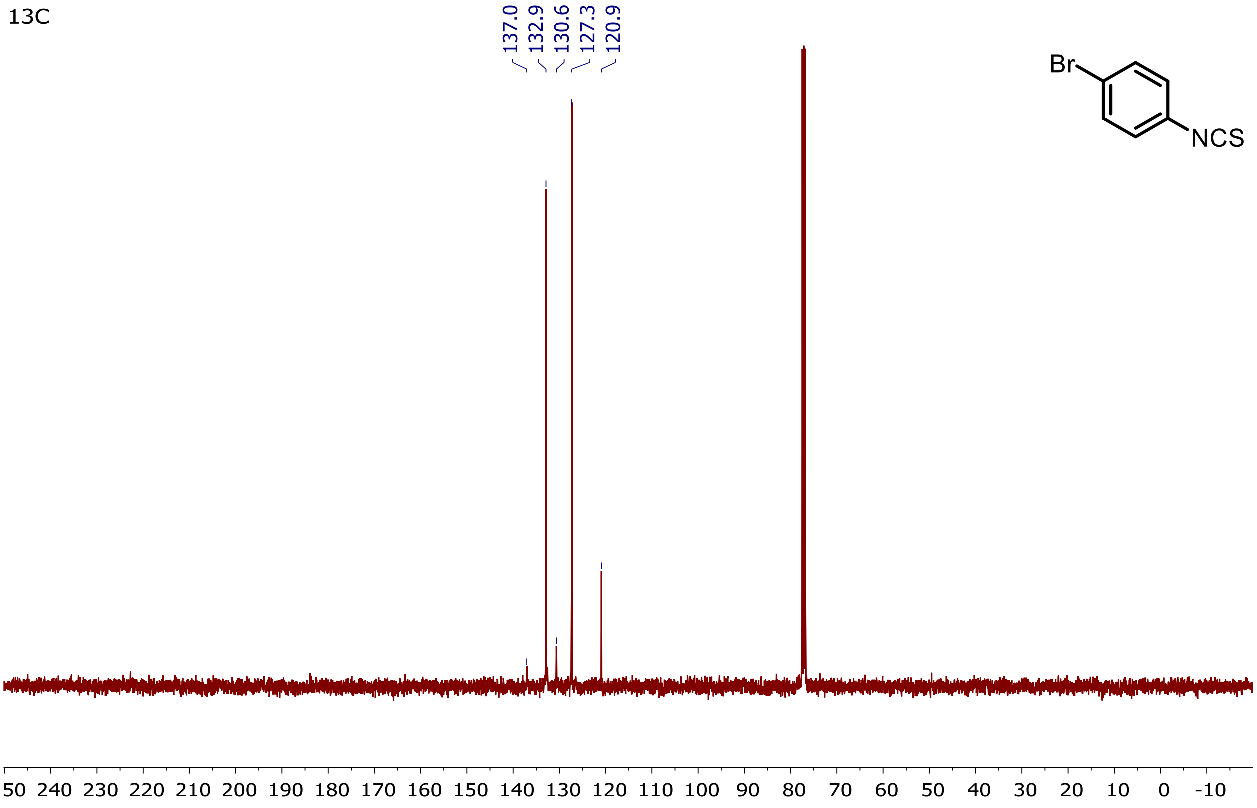

13C
137.0
132.9
130.6
127.3
120.9
Br
NCS
50
240
230
220
210
200
190
180
170
160
150
140
130
120
110
100
90
80
70
60
50
40
30
20
10
0
-10

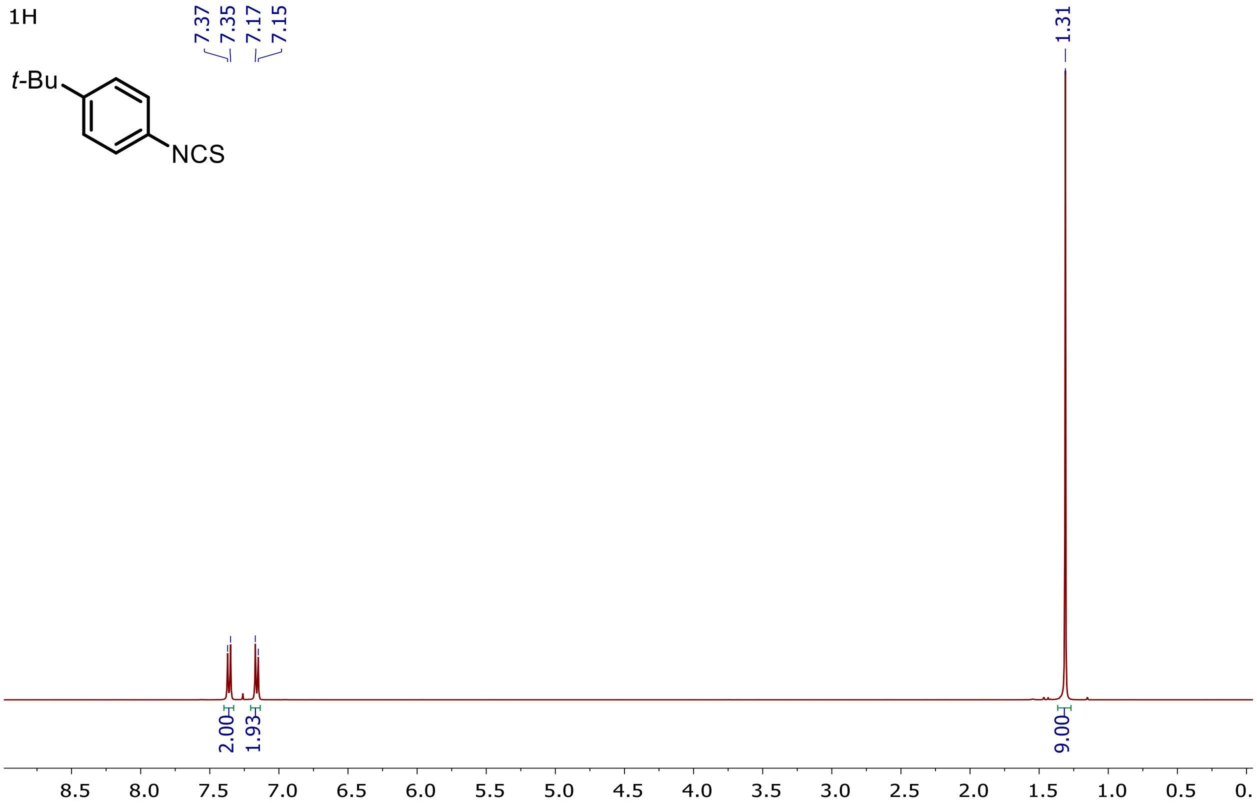

1H
t-Bu
NCS
7.37
7.35
7.17
7.15
1.31
2.00
1.93
9.00
8.5 8.0 7.5 7.0 6.5 6.0 5.5 5.0 4.5 4.0 3.5 3.0 2.5 2.0 1.5 1.0 0.5 0.

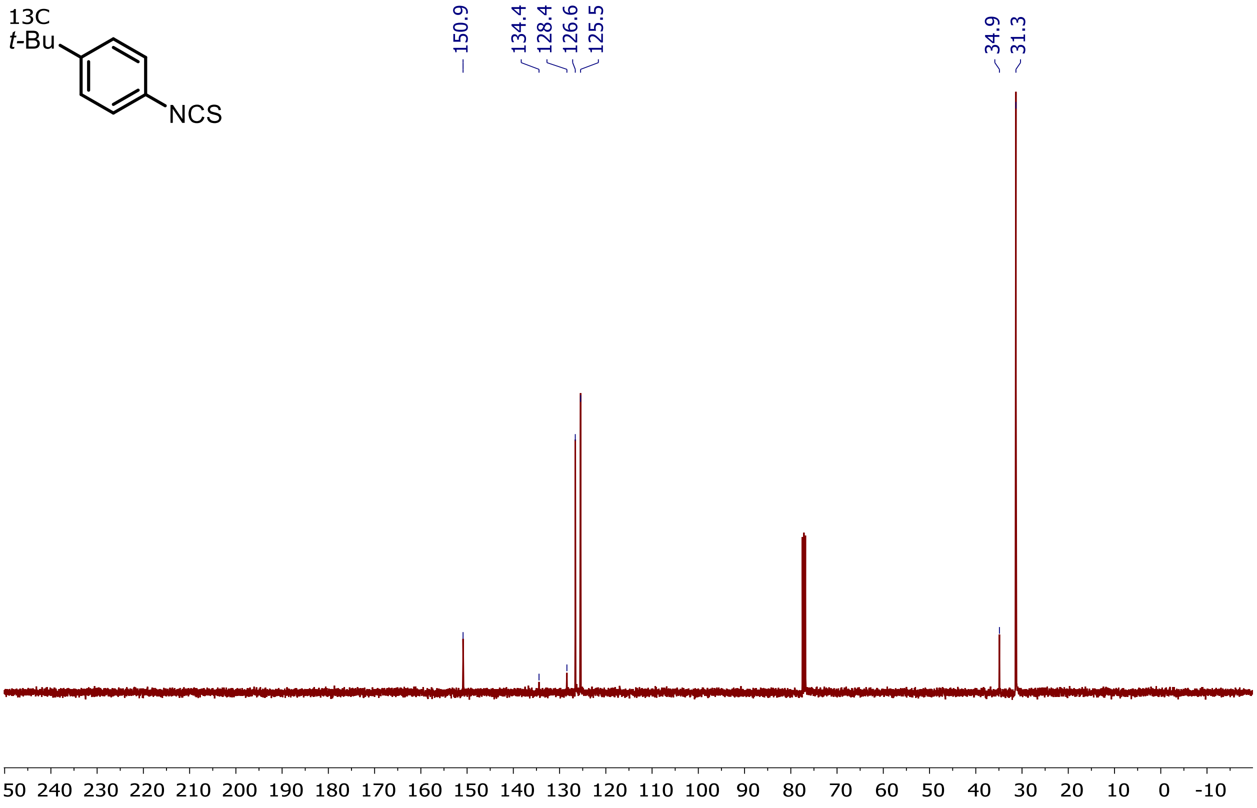

13C
t-Bu
NCS
150.9
134.4
128.4
126.6
125.5
34.9
31.3
50 240 230 220 210 200 190 180 170 160 150 140 130 120 110 100 90 80 70 60 50 40 30 20 10 0 -10

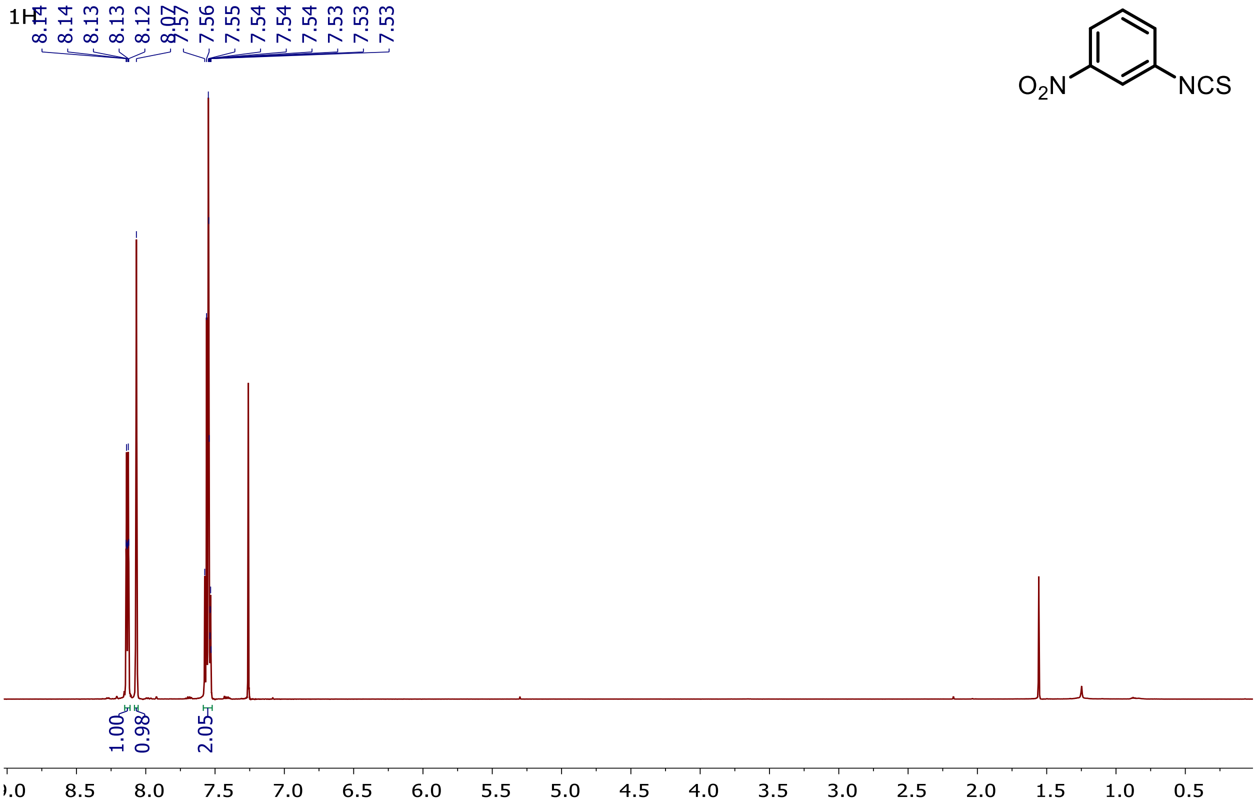

1H
8.14
8.14
8.13
8.13
8.12
8.07
7.57
7.56
7.55
7.54
7.54
7.54
7.53
7.53
7.53
1.00
0.98
2.05
O2N
NCS
9.0 8.5 8.0 7.5 7.0 6.5 6.0 5.5 5.0 4.5 4.0 3.5 3.0 2.5 2.0 1.5 1.0 0.5

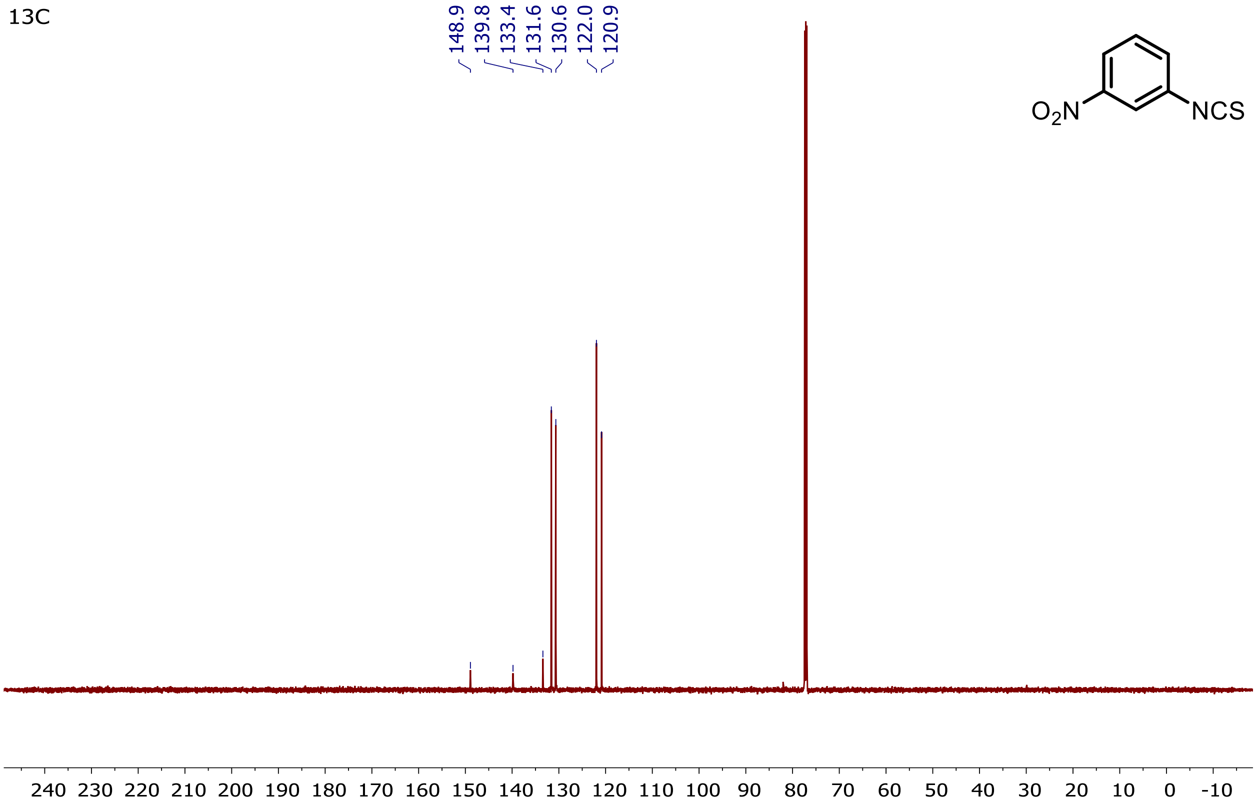

13C
148.9
139.8
133.4
131.6
130.6
122.0
120.9
O2N
NCS
240 230 220 210 200 190 180 170 160 150 140 130 120 110 100 90 80 70 60 50 40 30 20 10 0 -10

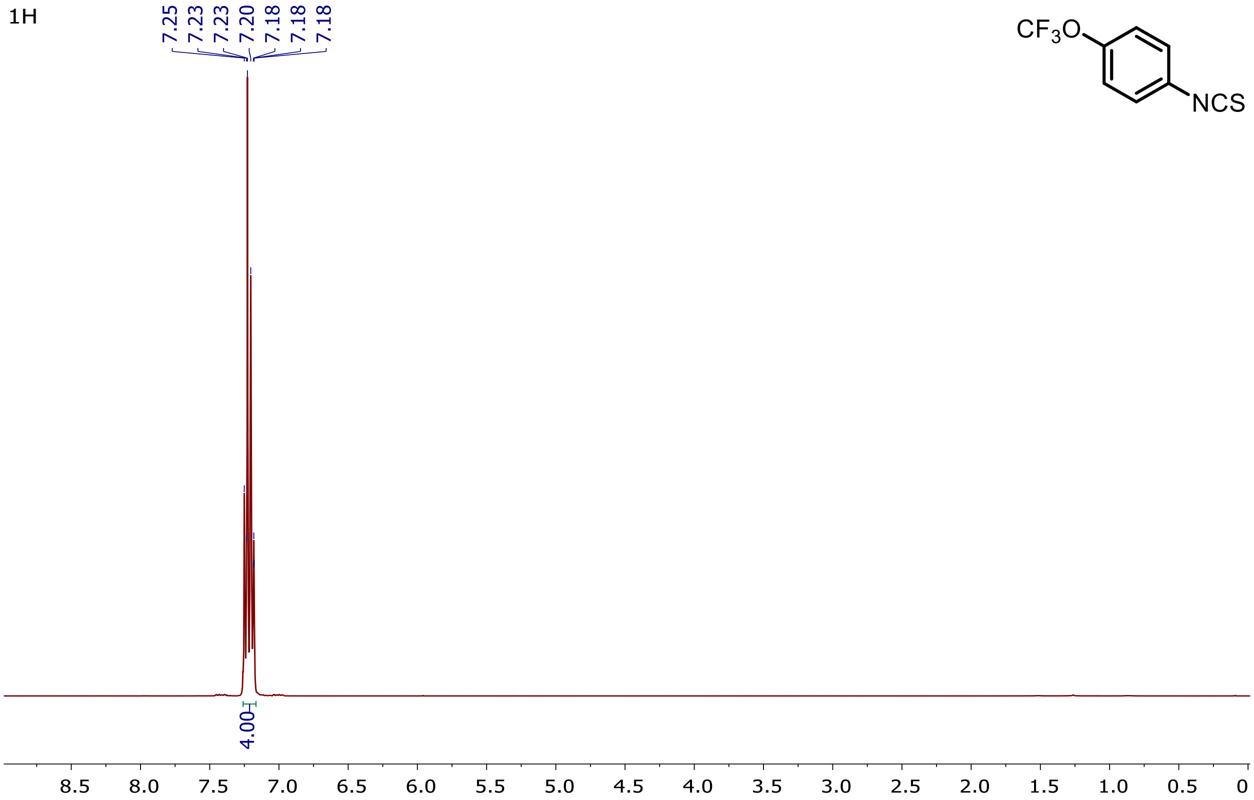
1H
7.25
7.23
7.23
7.20
7.18
7.18
7.18
4.00
8.5
8.0
7.5
7.0
6.5
6.0
5.5
5.0
4.5
4.0
3.5
3.0
2.5
2.0
1.5
1.0
0.5
0
CF3O
NCS

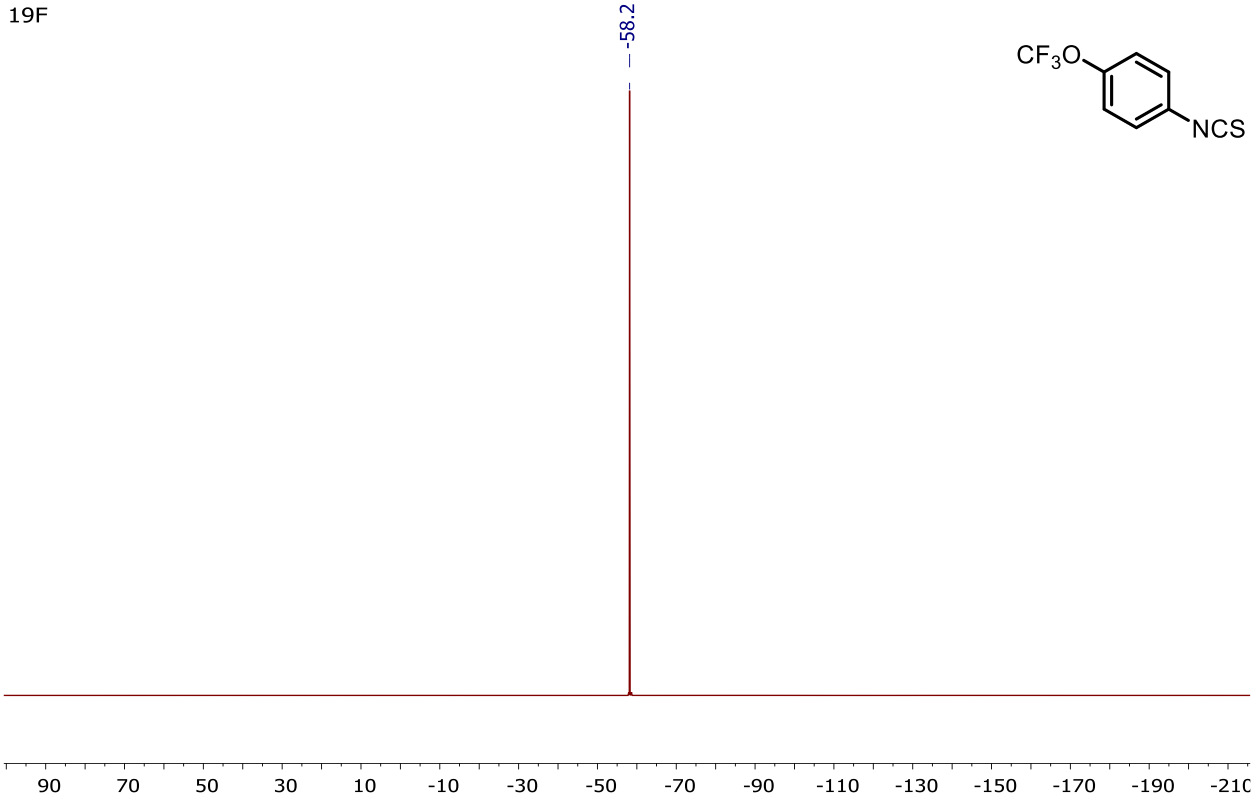
19F
-58.2
90
70
50
30
10
-10
-30
-50
-70
-90
-110
-130
-150
-170
-190
-210
CF3O
NCS

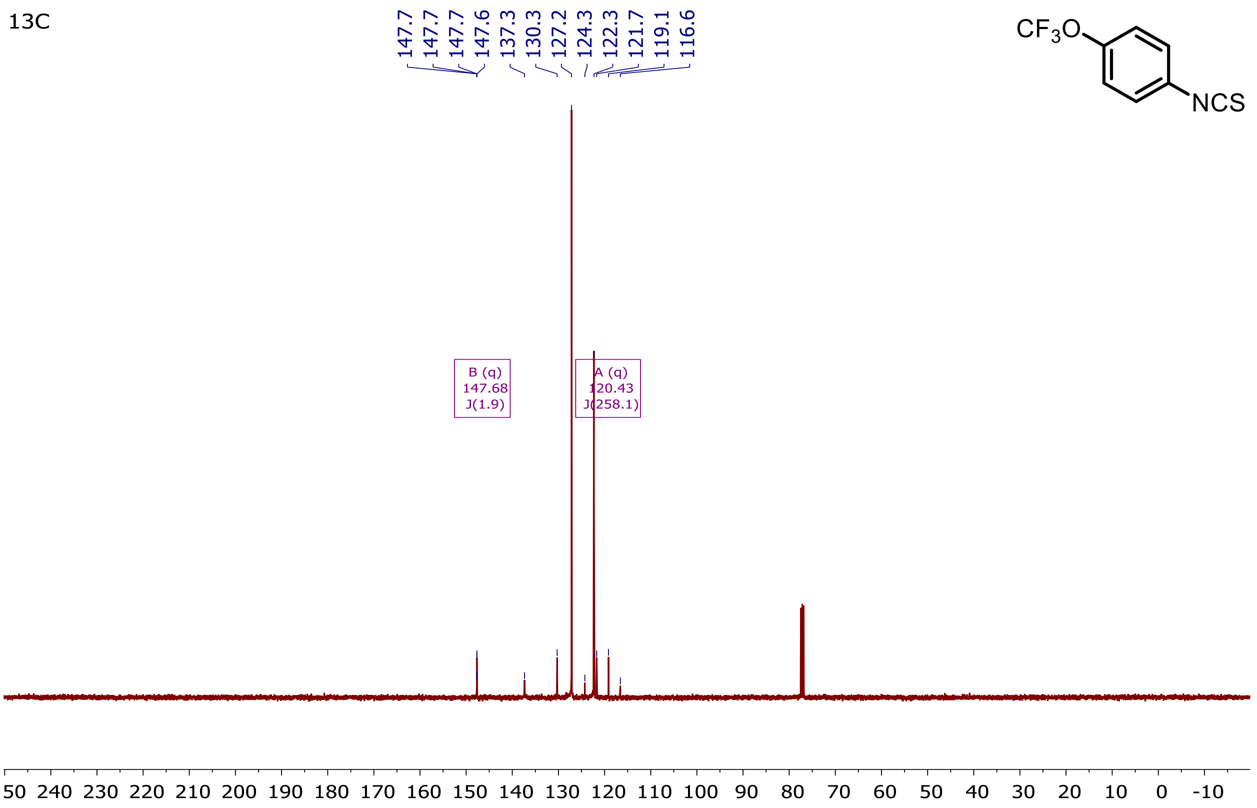
13C
147.7
147.7
147.7
147.6
137.3
130.3
127.2
124.3
122.3
121.7
119.1
116.6
B (q)
147.68
J(1.9)
A (q)
120.43
J(258.1)
CF_3O
NCS
50 240 230 220 210 200 190 180 170 160 150 140 130 120 110 100 90 80 70 60 50 40 30 20 10 0 -10

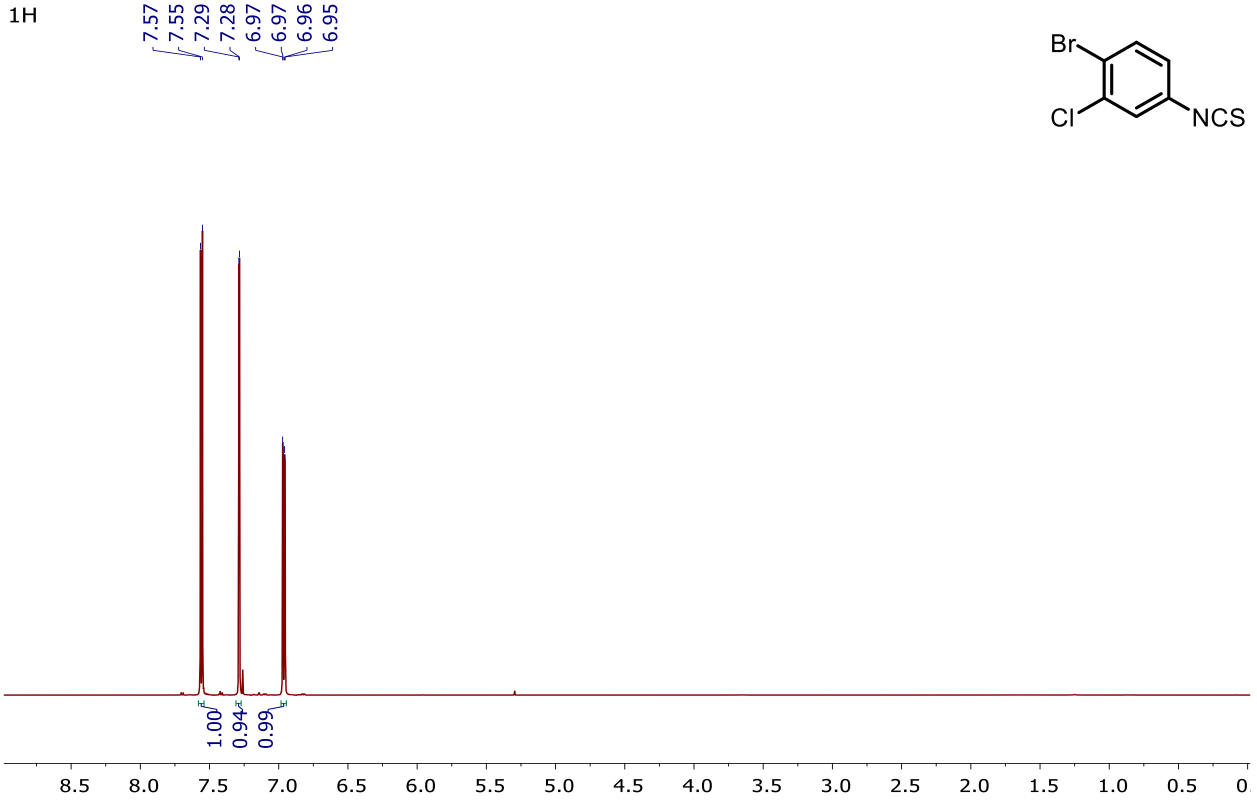
1H
7.57
7.55
7.29
7.28
6.97
6.97
6.96
6.95
Br
Cl
NCS
1.00
0.94
0.99
8.5 8.0 7.5 7.0 6.5 6.0 5.5 5.0 4.5 4.0 3.5 3.0 2.5 2.0 1.5 1.0 0.5 0.

13C

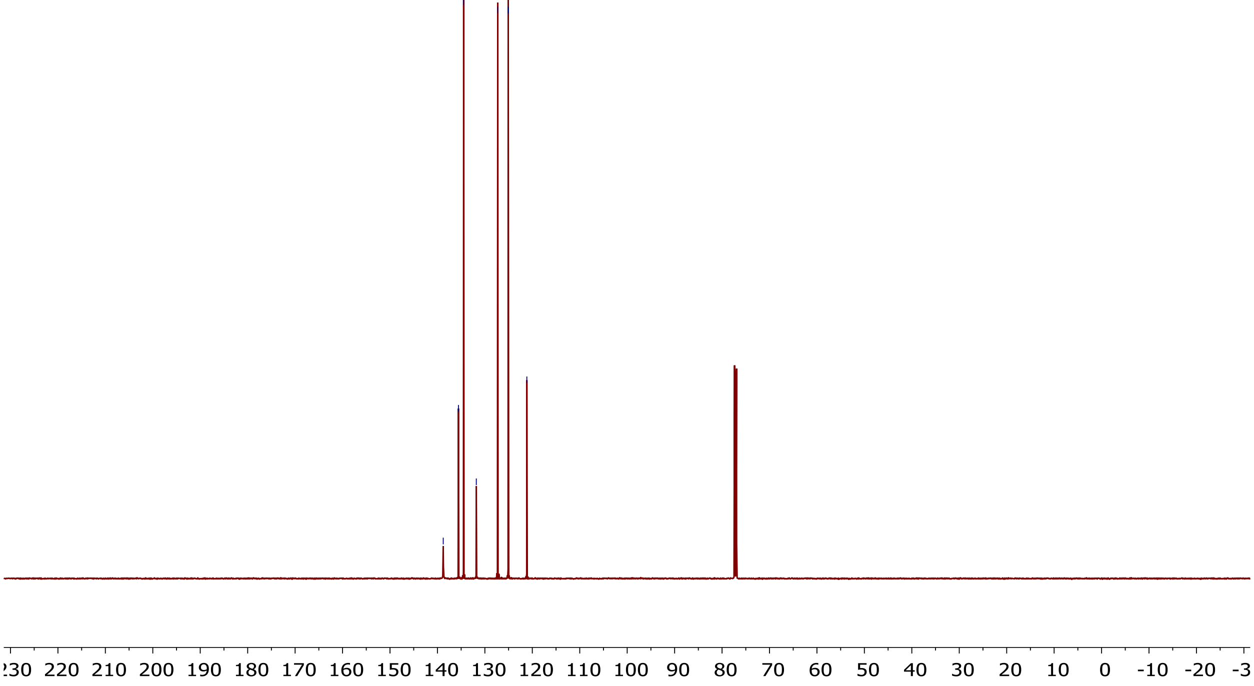
138.8
135.6
134.5
131.8
127.3
125.1
121.2
230 220 210 200 190 180 170 160 150 140 130 120 110 100 90 80 70 60 50 40 30 20 10 0 -10 -20 -3

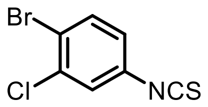
Br
Cl
NCS

7. References

[1] T. Scattolin, S. Bouayad-Gervais, F. Schoenebeck, *Nature* **2019**, *573*, 102-107.

[2] A. V. Ushkov, V. V. Grushin, *J. Am. Chem. Soc.* **2011**, *133*, 10999-11005.

[3] T. Scattolin, E. Senol, G. Yin, Q. Guo, F. Schoenebeck, *Angew. Chem. Int. Ed.* **2018**, *57*, 12425-12429.

[4] S. Zhou, J. Wang, F. Zhang, C. Song, J. Zhu, *Org. Lett.* **2016**, *18*, 2427-2430.

[5] I.-K. Park, S.-E. Suh, B.-Y. Lim, C.-G. Cho, *Org. Lett.* **2009**, *11*, 5454-5456.

[6] I. Kalvet, T. Sperger, T. Scattolin, G. Magnin, F. Schoenebeck, *Angew. Chem. Int. Ed.* **2017**, *56*, 7078-7082.

[7] I. Kalvet, G. Magnin, F. Schoenebeck, *Angew. Chem. Int. Ed.* **2017**, *56*, 1581-1585.

[8] G. M. Sheldrick, *Acta Crystallogr.* **2015**, *A71*, 3-8.

[9] O. V. Dolomanov, L. J. Bourhis, R. J. Gildea, J. A. K. Howard, H. Puschmann, *J. Appl. Crystallogr.* **2009**, *42*, 339-341.

[10] G. M. Sheldrick, *Acta Crystallogr.* **2015**, *C71*, 3-8.

[11] M. J. Frisch, et al., Gaussian 09, Revision D.01, Gaussian, Inc., Wallingford CT (2013).

[12] C. Y. Legault, CYLview, Version 1.0b, Université de Sherbrooke (2009).
